# Recent advances in thiazole-based derivatives targeting diverse anti-inflammatory pathways

**DOI:** 10.1039/d6ra02615j

**Published:** 2026-07-02

**Authors:** Dina H. Dawood, Sami A. Al-Hussain, Thoraya A. Farghaly, Amani M. R. Alsaedi, Magdi E. A. Zaki

**Affiliations:** a Chemistry of Natural and Microbial Products Department, Pharmaceutical and Drug Industries Research Institute, National Research Centre Dokki Giza 12622 Egypt; b Department of Chemistry, Faculty of Science, Imam Mohammad Ibn Saud Islamic University (IMSIU) Riyadh 11623 Saudi Arabia MEZAKI@imamu.edu.sa; c Department of Chemistry, Faculty of Science, Umm Al-Qura University Makkah Saudi Arabia tamohamed@uqu.edu.sa thoraya-f@hotmail.com; d Department of Chemistry, Collage of Science, Taif University P. O. Box 11099 Taif 21944 Saudi Arabia

## Abstract

Various acute and chronic disorders, such as cancer, neurological diseases, cardiovascular disorders, and arthritis, are linked to inflammation. However, the long-term utilization of the available anti-inflammatory medications is frequently restricted by side effects and inadequate selectivity, emphasizing the need for safer and more effective therapeutic agents. A thiazole scaffold emerged as a unique framework in medicinal chemistry owing to its prominent biological effectiveness and favorable pharmacokinetic characteristics. Recently, substantial interest has been gained by various thiazole-based derivatives as potential anti-inflammatory drugs. The thiazole-containing derivatives have exerted prominent anti-inflammatory effects *via* a variety of mechanisms, such as suppression of COX and LOX enzymes, inhibition of pro-inflammatory cytokines such as TNF-α and IL-6, as well as the impediment of the significant inflammatory signaling pathways. It has been demonstrated that the substitution patterns and hybridization of the thiazole scaffold with other bioactive pharmacophores led to the improvement of the anti-inflammatory effectiveness and selectivity. The current review highlights the thiazole-based anti-inflammatory derivatives as promising candidates, encompassing publications from 2020 to 2025 for the discovery of innovative anti-inflammatory therapies by focusing on varied synthetic routes, structure–activity relationships, and mechanistic insights, besides their binding modes within the active site of the target enzymes and cytokines.

## Abstract 2

Various thiazole-based anti-inflammatory agents targeting variety of mechanisms, such as suppression of COX and LOX enzymes, as well as the inhibition of pro-inflammatory cytokines.

## Graphical abstract

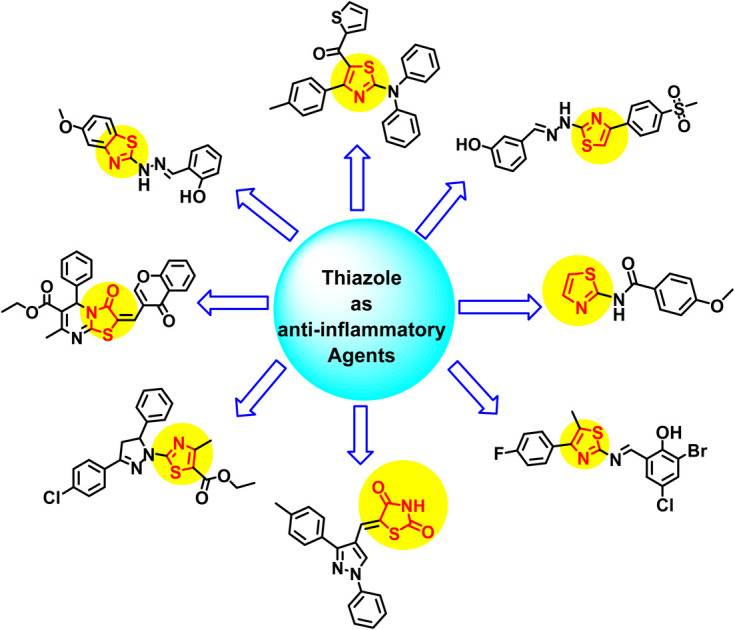

## 1 Introduction

Inflammation is a convoluted process where the body is sensitive to numerous intrinsic triggers such as autoimmune illnesses^1^ or external sources such as bacteria and viruses. It is marked by both systemic effects such as fever, chills, and organ malfunction as well as localized symptoms such as redness, swelling, heat, and pain.^2^ Controlling inflammation is perceived to be of great significance, as inflammation is linked to numerous illnesses, including cardiovascular disorders,^3^ multiple sclerosis,^4^ rheumatoid arthritis,^5^ asthma,^6^ cancer,^7^ psoriasis,^8^ diabetes mellitus,^9^ microbial infections^10^ and Alzheimer's disease.^11^

Inflammatory cytokines that considerably prolong the duration of inflammation include interleukin-6 (IL-6), interleukin-1β (IL-1β) and tumor necrosis factor-alpha (TNF-α).^12,13^ These small proteins produced by macrophages are crucial to the inflammatory process because of their vaso-activating characteristics, which increase the permeability of blood vessels for cells and fluid in the inflamed area.^14,15^

Rheumatoid arthritis, chronic inflammatory disorders in the gut, and acute lung damage might result from either excessive synthesis of pro-inflammatory mediators or insufficient control over them.^16,17^ Finding highly effective drugs and therapies that effectively prevent the production and release of these cytokines is therefore essential. It was discovered that the most effective way to treat inflammation-related illnesses was to either block the expression of inflammatory cytokines or use antibodies to counteract their effects.^18^

TNF-α inhibitors, namely, adalimumab, etanercept, infliximab, golimumab, and certolizumab pegol, are employed to treat autoimmune disorders such as psoriasis, rheumatoid arthritis, Crohn's disease, and ankylosing spondylitis by diminishing inflammation.^19^

The FDA has approved a number of IL-6 inhibitors, such as tocilizumab, sarilumab, and siltuximab, which target the IL-6 receptor and are employed to treat conditions including rheumatoid arthritis, Castleman disease, COVID-19, and giant cell arteritis.

Several IL-6 inhibitors such as tocilizumab, sarilumab, and siltuximab that target the IL-6 receptor and are used to treat diseases such rheumatoid arthritis, Castleman disease, COVID-19, and giant cell arteritis have been approved by the FDA.^20^ Furthermore, IL-1β inhibitors including anakinra, canakinumab, rilonacept target IL-1β and are utilized to treat autoinflammatory syndromes and gout.^21^

Moreover, NO is a special gasotransmitter involved in the processes of vasodilation, non-specific host defense, and acute or chronic inflammation. It is an essential signaling molecule and an endogenous free radical. Nitric oxide synthase (NOS) produces NO biogenetically by oxidizing l-arginine while consuming molecular oxygen, NADPH, and other cofactors.^22,23^ Among the NOS enzymes, iNOS is in charge of producing NO, which modifies inflammatory signals *via* a variety of mechanisms. Therefore, it has been acknowledged that another possible way to treat inflammation is to suppress the expression of iNOS to prevent excessive NO generation.^24^

Chronic inflammation is known to be caused by changes in the arachidonic acid pathway and an excess of prostaglandins (PGs) and leukotrienes (LTs).^25,26^ The up-regulation of COX-2 isoenzyme during inflammation and the significance of leukotrienes and lipoxins produced by lipoxygenases (LOX) in the pathophysiology of bronchial asthma and the development of edema account for these enzymes' critical role in inflammation.^27^

Lipoxygenases (LOX) are a class of non-heme iron-containing enzymes that catalyze lipid peroxidation.^28^ They are classified into 5-LOX, 12-LOX, and 15-LOX based on the peroxidation site of arachidonic acid (AA).^29^ Leukotrienes, eoxins, and lipoxins are examples of pro-inflammatory mediators that are produced from arachidonic acid (AA) by LOX enzymes, which are highly prevalent among mammalian species.^30,31^ One of the main pathways that changes arachidonic acid into 15-hydroxyeicosatetraenoic acid (15-HETE) and other pro-inflammatory mediators is 15-LOX.^32^ Research keeps showing that 15-LOX and its metabolites are involved in a wide range of maladies.^32^ According to recent research, 15-HETE directly contributes to airway inflammation,^33^ diabetic retinopathy,^34^ multiple sclerosis,^35^ osteoarthritis^36^ and many cancers.^37,38^ The only approved LOX inhibitor to date is zileuton, which works by chelating the iron metal found in the LOX enzyme's active site.^39^ Nevertheless, it has been linked to liver damage and has adverse pharmacokinetic characteristics.^40^ Furthermore, the interest in creating novel, safe, and efficient LOX inhibitors has increased due to the rise in demand for anti-LOX treatments. Despite the fact that a number of lipoxygenase inhibitors have been identified,^41^ producing effective inhibitors with advantageous physicochemical characteristics has proven to be challenging.^42^

**Fig. 1:**
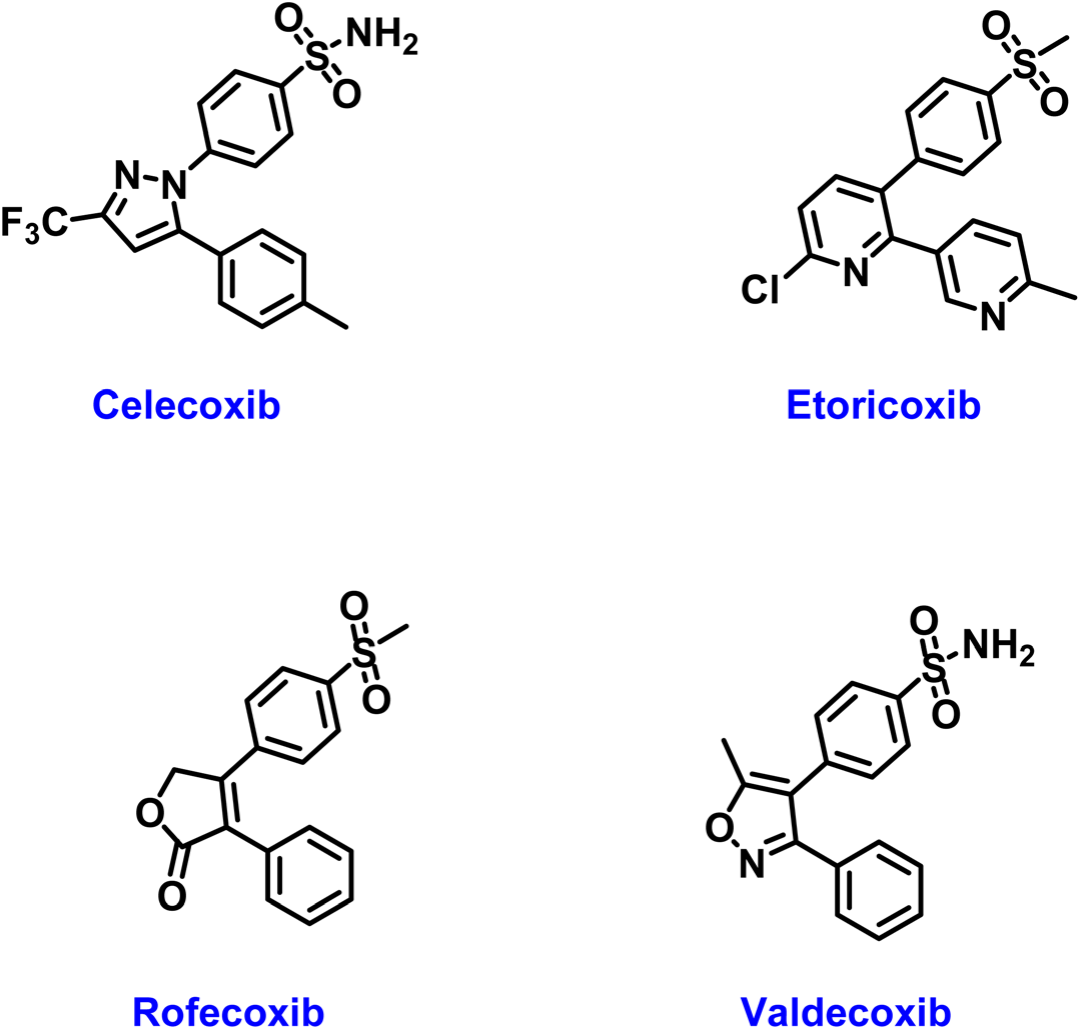
Examples of selective COX-2 inhibitors.

Another metabolic pathway of arachidonic acid includes cyclooxygenases (COX-1 and COX-2), along with LOX enzymes.^43,44^ They metabolize arachidonic acid into prostaglandin, prostacyclin, and thromboxane, all of which are important mediators of inflammation.^44^ The severe side effects of NSAIDs are caused by non-selective inhibition of the constitutive type of cyclooxygenase, *i.e.*, COX-1 alone or simultaneous COX-1 and COX-2 inhibition.^45^ Although PGs, particularly PGE2, induce inflammatory reactions, they play a physiological role in the GI tract's cytoprotective effects and regulate the kidney's renal functions.^46^ Consequently, aspirin and other traditional non-selective NSAIDs that block PGs result in bleeding, renal failure, and stomach ulcers.^47^ Meanwhile, the advantages of selective COX-2 inhibition and the initial interest in this novel class of anti-inflammatory medications have been made clear by growing recognition of the physiological roles of cyclooxygenase (COX), primarily COX-2 enzyme in a range of tissues, including the kidney and stomach.^48^ These findings have enhanced the therapeutic efficacy and decreased the adverse effects of selective COX-2 inhibitors. As a result, selective COX-2 inhibitor medications such as celecoxib and etoricoxib emerged as innovative NSAIDs with a low likelihood of harmful GI side effects (Fig. 1).^49^ However, due to the imbalance in the COX pathway, some coxibs, such as rofecoxib and valdecoxib, encountered market removal due to cardiovascular symptoms (Fig. 1).^50^ Thus, developing an efficient anti-inflammatory medication with fewer adverse effects is still a challenging issue for pharmaceutical chemists.

**Fig. 2:**
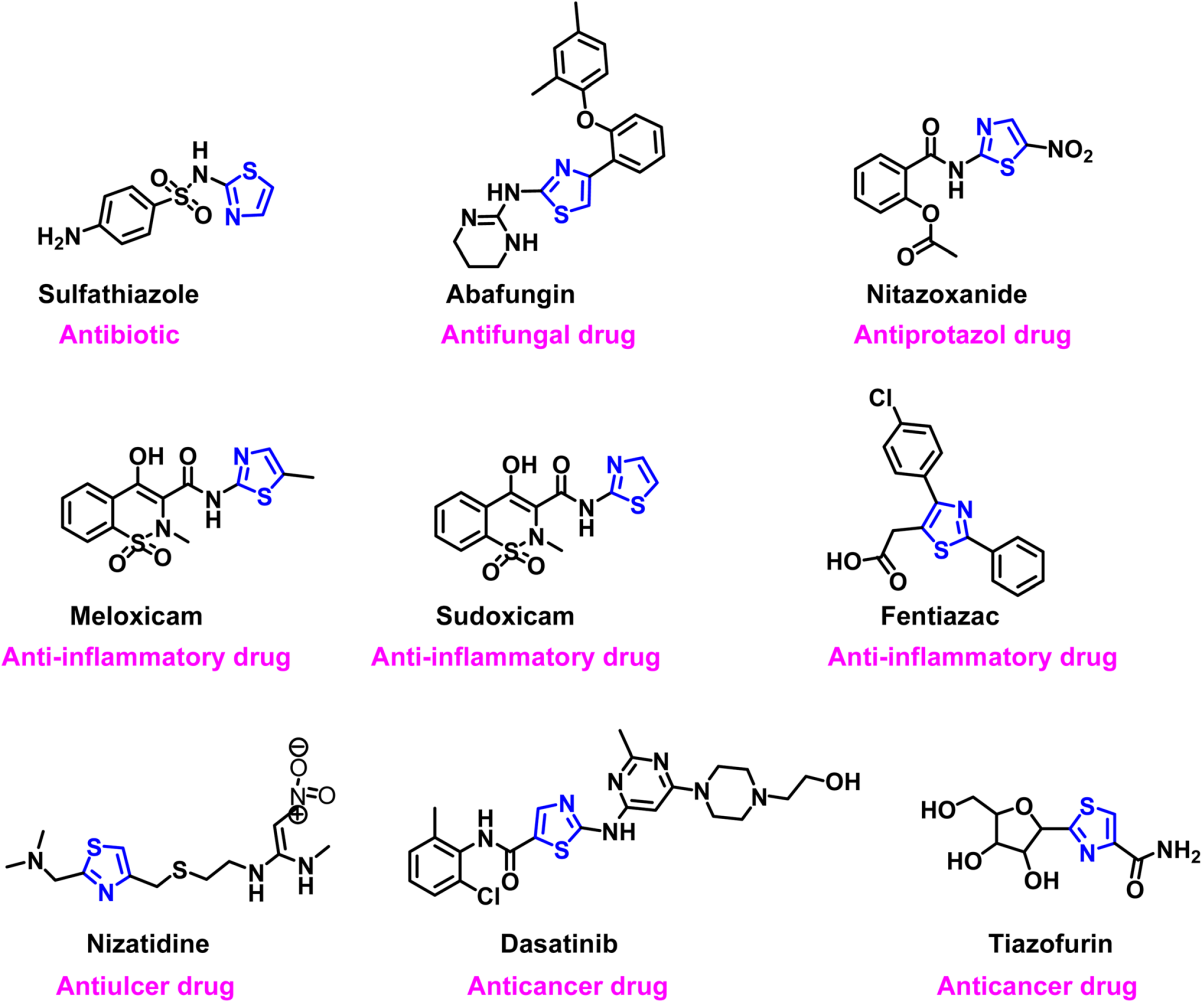
Various thiazole-based drugs.

Thiazole is a remarkable five-membered heterocyclic molecule with a variety of medicinal properties, including anticancer,^51,52^ antimicrobial,^53,54^ anti-HIV,^55^ antioxidant,^56^ and anti-inflammatory effects.^57–59^ Thiazole moiety-containing non-steroidal anti-inflammatory candidates include meloxicam, sudoxicam, and fentiazac. Compared to celecoxib, meloxicam is more cardiovascularly safe and comparatively selective for COX-2 at lower dosages.^60^ Various thiazole-based drugs are shown in Fig. 2.

This article discusses some of the previously documented thiazole derivatives as anti-inflammatory agents targeting distinct mechanisms such as LOX and COX enzymes as well as numerous cytokines and inflammatory mediators, in addition to their reported docking studies. The thiazole derivatives listed below are categorized based on the contained substituents; moreover, the hybridization or the fusion of the thiazole scaffold with various biologically active heterocyclic rings was introduced. Furthermore, the structure–activity relationships and the reported synthetic routes of thiazole derivatives are highlighted.

## 2 Thiazole-based derivatives as anti-inflammatory agents

### 2.1 Tri-substituted thiazole-based derivatives as anti-inflammatory agents

**Fig. 3:**
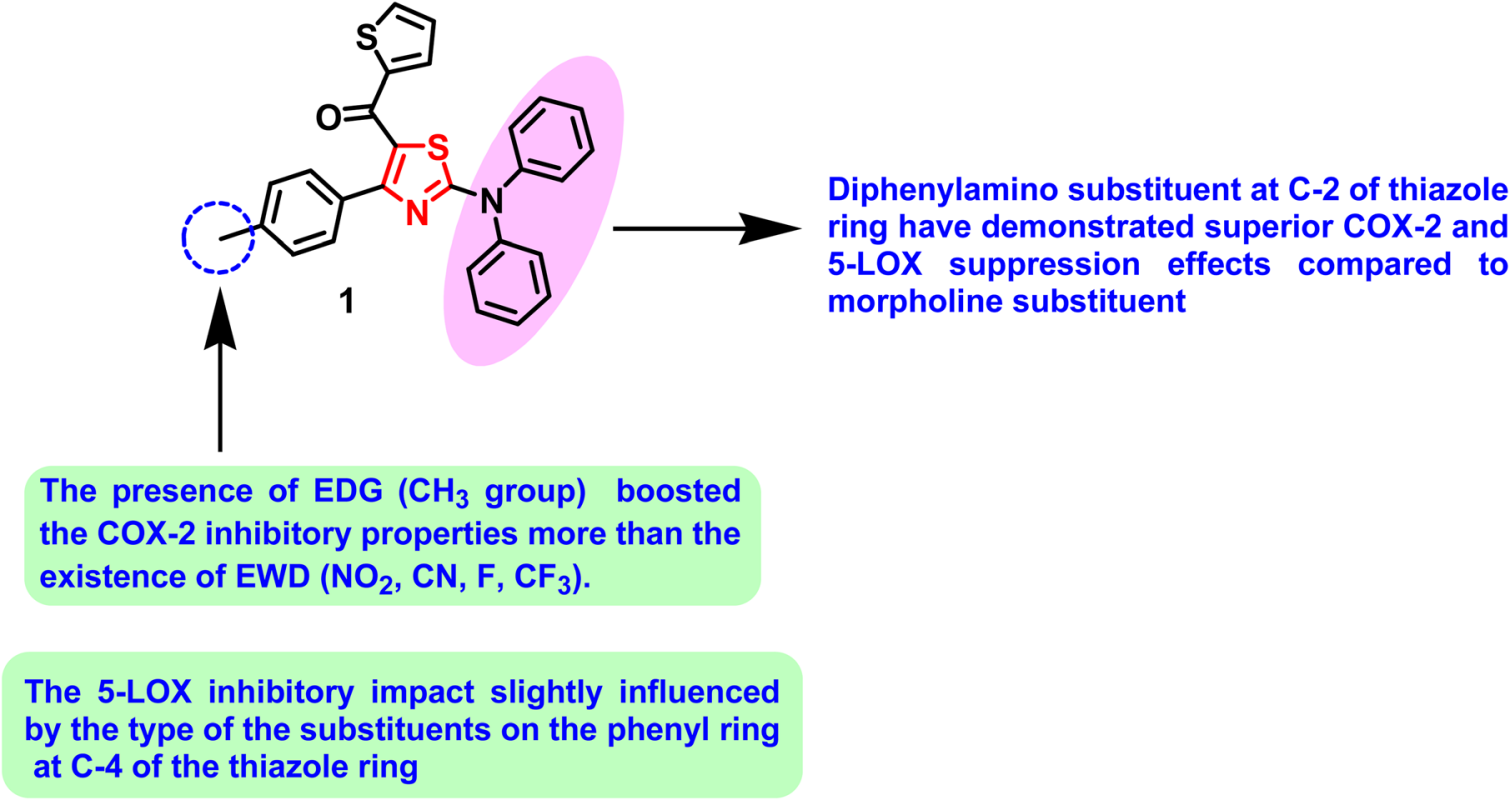
Structure of the tri-substituted thiazole derivative 1 as an anti-inflammatory agent targeting dual 5-LOX and COX-2 enzymes.

An innovative tri-substituted thiazole derivative 1 stood out as an eminent dual 5-LOX and COX-2 suppressor (IC_50_ = 0.38 and 0.09 µM, respectively), relative to zileuton and etoricoxib (IC_50_ = 0.14 and 0.07 µM, respectively). Derivative 1 significantly reduced edema (60.82%) compared to indomethacin (53.21%) after six hours. Over the course of the trial, the anti-inflammatory impact of derivative 1 increased gradually and remarkably in a dose-dependent manner. The histological analysis disclosed that derivative 1 had a normal histology, assuring both its potential therapeutic benefit and gastrointestinal safety. Furthermore, derivative 1 reduced the production of LTB4 at IC_50_ = 0.28 µM and PGE2 at IC_50_ = 0.48 µM. Derivatives with diphenylamino substituent at C-2 of the thiazole ring have demonstrated superior COX-2 suppression effect compared to morpholine substituent. Diminished COX-2 inhibition impact was seen by the absence of the substituents on the phenyl ring at C-4 of the thiazole scaffold, whereas the presence of EDG (CH_3_ group) on the phenyl ring at C-4 of thiazole boosted the COX-2 inhibitory properties more than the existence of EWD (NO_2_, CN, F, and CF_3_). Similarly, 5-LOX suppression effectiveness was enhanced by the presence of a diphenylamino substituent at C-2 of the thiazole ring relative to the morpholine substituent. Conversely, 5-LOX inhibition is slightly influenced by the type of substituents on the phenyl ring at C-4 of the thiazole ring. In the COX-2 active site, derivative 1 demonstrated the optimal binding position with a binding energy of −7.54 kcal mol^−1^. The carbonyl group's oxygen atom established a H-bond with His351. The Pro191, His90, Ser353, Thr94, Gly354, Pro514, Gln192, Asp515 and Tyr355 residues formed a hydrophobic pocket filled with a diphenyl amino group. Ser581 participated in a hydrophobic interaction with the thiophenyl ring. In a similar manner, Gln350 and His356 were interacting and enclosed the 4-methyl phenyl ring in a hydrophobic cavity. Derivative 1's binding energy at the 5-LOX active site was −6.99 kcal mol^−1^. Gln417 and Trp147 contributed to two H-bonds with the S atoms of thiophene and thiazole rings, respectively. Additionally, pi–sulfur interaction was seen between derivative 1 and Met145 (ref. 61) (Fig. 3).

**Scheme 1:**
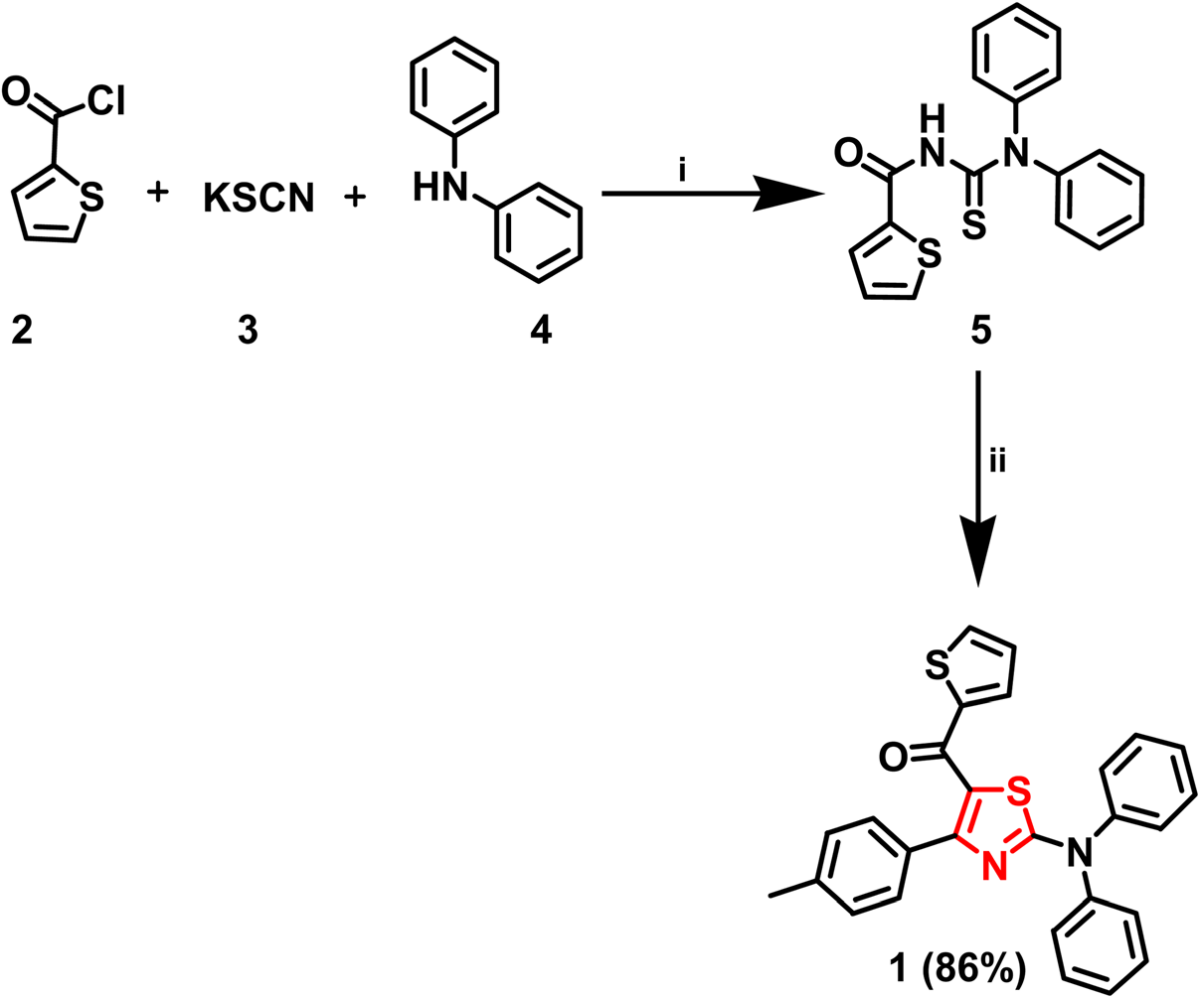
Synthesis of the tri-substituted thiazole derivative 1. Reagents and conditions: (i) water, TBAF, stirred for 30 min at RT; (ii) water, TBAF, 4-methylphenacylbromide, heat, 80 °C, 2 h.

A modified green synthetic approach is used to achieve target 1, as shown in Scheme 1. The reaction was conducted in a single pot using tetrabutylammonium fluoride (TBAF) and water.^61^

**Fig. 4:**
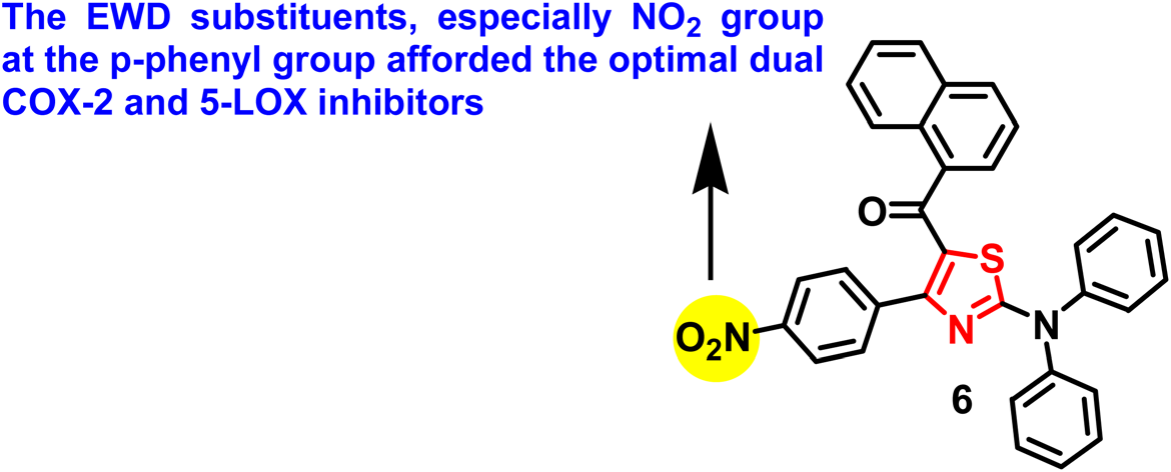
Structure of the tri-substituted thiazole derivative 6 as an anti-inflammatory agent targeting dual 5-LOX and COX-2 enzymes.

Moreover, further modifications were performed on the previous derivative 1*via* replacing the thiophene with a naphthyl ring to furnish novel thiazole candidate 6 that presented outstanding dual 5-LOX and COX-2 suppression impact (IC_50_ = 0.29 and 0.07 µM, respectively) with respect to zileuton and etoricoxib (IC_50_ = 0.15 and 0.07 µM, respectively). The EWD substituents, especially NO_2_ group at the p-phenyl group afforded the optimal dual COX-2 and 5-LOX inhibitors. Moreover, derivative 6 drastically diminished the production of PGE2 and LTB4 (IC_50_ = 0.41 and 0.25 µM, respectively). Meanwhile, etoricoxib and zileuton displayed PGE2 and LTB4 suppression effectiveness with IC_50_ = 0.46 and 0.45 µM, respectively. Additionally, at a dose of 10 mg kg^−1^, the derivative 6 produced a 53% decrease in edema similar to that of indomethacin. An oral dosage of 6 (10 and 50 mg kg^−1^) revealed a normal GI mucosal stomach architecture. On the other hand, rats' stomach mucosa showed extreme ulcerative susceptibility when indomethacin was administered at a dose of 10 mg kg^−1^, causing mucosal aberration and ulceration. At a COX-2 binding site, the optimal binding conformation was shown by derivative 6 (binding energy = −8.87 kcal mol^−1^). Derivative 6 demonstrated the creation of two H-bonds between Gln192 and His356 with a S atom of the thiazole ring and an O atom of the NO_2_ group, respectively, in addition to van der Waals interaction with Ser581. Moreover, His351 Gln354, Ser581, Gln350 and Tyr355 surrounded the naphthyl moiety. Derivative 6's docking simulations at the 5-LOX binding site produced a binding energy of −6.68 kcal mol^−1^. Furthermore, O atoms of the NO_2_ and C=O groups developed H-bonds with Arg411 and Trp147, respectively, while the naphthoyl group was involved in pi–alkyl, pi–anion and van der Waals interactions with the active site^62^ (Fig. 4).

**Scheme 2:**
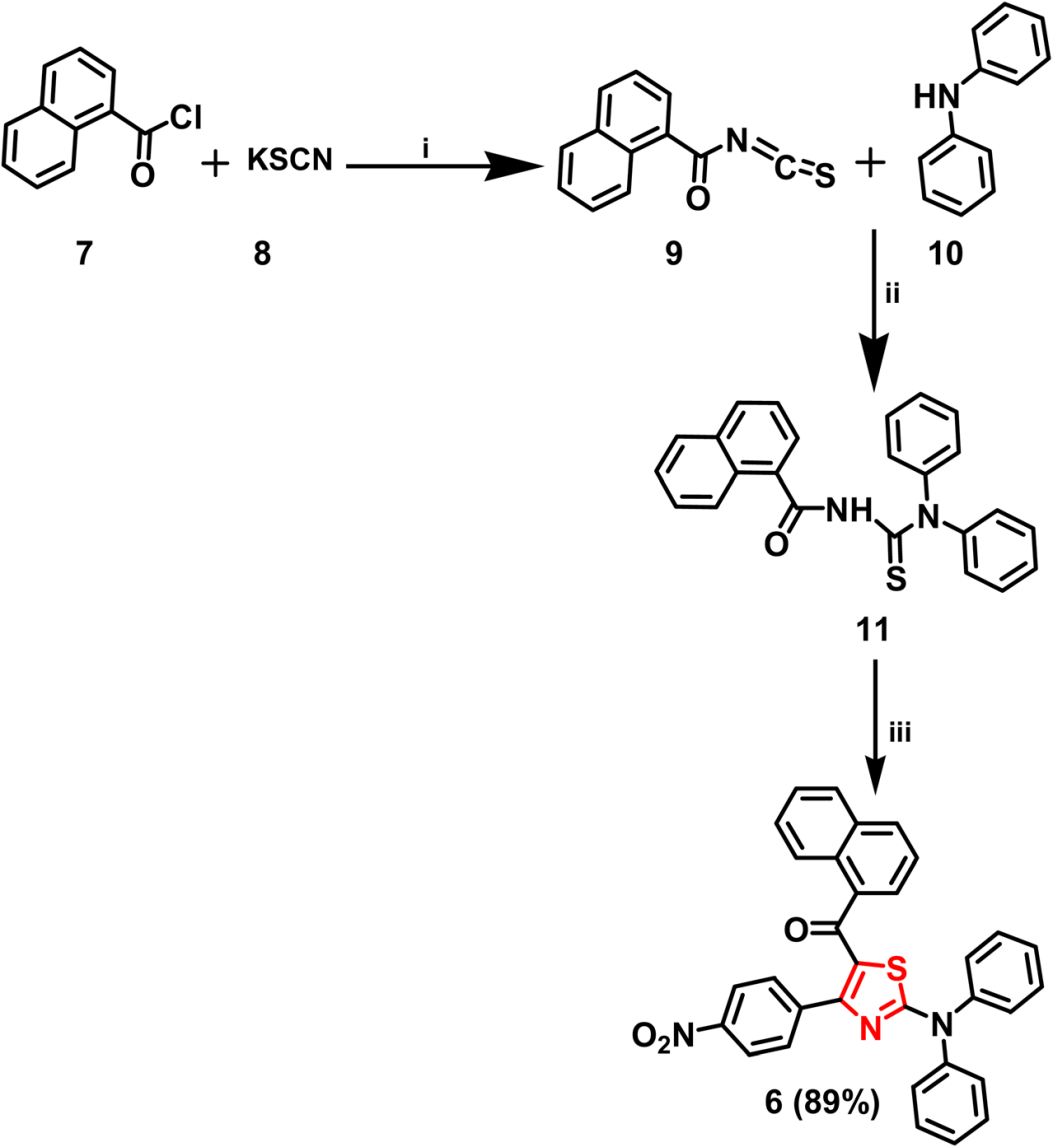
Synthesis of the tri-substituted thiazole derivative 6. Reagents and conditions: (i) water, toluene, TBAF, stirred for 1 h at RT; (ii) water, toluene, TBAF, stirred for 1 h at RT; (iii) TBAF, 4-nitrophenacylbromide, SFC, heat (oil bath), 80 °C, 1.5 h.

At first, 1-naphthoyl chloride 7 was treated with potassium thiocyanate 8 in a toluene-water system containing tetrabutylammonium fluoride (TBAF) to form naphthalene-1-carbonyl isothiocyanate 9, which underwent a reaction with diphenylamine 10 to yield 1-naphthoyl thiourea 11. The target thiazole 6 was accomplished by the reaction of 1-naphthoyl thiourea 11 with 4-nitro phenacylbromide in the presence of TBAF (Scheme 2).^62^

### 2.2 Thiazole-naphthalenone hybrids as anti-inflammatory agents

**Scheme 3:**
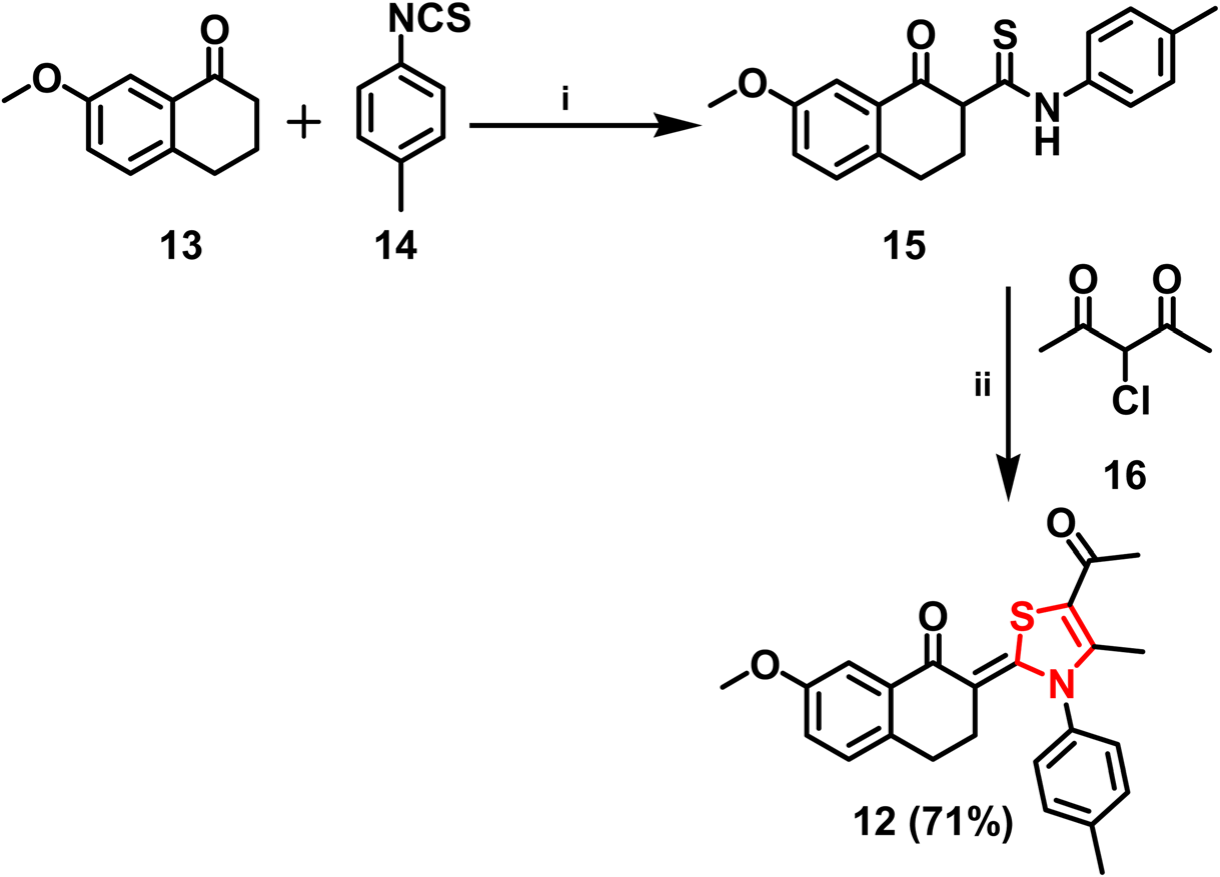
Synthesis of the thiazole-naphthalenone hybrid 12. Reagents and conditions: (i) NaH, DMF, stirring, 0 °C, 2 h; (ii) DMF, stirring, 25 °C, 4 h.

Yadav and Chaudhary^63^ reported the synthesis and assessment of the *in vitro* anti-inflammatory efficiency of new thiazole-naphthalenone hybrids against LPS-induced inflammation in RAW264.7 macrophage cells. Among the evaluated hybrids, derivative 12 displayed the most prominent anti-inflammatory impact by notably decreasing the expression of NO and TNF-α (IC_50_ = 62.93 and 67.50 µM, respectively). Moreover, derivative 12 suppressed COX-2 (IC_50_ = 73.18 µM) relative to celecoxib IC_50_ = 41.21 µM). Notably, the investigated derivatives did not disclose substantial toxic impact at a concentration of 50 µM when exposed to RAW264.7 cells. Furthermore, derivative 12 demonstrated the highest docking score (–183.72 kcal mol^−1^) within the COX-2 active region. Derivative 12 displayed two H-bonds with Cys47 and Asn34. In addition, hydrophobic interactions with Cys47, Arg44, Tyr130, Pro153, Lys137 and Cys36 were detected. The condensation of 7-methoxy-1-tetralone 13 with 4-methylphenyl isothiocyanate 14 produced 1,2,3,4-tetrahydronaphthalene-2-carbothioamide 15. Moreover, the regioselective synthesis of thiazole-naphthalenone hybrid 12 was accomplished by condensing intermediate 15 with 3-chloropentane-2,4-dione 16 (Scheme 3).

### 2.3 Thiazole derivatives bearing hydrazine/hydrazone/amide/amine and imine moieties as anti-inflammatory agents

**Fig. 5:**
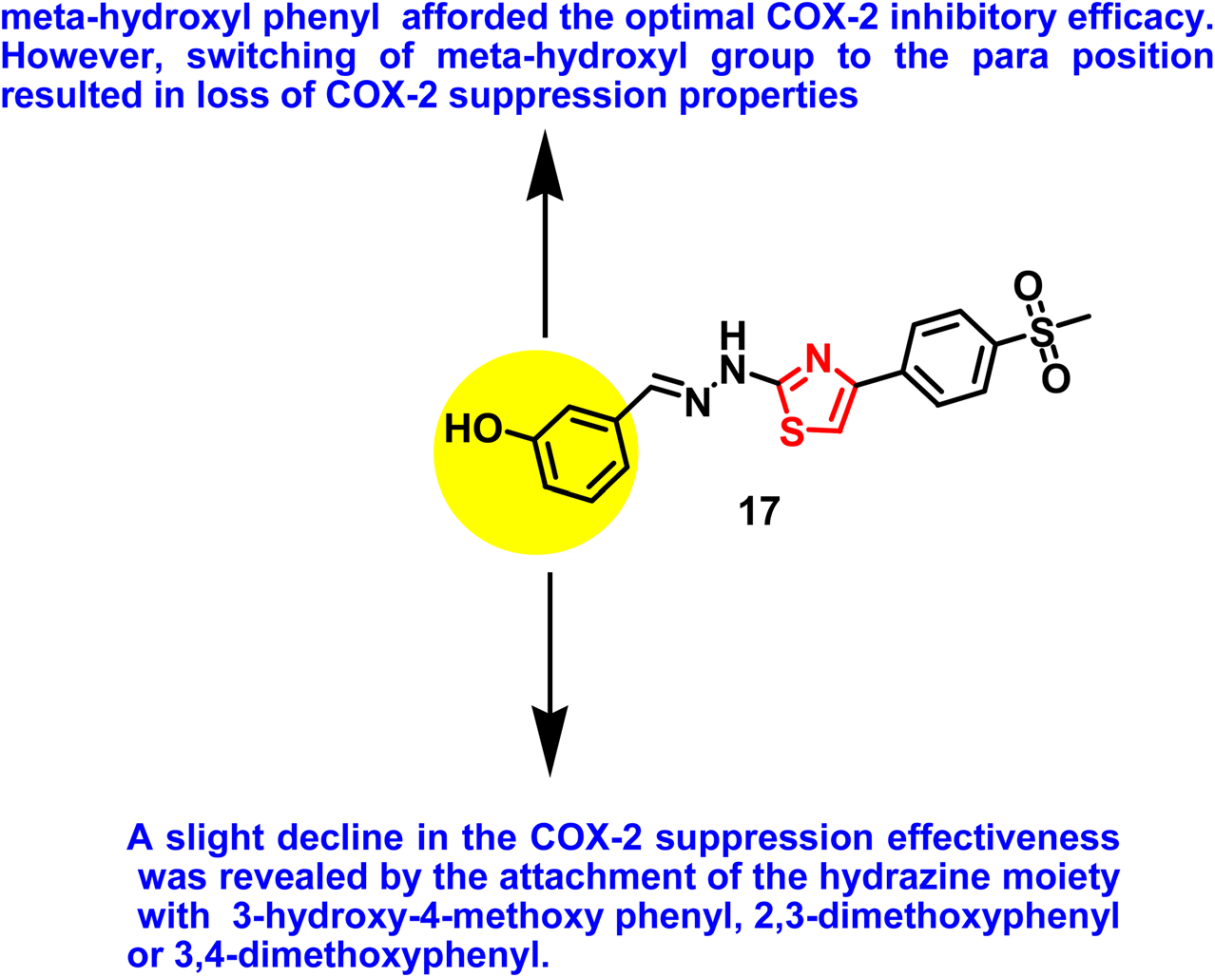
Structure of the thiazolylhydrazine derivative 17 as the COX-2 inhibitor.

A new thiazolylhydrazine derivative linked to a (4-methyl sulfonyl)phenyl moiety 17 stood out as a prominent COX-2 inhibitor (IC_50_ = 0.140 µM, SI > 714.286), relative to that of celecoxib (IC_50_ = 0.132 µM). It was noticed that the connection of the hydrazine moiety with *meta*-hydroxyl phenyl afforded the optimal COX-2 inhibitory efficacy. However, switching of the *meta*-hydroxyl group to the *para* position resulted in the loss of COX-2 suppression properties. Moreover, a slight decline in the COX-2 suppression effectiveness was revealed by the attachment of the hydrazine moiety with 3-hydroxy-4-methoxy phenyl, 2,3-dimethoxyphenyl or 3,4-dimethoxyphenyl. Docking study of derivative 17 inside the binding region of COX-2 revealed the formation of pi–pi interaction between phenyl bearing 4-methyl sulfonyl and Tyr341. Moreover, three H-bonds were disclosed between the OH group, the N atom of the thiazole ring and imine nitrogen with Met508, Val335 and Ser516, respectively. In addition, the O atoms of the methyl sulfonyl moiety interacted with Phe504 and Arg499 through two hydrogen bonds. Furthermore, it was demonstrated that all the physicochemical features fall within the acceptable range for drug candidacy. Among these parameters, PSA is a substantial measure of drug-likeness, and derivative 17 showed a PSA value of 95.154, suggesting good human intestinal absorption. Additionally, it was observed that derivative 17 demonstrated promising cell permeability in Caco-2 and MDCK cell lines with values of 250.659 and 219.101, respectively, which are very fundamental for drug absorption and bioavailability^64^ (Fig. 5).

**Scheme 4:**
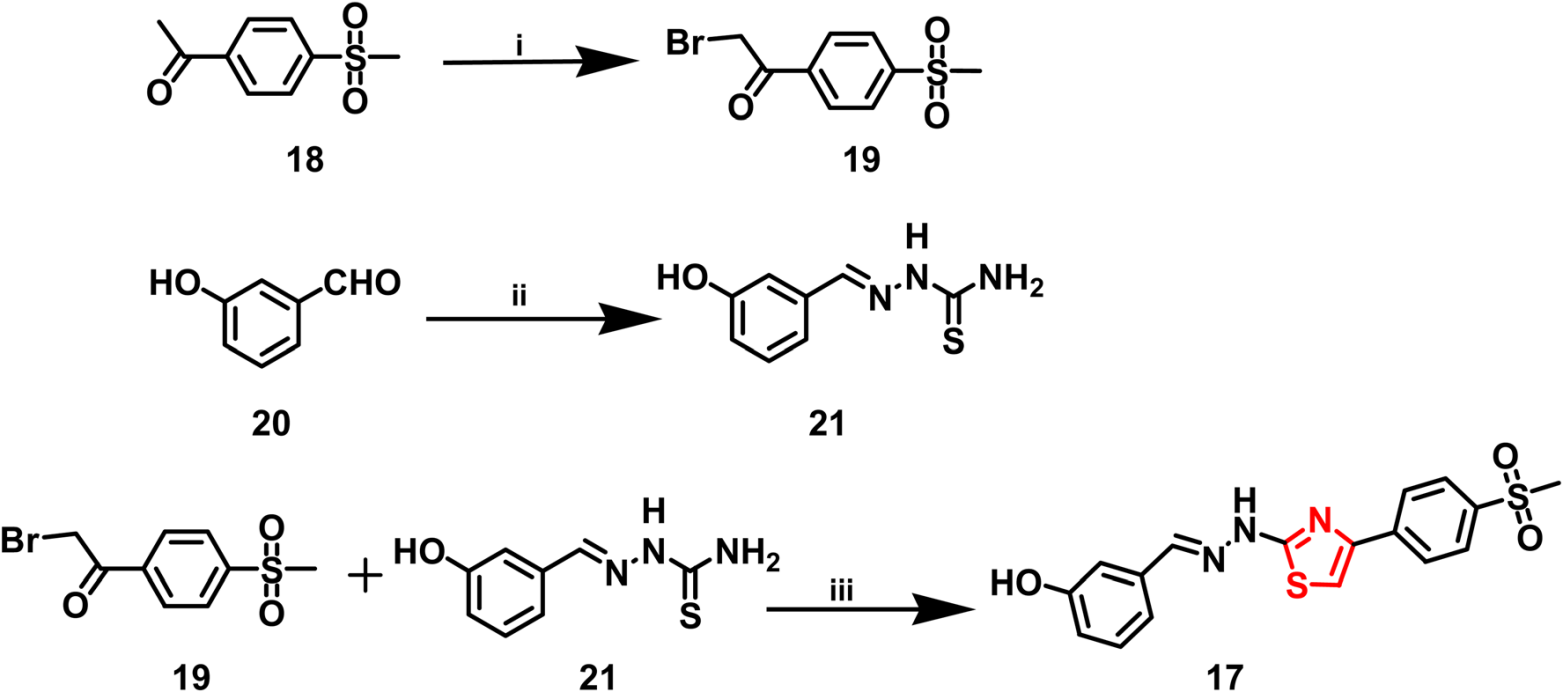
Synthesis of the thiazolylhydrazine derivative 17. Reagents and conditions: (i) Br_2_, HBr, AcOH, stirring, 0 °C, 20 h; (ii) NH_2_NHCSNH_2_, EtOH, reflux, 6 h; (iii) EtOH, reflux.

Derivative 17 was achieved *via* a ring closure reaction between equimolar amounts of 2-bromo-1-(4-(methylsulfonyl)phenyl)ethan-1-one 19 and 2-substituehydrazine-1-carbothioamide 21 (Scheme 4).^64^

**Fig. 6:**
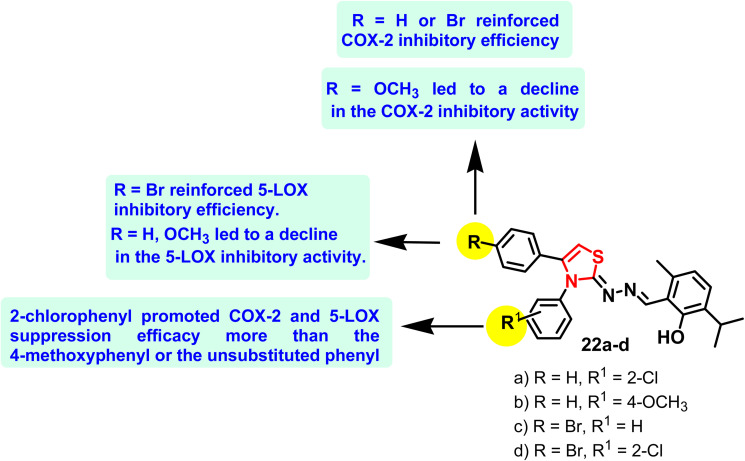
Structures of the thiazole-thymol hybrids bearing hydrazone moiety 22a–d as anti-inflammatory agents targeting dual 5-LOX and COX-2 enzymes.

A new set of thiazole-thymol hybrids bearing a hydrazone moiety were synthesized and assessed for their inhibitory impacts on COX-2/5-LOX enzymes. Among the examined hybrids, derivatives 22a–d manifested prominent COX-2 suppression efficacy with IC_50_ ranging from 0.037 to 0.046 µM, relative to celecoxib (IC_50_ = 0.045 µM) and remarkable COX-2 selectivity varying from 301 to 379. In addition, derivatives 22c, d demonstrated the highest 5-LOX inhibitory effectiveness (IC_50_ = 1.75 and 1.53 µM, respectively) surpassing that of quercetin (IC_50_ = 3.34 µM). It was observed that the presence of a *p*-bromo group on the phenyl ring at the 4th position of the thiazole ring enhanced COX-2 inhibitory activity, ranking second only to the unsubstituted derivatives. Conversely, the substitution with a methoxy group led to a decline in the activity. Otherwise, the appearance of *p*-bromo halogen on the phenyl ring at the 4th position of the thiazole ring boosted 5-LOX inhibitory properties over the unsubstituted and *p*-methoxy groups. Moreover, the attachment of 2-chlorophenyl at the 3rd position of the thiazole scaffold promoted dual COX-2 and 5-LOX suppression efficacy more than the 4-methoxyphenyl or the unsubstituted phenyl. The *in vivo* investigation indicated that derivatives 22a–d demonstrated a higher percentage of edema inhibition than celecoxib. Derivatives 22a, d demonstrated a better gastrointestinal safety profile (no ulcers) in a group of starving rats, according to gross inspection. Conversely, compounds 22b, c displayed varying levels of hyperemia. The potential actions of derivatives 22a, d against COX-2 were validated by the docking study, since the forms of interaction were similar to those of the reference celecoxib. Both derivatives displayed hydrophobic and polar interactions with Leu338 and Ser339, respectively. Furthermore, derivatives 22a, d contributed to H-bonds between imino N and Arg106, whereas derivative 22a displayed an additional H-bond between the Cl atom and Val335. Otherwise, the docking of derivatives 22a, d inside 5-LOX active sites displayed hydrophobic interactions with Ile673, Leu368, Leu607, Leu414 and Ile406. Additionally, they demonstrated polar interactions with His372, Gln363 and His360 (ref. 65) (Fig. 6).

**Scheme 5:**
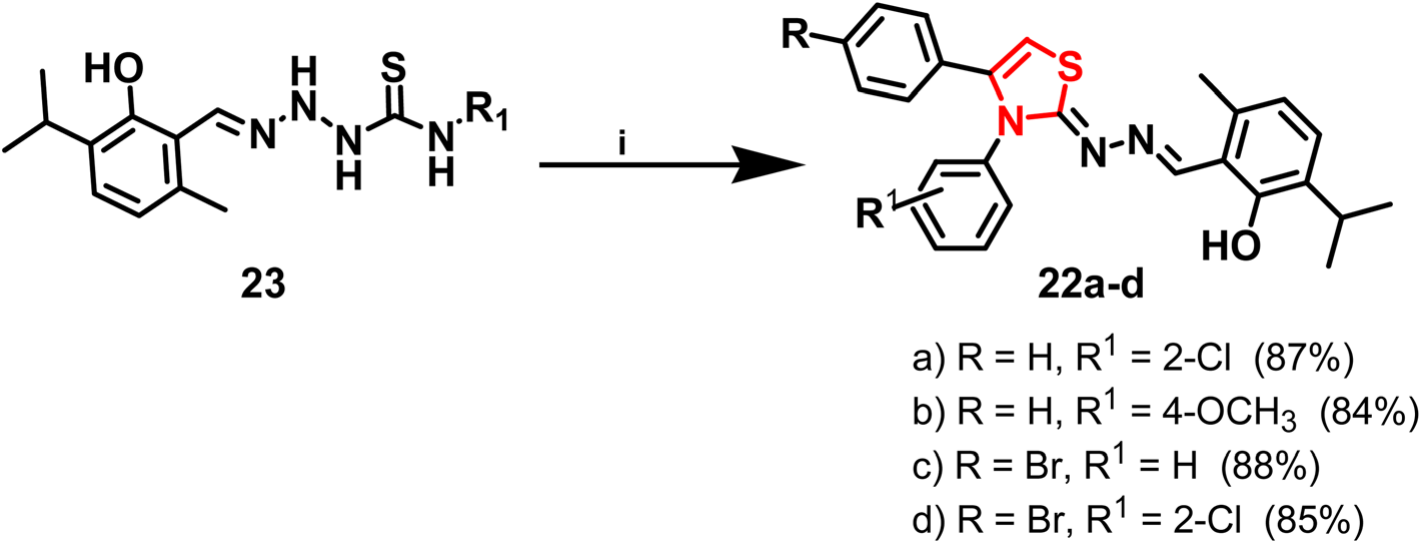
Synthesis of the thiazole-thymol hybrids bearing hydrazone moiety 22a–d. Reagents and conditions: (i) gl.AcOH, fused sodium acetate, (un)substituted phenacylbromide, reflux, 5–8 h.

Target thiazole-thymol hybrids bearing hydrazone moiety 22a–d were obtained as illustrated in Scheme 5.^65^

**Scheme 6:**
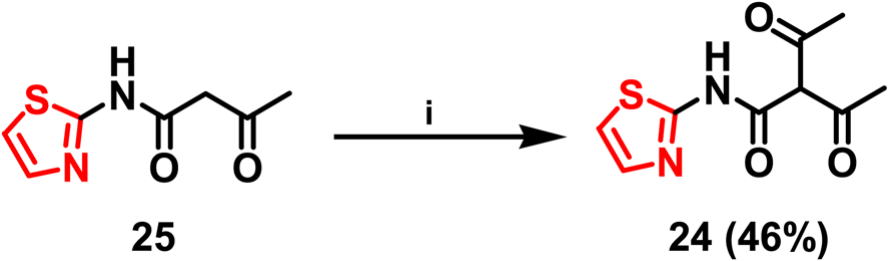
Synthesis of the thiazole-butanamide derivative 24. Reagents and conditions: (i) CH_3_COCl, benzene, reflux, 12 h.

In 2020, thiazole-butanamide derivative 24 emerged as a notable and selective COX-2 inhibitor (IC_50_ = 0.09 µM, SI = 15.56), which was nearly 9.2-fold more effective than celecoxib (IC_50_ = 0.83 µM, SI = 8.68). Refluxing of 3-oxo-*N*-(thiazol-2-yl)butanamide 25 with acetyl chloride in benzene furnished derivative 24 (Scheme 6).^66^

**Fig. 7:**
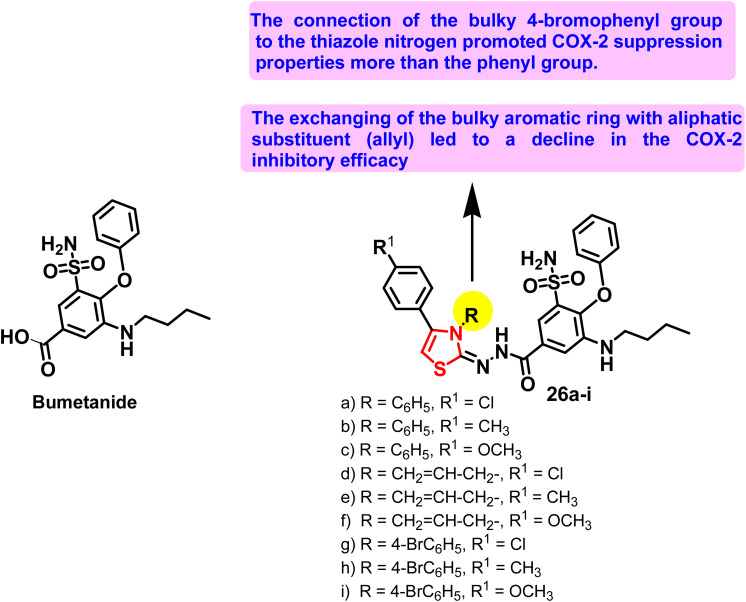
Structures of the thiazole-bumetanide hybrids 26a–i as COX-2 inhibitors.

Ibrahima *et al.*^67^ utilized bumetanide as a precursor to create new thiazole-bumetanide hybrids 26a–i as notable COX-2 inhibitors (IC_50_ = 1.73, 1.85, 1.90, 3.43, 3.82, 3.46, 1.52, 1.56 and 1.54 µM, respectively) superior to that of celecoxib (IC_50_ = 4.04 µM). It was detected that the connection of the bulky 4-bromophenyl group to the thiazole nitrogen promoted COX-2 suppression properties more than the phenyl group. Otherwise, the exchanging of the bulky aromatic ring with aliphatic substituent (allyl) led to a decline in the COX-2 inhibitory efficacy (Fig. 7).

**Scheme 7:**
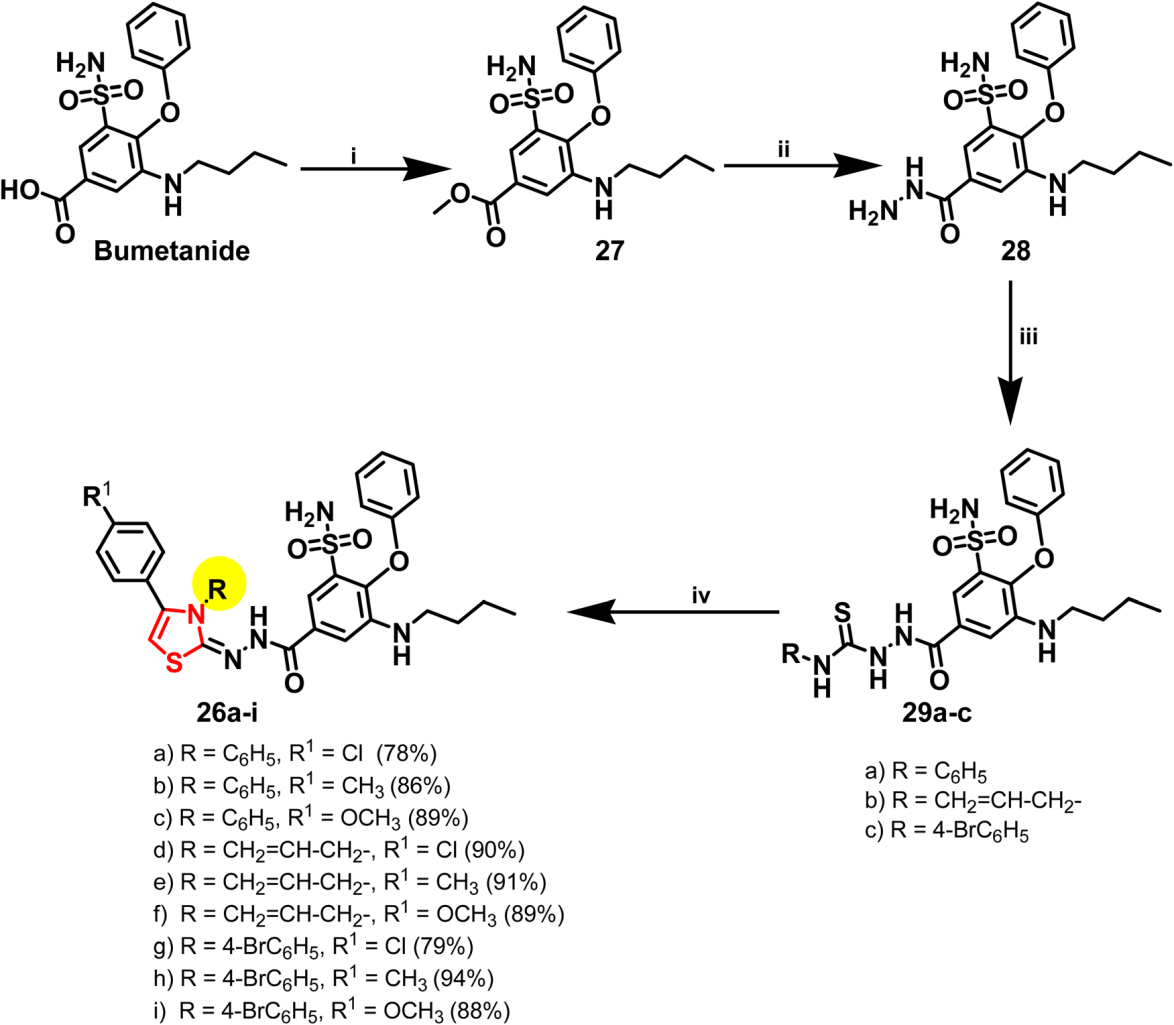
Synthesis of the thiazole-bumetanide hybrids 26a–i. Reagents and conditions: (i) MeOH, H_2_SO_4_, 60 °C, 1 h; (ii) NH_2_NH_2_, EtOH, 80 °C, 6 h; (iii) RNCS, EtOH, 80 °C, 3 h; (iv) substituted phenacyl bromide, dioxane, AcONa, 6 h.

The esterification of bumetanide yielded the methyl ester derivative 27,^68^ which underwent hydrazinolysis to furnish the acid hydrazide derivative 28. Thiosemicarbazide derivatives 29a–c were accomplished *via* the treatment of the acid hydrazide 3 with various isothiocyanate derivatives. The prepared thiosemicarbazide derivatives 29a–c were cyclized to afford the new thiazole-bumetanide hybrids 26a–i*via* condensation with different phenacyl bromides (Scheme 7).^67^

**Fig. 8:**
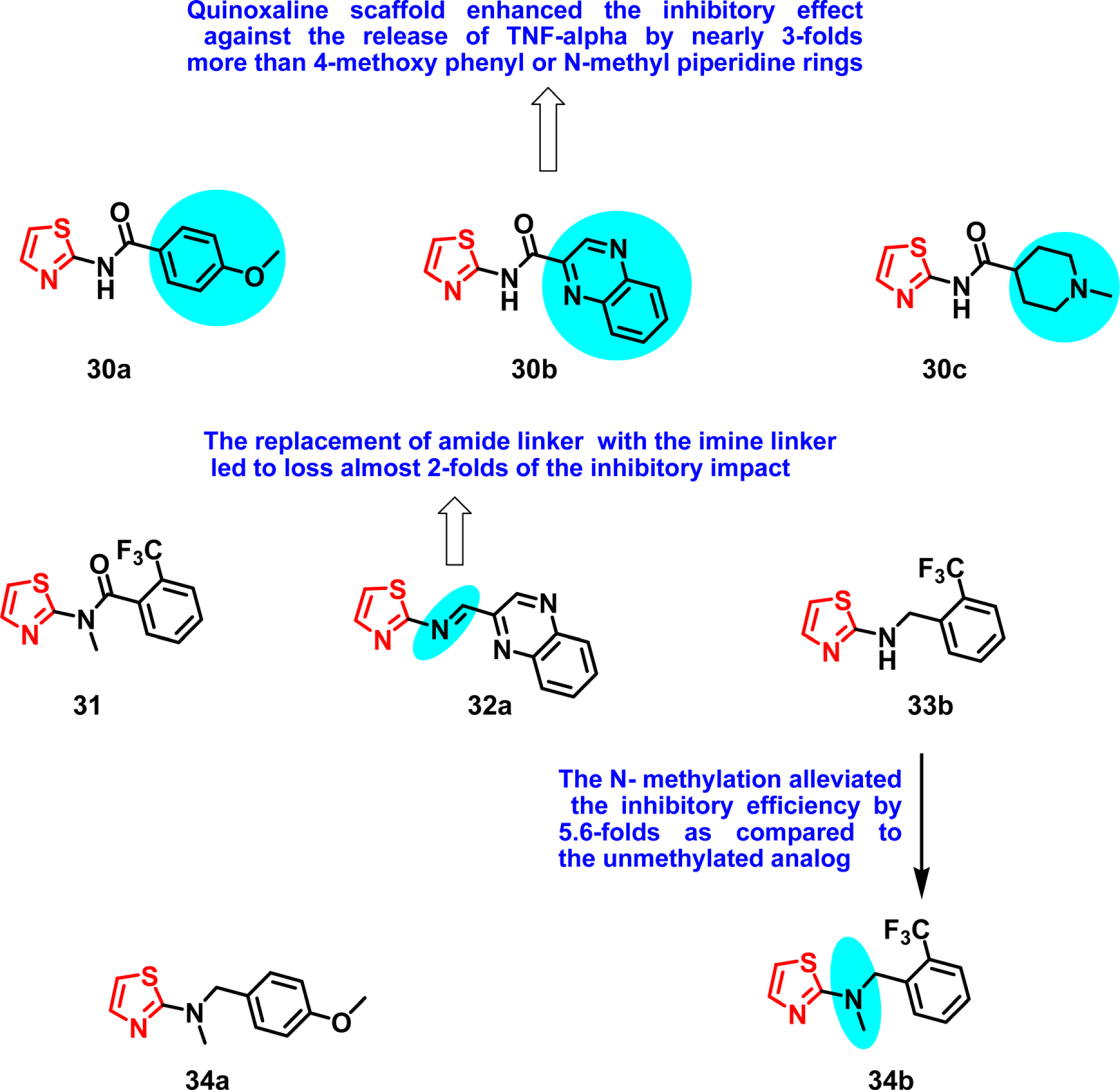
Structures of the thiazole derivatives bearing amide (30a–c, 31), imine (32a) and amine (33b, 34a, b) moieties as TNF-α and IL-8 inhibitors.

Modric *et al.*^69^ discovered some thiazole derivatives bearing amide (30a–c, 31), imine (32a) and amine (33b, 34a, b) functional groups as efficient anti-inflammatory candidates, and they suppressed the production of LPS-induced TNF-α with IC_50_ ranging from 8 to 56 µM. Meanwhile, only derivative 34b suppressed the release of IL-8 (IC_50_ = 47 µM). Notably, the screened derivatives presented a prominent safety profile against SK-OV-3 cells. The thiazole amide derivative 30b bearing a quinoxaline scaffold presented the most prominent inhibitory effect against the release of TNF-α (IC_50_ = 8 µM), whereas the TNF-α suppression effectiveness was declined by nearly 3-folds *via* exchanging of quinoxaline with 4-methoxy phenyl 30a or *N*-methyl piperidine rings 30c. Furthermore, the replacement of the amide linker for derivative 30b with the imine linker 32a led to loss almost 2-folds of the inhibitory impact. In addition, the *N*-methylation derivative 34b alleviated the inhibitory efficiency by 5.6-fold as compared to the unmethylated analog 33b (Fig. 8).

**Scheme 8:**
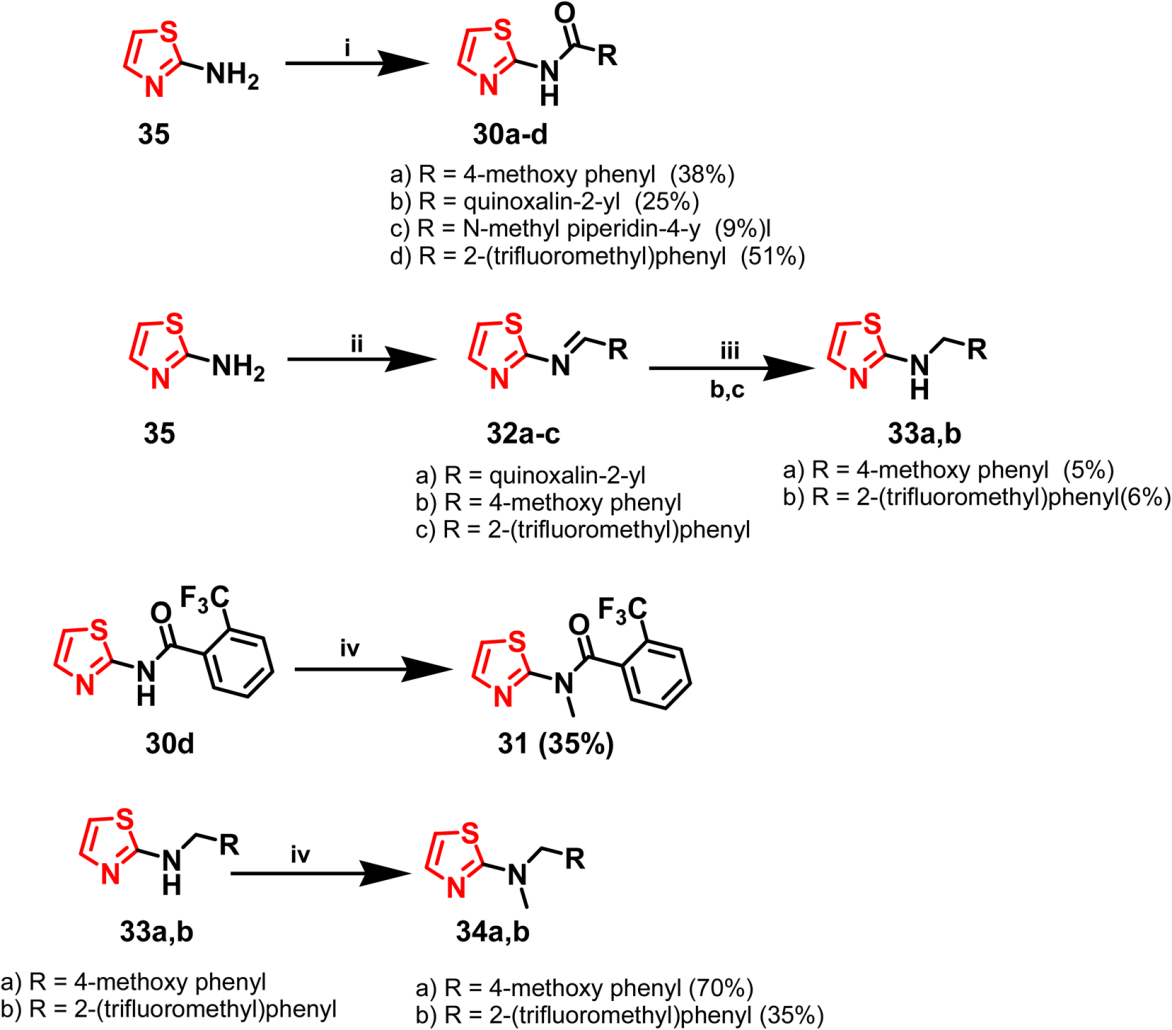
Synthesis of the thiazole derivatives bearing amide (30a–d, 31), imine (32a–c) and amine (33a, b, 34a, b) functional groups. Reagents and conditions: (i) RCOOH, HATU, DIPEA, DMF, 50 °C, 24 h; (ii) RCHO, EtOH, reflux, 24 h; (iii) NaBH_4_, EtOH, 70 °C, 3 h, RT; (iv) CH_3_I, NaH, THF, 24 h, RT.

The desired amide derivatives 30a–d were accomplished *via* the reaction of 2-amino thiazole 35 and different carboxylic acids. Moreover, 2-amino thiazole 35 reacted with different aldehydes to yield the imine derivatives 32a–c. The corresponding imines 32b, c were reduced using NaBH_4_ to furnish the amine derivatives 33a, b. Moreover, the amide derivative 30d as well as the amine derivatives 33a, b were methylated utilizing iodomethane and NaH to afford the methylated analogs 31 and 34a, b, respectively (Scheme 8).^69^

**Fig. 9:**
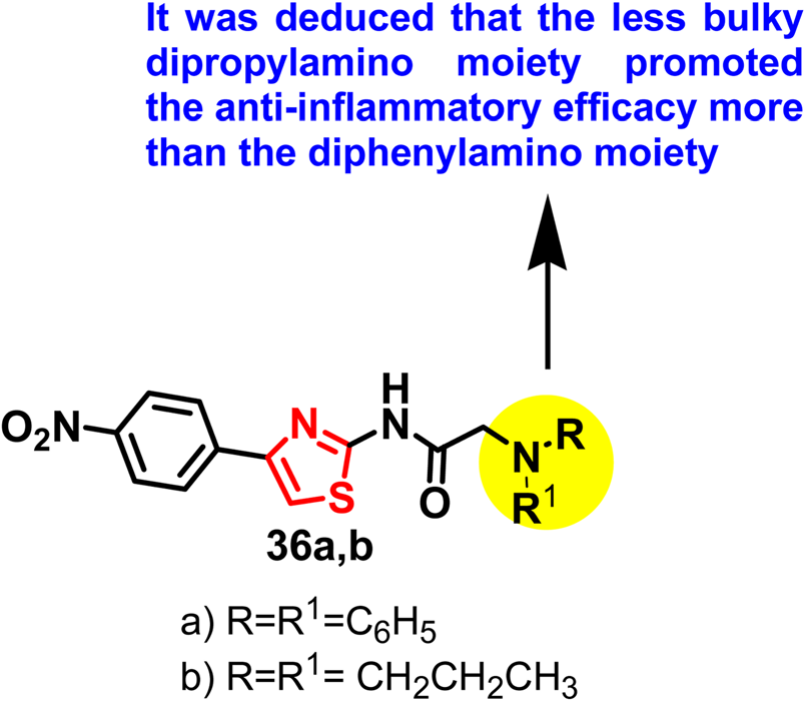
Structures of phenylthiazole derivatives 36a, b carrying an acetamide moiety as anti-inflammatory agents.

Porwal *et al.*^70^ described the preparation and determination of the *in vivo* anti-inflammatory effectiveness of new phenylthiazole derivatives carrying an acetamide moiety. According to the formalin-stimulated paw edema, derivatives 36a, b demonstrated a modest anti-inflammatory impact relative to indomethacin (edema inhibition = 36%, 41% and 60%, respectively). It was deduced that the less bulky dipropylamino moiety promoted the anti-inflammatory efficacy more than the diphenylamino moiety (Fig. 9).

**Scheme 9:**
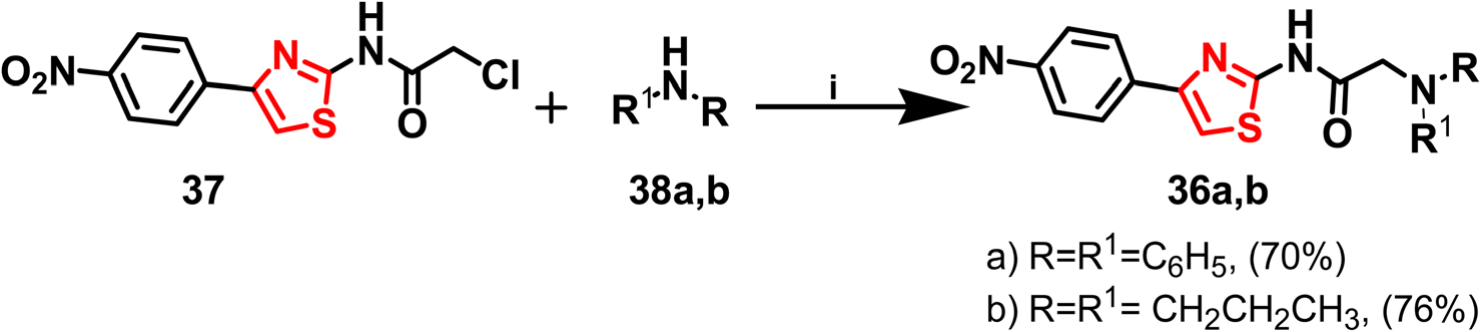
Synthesis of the new phenylthiazole derivatives 36a, b carrying an acetamide moiety. Reagents and conditions: (i) EtOH, reflux, 2 h.

The target products 36a, b were attained as described in Scheme 9.^70^

**Scheme 10:**
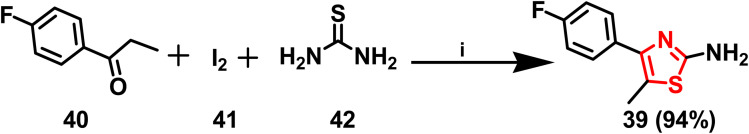
Synthesis of the thiazole-2-amine derivative 39. Reagents and conditions: (i) solvent free, MW, 120–140 °C, 10 min.

New thiazole-2-amine derivative 39 was prepared utilizing a green microwave irradiation method and assessed for its anti-inflammatory efficacy using the protein denaturation method. It manifested the highest inhibition at 1000 µg mL^−1^ (82.63%) as compared to ibuprofen (80.44%). All the requirements for drug-likeness features were met for investigated derivatives, and there were no violations. Derivative 39 was synthesized utilizing a one-pot reaction of 4-fluoropropiophenone 40, iodine 41 and thiourea 42 under solvent-free conditions, using microwave irradiation (Scheme 10).^71^

**Fig. 10:**
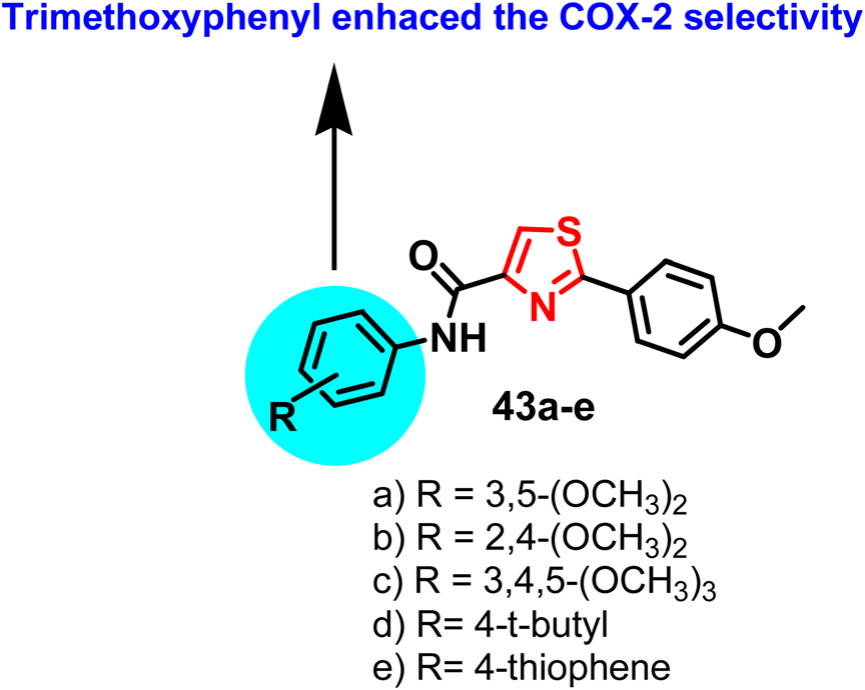
Structures of thiazole carboxamide derivatives 43a–e as COX-2 inhibitors.

New thiazole carboxamide derivatives 43a–e emerged as COX-2 inhibitors. Derivatives 43a–e displayed COX-2 inhibitory efficacy in the range of 53.9–81.5%, at 5 µM concentration, whereas the inhibitory effect against COX-1 was 14.7–74.8%. Moreover, they demonstrated COX-2 suppression impact in the range of 90.9–99%, and COX-1 inhibitory efficiency in the range of 39.1–99.8%, at 40 µM concentration. Derivative 43c was the most selective candidate at both 5 and 40 µM concentrations (SI = 3.67 and 2.37, respectively). In fact, this compound's selectivity results from the bulky functional group trimethoxyphenyl. The docking score of derivative 43c with the COX-2 enzyme was −5.492 kcal mol^−1^. It established two H-bonds with His90 and Arg120, in addition to hydrophobic interactions with Tyr355, Val523 and Leu93^72^ (Fig. 10).

**Scheme 11:**
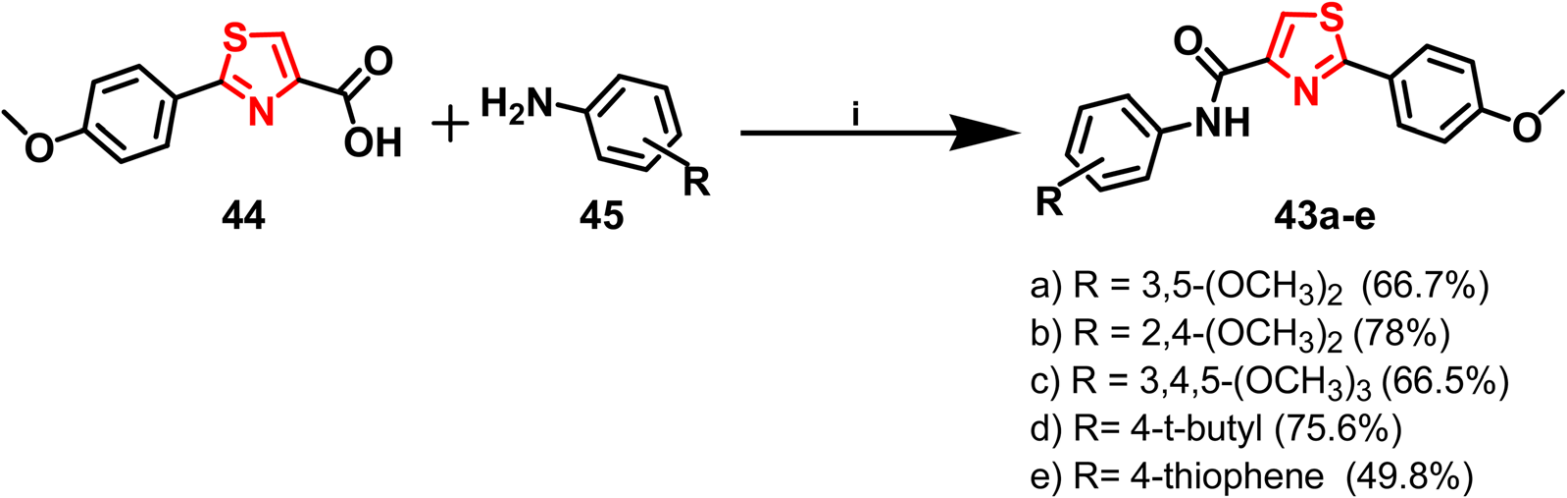
Synthesis of the thiazole carboxamide derivatives 43a–e. Reagents and conditions: (i) DCM, DMAP, EDC, inert argon gas, stirring, 48 h. Reagents and conditions: (i) solvent free, MW, 120–140 °C, 10 min. Reagents and conditions: (i) DCM, DMAP, EDC, inert argon gas, stirring, 48 h.

The preparation of the thiazole carboxamide derivatives 43a–e is described as outlined in Scheme 11.^72^

**Fig. 11:**
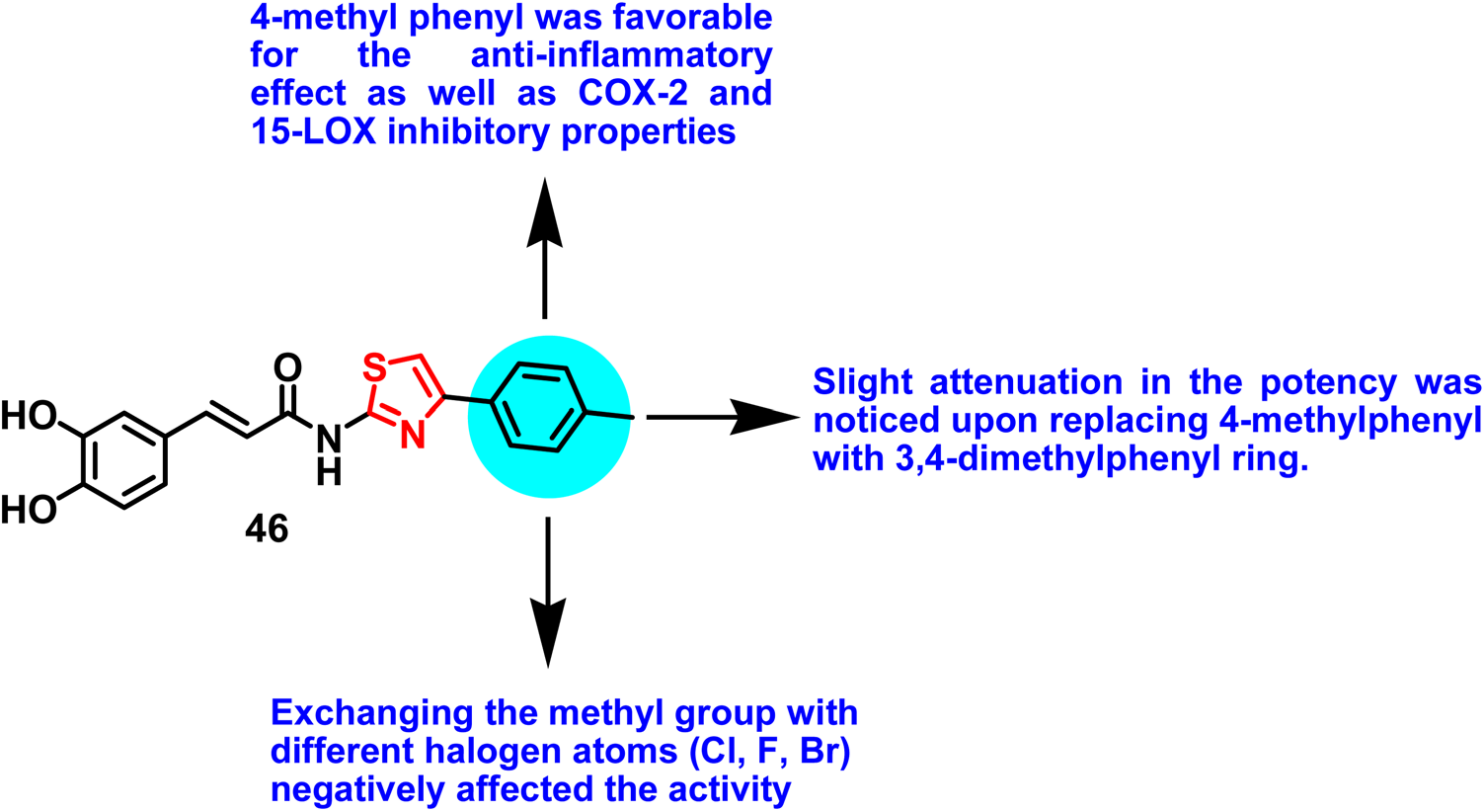
Structure of 4-phenyl acrylamide-thiazole hybrid 46 as an anti-inflammatory agent targeting dual 5-LOX and COX-2 enzymes.

In 2023, new 4-phenyl acrylamide-thiazole hybrid 46 was discovered as a notable anti-inflammatory candidate (inhibition of albumin denaturation = 77.85%). In addition, it was identified as a dual outstanding COX-2 and 15-LOX inhibitor (IC_50_ = 8.68 and 11.14 µM, respectively). It was noted that the conjugation of the thiazole core with 4-methyl phenyl was favorable for the anti-inflammatory effect as well as COX-2 and 15-LOX inhibitory properties, while a slight attenuation in the potency was noticed upon replacing 4-methylphenyl with a 3,4-dimethylphenyl ring. Otherwise, exchanging the methyl group with different halogen atoms (Cl, F, and Br) negatively affected the activity. Furthermore, the anti-ulcer activity was assessed utilizing the *in vitro* H+/K + ATPase inhibition method, and the outcomes indicated that derivative 46 displayed eminent inhibitory effects against H^+^/K^+^ ATPase (IC_50_ = 35.35 µg mL^−1^) relative to the standard omeprazole (IC_50_ = 37.91 µg mL^−1^). Derivative 46 demonstrated a remarkable docking score against COX-2 (−10.55 kcal mol^−1^). Moreover, Tyr385 and Tyr355 residues participated in pi–pi stacking with derivative 46, whereas Leu384, Trp387, Ser530 and Leu352 are engaged in H-bonds with derivative 46 (ref. 73) (Fig. 11).

**Scheme 12:**
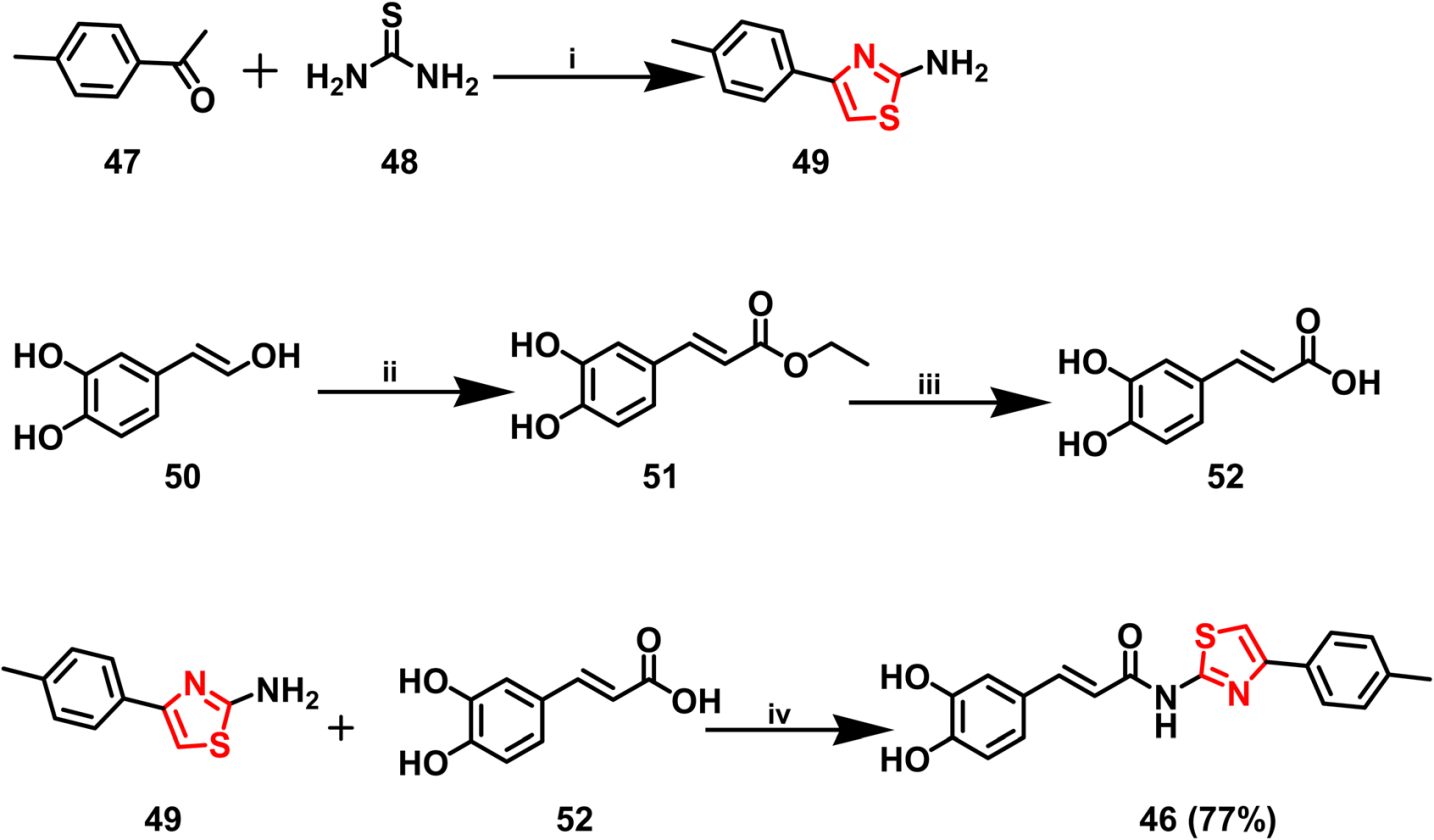
Synthesis of the 4-phenyl acrylamide-thiazole hybrid 46. Reagents and conditions: (i) I_2_, EtOH, reflux, 12 h; (ii) ethyl chloroacetate, (CH_3_)_2_CO, anhydrous potassium carbonate, reflux, 10 h; (iii) sodium hydroxide, EtOH, reflux, 9 h; (iv) dry DCM, lutidine, TBTU, stirring.

The refluxing of 4-methyl acetophenone 47 and thiourea 48 afforded the intermediate 49, while refluxing 4-(2-hydroxy vinyl)benzene-1,2-diol 50 and ethyl chloroacetate yielded ethyl-3-(3,4-dihydroxyphenyl) acrylate 51, which underwent a reaction with sodium hydroxide to furnish the intermediate 52. Finally, the target product 46 was obtained *via* stirring the intermediates 49 and 52 (Scheme 12).^73^

**Fig. 12:**
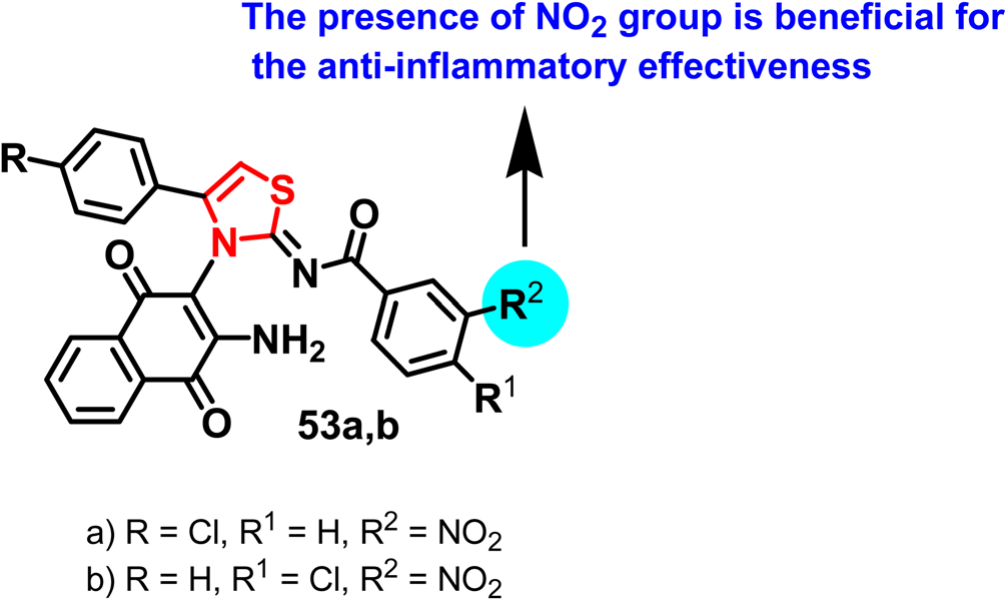
Structures of naphthoquinone-thiazole hybrids bearing amide moieties 53a, b as IL-6 and TNF-α inhibitors.

The release of IL6 and TNF-α in the LPS-stimulated cells was attenuated by two naphthoquinone-thiazole hybrids bearing amide moieties 53a, b, which demonstrated notable anti-inflammatory immunomodulatory activities. The presence of a NO_2_ group at the *R*_2_ position is beneficial for the anti-inflammatory effectiveness. Derivatives 53a, b were anticipated to possess an appropriate brain/blood partition coefficient with values of −1.58 and −1.59, respectively, which is crucial for medications that target the central nervous system. Additionally, the QPlogPo/w values are 3.78 and 3.77 that fall within the range, which is allowed for CNS penetration. Moreover, their QPPCaco values are 104.67 and 107.85, respectively, suggesting their good cell permeability. In addition, further *in silico* investigations for derivatives 53a, b were performed with key signaling pathways that are crucial for inflammatory responses. It was predicted that PI3K would be the possible target, and both derivatives 53a, b presented notable docking scores with values of −6.82 and −7.01 kcal mol^−1^, respectively, as compared to that of the native Ligand (–9.02 kcal mol^−1^)^74^ (Fig. 12).

**Scheme 13:**
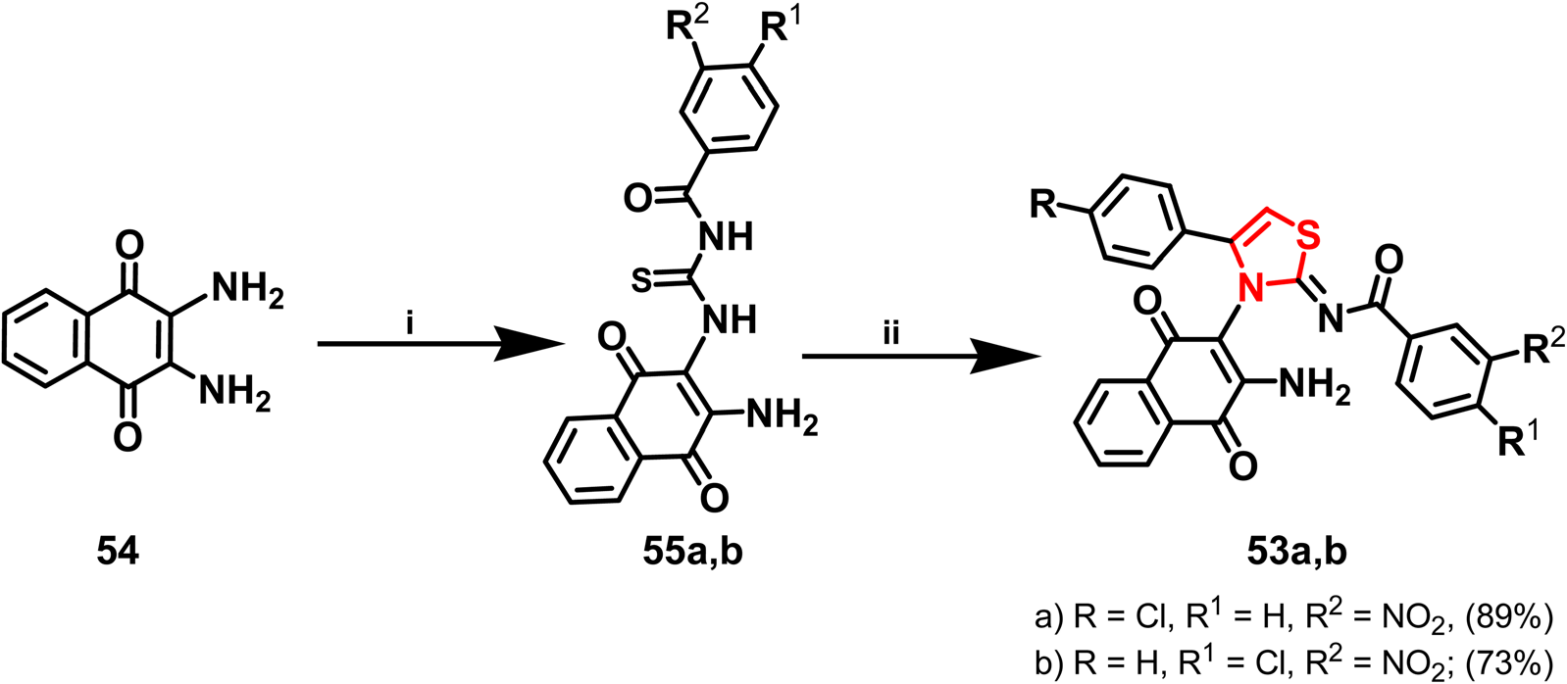
Synthesis of the naphthoquinone-thiazole hybrids bearing amide moieties 53a, b. Reagents and conditions: (i) substituted acyl isothiocyanate, (CH_3_)_2_CO, reflux, 18 h; (ii) (un) substituted phenacyl bromide, (CH_3_)_2_CO, reflux, 36 h.

Following the reported method,^75^ the intermediates 55a, b were prepared *via* refluxing of diaminonaphthalene-1,4-dione 54 and the appropriate acyl isothiocyanate. The prepared intermediates 55a, b underwent a reaction with (un)substituted phenacyl bromide to afford naphthoquinone-thiazole hybrids 53a, b (Scheme 13).^74^

**Fig. 13:**
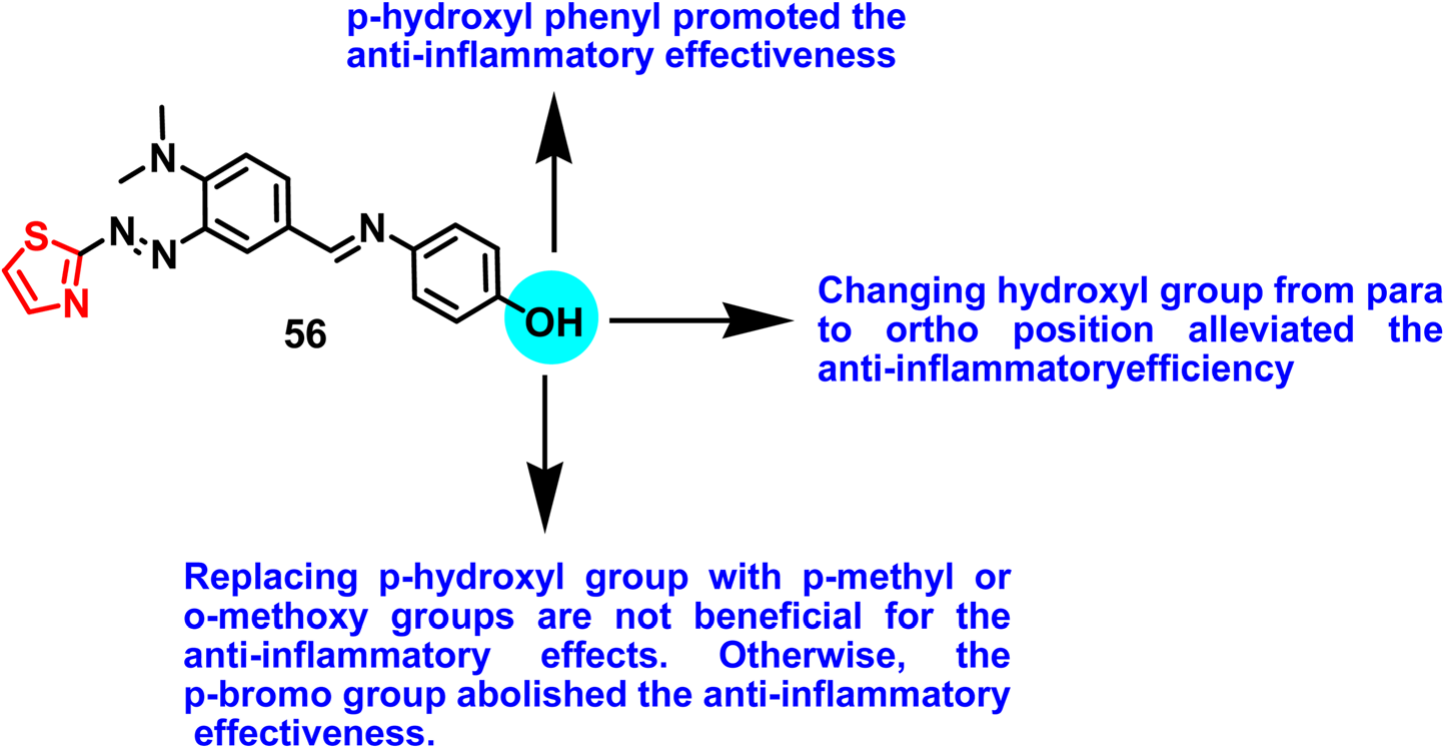
Structure of thiazole derivatives bearing azo-azomethine moiety 56 as an anti-inflammatory agent.

A new thiazole derivative bearing azo-azomethine moiety 56 suppressed hemolysis of red blood cells (percentage of prevention of lysis ranging from 71.37% to 84.72%) at different concentrations, indicating its prominent *in vitro* anti-inflammatory efficiency. It was noticed that the conjugation of the azomethine moiety with *p-*hydroxyl phenyl promoted the anti-inflammatory effectiveness. Otherwise, changing a hydroxyl group from the para to the *ortho* position alleviated the anti-inflammatory efficiency. Moreover, replacing *p-*hydroxyl groups with *p*-methyl or *o*-methoxy groups is not beneficial for the anti-inflammatory effects. Otherwise, the *p*-bromo group abolished the anti-inflammatory effectiveness. Furthermore, derivative 56 disclosed remarkable burn healing properties relative to Hamazine cream as a standard medication. Additionally, the administration of derivative 56 attenuated the levels of AST and ALT relative to the negative control^76^ (Fig. 13).

**Scheme 14:**
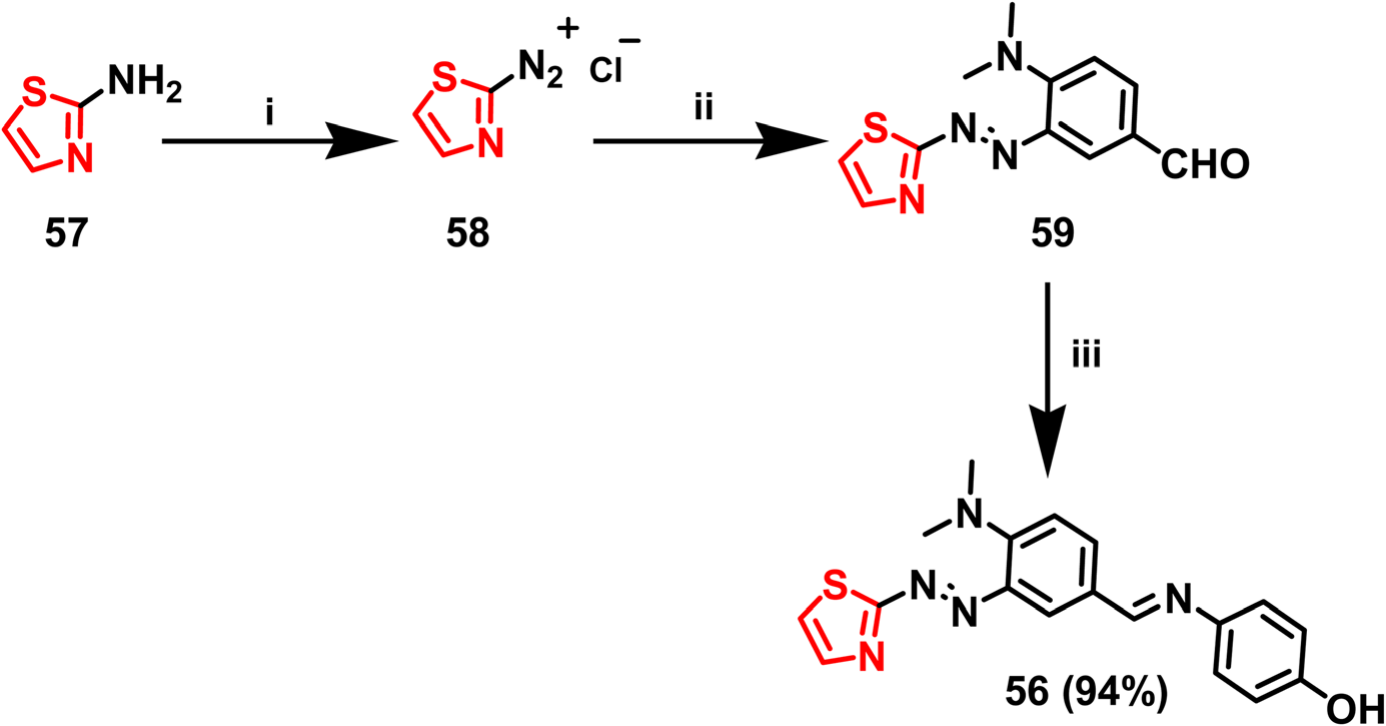
Synthesis of the thiazole derivative bearing azo-azomethine moiety 56. Reagents and conditions: (i) NaNO_2_, HCl, 0 °C, stirring 10 min; (ii) 4-(dimethylamino)benzaldehyde, acetonitrile, 0 °C, stirring, 2 h; (iii) *para* amino phenol, EtOH, AcOH, sonication, 60 °C, 20 min.

Thiazole-2-diazonium chloride 58 was prepared *via* the diazotization of 2-aminothiazole 57, which underwent a coupling reaction with 4-(dimethylamino)benzaldehyde to furnish the intermediate 59. The target thiazole 56 was accomplished through a reaction between the intermediate 59 with para amino phenol (Scheme 14).^76^

**Fig. 14:**
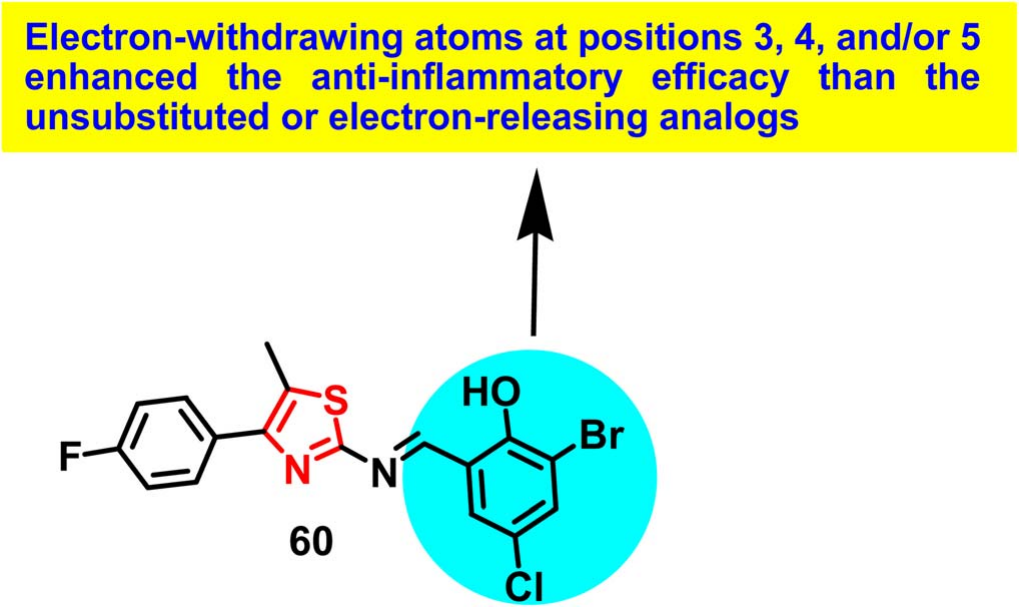
Structure of thiazole-bearing Schiff base derivative 60 as an anti-inflammatory agent.

New thiazole-bearing Schiff base derivative 60 emerged as an eminent anti-inflammatory candidate utilizing the protein denaturation assay. It disclosed promising inhibition values of 63.19% and 77.77%, comparable to that of ibuprofen (inhibition = 42.36% and 80.55%, respectively) at a concentration of 100 and 1000 µg mL^−1^, respectively. It was noted that the phenyl ring attached to the azomethine moiety bearing electron-withdrawing atoms at positions 3, 4, and/or 5 enhanced the anti-inflammatory efficacy more than the unsubstituted and electron-releasing analogs. Furthermore, ADME analysis displayed that derivative 60 presented promising drug-like characteristics, suggesting that derivative 60 is orally active. It exhibited exceptionally high lipophilicity, which may protect them from being destroyed by reactive oxygen species, with a log *P* value of 3.66. Moreover, it demonstrated efficient oral transportation and passage through the gastrointestinal system and blood–brain barrier^77^ (Fig. 14).

**Scheme 15:**
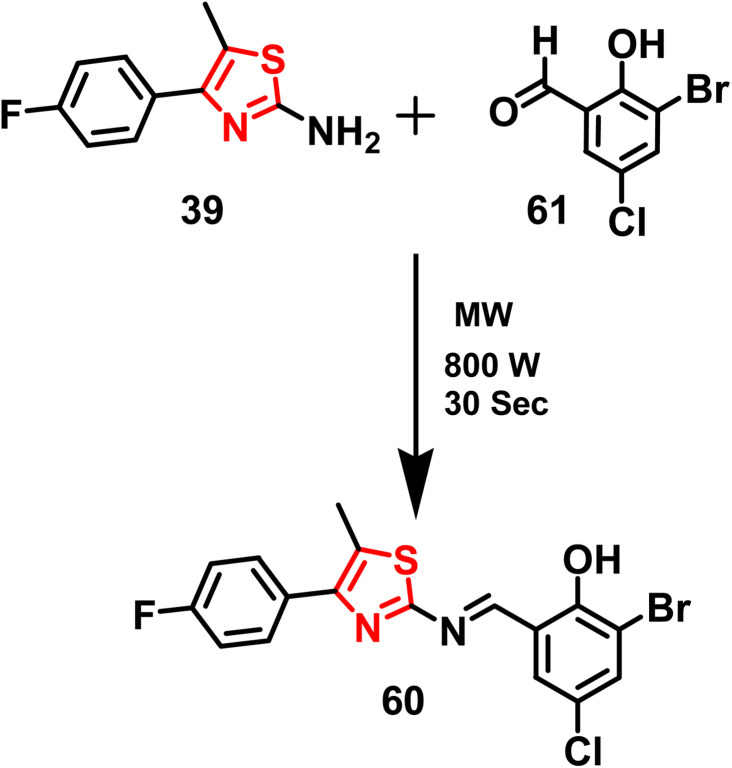
Synthesis of the thiazole-bearing Schiff base derivative 60.

Intermediate 39 was achieved *via* a microwave-assisted procedure.^71^ The target thiazole derivative 60 was obtained *via* the microwave-assisted reaction of equimolar of intermediate 39 and 3-bromo-5-chloro salicylaldehyde 61 in ethanolic solution-containing piperidine (Scheme 15).^77^

### 2.4 Thiazole–pyrazole hybrids as anti-inflammatory agents

**Fig. 15:**
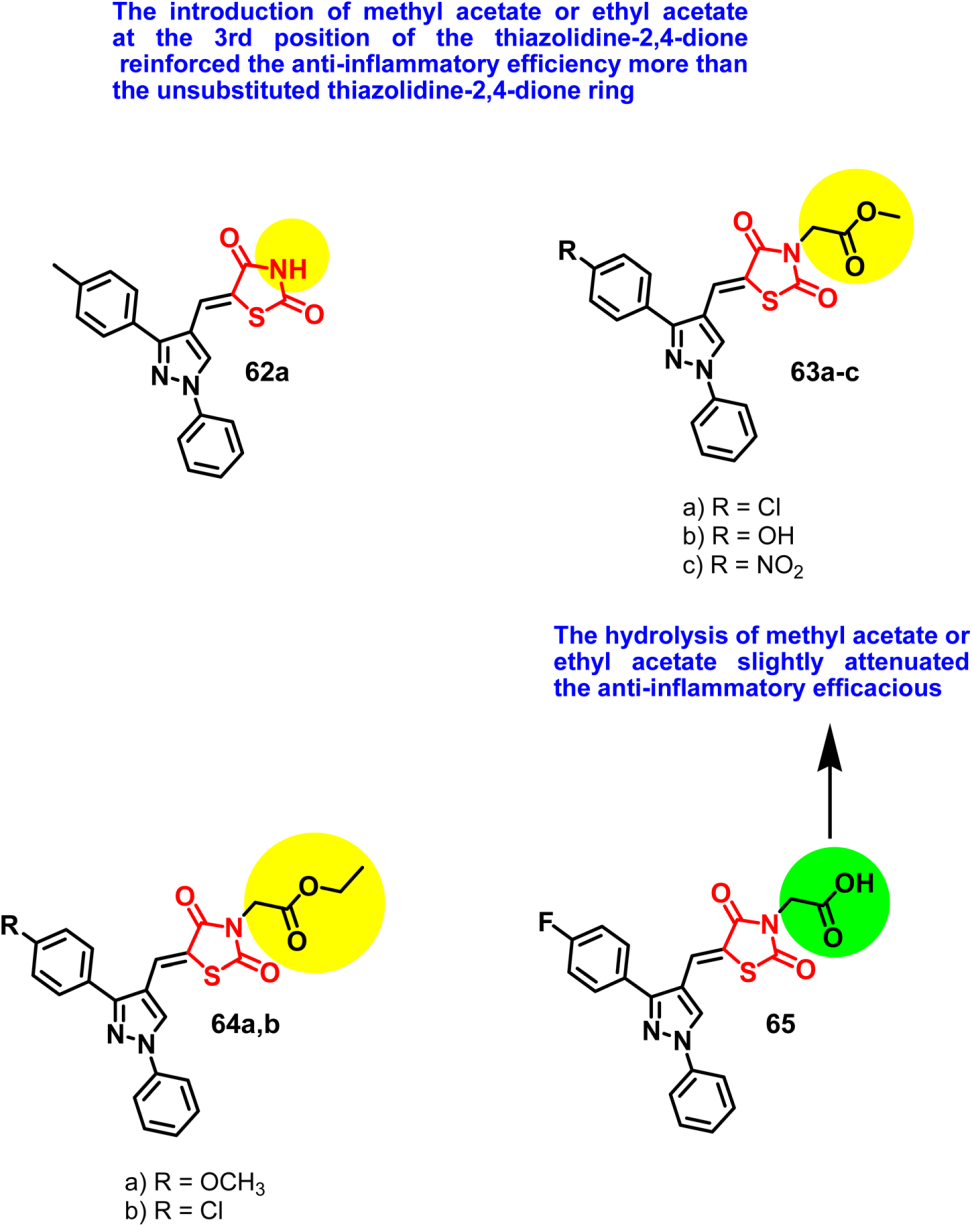
Structures of pyrazolyl-2,4-thiazolidinedione 62a, 63a–c, 64a, b and 65 as anti-inflammatory agents targeting COX-2 enzyme.

Aneja and Kaushik^78^ discussed the synthesis and the *in vivo* anti-inflammatory assessment of new sets of pyrazolyl-2,4-thiazolidinedione. Amongst the evaluated candidates, derivatives 62a, 63a–c, 64a, b and 65 presented an outstanding anti-inflammatory efficacy (edema inhibition = 82%, 86%, 87%, 85%, 91%, 89% and 86%, respectively) with regard to indomethacin (edema inhibition = 90%). It was deduced that the introduction of methyl acetate or ethyl acetate at the 3rd position of thiazolidine-2,4-dione reinforced the anti-inflammatory efficiency more than the unsubstituted thiazolidine-2,4-dione ring. However, the hydrolysis of methyl acetate or ethyl acetate slightly attenuated the anti-inflammatory efficacy. The docking scores of derivatives 62a, 63a–c, 64a, b and 65 within the COX-2 active site range from −123.521 to −162.365 kcal mol^−1^. The hydrophobic pocket comprising amino acids Leu359, Leu352, Val523, Ser353, Arg513, Arg120, Phe518, His90, Tyr385, Gln192 and Ala516 surrounded the pyrazolyl-2,4-thiazolidinedione derivatives. Derivative 64a demonstrated two H-bonds between *N*-2-Pyrazole and the methoxy group with Tyr355 and His90, respectively. In addition, the C=O group at the 2nd position of thiazolidine-2,4-dione was engaged in the H-bond with Ser530. Furthermore, Ser353 and Arg120 contributed to two H-bonds with the C=O group of thiazolidine-2,4-dione and C=O group of the ethyl acetate moiety (Fig. 15).

**Scheme 16:**
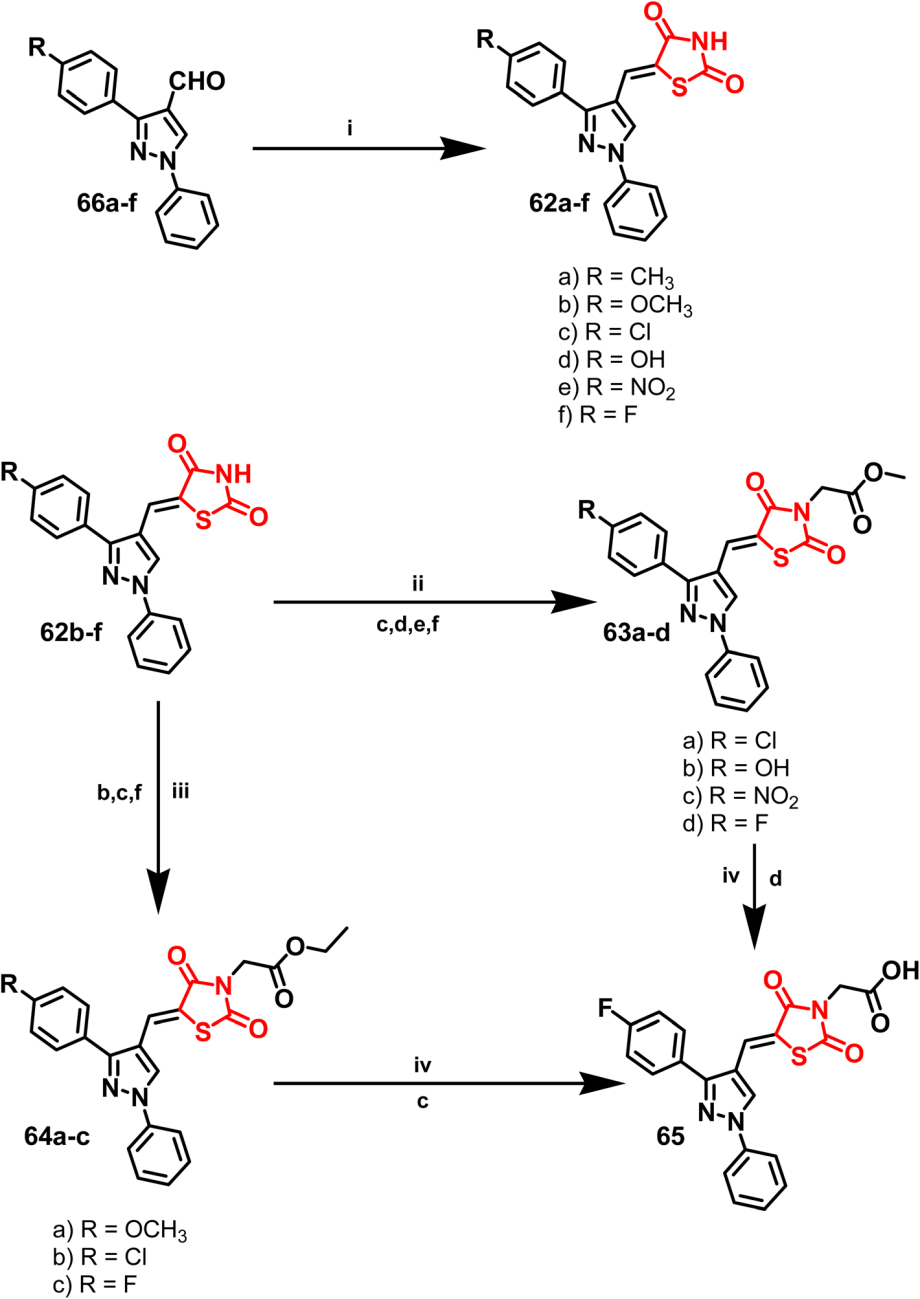
Synthesis of the pyrazolyl-2,4-thiazolidinedione 62a–f, 63a–d, 64a–c and 65. Reagents and conditions: (i) 2,4-thiazolidinedione, gl. AcOH, anhdrous sodium acetate, reflux, 6–8 h; (ii) ClCH_2_COOCH_3_, K_2_CO_3_, (CH_3_)_2_CO, reflux, 8–10 h; (iii) ClCH_2_COOCH_2_CH_3_, K_2_CO_3_, (CH_3_)_2_CO, reflux, 8–10 h; (iv) sodium hydroxide, EtOH, reflux, 10 h.

New sets of pyrazolyl-2,4-thiazolidinedione 62a–f, 63a–d, 64a–c and 65 were obtained as described in Scheme 16.^78^

**Fig. 16:**
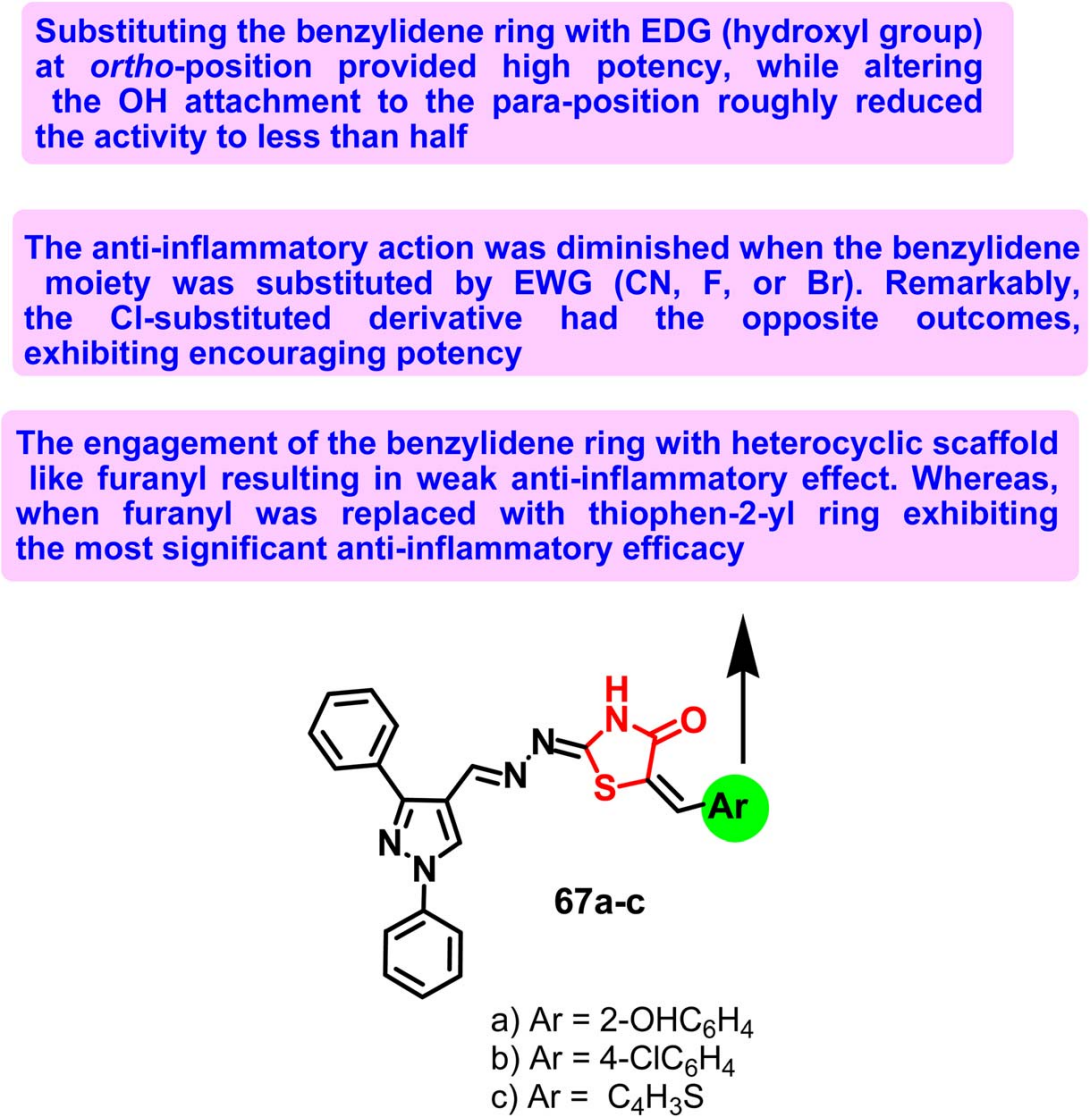
Structures of pyrazole-methylenehydrazono-thiazolidinone hybrids 67a–c as anti-inflammatory agents targeting TNF-α.

The production and screening of novel pyrazole-methylenehydrazono-thiazolidinone hybrids as potential anti-inflammatory agents were demonstrated by Abd El-Karim *et al.*^79^ Derivatives 67a–c displayed the highest anti-inflammatory efficacy four hours after administration with edema inhibition of 96.73%, 88.81% and 98.16%, respectively, surpassing that of celecoxib (73.4%). Moreover, on the first hour, they showed a quick onset of action with edema inhibition of 78.30%, 78.29% and 84.42%, respectively, demonstrating the long-lasting and quick onset of anti-inflammatory action. In addition, the evaluated candidates had a better GIT safety profile with ulcer severity ranging from 0 to 17.90 ± 1.60 compared to indomethacin (ulcer severity = 23.70 ± 1.28). Furthermore, derivatives 67a–c demonstrated outstanding TNF-α suppression effects (serum level = 34.13, 48.15 and 91.63 pg mL^−1^, respectively) comparable to celecoxib (50.65 pg mL^−1^). Substituting the benzylidene ring with EDG (hydroxyl group) at the *ortho*-position provided high potency, while altering the OH attachment at the *para*-position roughly reduced the activity to less than half. However, the anti-inflammatory action was diminished when the benzylidene moiety was substituted by EWG (CN, F, or Br). Remarkably, the Cl-substituted derivative had the opposite outcomes, exhibiting encouraging potency. Otherwise, the engagement of the benzylidene ring with a heterocyclic scaffold such as furanyl results in weak anti-inflammatory effects, whereas, when furanyl is replaced with a thiophen-2-yl ring, the most significant anti-inflammatory efficacy is observed. Moreover, derivatives 67a–c presented a high binding affinity for TNF-α. They were able to fit into the binding pocket through an arene–arene interaction between the amino acid Tyr119 and the pyrazole moiety. The thiazolidinone scaffold in compound 67b was seen to share fixation through a H-bond donor between Tyr151's side chain and NH group. Compound 67a's 2-hydroxyl group at the benzylidene ring created two H-bond acceptors with Gly121 (Fig. 16).

**Scheme 17:**
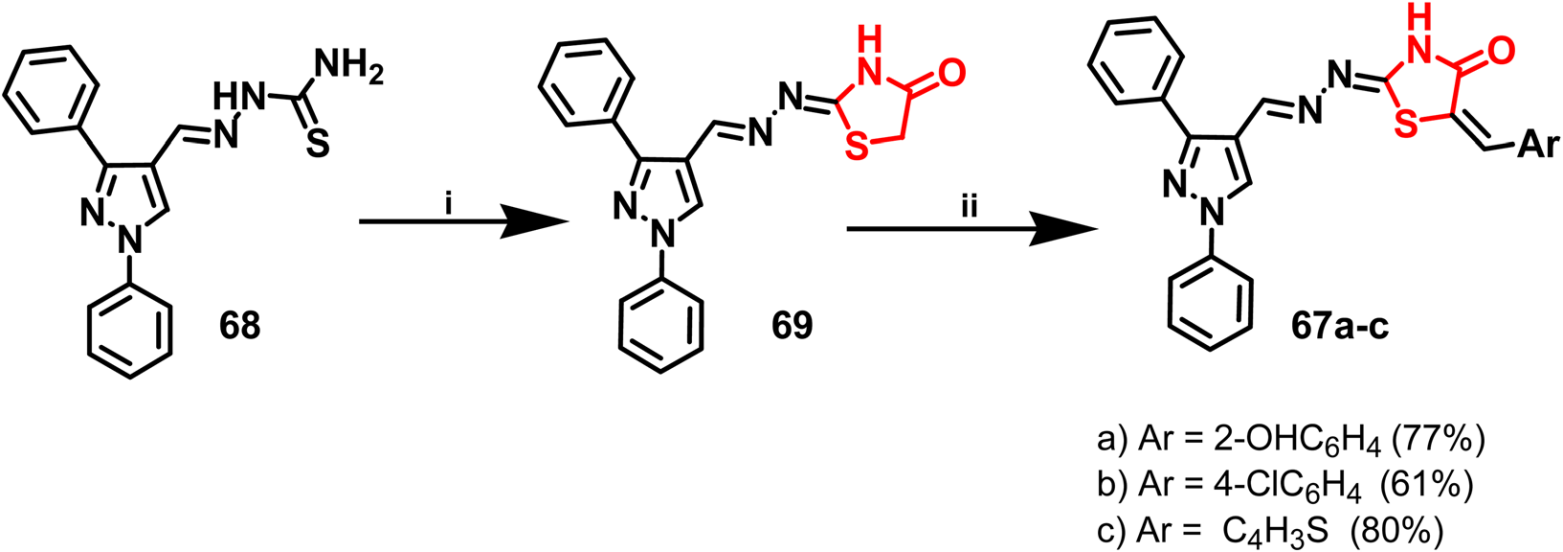
Synthesis of the pyrazole-methylenehydrazono-thiazolidinone hybrids 67a–c. Reagents and conditions: (i) BrCH_2_COOEt, EtOH, piperidine, reflux for 3 h; (ii) aromatic/heterocyclic aldehydes, alcoholic NaOH (10%), stirring overnight.

Thiazolidinone derivative 69 was accomplished by treating carbothioamide 68 with ethyl bromoacetate in refluxing ethanol that included piperidine. Stirring the intermediate 69 and the proper aldehydes in alcoholic sodium hydroxide at room temperature furnished the new pyrazole-methylenehydrazono-thiazolidinone hybrids 67a-c (Scheme 17).^79^

**Fig. 17:**
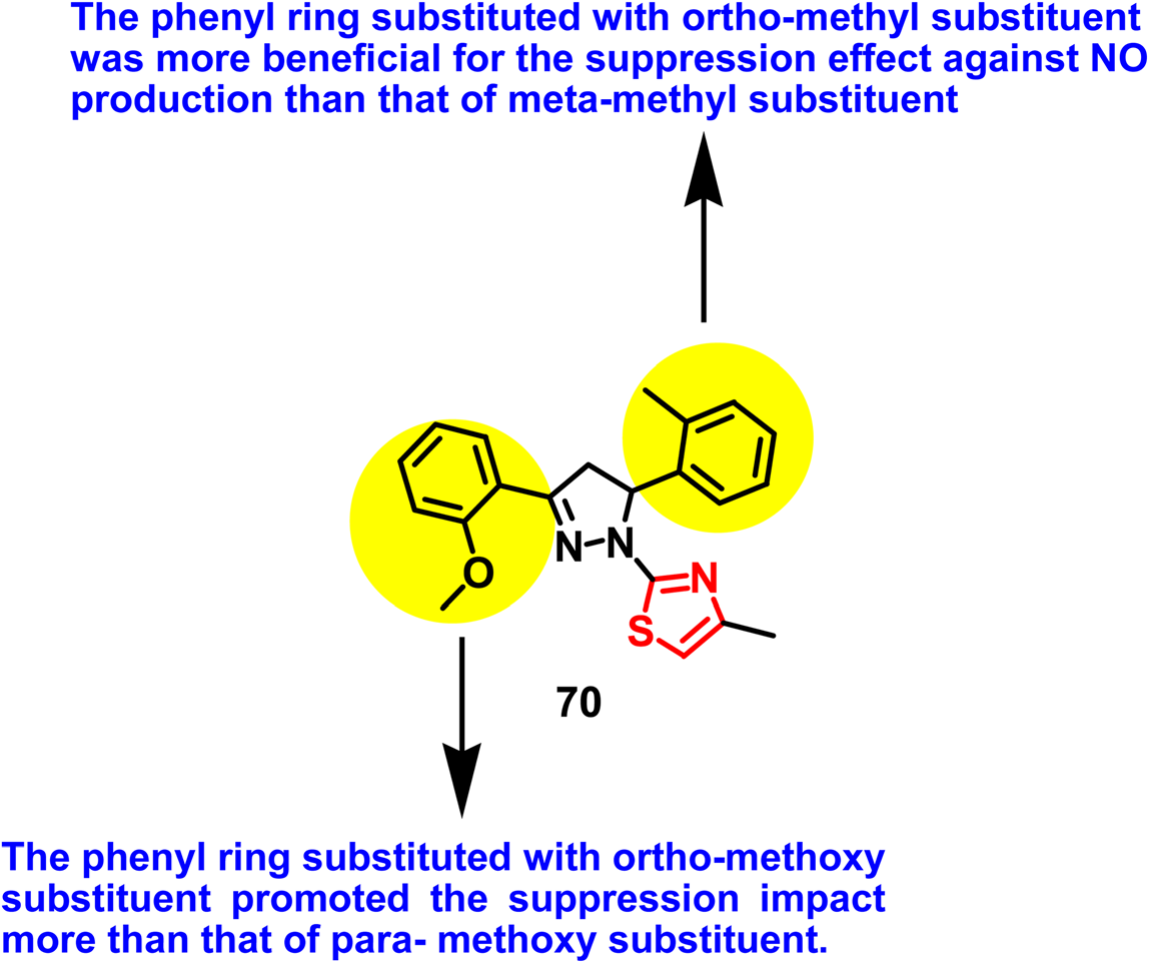
Structure of the thiazole-pyrazoline hybrid 70 as an anti-inflammatory agent targeting COX-2 enzyme and various inflammatory mediators.

A new thiazole-pyrazoline hybrid 70 disclosed the optimal suppression effects against NO production at various concentrations 1 and 10 µM. It was detected that the conjugation of the pyrazoline core at the 5th position with the phenyl ring substituted with an *ortho*-methyl substituent was more beneficial for the suppression effect against NO production than that of the *meta*-methyl substituent. However, the engagement of the pyrazoline core at the 3rd position with the phenyl ring substituted with an *ortho*-methoxy substituent promoted the suppression impact more than that of the *para*-methoxy substituent. Furthermore, derivative 70 effectively diminished TNF-α and IL-1β mRNA levels in a concentration-dependent way. Moreover, derivative 70 suppressed the expressions of COX-2 and iNOS *via* inhibiting the MAPK signaling pathway. Additionally, the *in vivo* investigation indicated that compound 70 prevented LPS-induced sepsis in C57BL/6J mice by reducing thespleen-, kidney- and heart-to-body weight ratios and efficiently decreasing serum AST, CREA, UREA, HBDH and LDH levels. E26 could be applied as a superior anti-inflammatory medication to treat sepsis^80^ (Fig. 17).

**Scheme 18:**
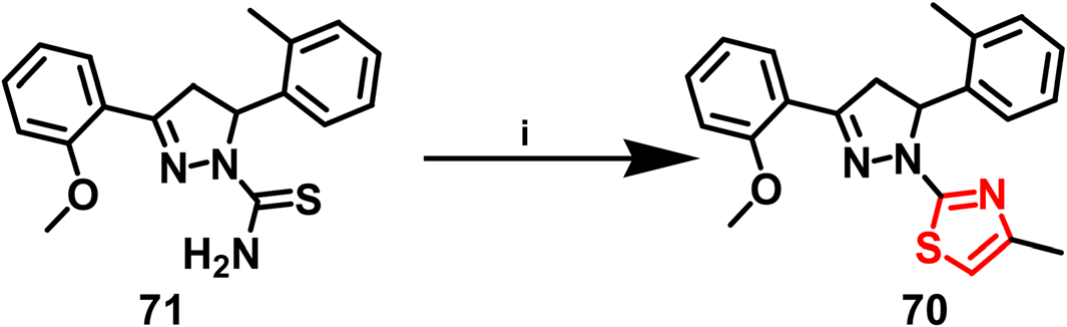
Synthesis of the thiazole-pyrazoline hybrid 70. Reagents and conditions: (i) chloroacetone, DMF, reflux, 3 h.

New thiazole-pyrazoline hybrid 70 was attained *via* refluxing the intermediate 71 with chloroacetone in DMF (Scheme 18).^80^

**Fig. 18:**
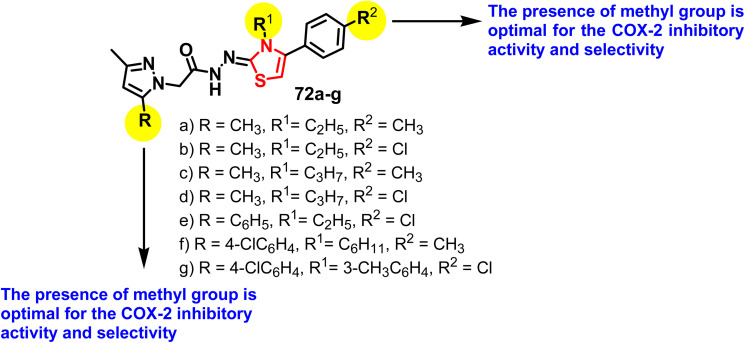
Structures of thiazole-pyrazole hybrids 72a–g as anti-inflammatory agents targeting the COX-2 enzyme.

Marzouk *et al.*^81^ described the preparation and the COX assessment of new thiazole-pyrazole hybrids. Among the investigated hybrids, seven derivatives 72a–g demonstrated eminent COX-2 suppression properties with IC_50_ values ranging from 0.05 to 0.07 µM as well as promising COX-2 selectivity ranging from 130 to 264, relative to celecoxib (IC_50_ = 0.05 µM, SI = 294). The presence of electron-donating substituents on both the thiazole (tolyl) and pyrazole (di-methyl) rings (derivative 72c) has validated the findings of Kamble *et al.*^8^ that an electron-donating substituent may be a pharmacophore for COX-2 selective inhibition. Furthermore, derivatives 72a–d manifested notable *in vivo* anti-inflammatory impacts (% protection = 87.84%, 77.14%, 92.20% and 97.30%, respectively). Derivative 72b revealed mild mucosal injury with an ulcer index = 120. Light microscopic examinations displayed that the stomach treated with 72b had nearly intact mucosa showing only localized lesions in the form of tissue loss or exfoliation. Furthermore, derivative 72b expressed highly significant difference to control in all parameters except in MDA activities (derivative 6b, MDA = 12.47 nmol mg^−1^, CAT = 5.35 ng mg^−1^, SOD = 132.75 U mg^−1^ and GSH = 112.27 ng mg^−1^) as compared to the control (MDA = 7.84 nmol mg^−1^, CAT = 8.76 ng mg^−1^, SOD = 168.75 U mg^−1^ and GSH = 158.34 ng mg^−1^). In addition, the most prominent COX-2 inhibitor 72c had a higher affinity and docking score into the active site of COX-2 (−9.4 kcal mol^−1^) relative to celecoxib (−8.9 kcal mol^−1^). Arg 120 is involved in the H-bond with the carbonyl group. Additionally, van der Waals interactions with Ser530, which is a key component of the majority of COX-2 inhibitory complexes, His90, which is an essential side pocket of the COX active site that creates the main connecting point that contributes to the substrate inhibitory binding, and Tyr-348, which is situated in the COX-2 active site, hold a very important therapeutic position. Furthermore, it demonstrated alkyl interaction with Val349, Leu352 and Val523. According to the pharmacokinetic effects of derivatives 72a–g on the cytochrome P450 enzymes, CYP2C19, CYP2C9, and CYP3A4 are projected to be inhibited by all the investigated candidates, although CYP1A2 and CYP2D6 are predicted to have no chance of inhibition. Moreover, derivative 72e was anticipated to be a P-gp substrate (inducer). While compounds 72a–e were projected to be significantly absorbed passively through the GIT, all other compounds were predicted not to cross the BBB. Lipinski's RO5 was not violated by compounds 72a–e, suggesting that these molecules had good oral bioavailability (Fig. 18).

**Scheme 19:**
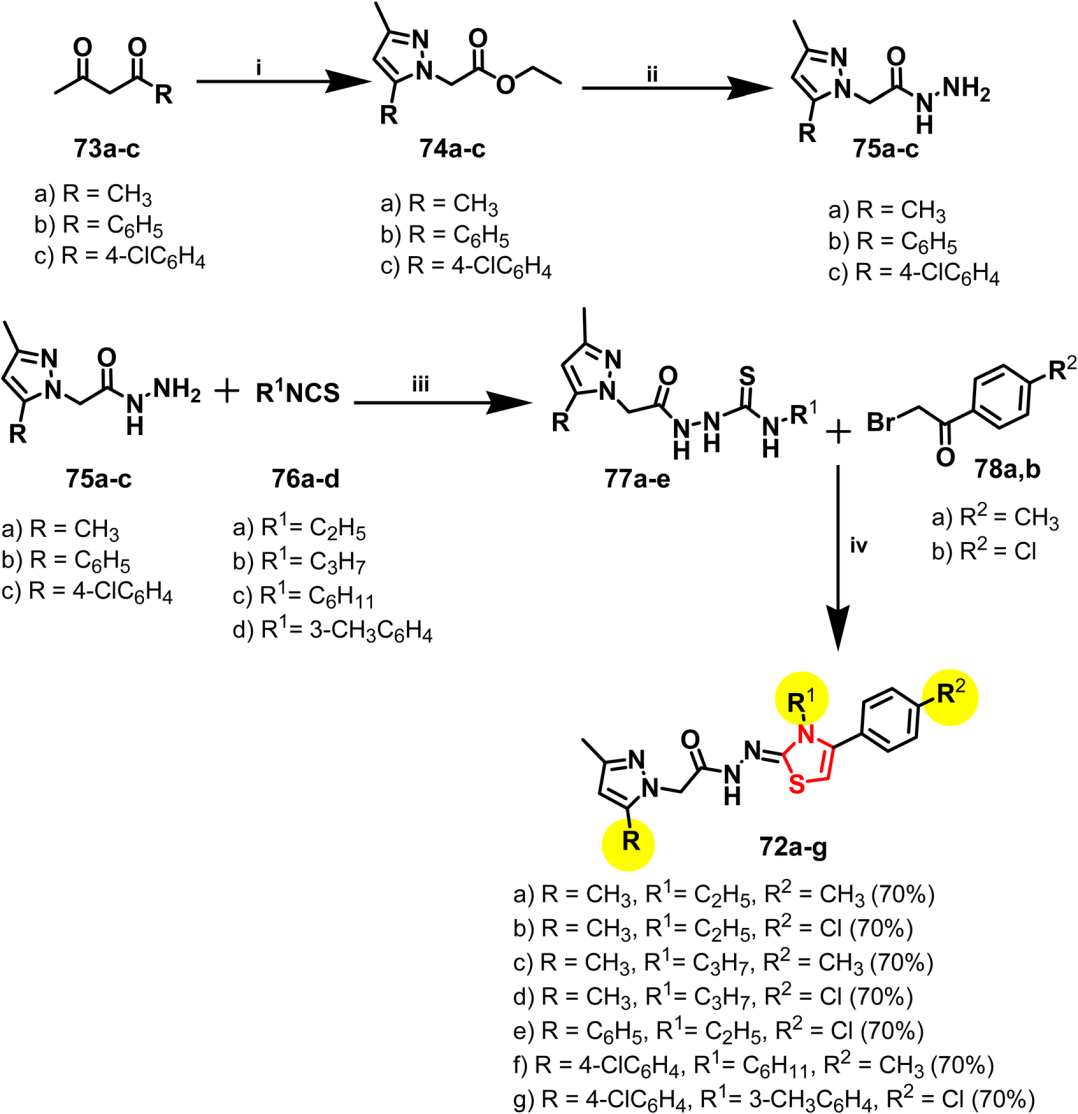
Synthesis of the thiazole-pyrazole hybrids 72a–g. Reagents and conditions: (i) ethyl hydrazine acetate hydrochloride, distilled water, sodium bicarbonate, gl.AcOH, reflux, 2 h; (ii) hydrazine hydrate, ethanol, reflux, 3 h; (iii) ethanol, reflux, 6 h; (iv) ethanol, triethylamine, reflux, 3 h.

Cyclocondensation of the appropriate 1,3-dicarbonyl compounds 73a–c with ethyl hydrazine acetate hydrochloride afforded the pyrazole ester derivatives 74a–c, while the hydrazide derivatives 75a–c were obtained *via* hydrazinolysis of the pyrazole ester derivatives 74a–c in an ethanolic solution. The treatment of the hydrazide derivatives 75a–c with appropriate isothiocyanate derivatives 76a–d yielded the thiosemicarbazide intermediates 77a–e, which was then subjected to cyclization through a reaction with appropriate phenacyl bromide derivatives 78a, b to furnish the target thiazole-pyrazole hybrids 72a–g (Scheme 19).^81^

**Fig. 19:**
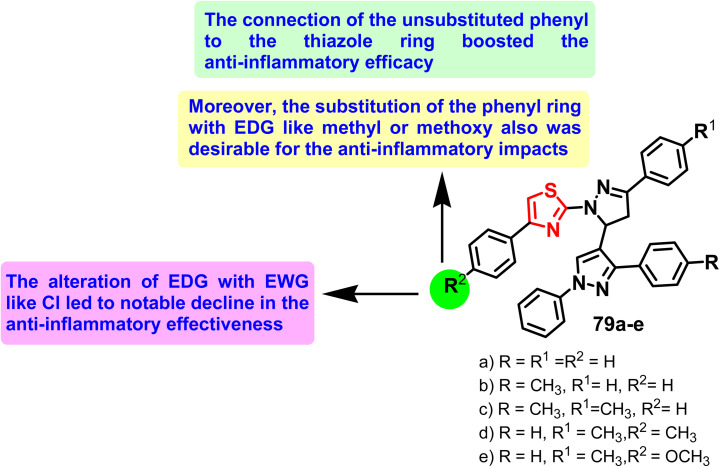
Structures of thiazolyl pyrazoline candidates 79a–e as anti-inflammatory agents.

New thiazolyl pyrazoline candidates 79a–e were discovered as eminent anti-inflammatory agents. The anti-inflammatory impacts were assessed utilizing the protein denaturation method. The inhibition of protein denaturation of 79a–e was 87.15%, 86.23%, 91.74%, 88.07% and 87.15%, respectively, comparable to diclofenac sodium (inhibition = 90.82%). It was pointed out that the connection of the thiazole ring to unsubstituted phenyl boosted the anti-inflammatory efficacy. Moreover, the substitution of the phenyl ring with EDG such as methyl or methoxy was desirable for the anti-inflammatory impacts; however, the alteration of EDG with EWG such as Cl led to a notable decline in the anti-inflammatory effectiveness^82^ (Fig. 19).

**Scheme 20:**
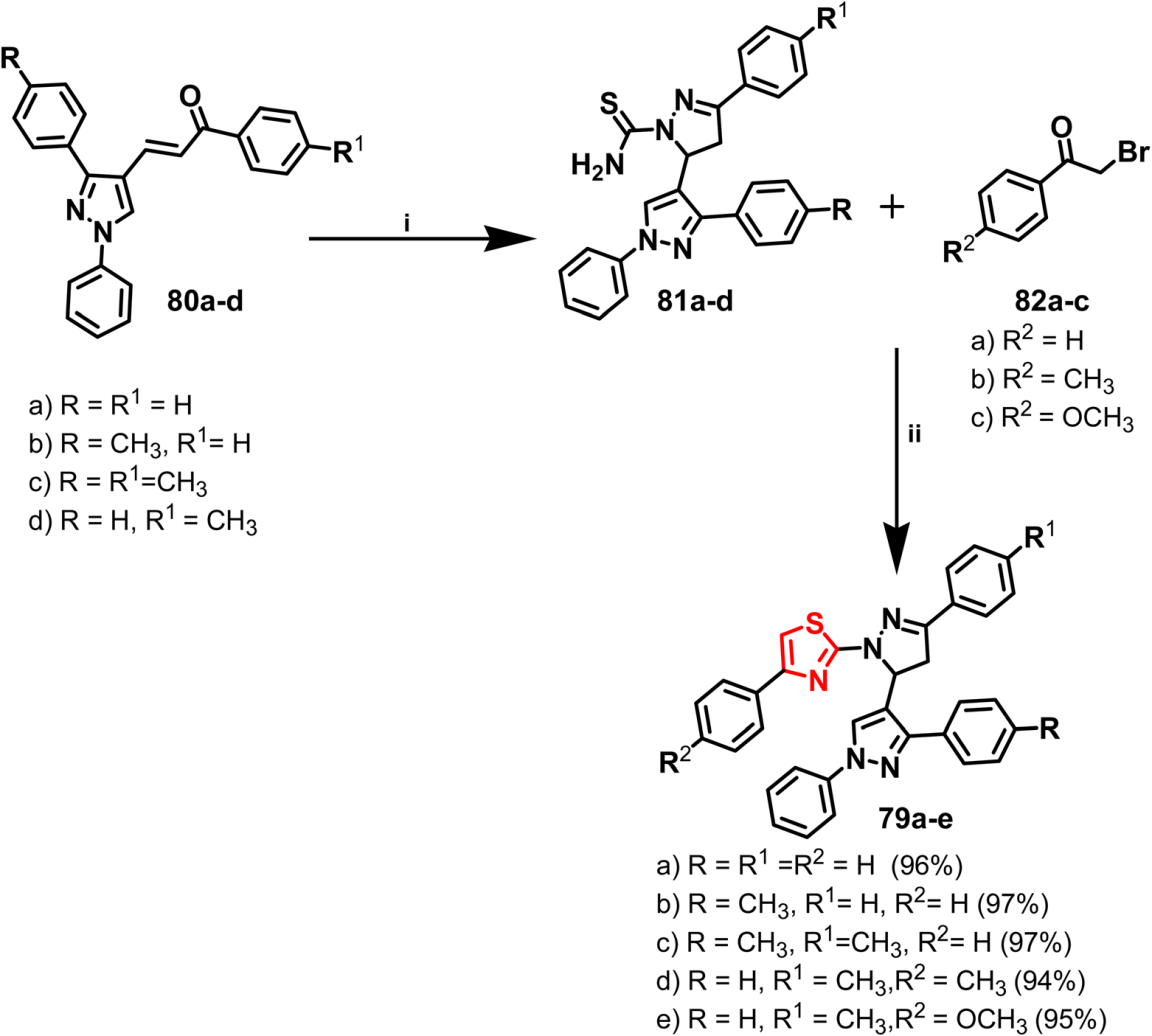
Synthesis of the thiazolyl pyrazoline candidates 79a–e. Reagents and conditions: (i) NH_2_CSNHNH_2_, KOH, ethanol (absolute) reflux, 4–5 h; (ii) PEG-300, rt, 1–2 h.

1-Thiocarbamoyl pyrazole derivatives 81a–d were produced by cyclizing chalcones 80a–d with thiosemicarbazide in the presence of potassium hydroxide in refluxing ethanol. In the last stage, 1-thiocarbamoyl pyrazole derivatives 81a–d and suitable phenacylbromides 82a–c were mixed at room temperature in the presence of PEG-300, a green reaction medium to afford the new thiazolyl pyrazoline candidates 79a–e (Scheme 20).^82^

**Fig. 20:**
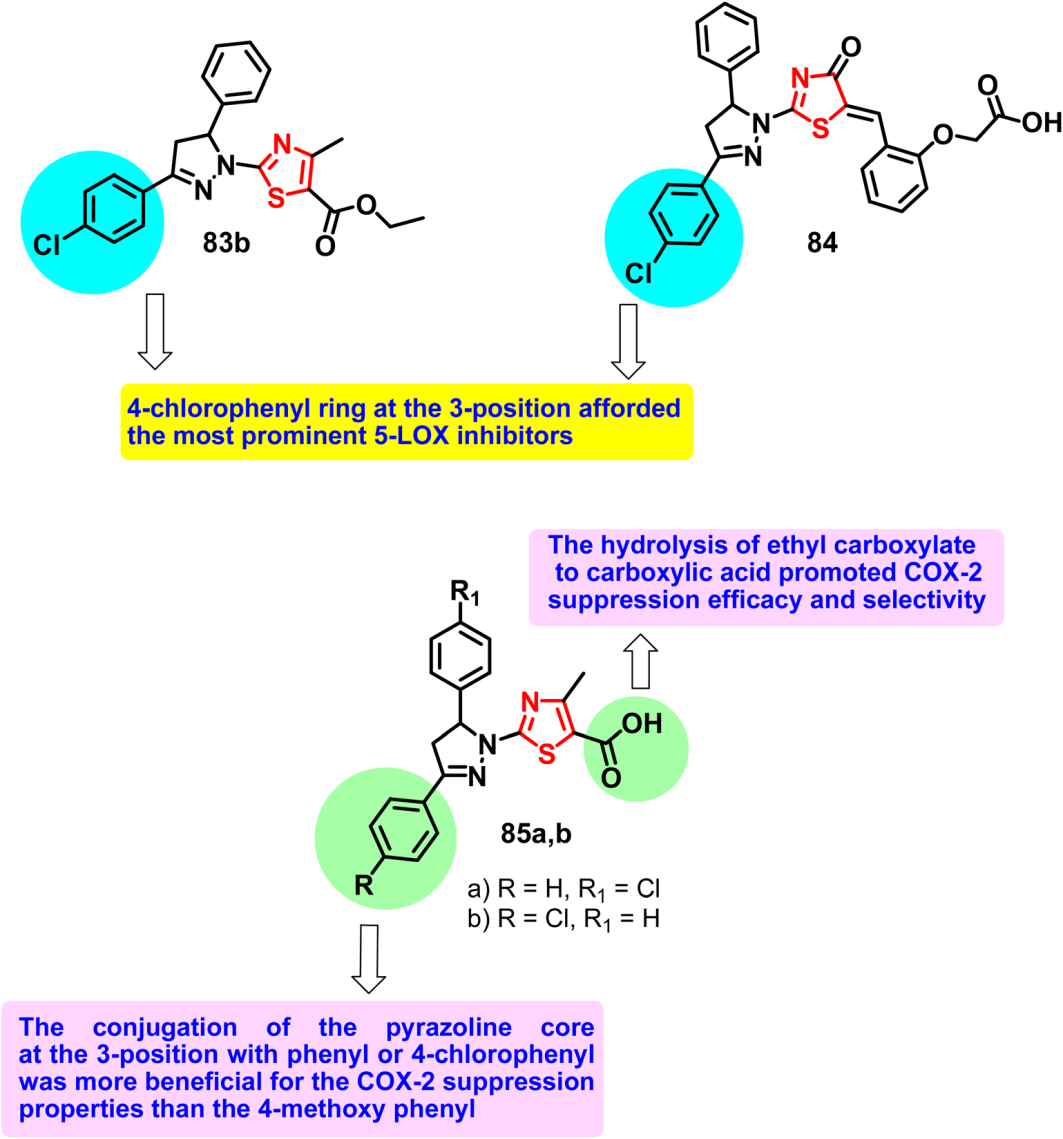
Structures of thiazole/thiazolidinone-pyrazoline hybrids 83b, 84 and 85a, b as anti-inflammatory agents targeting dual 5-LOX and COX-2 enzymes.

New thiazole/thiazolidinone-pyrazoline hybrids stood out as dual 5-LOX and COX-2 suppressors. It was detected that hybrids 83b and 84 demonstrated outstanding 5-LOX inhibitory effectiveness (IC_50_ = 2.40 and 1.58 µM, respectively), while derivatives 85a, b were identified as eminent and selective COX-2 inhibitors (IC_50_ = 0.04 and 0.03 µM, respectively; SI = 385 and 472.9, respectively). Moreover, the *in vivo* assay disclosed that derivatives 83b and 85a, b effectively alleviated the paw edema by nearly 83%, 91% and 87% in comparison to the untreated model. Compared to control or P.E. model rats, rats treated with derivative 85a displayed normal morphological characteristics, such as intact submucosa, glandular structures, outer muscular coat and serosa. With IC_50_ values of 603 and 452 µM against Vero and BNL cells, respectively, derivative 85a showed no cytotoxic influence against either cell line. Interestingly, the pyrazoline core bearing a 4-chlorophenyl ring at the 3-position afforded the most prominent 5-LOX inhibitors. Otherwise, it was discovered that the functional group at the 5-position of the thiazole scaffold had an impact on the COX-2 suppression effectiveness and selectivity of thiazole-pyrazoline hybrids. The hydrolysis of ethyl carboxylate to carboxylic acid promoted COX-2 suppression efficacy and selectivity. Moreover, it was noted that the conjugation of the pyrazoline core at the 3-position with phenyl or 4-chlorophenyl was more beneficial for the suppression properties than the 4-methoxy phenyl. Additionally, derivatives 83b, 84 and 85a, b satisfied both Lipinski's and Veber's requirement and were anticipated to have sufficient intestinal absorption in humans, suggesting that they may have advantageous oral absorption. Low BBB penetration was also predicted; thus, it won't have a negative impact on the CNS. Furthermore, it is anticipated that these derivatives will not be effluxed from the cells and will not act as substrates of permeability glycoprotein (P-gp). Docking of 85a inside the COX-2 active site indicated varied hydrophobic interactions between the chloro groups with Trp387, Leu384, Met522 and Tyr385. Furthermore, the 3-phenyl ring is stabilized by hydrophobic interactions with Leu359, Val116, Leu531 and Val-349 and pi–cation interaction with Arg120. Meanwhile, the thiazole core is engaged in π–sulfur interaction with His90, whereas the carboxylate moiety participates in H-bonds with Arg513 and Ser353 (ref. 83) (Fig. 20).

**Scheme 21:**
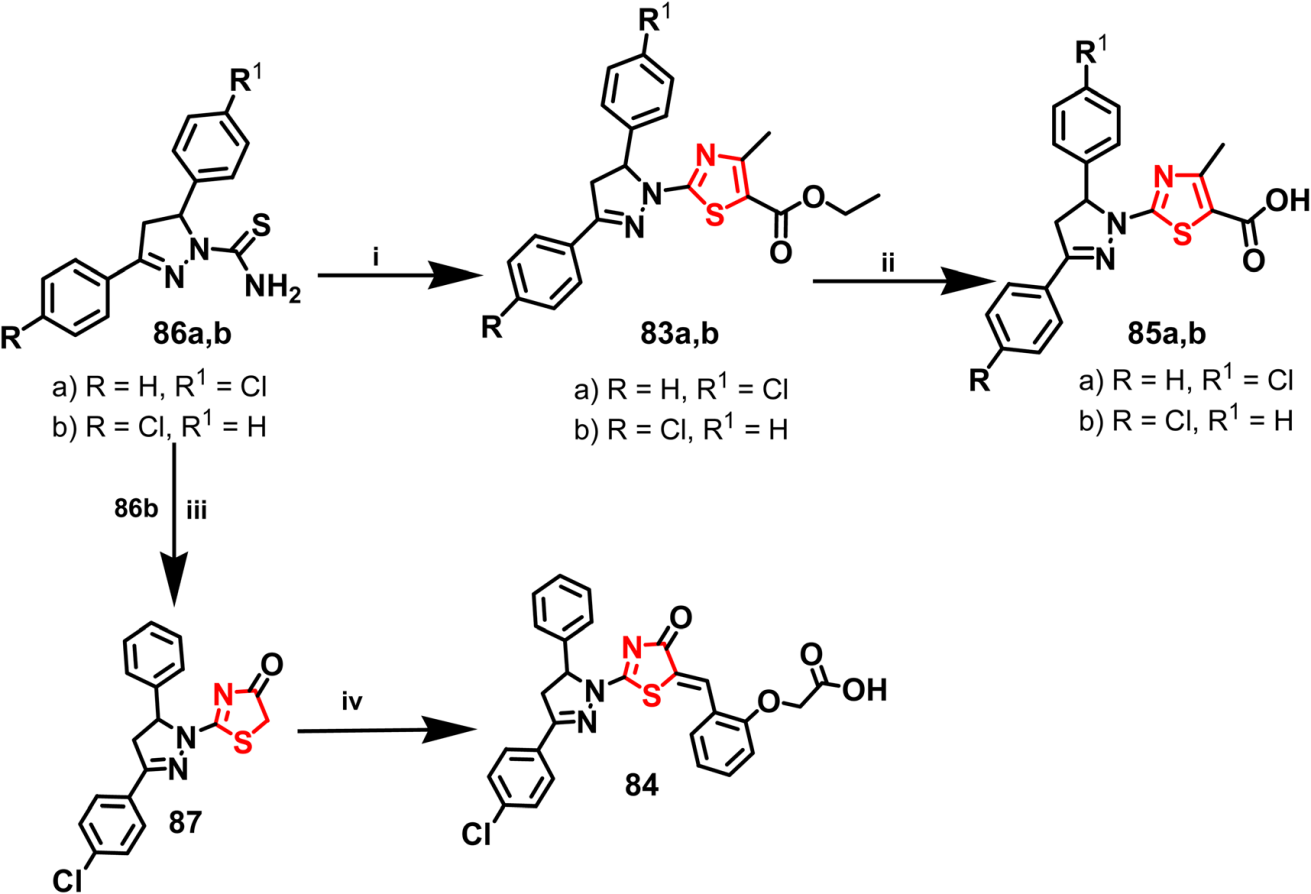
Synthesis of the thiazole/thiazolidinone-pyrazoline hybrids 83a, b, 84 and 85a, b. Reagents and conditions: (i) ethyl 2-chloroacetoacetate, Abs. EtOH, reflux, 10 h; (ii) NaOH, MeOH, reflux 4–6 h then neutralized with Conc HCl; (iii) bromoacetic acid, abs. EtOH, anhydrous CH_3_COONa, reflux, 5h; (iv) 2-(2-formylphenoxy)acetic acid, glacial acetic acid, anhydrous sod. Acetate, reflux, 12 h.

Target thiazole/thiazolidinone-pyrazoline hybrids 85a, b, 84 and 85a, b were accomplished starting from pyrazoline carbothioamide derivatives 86a, b, as depicted in Scheme 21.^83^

**Fig. 21:**
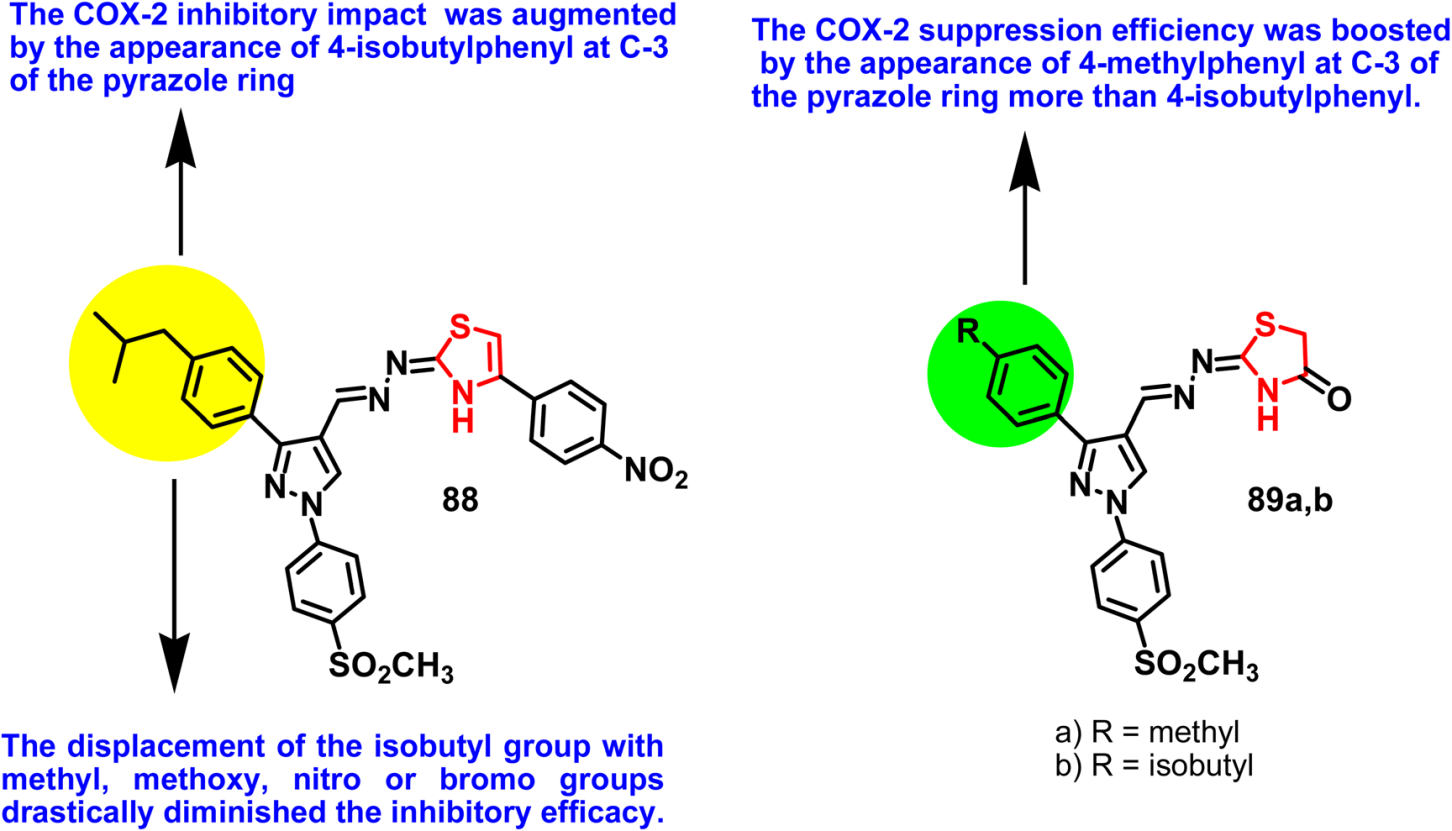
Structures of thiazole/thiazolidinone-pyrazole hybrids 88 and 89a, b as COX-2 inhibitors.

Novel thiazole/thiazolidinone-pyrazole hybrids 88 and 89a, b unveiled eminent selective and COX-2 inhibitory impacts (IC_50_ = 0.82, 0.35 and 0.76 µM, respectively) with SI = 42.13, 134.6 and 26.08, respectively, with respect to celecoxib (IC_50_ = 0.69, SI = 24.09). It was discovered that thiazolidinone-pyrazole hybrids 89a, b augmented the COX-2 suppression influence more than the thiazole-pyrazole hybrid. The COX-2 suppression efficiency of the thiazolidinone-pyrazole hybrids was boosted by the appearance of 4-methylphenyl at C-3 of the pyrazole ring more than 4-isobutylphenyl. Inversely, the COX-2 inhibitory impact of the thiazole-pyrazole hybrids was augmented by the appearance of 4-isobutylphenyl at C-3 of the pyrazole ring, whereas the displacement of the isobutyl group with methyl, methoxy, nitro or bromo groups drastically diminished the inhibitory efficacy. In addition, derivatives 88 and 89a, b possessed 6–7 H-bond acceptors and 1 H-bond donor, while the number of rotatable bonds ranged from 5 to 9 bonds. Only the derivative 89a demonstrated a satisfactory molar refractivity value of 126.21 m^3^ mol^−1^, but the molar refractivity values of derivatives 88 and 89b were 167.57 and 140.63 m^3^ mol^−1^, which suggested that the derivatives 88 and 89b may have poor oral bioavailability and GI absorption. Moreover, derivatives 88 and 89a, b disclosed a high TPSA value ranging from 139.46 to 174.91, which showed their inability to cross the BBB. The estimated values of *I* log *P* were found to be less than five (3.15–3.39), as proposed by Lipinski's rule of five. It had been assumed that the thiazolidinone derivatives 89a, b would block the CYP2C19 and CYP2C9 enzymes, lessening their susceptibility to inhibitory drug metabolism. In summary, the thiazolidinone-pyrazole hybrids 89a, b have a suitable bioavailability score of 0.55 and no violation against Lipinski's rule. Otherwise, thiazole-pyrazole hybrid 88 unveiled 2 violations of Lipinski's rule with a low score of bioavailability of 0.17, demonstrating its low oral-drug similarity. Derivatives 88 and 89a, b demonstrated docking scores varying from −3.5 to −3.7 kcal mol^−1^ as compared to celecoxib (−4.9 kcal mol^−1^). Regarding the derivative 88, His75 and Leu338 contributed to H-bonds with NH and S atoms of the thiazole ring, respectively; hydrophobic interaction also occurred between the thiazole ring and Thr79. Concerning derivatives 89a, b, the azo groups (N=N) are involved in H-bonds with Ala513 and Arg499, respectively, while the SO_2_CH_3_ groups contributed to H-bonds with Phe504 and Pro71, respectively. The presence of thiazole, azo groups, or SO_2_CH_3_ moieties may be accountable for these hybrids' increased selectivity index^84^ (Fig. 21).

**Scheme 22:**
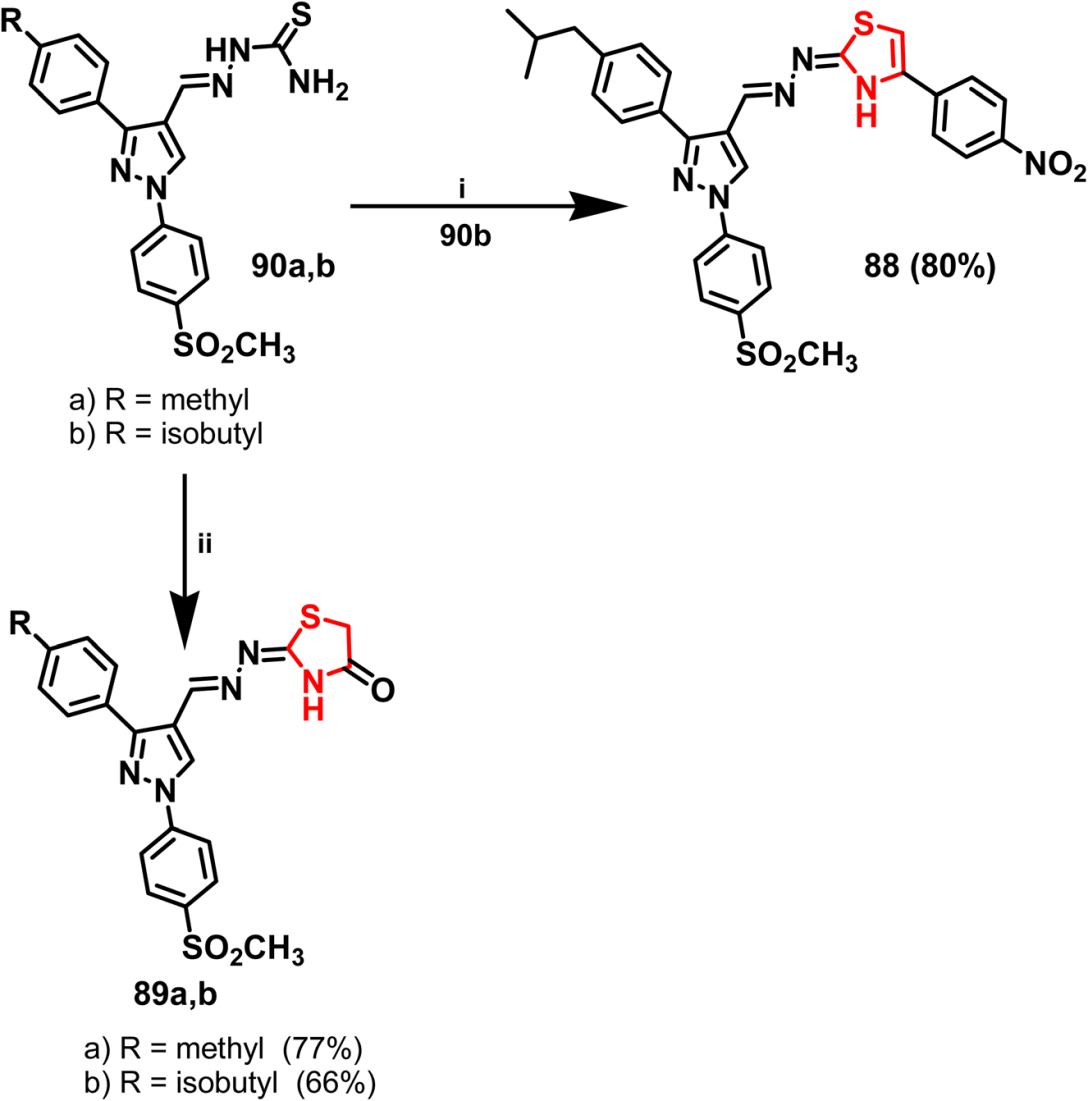
Synthesis of the thiazole/thiazolidinone-pyrazole hybrids 88 and 89a, b. Reagents and conditions: (i) 4-nitrophenacyl bromide, ethanol 95%, pyridine, reflux, 24 h; (ii) ethyl chloro acetate, ethanol 95%, CH_3_COONa, reflux, 24 h.

The cyclization of the thiosemicarbazone 90b with 4-nitrophenacyl bromide derivatives furnished the thiazole-pyrazole hybrid 88, while the target thiazolidinone-pyrazole hybrids 89a, b were obtained *via* the cyclization of thiosemicarbazones 90a, b with ethyl chloroacetate (Scheme 22).^84^

**Fig. 22:**
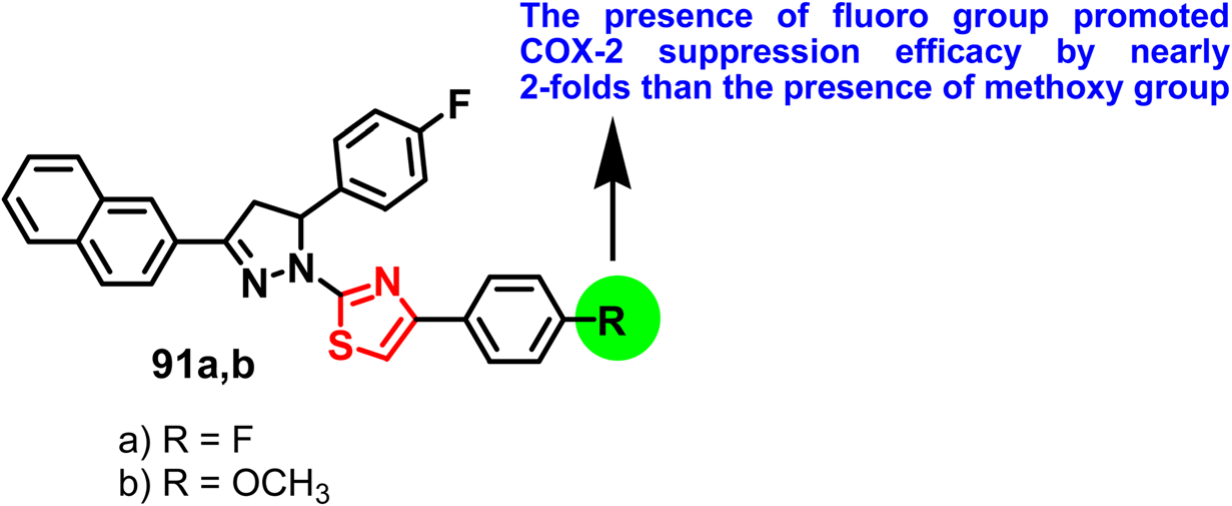
Structures of naphthalene-bearing thiazolylpyrazole ring systems 91a, b as anti-inflammatory agents targeting the COX-2 enzyme.

One year later, *in vivo* anti-inflammatory investigation of a new set of naphthalene-bearing thiazolylpyrazole ring systems was performed. Two derivatives 91a, b demonstrated a prominent edema inhibition percentage after 3 h (72.12% and 79.39%, respectively), and low ulcer indices (3.2 and 3.8, respectively). Moreover, the COX suppression assay revealed that derivatives 91a, b presented a good COX-2 suppression impact (IC_50_ = 87.74 and 166.5 nM, respectively) and low selectivity (SI = 2.05 and 0.23, respectively) relative to celecoxib (IC_50_ = 53.30 nM, SI = 8.31). It was obvious that the presence of a fluoro group on the phenyl ring attached to the thiazole scaffold promoted COX-2 suppression efficacy by nearly 2-folds compared to the presence of a methoxy group. Docking analysis of derivative 91a demonstrated H-arene interactions between the naphthalene rings with Phe504 and Ser339, in addition to hydrophobic interactions between the thiazolylpyrazole ring systems with Val509, Leu517 and Ala513, while the two 4-fluorophenyl rings displayed hydrophobic interactions with Phe367, Met99, Trp373, Leu370, Val102 and Leu103. The latter residue was engaged in H-bond with the fluoro group on the phenyl ring attached to the thiazole core. Furthermore, two H-bonds were created between N and S atoms of the thiazole ring with Leu517 and Ser516, respectively. An extra H-bond was formed between N-2 of the pyrazole core and Val509. Moreover, a UPLC-MS/MS assay was applied to determine the plasma level for derivative 91b in rats after a single subcutaneous dose (10 mg kg^−1^). Derivative 91b displayed remarkable pharmacokinetic parameters with a maximum concentration in the plasma (*C*_max_, 550.82 ng mL^−1^), which was accomplished at 2 h. In addition, the half-life (*t*_1/2_) of derivative 91b was 38.501 h, whereas AUC0–∞ was 4910.04 ng h mL^−1^ (ref. 85) (Fig. 22).

**Scheme 23:**
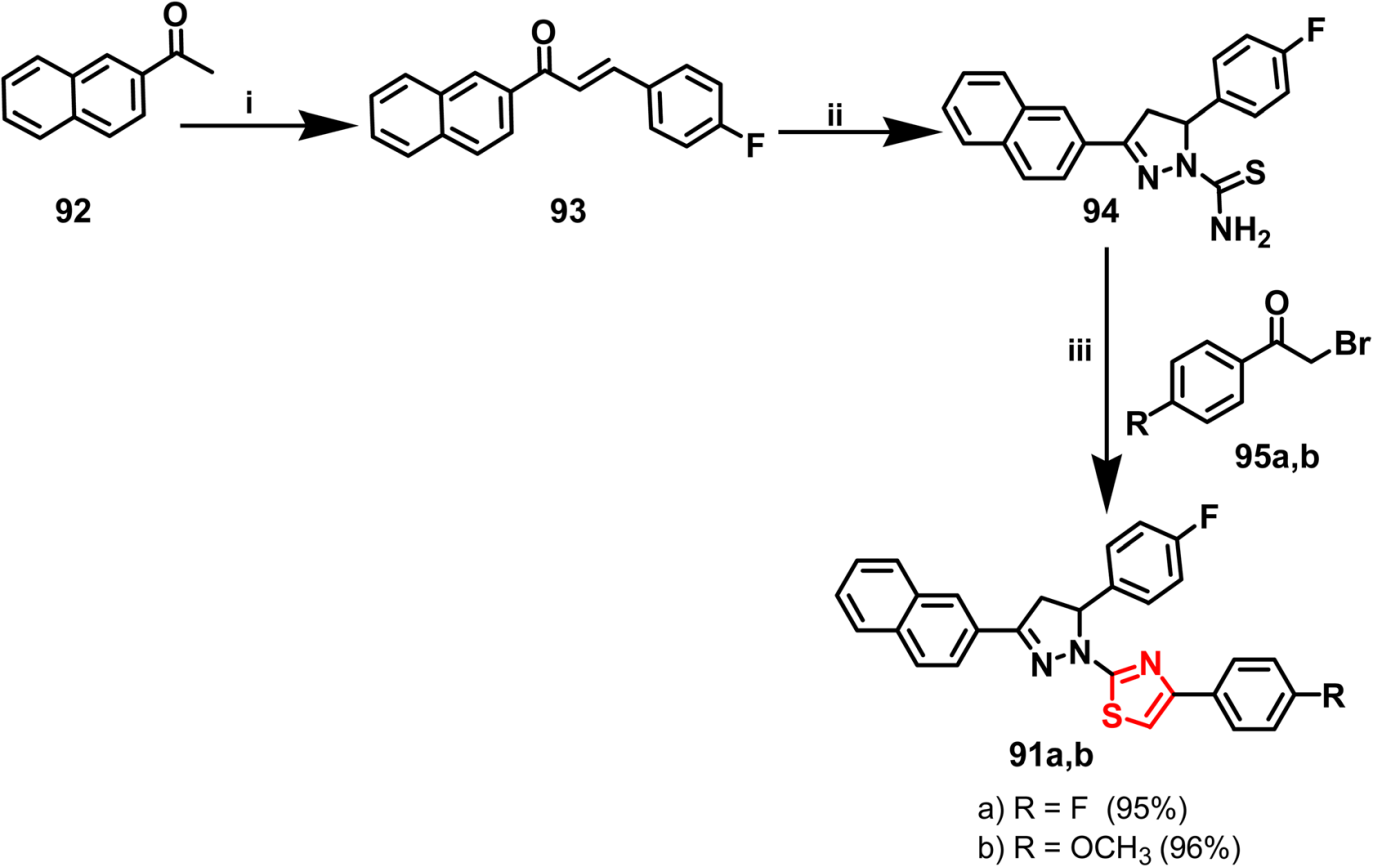
Synthesis of the naphthalene-bearing thiazolylpyrazole ring systems 91a, b. Reagents and conditions: (i) 4-fluorobenzaldehyde, ethanol, NaOH, rt, stirring overnight; (ii) NH_2_NHCSNH_2_, ethanol, NaOH, reflux, 8 h; (iii) ethanol, reflux, 6 h.

The intermediate 93 was prepared following the reported procedure.^86^ Finally, the pyrazole-1-carbothioamide intermediate 94 reacted with the appropriate phenacyl bromide derivatives 95a, b to furnish the target derivatives 91a, b (Scheme 23).^85^

**Fig. 23:**
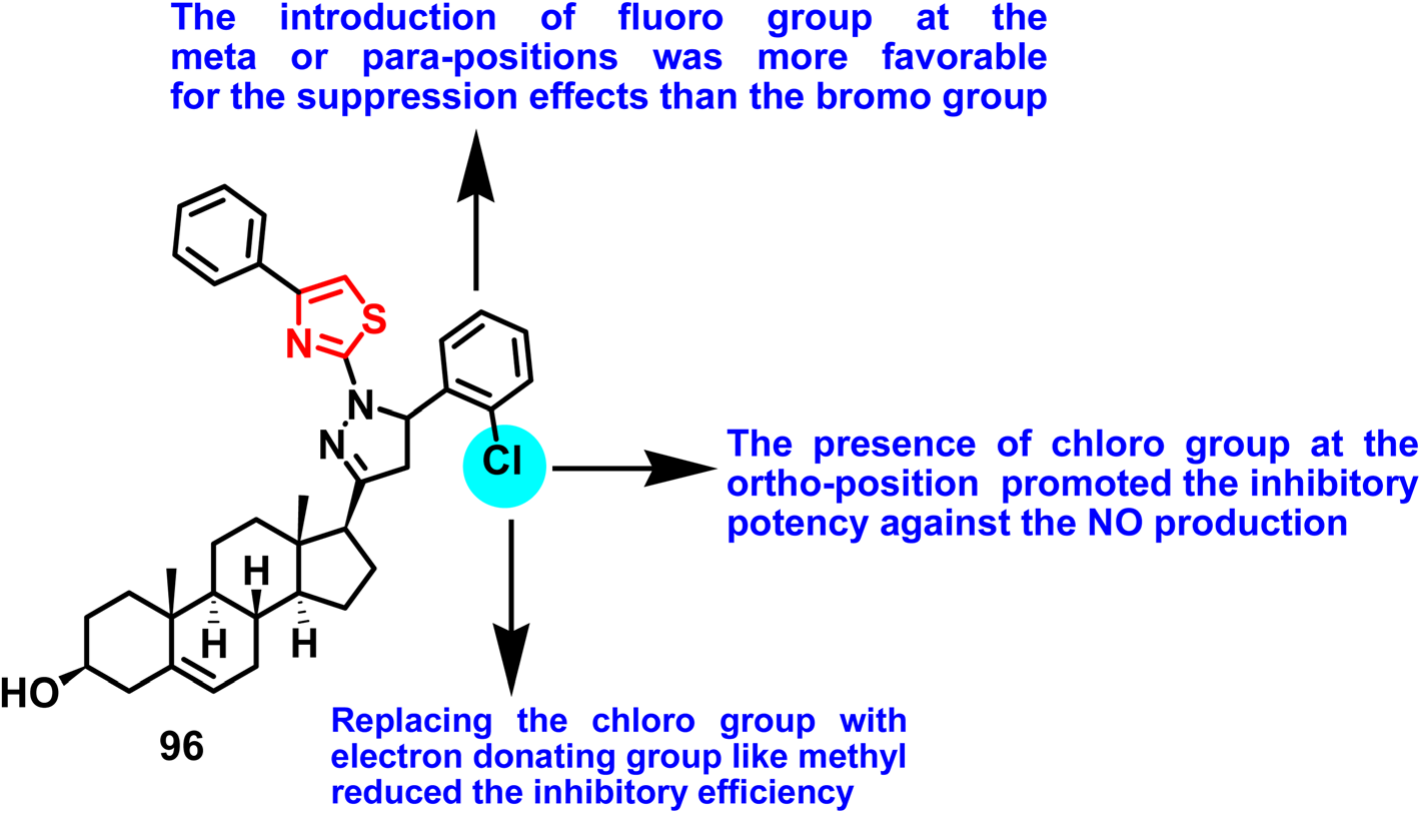
Structure of the D-ring-substituted steroidal 4,5-dihydropyrazole thiazole derivative 96 targeting COX-2 enzyme and various inflammatory mediators.

Fang *et al.*^87^ described the synthesis and the inhibitory evaluation of new D-ring-substituted steroidal 4,5-dihydropyrazole thiazole derivatives against NO, TNF-α, IL-6, iNOS and COX-2 in LPS-induced RAW 264.7 cells. Derivative 96 significantly suppressed the NO production with an IC_50_ value of 2.59 µM, exceeding that of the positive control methylprednisolone (MPS) (IC_50_ = 4.79 µM). SAR analysis detected that the presence of chloro group at the *ortho*-position on the phenyl ring attached to the pyrazoline moiety promoted the inhibitory potency against the NO production. However, replacing the chloro group with an electron-donating group such as methyl reduced the inhibitory efficiency by nearly 13-folds. In addition, the introduction of fluoro group at the *meta* or *para*-positions was more favorable for the suppression effects than the bromo group, while the presence of electron-donating groups at *meta* or *para* positions declined the inhibitory properties. Further studies disclosed that derivative 96 was able to downregulate the expression of other inflammatory mediators such as TNF-α, IL-6, iNOS and COX-2 in a concentration-dependent manner. Also, derivative 96 blocked the translocation of NF-κB p65 from the cytosol to the nucleus, indicating the inhibition of NF-κB translocation and activation. Moreover, it suppressed the LPS-induced phosphorylation of JNK1/2 and p38 MAPK, suggesting that derivative 96 exerted its *in vitro* anti-inflammatory effectiveness *via* the suppression of the MAPK signaling pathway (Fig. 23).

**Scheme 24:**
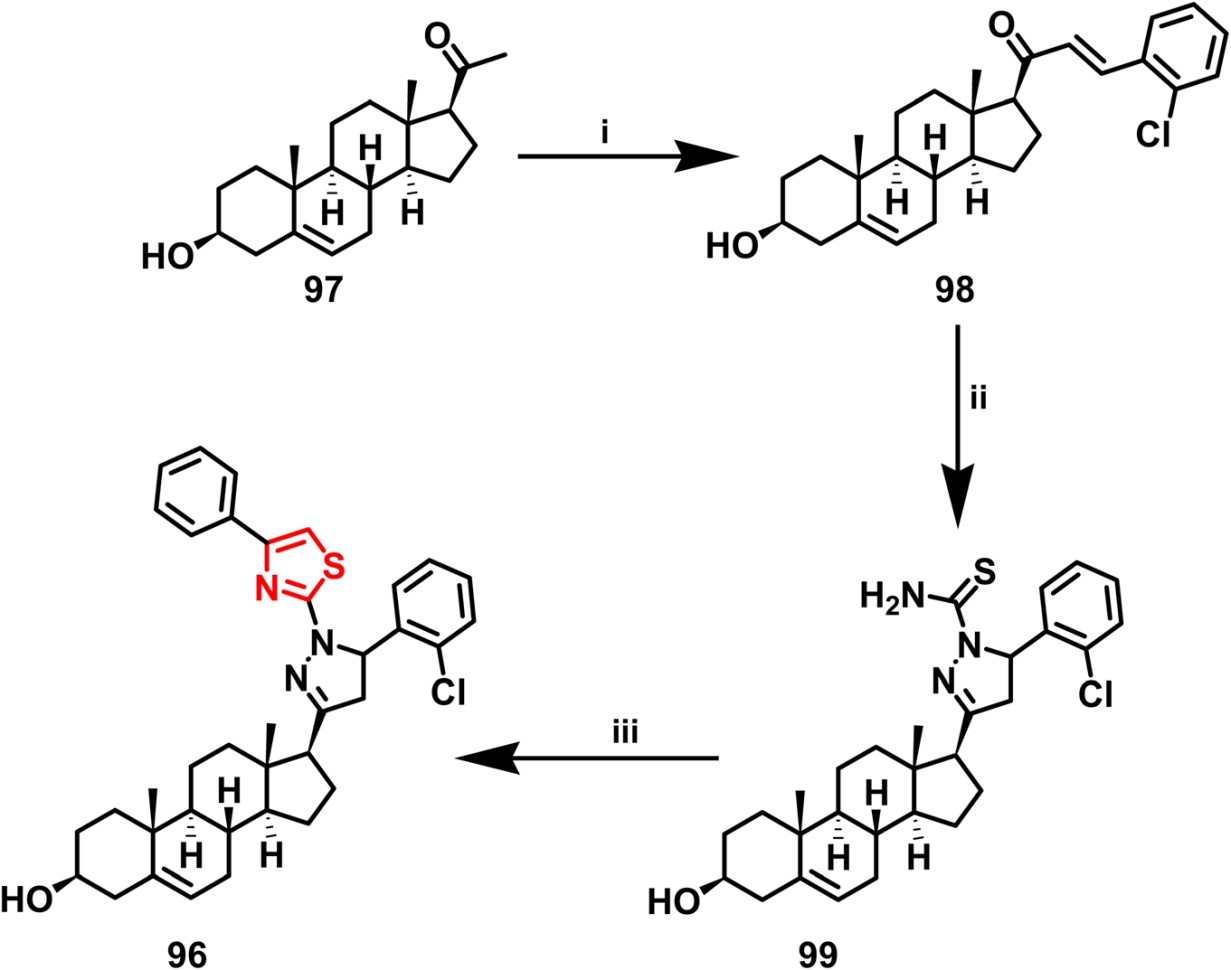
Synthesis of the D-ring-substituted steroidal 4,5-dihydropyrazole thiazole derivative 96. Reagents and conditions: (i) 2-chloro benzaldehyde, absolute ethanol, NaOH, rt, 48 h; (ii) NH_2_NHCSNH_2_, absolute ethanol, NaOH, reflux, 8 h; (iii) phenacyl bromide, reflux, 8 h.

The chalconyl pregnenolone derivative 98 was prepared *via* the Claisen–Schmidt condensation reaction of pregnenolone 97 with 2-chloro benzaldehyde in a basic medium. Furthermore, the steroidal pyrazoline thioamide 99 was obtained *via* the nucleophilic reaction of derivative 98 and thiosemicarbazide. Finally, the synthesis of the steroidal 4,5-dihydropyrazole thiazole derivative 96 was accomplished *via* the cycloaddition reaction of derivative 99 and phenacyl bromide (Scheme 24).^87^

**Fig. 24:**
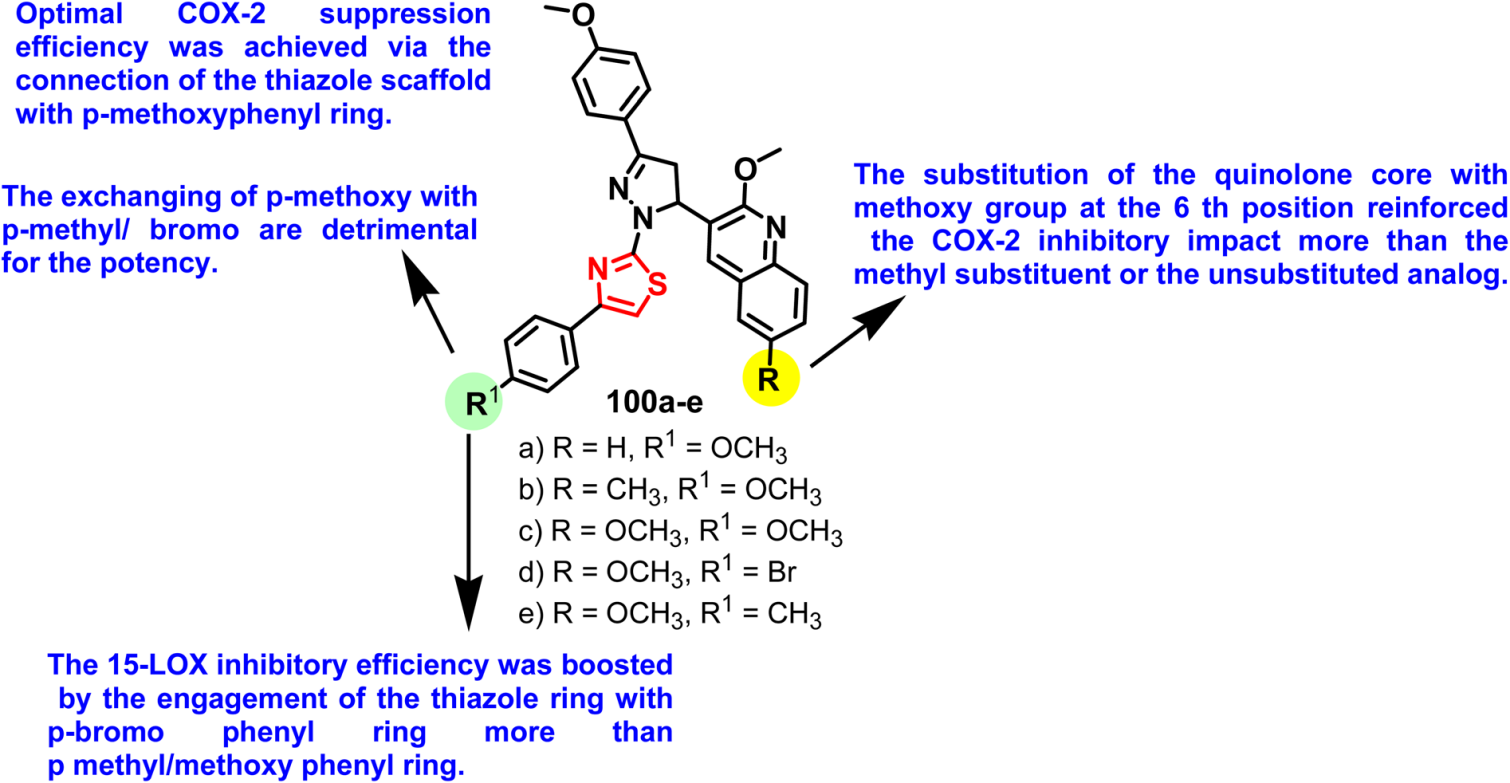
Structures of quinoline-containing thiazolylpyrazole derivatives 100a–e as anti-inflammatory agents targeting dual COX-2 and 15-LOX enzymes and various inflammatory mediators.

New quinoline-containing thiazolylpyrazole derivatives 100a–c protrude out as outstanding and selective COX-2 inhibitors (IC_50_ = 0.24, 0.23 and 0.20 µM, respectively, SI = 8.95, 20.35, and 12.42, respectively) with respect to celecoxib (IC_50_ = 0.512 µM, SI = 4.28). It was pointed out that the substitution of the quinolone core with the methoxy group at the 6th position reinforced the COX-2 inhibitory impact more than the methyl substituent or the unsubstituted analog. Moreover, it was concluded that optimal COX-2 suppression efficiency was achieved *via* the connection of the thiazole scaffold with a *p*-methoxy phenyl ring. The exchanging of *p*-methoxy with *p*-methyl/bromo are detrimental for the potency, whereas derivatives 100a, d, e presented a modest 15-LOX suppression efficacy (IC_50_ = 5.54, 5.29 and 5.68 µM, respectively) relative to meclofenamate (IC_50_ = 3.84 µM). It was deduced that the 15-LOX inhibitory efficiency was boosted by the engagement of the thiazole ring with a *p*-bromo phenyl ring more than the *p*-methyl/methoxy phenyl ring. In comparison to celecoxib (EI = 70%), the results highlighted the gradual advance and extended suppression effectiveness of edema for derivatives 100a–c at the 5th hour interval (EI = 72.30%, 81.74%, and 79.44%, respectively). Compared to celecoxib (UI = 3.02), derivatives 100a–c demonstrated better safety profiles, with ulcer indices of 2.6, 2.16 and 1.22, respectively. Consequently, these findings supported their clinical significance as prospective anti-inflammatory drugs. All studied derivatives disclosed a substantial decrease in the serum concentrations of PGE, TNF-α and IL-6 (PGE Serum conc. ranging from 498.25 to 527.98 pg mL^−1^; TNF-α Serum conc. ranging from 11.48 to 11.67 pg mL^−1^ and IL-6 Serum conc. ranging from 309.98 to 320.14 pg mL^−1^) as compared to celecoxib (546.89, 12.68 and 337.09 pg mL^−1^, respectively). The treatments with compounds 100c displayed the fewest histopathological alterations, suggesting a safe gastric profile. Moreover, the inspection of the kidney section displayed that the treatment with derivative 100c uncovered some tubular cells with darkly pigmented nuclei and a renal cortex with less dilated distal convoluted tubules, whereas renal glomerular fibrosis was not visible with the Sirius red stain. However, the derivative 100c-treated group's cardiac histopathological analysis revealed little to no toxic effects on the heart and revealed viable cardiac muscle fibers with distinct cell borders, average blood vessels, central oval nuclei and normal cardiac muscle fibers. Furthermore, in comparison to celecoxib, derivatives 100a–c attained competitive binding scores of −8.65, −8.92, and −9.08 kcal mol^−1^, respectively, within the COX-2 binding site. Derivative 100c participated in pi-sigma interactions with Val335, Ala513, Ser339 and Val509, in addition to the H-bond with Arg106. Moreover, pi-alkyl interactions were detected with Leu370, Val102, Phe504, Leu345, Tyr101 and Trp373 (ref. 88) (Fig. 24).

**Scheme 25:**
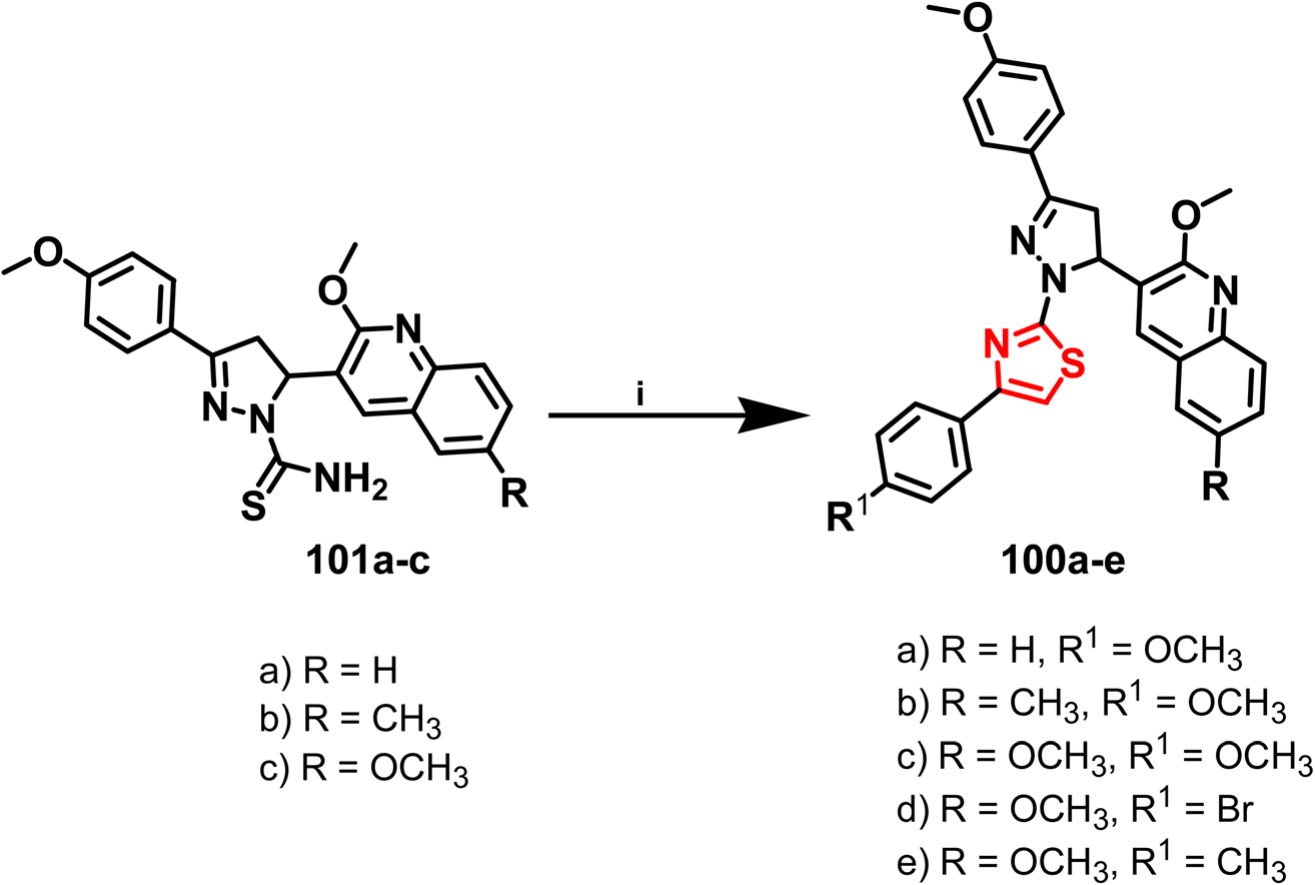
Synthesis of the quinoline-containing thiazolylpyrazole derivatives 100a–e. Reagents and conditions: (i) substituted phenacyl bromide, TEA, EtOH, reflux, 3 h.

The thioamide derivatives 101a–c underwent *S*-alkylation and condensation through a reaction with phenacyl bromide in the presence of a few drops of triethylamine, which resulted in additional cyclization, where the quinoline-containing thiazolylpyrazole derivatives 100a–e have been attained (Scheme 25).^88^

**Fig. 25:**
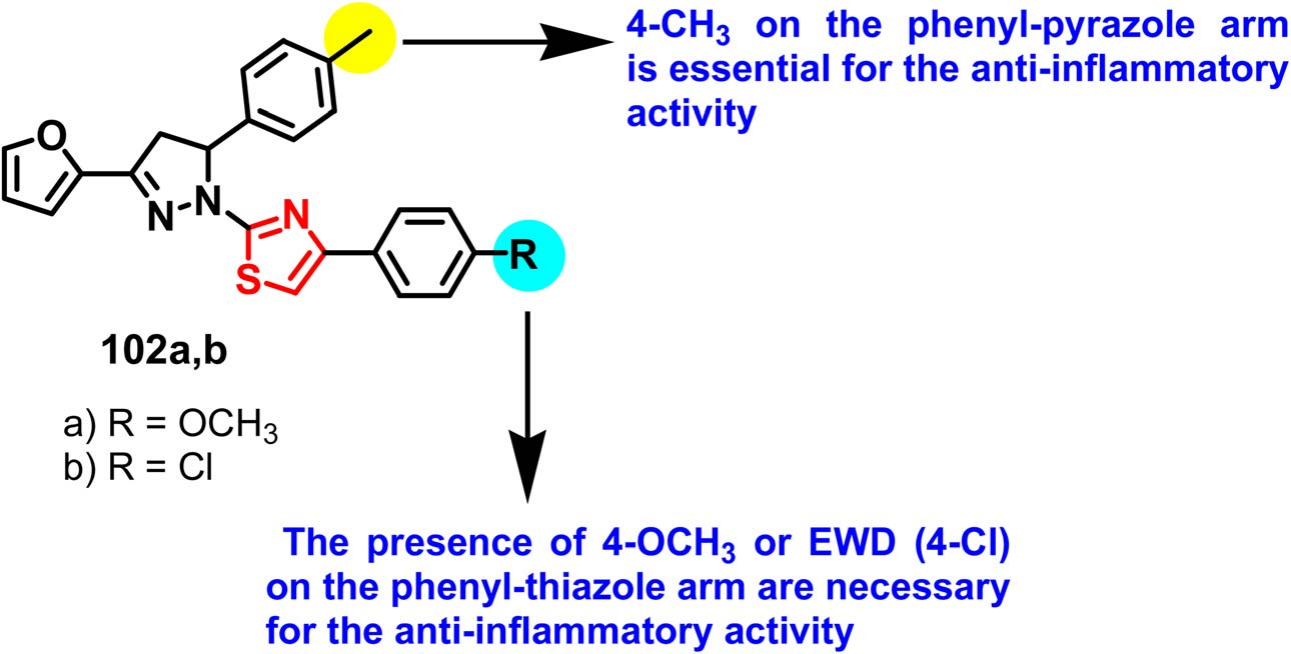
Structures of thiazole-pyrazole hybrids 102a, b as anti-inflammatory agents targeting the COX-2 enzyme.

Fadaly *et al.*^89^ illustrated the synthesis of new thiazole-pyrazole hybrids and assessed their COX-2 suppression impacts. Among the screened hybrids, derivative 102a, b demonstrated the optimal COX-2 inhibitory efficacy (IC_50_ = 0.772 and 0.686 µM, respectively) with promising selectivity indices (SI = 18.7 and 31.7, respectively), as compared to celecoxib (IC_50_ = 0.446 µM, SI = 10.3). Furthermore, the *in vivo* anti-inflammatory outcomes displayed that derivatives 102a, b (ED_50_ = 8.2 and 24 mg kg^−1^, respectively) were more efficient than celecoxib (ED_50_ = 30 mg kg^−1^). It was noticed that the appearance of EDG (4-CH_3_) on the phenyl-pyrazole arm and the presence of 4-OCH_3_ or EWD (4-Cl) on the phenyl-thiazole arm were the most essential moieties for the anti-inflammatory effectiveness. Furthermore, derivatives 102a, b presented excellent fitting inside the binding region of the COX-2 active site (docking scores = −15.76 and −15.70 kcal mol^−1^, respectively). Both derivatives 102a, b established two H-bonds between S of the thiazole ring and *N*_2_-pyrazole with Glu524 and Arg120 residues, respectively. An extra H-bond was displayed between the Cl atom of derivatives 102b and Gln192. Interestingly, derivatives 102a, b follow Lipinski's rule by having no H-bond donor, and 4 and 3H bond acceptors, respectively. In addition, the number of rotatable bonds of derivatives 102a, b are 5 and 4, respectively, whereas the TPSA values for derivatives 102a, b were 79.10 and 69.87 Å, respectively. Additionally, as suggested by Lipinski's rule of five, the anticipated values of *m* log *P* were determined to be less than 4.15. They possessed high GI absorption and good bioavailability score (0.55), but cannot cross the BBB. They were predicted to suppress both CYP2C9 and CYP2C19 enzymes, which indicates that they are less exposed to inhibitory drug metabolism. Otherwise, derivatives 102a, b are expected to be AMES-positive and so mutagenic. Moreover, derivatives 102a, b demonstrated maximum recommended tolerated human dose (MRTD) values of −0.39 and −0.048, respectively, which suggest the low-toxic-dose threshold of these derivatives. Both derivatives did not reveal hERG-I inhibitory effects; in addition, derivatives 102a, b displayed oral rat acute toxicity (LD_50_) values of 2.363 and 2.246 mol kg^−1^, respectively. Only derivative 102b is projected to be non-hepatotoxic and non-immunotoxic; otherwise, both derivatives 102a, b did not show skin sensitization. Furthermore, derivatives 102a, b were predicted to demonstrate no cardiotoxicity, cytotoxicity and nephrotoxicity (Fig. 25).

**Scheme 26:**
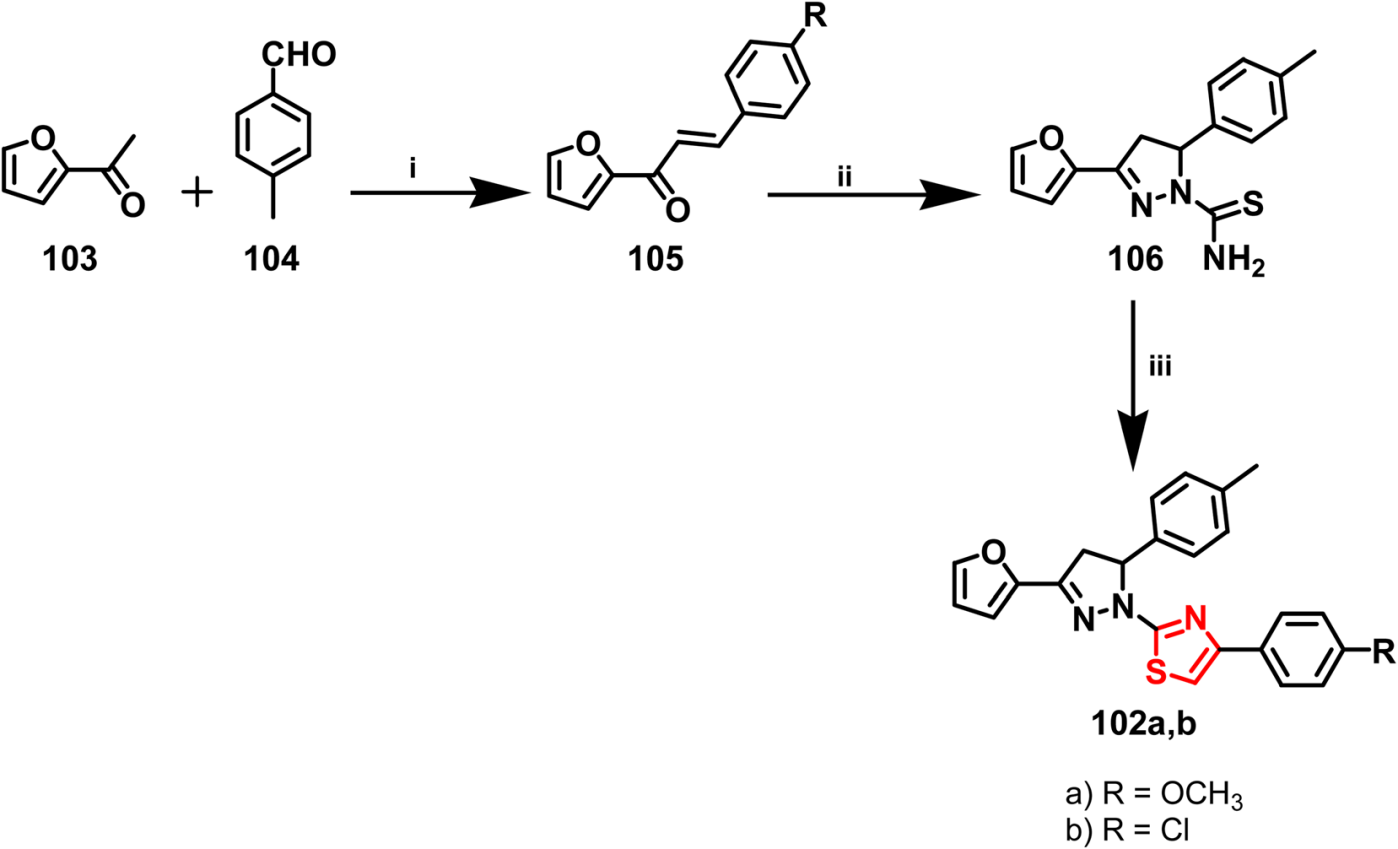
Synthesis of the thiazole-pyrazole hybrids 102a, b. Reagents and conditions: (i) ethanolic NaOH, stirring 24 h; (ii) NH_2_CSNHNH_2_, ethanolic NaOH, reflux 12 h; (iii) substituted phenacyl bromide, Na acetate, ethanol 95%, reflux 6 h.

The Claisen–Schmidt condensation reaction of 2-acetylfuran 103 with the 4-methylbenzaldehyde 104 afforded chalcone derivative 105. Cyclocondensation of the chalcone derivative 105 with thiosemicarbazide furnished the pyrazolyl-thioamide derivative 106. The target thiazole-pyrazole hybrids 102a, b were accomplished *via* the cycloaddition of 4-substitutedphenacylbromide to the pyrazolyl-thioamide derivative 106 (Scheme 26).^89^

### 2.5 Thiazole-imidazole hybrids as anti-inflammatory agents

**Fig. 26:**
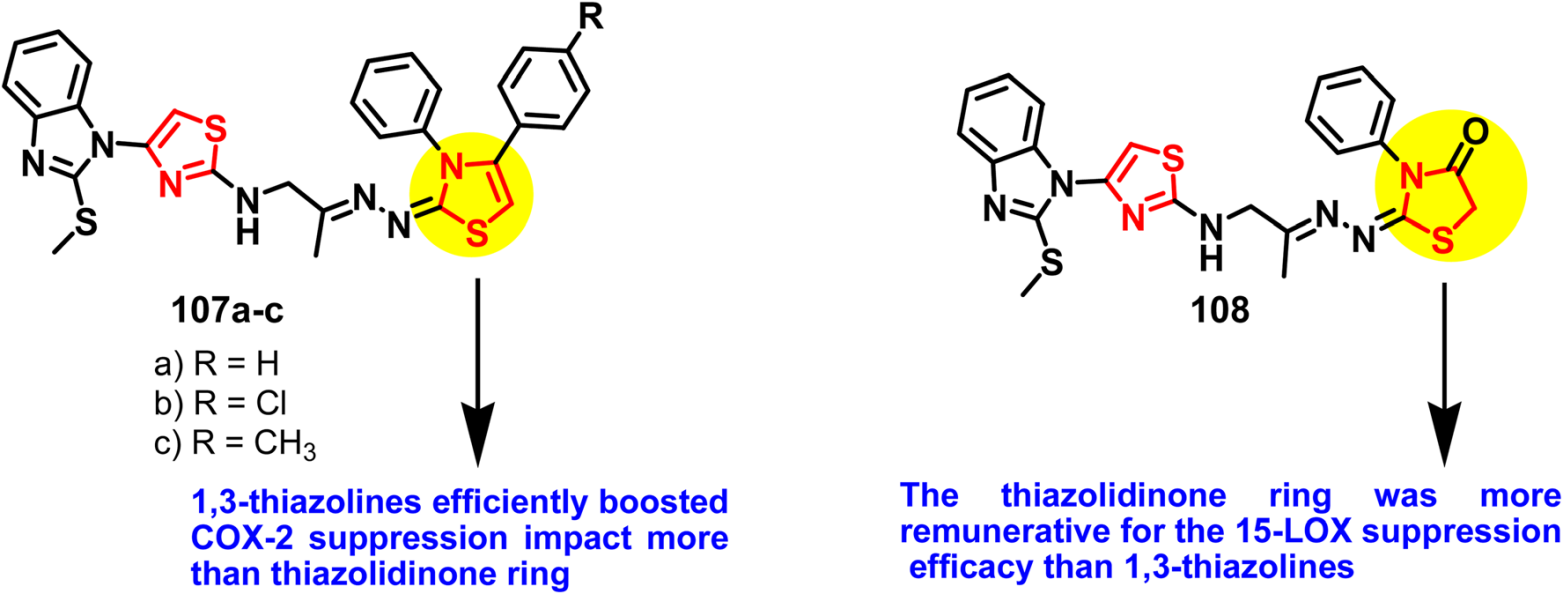
Structures of thiazole-benzimidazole hybrids 107a–c and 108 as anti-inflammatory agents targeting dual COX-2 and 15-LOX enzymes.

New thiazole-benzimidazole hybrids 107a–c and 108 were uncovered as notable COX-2 suppressors (IC_50_ = 0.058, 0.045, 0.054 and 0.067 µM, respectively) with remarkable COX-2 selectivity (SI = 142–293.78). Intriguingly, the hybrids 107a–c and 108 unveiled eminent 15-LOX suppression effectiveness (IC_50_ = 1.85, 1.67, 2.14, and 1.96 µM, respectively). It became apparent that the connection of 1,3-thiazolines to the benzimidazole-thiazole hybrids efficiently boosted COX-suppression impact more than the thiazolidinone ring. Conversely, the combination of the benzimidazole-thiazole hybrids with the thiazolidinone ring was more remunerative for the 15-LOX suppression efficacy than 1,3-thiazolines. The efficacious hybrids (107b and 108) were elected for *in vivo* assessment by means of a carrageenan-stimulated paw edema assay. At both 3- and 4-hours intervals, the benzimidazole-thiazole hybrid associated with 4-thiazolidinone 108 demonstrated the greatest edema inhibition values of 82.83% and 80.57%, respectively, in comparison to indomethacin (69.37% and 78.83%, respectively). Even more, rat stomach mucosa seemed normal after treatment with the derivative 108, with intact glands and surface epithelium; however, there was considerable blood vessel congestion. Thus, derivative 108 highlighted an improved gastrointestinal safety profile. Moreover, a strong binding affinity (−7.40 kcal mol^−1^) for the COX-2 active site is shown by derivative 108. At the hydrophobic pocket of the COX-2 active site, hydrophobic interactions with varied amino acids were detected. In addition, Ser339 and Asp501 participated in three H-bonds with NH, N and S, respectively. Conversely, strong binding affinity (−9.40 kcal mol^−1^) into the 15-LOX active site was shown by hybrid 108. It created numerous hydrophobic interactions with His80, Val509, Leu338, Ala513, His342, His75, Val335, Gly340, Thr79, Pro500 and Phe504, whereas NH and N are involved in H-bonds with Ser339. Hence, the benzimidazole-thiazole is a viable building block for creating novel anti-inflammatory candidates that have dual suppression effects on the COX-2 and 15-LOX enzymes^90^ (Fig. 26).

**Scheme 27:**
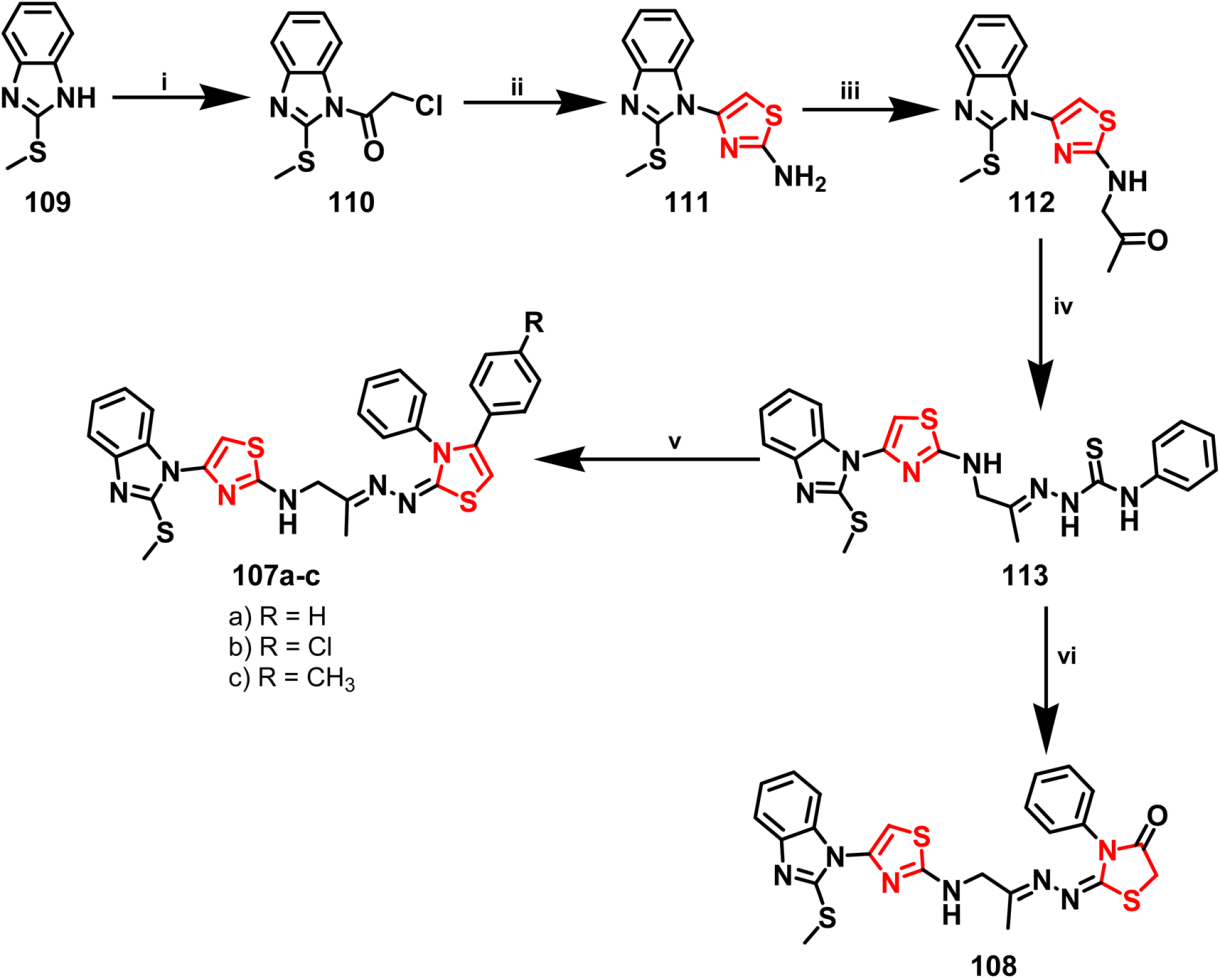
Synthesis of the thiazole-benzimidazole hybrids 107a–c and 108. Reagents and conditions: (i) chloroacetyl chloride, NaH,DMF, 0 °C, 9 h; (ii) thiourea, ethanol, reflux, 6 h then NH_4_OH; (iii) chloroacetone, K_2_CO_3_, acetone, RT, 2 h then reflux 7 h; (iv) phenyl thiosemicarbazide, few drops of glacial acetic acid, ethanol, reflux, 10 h; (v) *p*-(un)substituted phenacyl bromide, ethanol, reflux 20–24 h then neutralizationwith sodium acetate; (vi) methyl bromo acetate, ethanol, reflux, 12 h then neutralization with sodiumacetate.

The target thiazole-benzimidazole hybrids 107a–c and 108 were achieved as depicted in Scheme 27.^90^

**Fig. 27:**
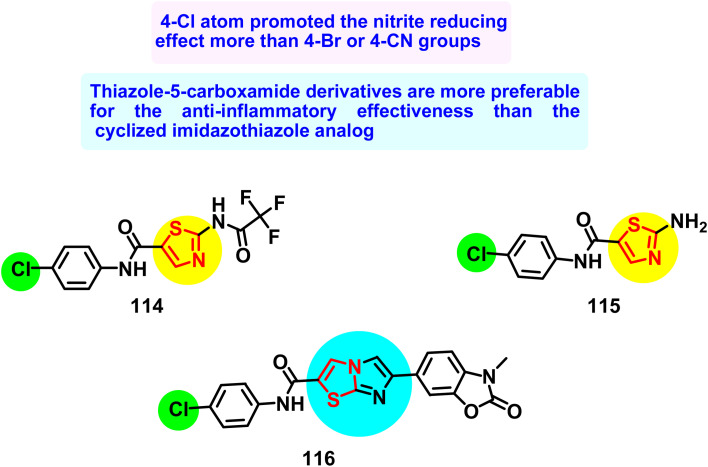
Structures of thiazole-5-carboxamide derivatives 114 and 115 and imidazothiazole-bearing carboxamide derivative 116 as anti-inflammatory agents targeting a nitrite inflammatory mediator.

Can *et al.*^91^ discussed the synthesis and the anti-inflammatory investigation of new imidazothiazole-bearing carboxamide or methyl carboxylate moieties. Derivative 114 demonstrated weak nitrite reducing effects inhibition = 19.41%) relative to indomethacin (inhibition = 47.98%). However, the precursor thiazole-5-carboxamide derivatives 115 and 116 presented prominent nitrite reducing impacts (inhibition = 39.07% and 40.86%, respectively) similar to that of indomethacin. It was inferred that the appearance of 4-Cl atom on the phenyl ring linked to the carboxamide moiety promoted the nitrite reducing effect more than 4-Br or 4-CN groups. Furthermore, the thiazole-5-carboxamide derivatives are more preferable for the anti-inflammatory effectiveness than the cyclized imidazothiazole analog (Fig. 27).

**Scheme 28:**
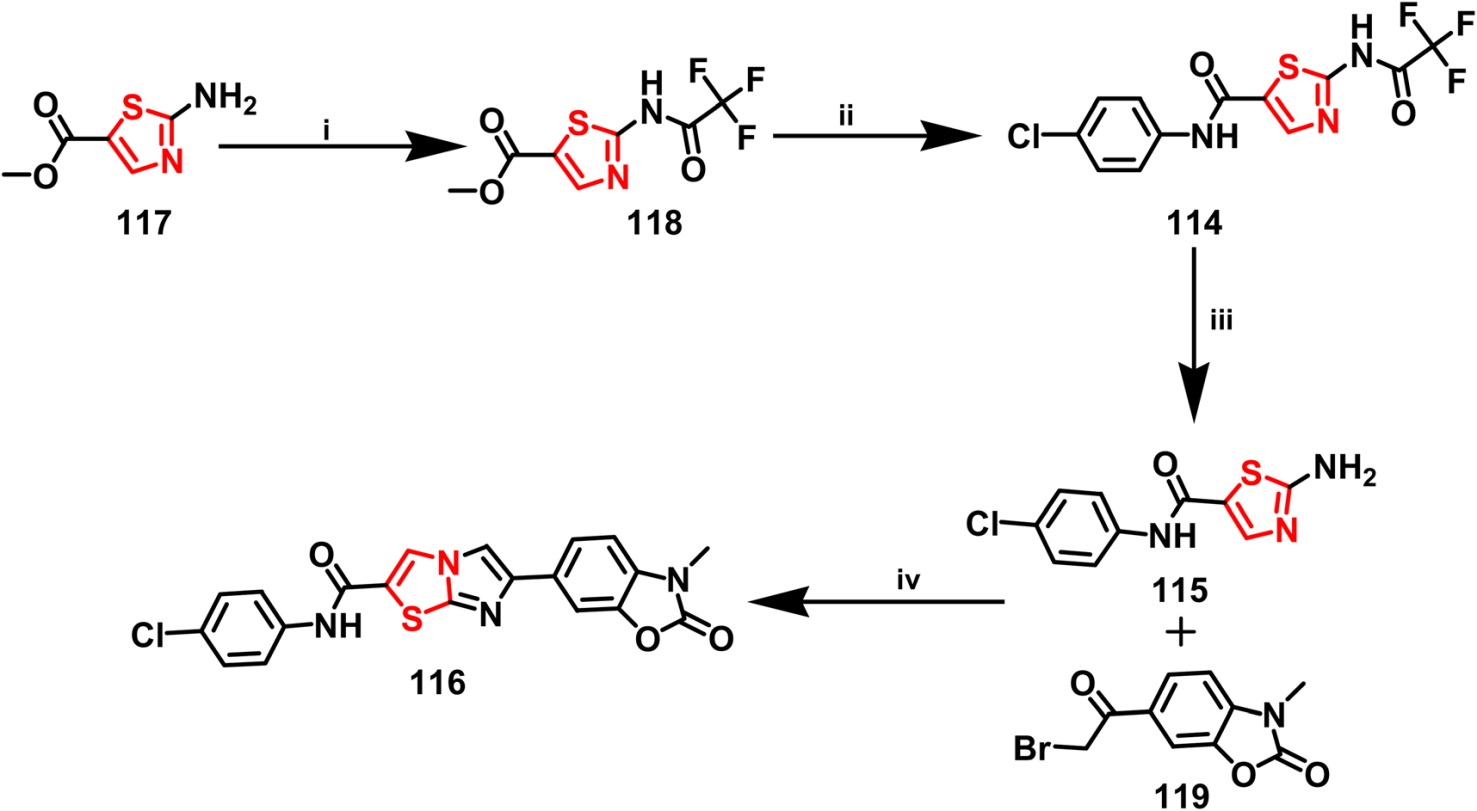
Synthesis of the thiazole-5-carboxamide derivatives 114 and 115 and imidazothiazole-bearing carboxamide derivative 116. Reagents and conditions: (i) (CF_3_CO)_2_O,CH_2_Cl_2_, pyridine, RT; (ii) 4-chloro aniline, potassium *tert*-butoxide, DMF, RT; (iii) K_2_CO_3_, MeOH/H_2_O, RT; (iv) *n*-BuOH, reflux.

The reaction of methyl 2-amino-1,3-thiazole-5-carboxylate 117 with trifluoroacetic anhydride furnished the trifluoroacetamide derivative 118. The latter analog 118 underwent a reaction with *p*-chloro aniline to afford a new trifluoroacetamide-thiazole carboxamide derivative 114. The acetyl deprotection reaction of derivative 114 yielded 2-amino-*N*-(4-(chlorophenyl)-1,3-thiazole-5-carboxamide 115, which upon cyclization with 6-bromoacetyl-3-methyl-2-oxo-3*H*-benzoxazole 119 furnished the imidazothiazole-bearing carboxamide derivative 116 (Scheme 28).^91^

**Fig. 28:**
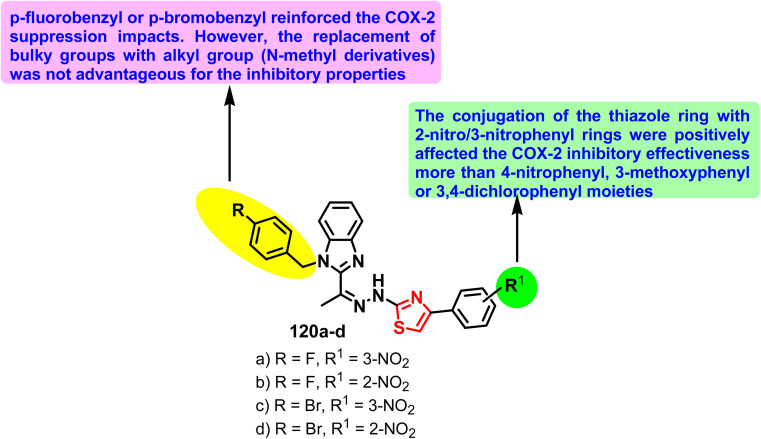
Structures of benzimidazole-thiazole hybrids 120a–d as COX-2 inhibitors.

In 2023, benzimidazole-thiazole hybrids 120a–d stood out as notable COX-2 inhibitors (IC_50_ = 0.297, 0.311, 0.279 and 0.215 µM, respectively), comparable to that of celecoxib (IC_50_ = 0.132 µM). It was noted that the introduction of *p*-fluorobenzyl or *p*-bromobenzyl at the 1st position of benzimidazole reinforced the COX-2 suppression impacts. However, the replacement of bulky groups with alkyl groups (*N*-methyl derivatives) at the 1st position of benzimidazole was not advantageous for the inhibitory properties. Furthermore, the conjugation of the thiazole ring with 2-nitro/3-nitrophenyl rings positively affected the COX-2 inhibitory effectiveness more than 4-nitrophenyl, 3-methoxyphenyl or 3,4-dichlorophenyl moieties. In addition, derivatives 120a–d are properly positioned inside the COX-2 binding region and revealed promising docking scores ranging from −8.485 to −8.927 kcal mol^−1^. Derivatives 120a–d established H-bonds with Tyr355 and Arg120. Additionally, Tyr385 was engaged in halogen bonds for derivatives 120a–c. Furthermore, several hydrophobic interactions were detected with Leu352, Val349, Arg120, Tyr115, Ala527, Val116 and Val523 residues, for the derivative 120a, c. Otherwise, derivatives 120b, d interacted hydrophobically with Ala527, Phe518, Leu352, Tyr115, Val523, Val349, Val116 and Val89 residues. These interactions play a major role in the high binding affinity of derivatives 120a–d, which boosting its ability to suppress the COX-2 and highlight the effective role of derivatives 120a–d as prominent COX-2 inhibitors^92^ (Fig. 28).

**Scheme 29:**
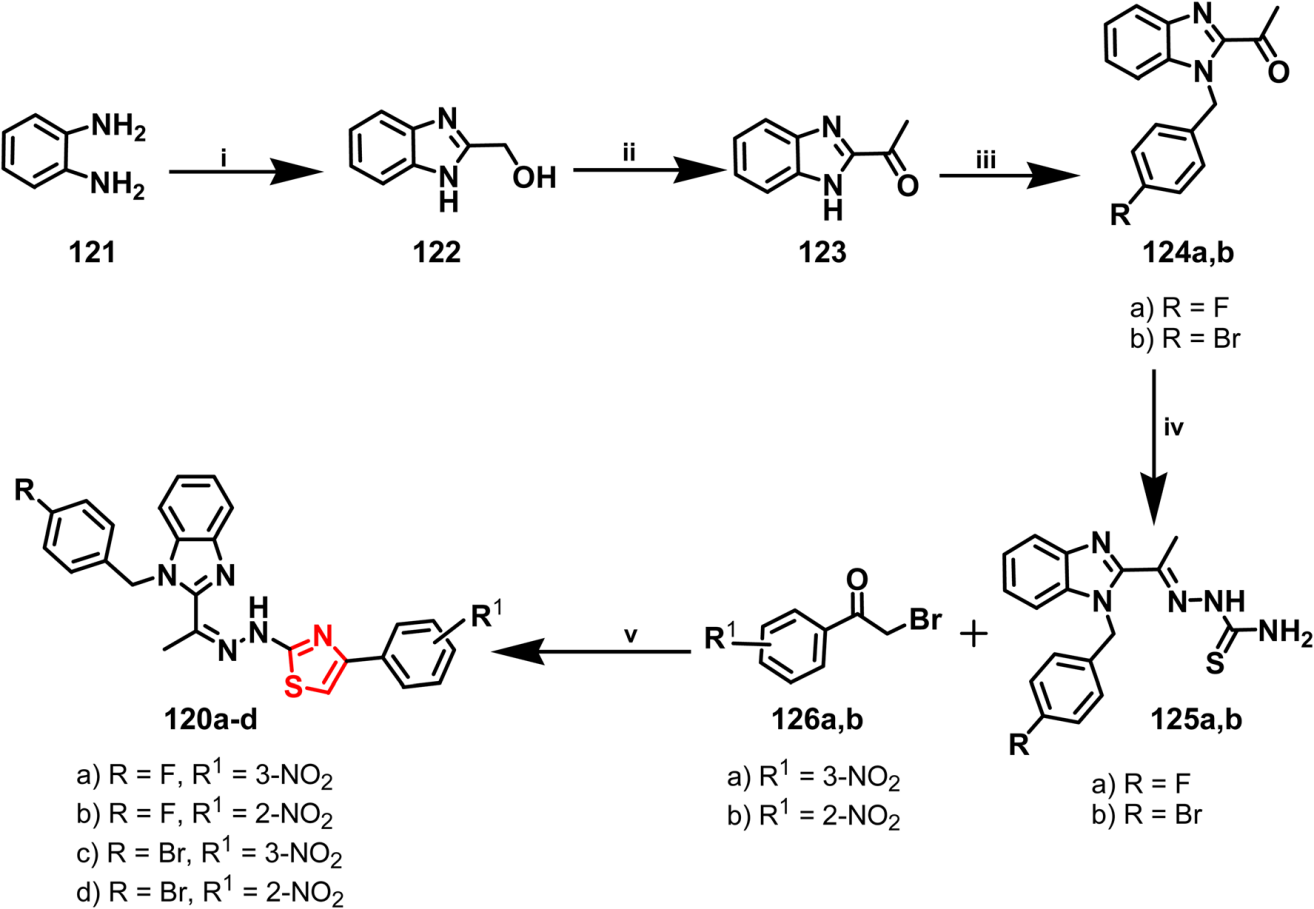
Synthesis of the benzimidazole-thiazole hybrids 120a–d. Reagents and conditions: (i) lactic acid, reflux, 3 h; (ii) 5% w/v H_2_SO_4_, K_2_Cr_2_O_7_, stirring, RT, 2 h; (iii) *p*-fluorobenzyl bromide/*p*-bromobenzylbromide, acetone, anhdr K_2_CO_3_; (iv) H_2_NNHCSNH_2_, 5% w/v *p*-TsOH EtOH, reflux, 8 h; (v) dioxane, reflux.

At first, the benzimidazole ring 122 was cyclized by reacting *o*-phenylenediamine 121 with lactic acid. The 2-acetylbenzimidazole 123 is produced when 1-hydroxy ethyl 122 is oxidized. Next, *p*-fluoro, and *p*-bromo benzyl were used to derivatize the benzimidazole ring 123 from the first position to afford derivatives 124a, b. Thiosemicarbazone derivatives 125a, b were created *via* the reaction of derivative 124a, b with thiosemicarbazide. Finally, benzimidazole-thiazole hybrids 120a–d were acquired *via* treating the thiosemicarbazone derivatives 125a, b with substituted phenacyl bromide 126a, b (Scheme 29).^92^

**Fig. 29:**
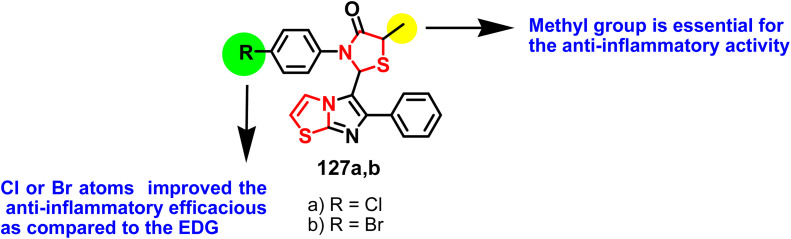
Structures of imidazothiazole derivatives containing thiazolidinone ring 127a, b as anti-inflammatory agents.

Kamboj *et al.*^93^ developed and assessed novel imidazothiazole derivatives containing a thiazolidinone ring for their anti-inflammatory effectiveness. Derivatives 127a, b disclosed the highest edema inhibition percentages among the investigated candidates, with respective values of 81.34% and 80.17%. Additionally, compounds 127a, b showed denaturation inhibition of 84.94% and 83.64%, respectively. They also showed a notable decline in the severity index (SI 0.416 and 0.5, respectively), indicating a diminished capacity to cause ulcers. Moreover, lipid peroxidation was significantly reduced by the investigated candidates 127a, b with values of 4.12 and 5.37 nmol MDA/100 mg tissue. As a result, it was determined that lipid peroxidation inhibition may be linked to the protection of the stomach mucosa. Derivatives 127a, b have a methyl group at the fifth position of the thiazolidinone molecule and are shown to be more effective anti-inflammatory candidates than derivatives lacking a methyl group. In addition, the presence of halogen especially Cl or Br atoms at the para position of the phenyl ring connected to the thiazolidinone scaffold improved the anti-inflammatory efficacious as compared to the EDG (Fig. 29).

**Scheme 30:**
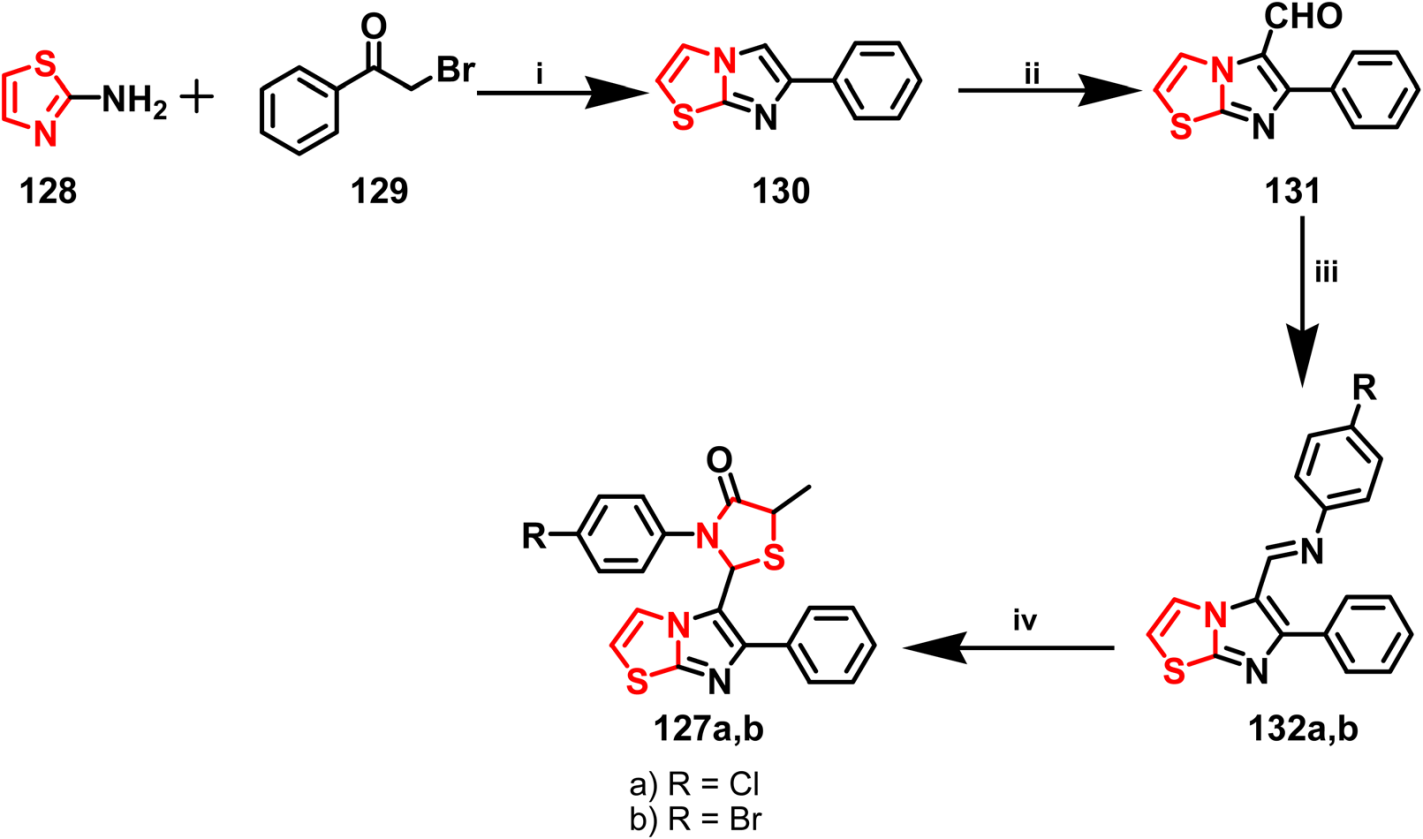
Synthesis of the imidazothiazole derivatives containing thiazolidinone ring 127a, b. Reagent and conditions. (i) Methanol, 70 °C, reflux; (ii) CHCl_3_, DMF, POCl_3_, 120 °C, reflux; (iii) substituted anilines, Toluene, PTSA, 120 °C; (iv) thiolactic acid, dioxane, anhydrous zinc chloride, RT, 16–20 h.

Refluxing aminothiazole 128 and phenacyl bromide 129 afforded the imidazothiazole derivative 130, which further reacted *via* the Vilsmeier–Haack reaction to get the imidazothiazole carbaldehyde 131. Moreover, the treatment of imidazothiazole carbaldehyde 131 with diverse substituted anilines furnished Schiff bases 132a, b. Using anhydrous zinc chloride as a catalyst, the intermediates 132a, b and thiolactic acid were stirred to acquire the target derivatives 127a, b (Scheme 30).^93^

**Fig. 30:**
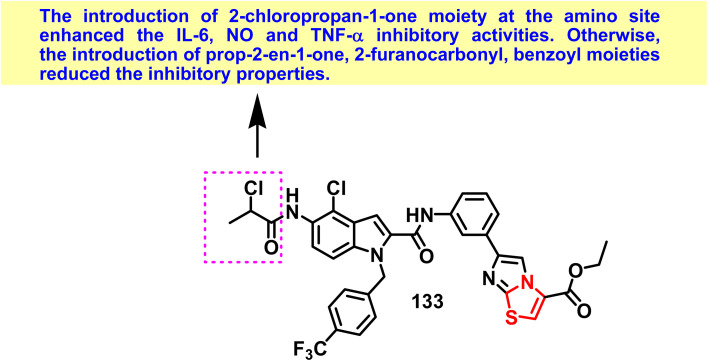
Structure of indole-2-formamide benzimidazole[2,1-*b*]thiazole derivative 133 as an anti-inflammatory agent targeting various inflammatory mediators.

A new set of indole-2-formamide benzimidazole[2,1-*b*]thiazole derivatives were obtained by using the molecular hybridization approach of the indole ring and imidazole[2,1-*b*]thiazole. Additionally, the inhibitory effects of the investigated candidates on the LPS-induced production of pro-inflammatory cytokines such as IL-6, NO, and TNF-α in RAW264.7 cells were assessed. Derivative 133 demonstrated the strongest inhibition of IL-6, NO and TNF-α with IC_50_ values of 2.29, 10.99 and 12.90 µM, respectively. Furthermore, derivative 133 significantly elevated Fe^2+^ levels, reactive oxygen species and malondialdehyde levels relative to the control group, while it reduced the glutathione content as compared to the control group, thus facilitating the iron death process. Moreover, it does not exert any toxic effects on RAW264.7 cells. SAR analysis detected that the introduction of a 2-chloropropan-1-one moiety at the amino site enhanced the IL-6, NO and TNF-α inhibitory activities. Otherwise, the introduction of prop-2-en-1-one, 2-furanocarbonyl, and benzoyl moieties reduced the inhibitory properties^94^ (Fig. 30).

**Scheme 31:**
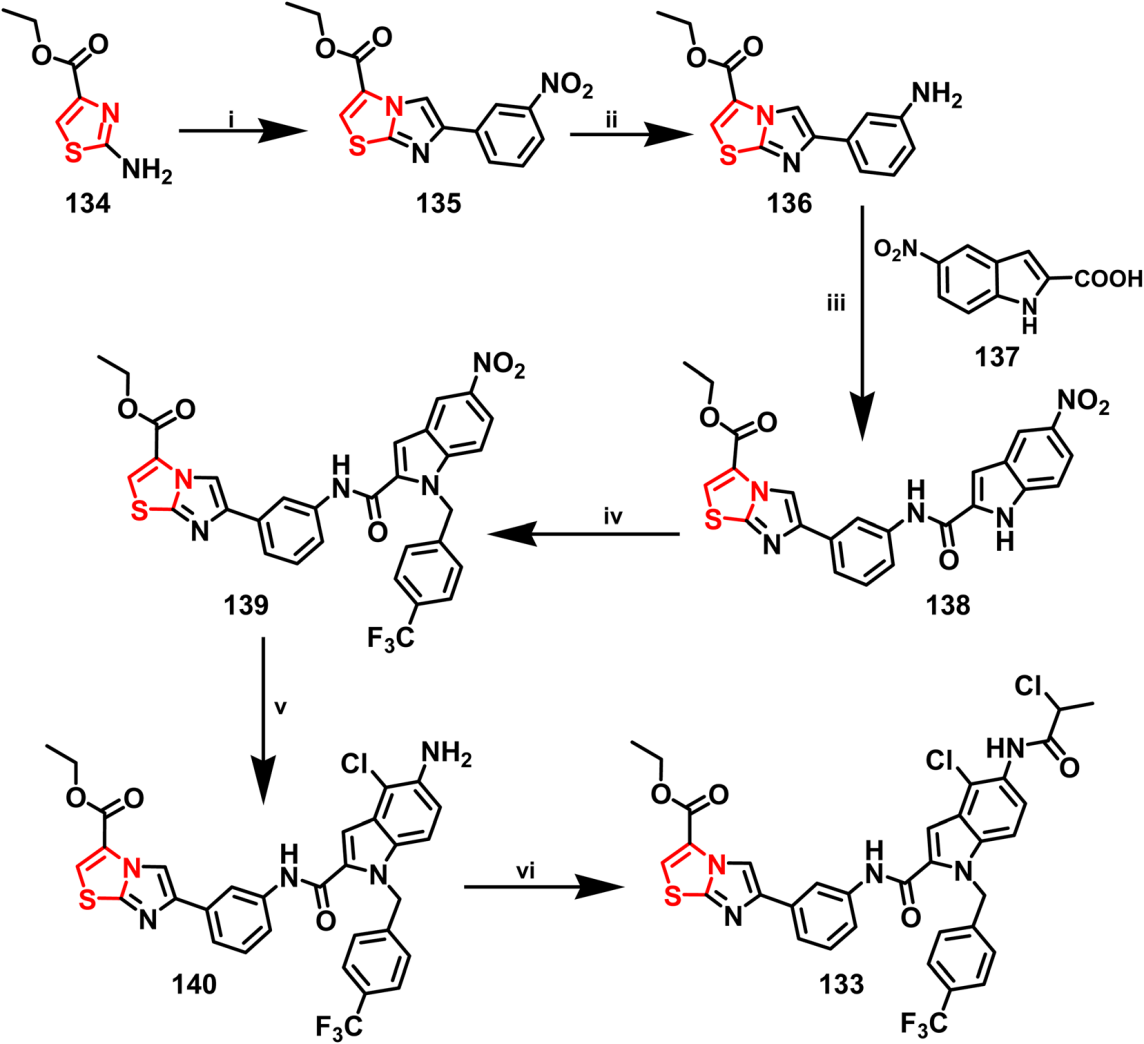
Synthesis of the indole-2-formamide benzimidazole[2,1-*b*]thiazole derivative 133. Reagents and conditions: (i) 3-nitro phenacyl bromide, 1,4-dioxane, reflux, 110 °C, 12 h; (ii) SnCl_2_, 65 °C, 10 min; (iii) DMA, TBTU, DIPEA, rt; (iv) CH_3_CN, K_2_CO_3_, 50 °C, 5 h; (v) SnCl_2_, HCl, 3 h, rt; (vi) 2-chloropropanoyl chloride, DMA, DIPEA, rt.

Refluxing ethyl 2-aminothiazole-4-carboxylate 134 and 3-nitro phenacyl bromide yielded derivative 135. Then, compound 135 underwent a reduction reaction to afford the amino derivative 136. Subsequently, compound 136 was reacted with 5-nitro-1H-indole-2-carboxylic acid 137 to get derivative 138, which was followed by condensation with 4-trifluoromethyl benzyl bromide to obtain derivative 139. Furthermore, derivative 139 was subjected to nitro reduction and halogen introduction to afford derivative 140. Finally, the target product 133 was accomplished *via* the reaction of derivative 140 with 2-chloropropanoyl chloride (Scheme 31).^94^

### 2.6 Thiazole-triazole hybrids as anti-inflammatory agents

**Fig. 31:**
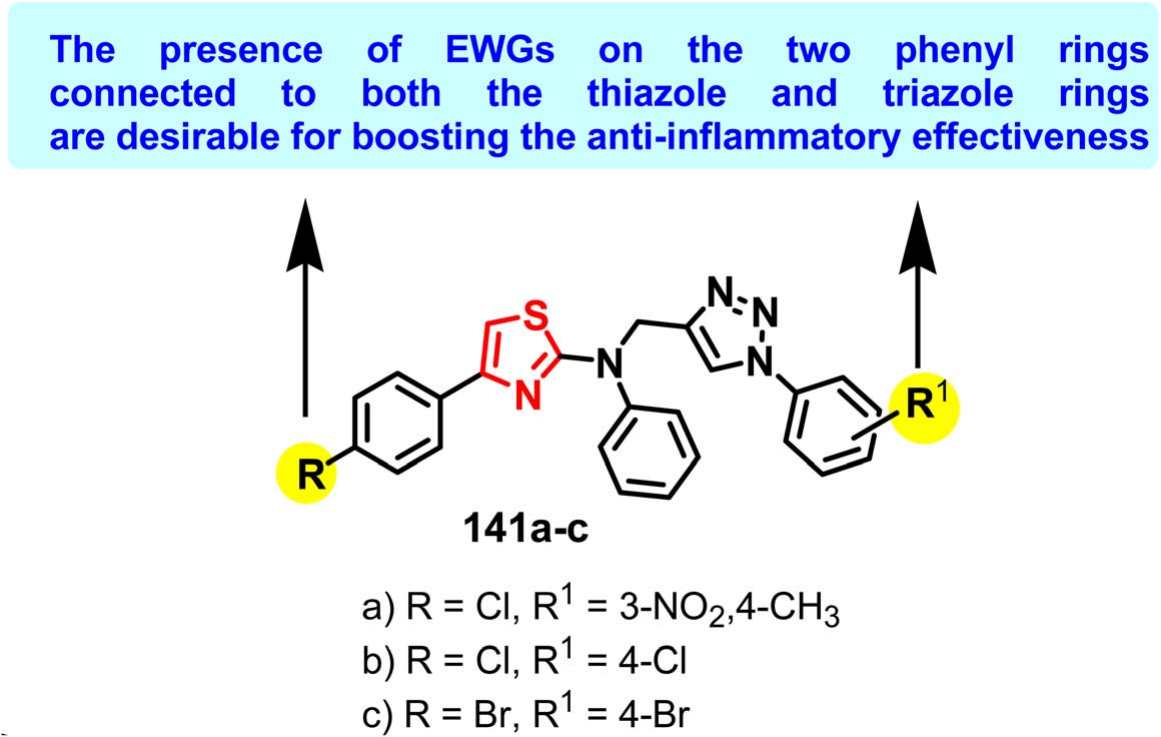
Structures of thiazole-triazole hybrids 141a–c as anti-inflammatory agents.

Ankali *et al.*^95^ explained the synthesis and determination of the anti-inflammatory efficacy of novel thiazole-triazole hybrids. Amongst the investigated candidates, derivatives 141a–c possessed the optimal *in vivo* anti-inflammatory impacts with edema inhibition of 57.07%, 61.21% and 58.55%, respectively, preferable than diclofenac (edema inhibition = 48.29%). It was elucidated that the presence of EWGs on the two phenyl rings connected to both the thiazole and triazole rings is desirable for boosting the anti-inflammatory effectiveness. Moreover, the LD 50 was determined to be more than 2000 mg kg^−1^, clarifying their safety profiles (Fig. 31).

**Scheme 32:**
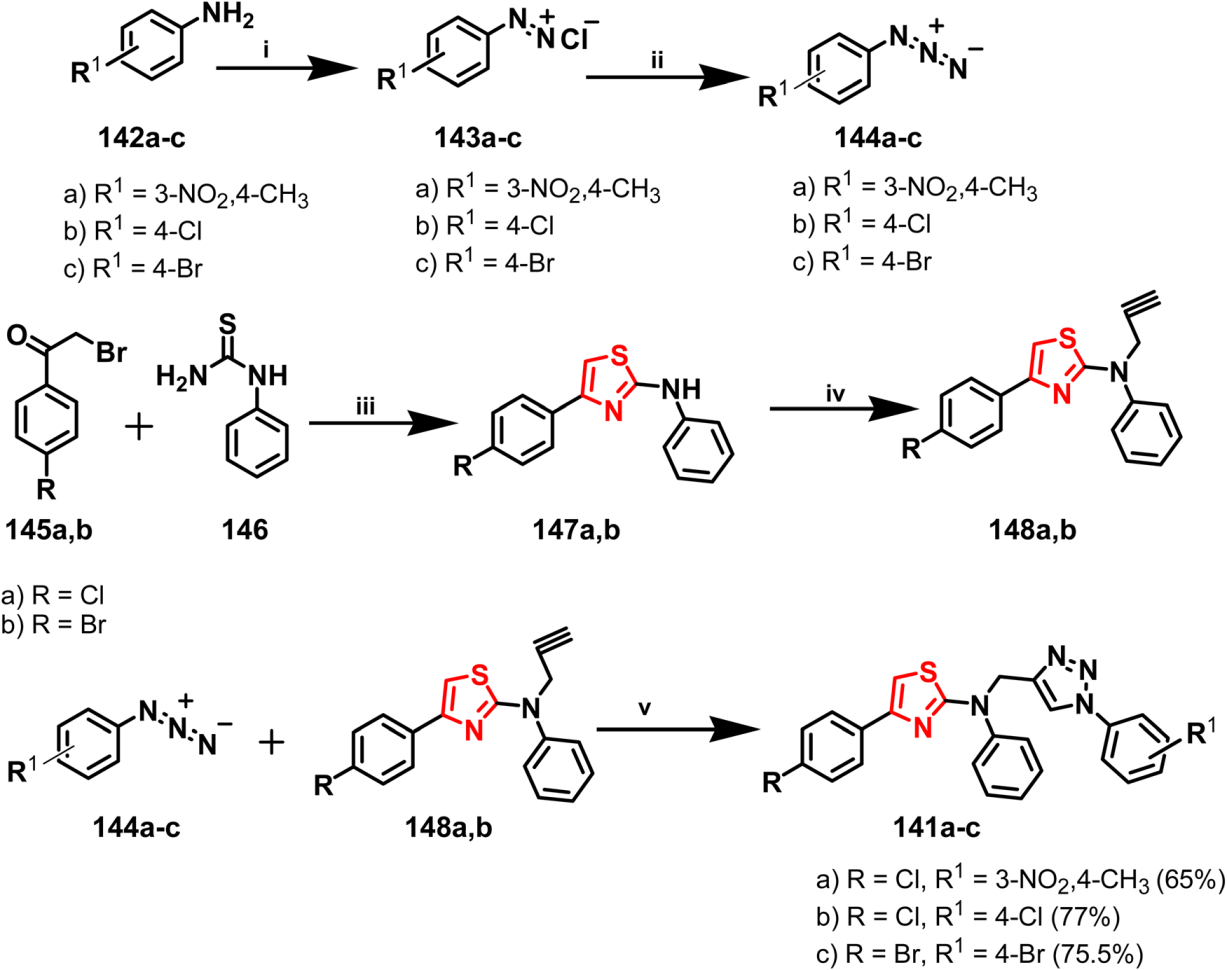
Synthesis of the thiazole-triazole hybrids 141a–c. Reagents and conditions: (i) NaNO_2_, Conc. HCl, 0–5 °C, 15 min; (ii) Aq. NaN_3_; (iii) K_2_CO_3_, DMF; (iv) propargyl bromide, K_2_CO_3_, DMF, N_2_ atmosphere, 60 °C, 24 h; (v) AcCN/H_2_O = 2 : 1, 1 M CuSO_4_·6H_2_O solution, 1 M sodium ascorbate, 60 °C, 8 h.

The diazotization of substituted aniline 142a–c afforded the aryl diazonium salt 143a–c, which upon reaction with sodium azide yielded the azidobenzene derivatives 144a–c. The intermediate derivative 147a, b was accomplished by the reaction of substituted phenacyl bromide 145a, b and 1-phenyl thiourea 146. Moreover, the key intermediates 148a, b were acquired by a base-stimulated reaction between the intermediate derivatives 147a, b with propargyl bromide. Finally, the thiazole-triazole hybrids 141a–c were furnished by the reaction of the key intermediates 148a, b and azidobenzene derivatives 144a–c (Scheme 32).^95^

**Fig. 32:**
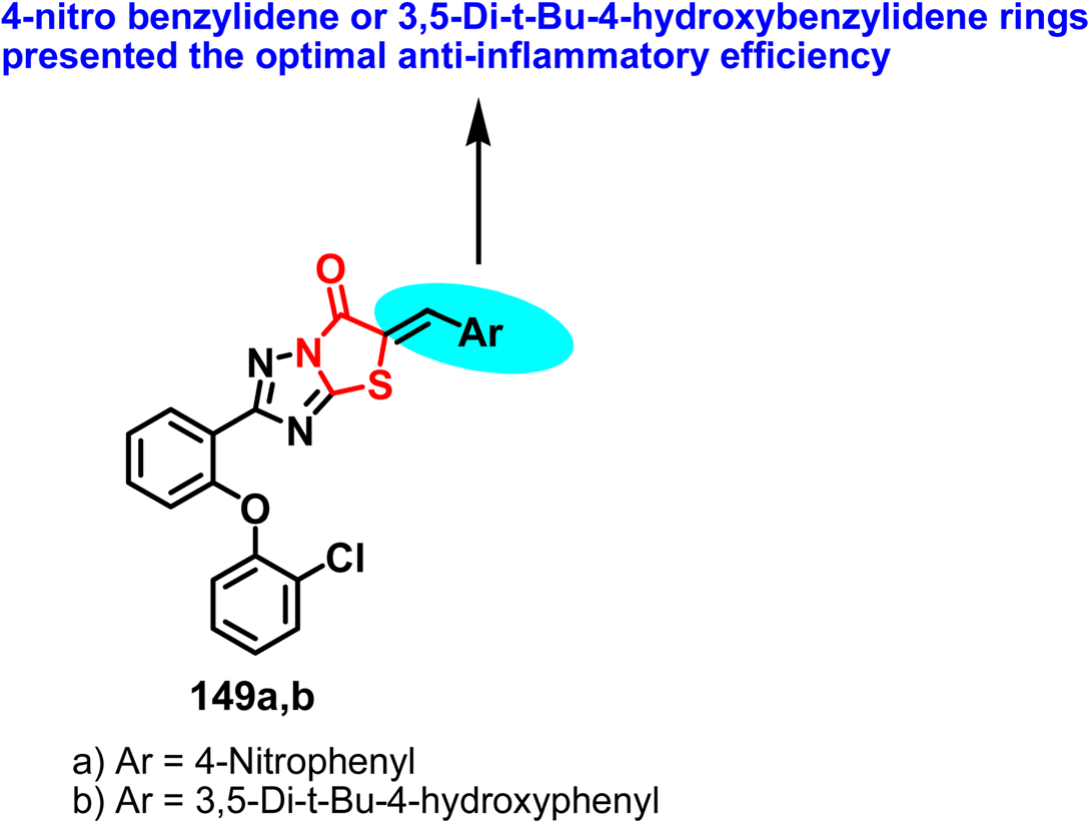
Structures of thiazolotriazolone derivatives 149a, b as anti-inflammatory agents targeting the COX-2 enzyme.

Almasirad *et al.*^96^ described the synthesis and the anti-inflammatory screening of new thiazolotriazolone derivatives. Within the screened candidates, derivatives 149a, b disclosed remarkable anti-inflammatory efficacy (edema inhibition = 53.1% and 49.3%, respectively) 4 h post administration with respect to mefenamic acid (edema inhibition = 42.30%). The biological outcomes unveiled that the connection of the thiazolotriazolone scaffold with 4-nitro benzylidene or 3,5-Di-*t*-Bu-4-hydroxybenzylidene rings presented the optimal anti-inflammatory efficiency. Moreover, derivatives 149a, b demonstrated low ulcerogenic risks with scores of 2.83 and 2.00, respectively, as compared to indomethacin, which displayed a high score of 11. Additionally, the most prominent anti-inflammatory derivatives 149a, b displayed the lowest docking energy of −11.95 and-13.10 kcalmol^−1^ over COX-2. The S atom of thiazolo[3,2-*b*][1, 2,4]triazole in both derivatives 149a, b interacted hydrophobically with Leu532. Furthermore, derivatives 149a, b demonstrated π–π stacking interaction between Tyr356 and the triazole ring. Ser531 and Arg120 contributed to two H-bonds with OH and C=O groups, respectively, for derivative 149b. In addition, π-cation interaction and an aromatic H-bond were detected between the phenoxy group Arg120 and Asn87, respectively, for derivative 149b. Derivative 149a did not display Lipinski violation, which shows a high likelihood of finding the drug-like potential, indicating a significant probability of discovering the drug-like potential. All of the parameters were found to be within the acceptable range for these derivatives' oral bioavailability, with the exception of the water solubility (log *S*_wat_). Reducing the size of 3,5-di-*t*-but substitution into a lighter alkyl side chain may increase the water solubility of derivative 149b, which may have some water solubility issues (log *S*_wat_ = −9.43). Because of the polar nitro group, derivative 149a (log *S*_wat_ = −6.45) is in better condition than derivative 149b. Additionally, derivative 149a may be a suitable option for oral absorption if efforts are made to boost its caco-2-cell permeability (Fig. 32).

**Scheme 33:**
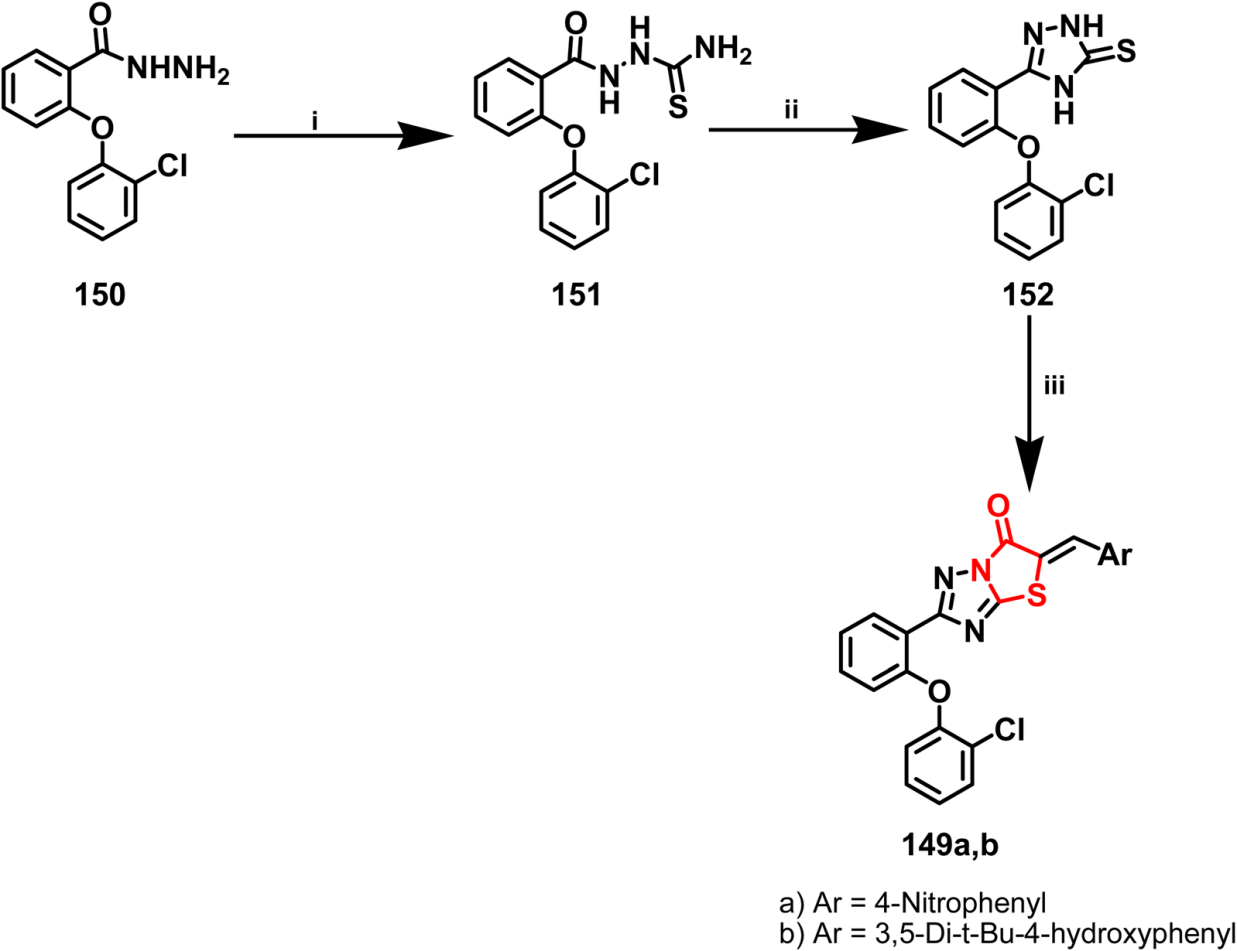
Synthesis of the thiazolotriazolone derivatives 149a, b. Reagents and conditions: (i) water, KSCN, HCl, reflux, 18 h; (ii) 4% NaOH, reflux, 8 h; (iii) ClCH_2_COOH, anhydrous sodium acetate, substituted aryl aldehydes, AcOH, Ac_2_O, reflux, 18 h.

The treatment of the hydrazide derivative 150 with potassium thiocyanate afforded the thiosemicarbazide derivative 151, which then refluxed in 4% NaOH to give triazole-3-thione derivative 152. The one pot reaction of the latter derivative 152 with chloroacetic acid, anhydrous sodium acetate and substituted aryl aldehydes furnished the thiazolotriazolone derivatives 149a, b (Scheme 33).^96^

### 2.7 Thiazole-pyridine hybrids as anti-inflammatory agents

**Fig. 33:**
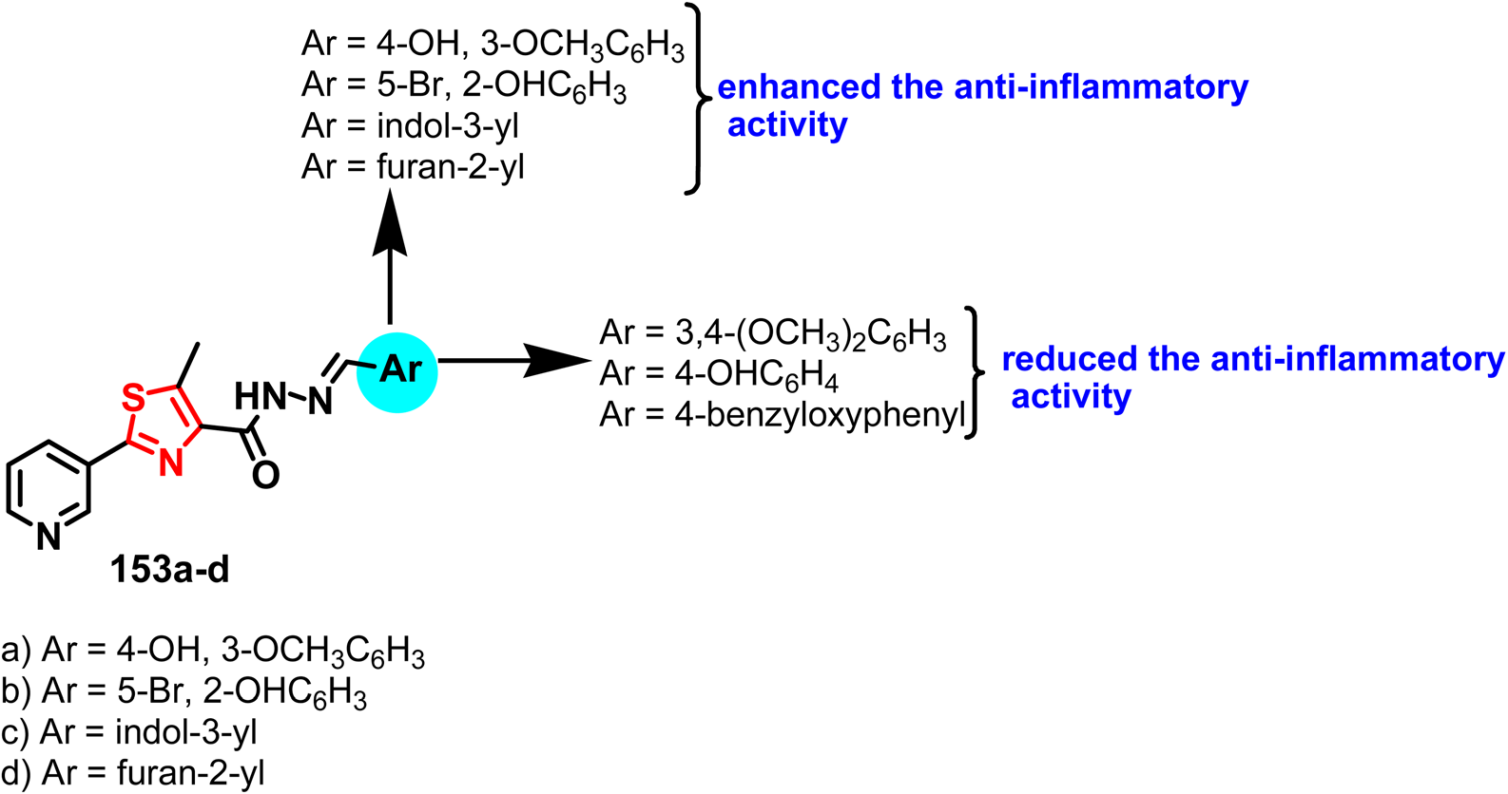
Structures of thiazole-pyridine hybrids 153a–d as anti-inflammatory agents targeting the COX-2 enzyme.

Kamat *et al.*^97^ illustrated the synthesis of new thiazole-pyridine hybrids and investigated their *in vitro* anti-inflammatory efficiency using denaturation of the bovine serum albumin method. Among the assessed derivatives, compound 153a demonstrated the highest inhibitory effect with a remarkable IC_50_ value of 46.29 µg mL^−1^, relative to that of diclofenac sodium (IC_50_ = 35.03 µg mL^−1^). Moreover, derivatives 153b–d demonstrated promising anti-inflammatory potencies with IC_50_ values of 55.58, 52.89 and 58.62 µg mL^−1^, respectively. SAR analysis disclosed that the nature of the substituents on the phenyl ring affected the anti-inflammatory effectiveness. It was noticed that the presence of the hydroxyl and methoxy substituents at the 4th and the 3rd positions, respectively, promoted the anti-inflammatory potency more than the presence of 3,4-dimethoxy substituents or the 4-hydroxy group. In addition, the presence of 5-bromo-2-hydroxy substituents was favorable for the anti-inflammatory effect. Furthermore, the replacement of the phenyl ring with heterocyclic rings such asindol-3-yl or furanyl-2-yl positively affected the anti-inflammatory efficacy. Otherwise, the lowest anti-inflammatory activity was observed for the derivative bearing the 4-benzyloxyphenyl group. The docking study of derivative 153b within the active site of COX-2 displayed one aromatic bond interaction between the pyridine moiety and the Ser516 residue, which also revealed a docking score of −9.80 kcal mol^−1^. The physicochemical parameters of the tested derivatives were found suitable in the reference range (Fig. 33).

**Scheme 34:**
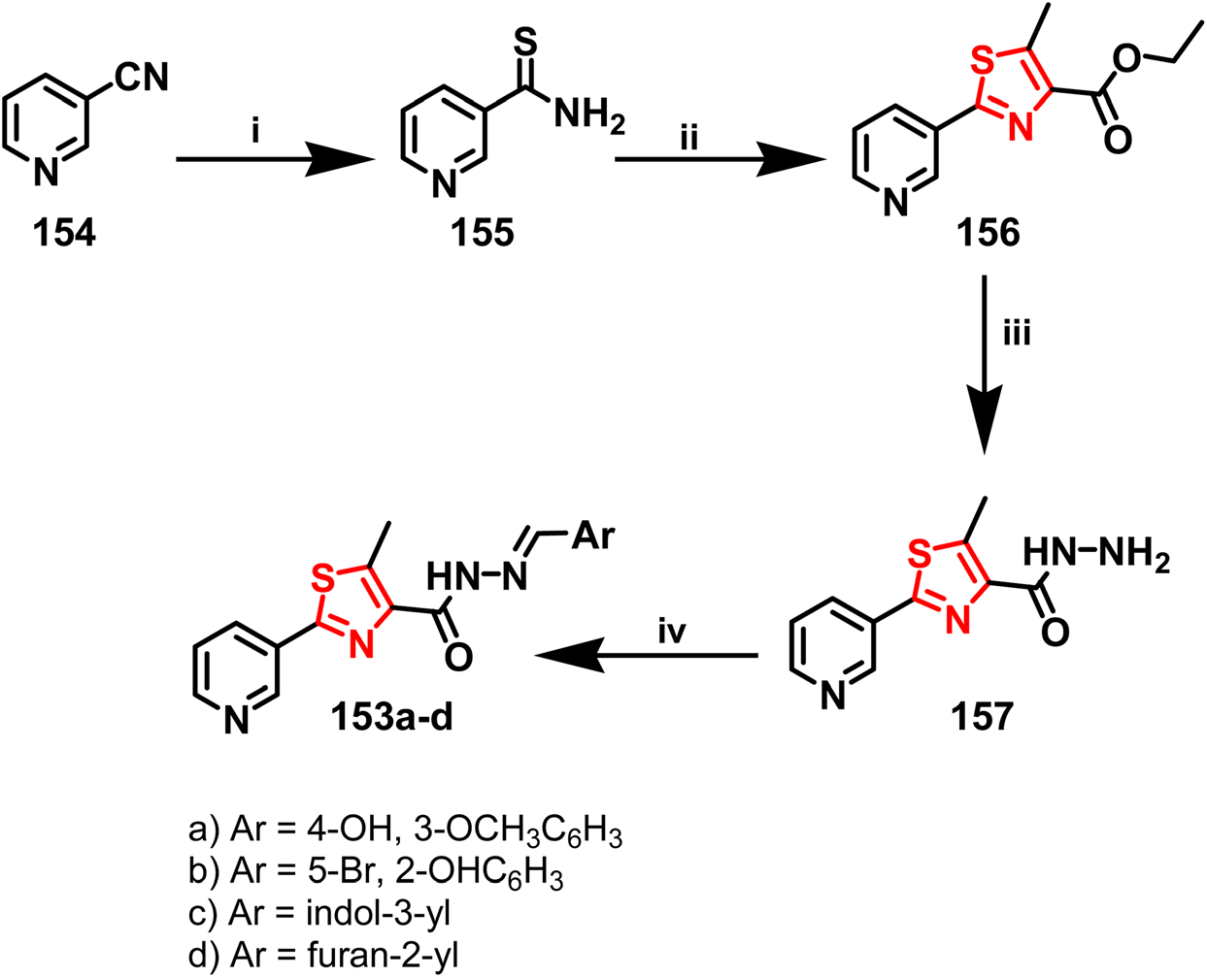
Synthesis of the thiazole-pyridine hybrids 153a–d. Reagents and conditions: (i) P_4_S_10_, ethanol, reflux, 70 °C, 4 h; (ii) ethyl 2-chloroacetoacetate, ethanol, reflux, 70 °C, 8 h; (iii) hydrazine hydrate, ethanol, reflux, 70 °C, 4 h; (iv) Ar-CHO, ethanol, reflux, 70 °C, 12 h.

Initially, 3-cyanopyridine 154 was treated with P_4_S_10_ to yield the corresponding pyridine-3-carbothiamide 155. The refluxing of derivative 155 with ethyl-2-chloroacetoacetate afforded the ester derivative 156. Then, the treatment of the ester derivative 156 with hydrazine hydrate furnishes 5-methyl-2-(pyridine-3-yl)thiazole-4-carbohydrazide 157. Moreover, derivative 157 was condensed with the appropriate aldehyde to get the target pyridine-thiazole hybrids 153a–d (Scheme 34).^97^

### 2.8 Thiazole-pyrimidine hybrids as anti-inflammatory agents

**Fig. 34:**
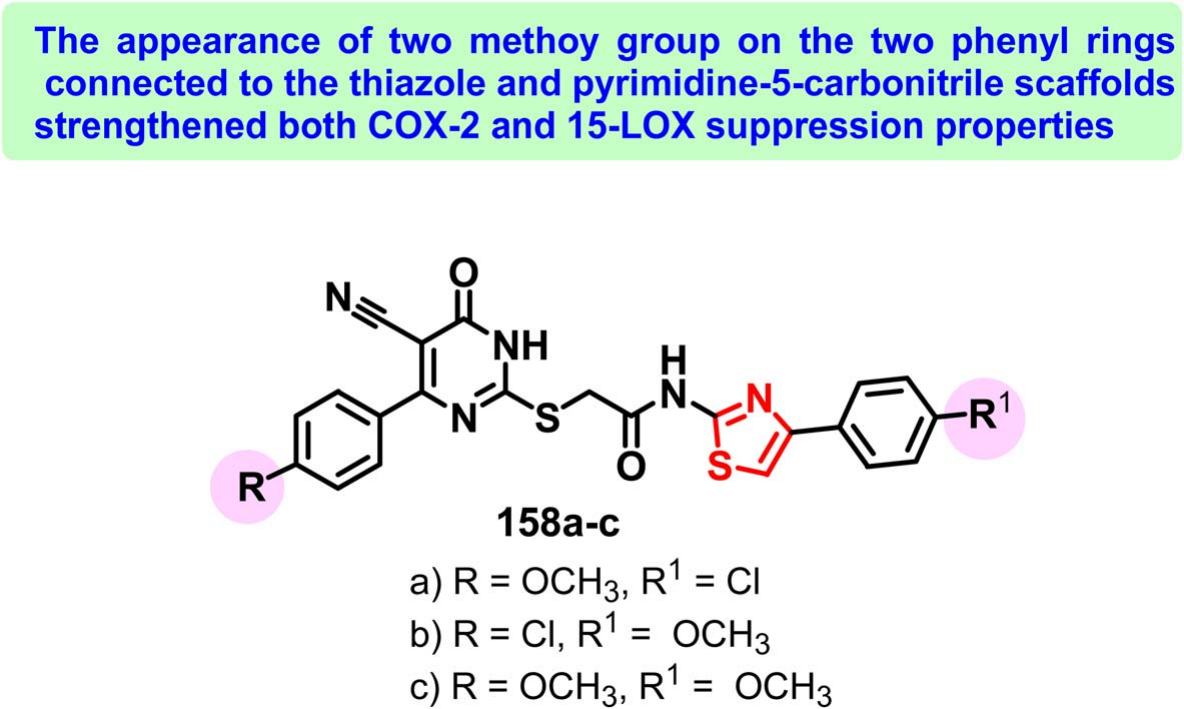
Structures of thiazole carrying pyrimidine-5-carbonitrile candidates 158a–c as anti-inflammatory agents targeting dual COX-2 and 15-LOX enzymes and various inflammatory mediators.

New thiazole carrying pyrimidine-5-carbonitrile candidates 158a–c emerged as promising and selective inhibitors of COX-2 (IC_50_ = 1.71, 1.13 and 1.03 µM, respectively) and COX-2 SI between 5.78 and 8.21 with respect to celecoxib (IC_50_ = 0.88 µM, SI = 8.31). Compared to meclofenamate sodium (IC_50_ = 5.64 µM), derivatives 158b, c were the most effective 15-LOX inhibitors (IC_50_ = 5.73 and 5.29 µM, respectively). It was displayed that the appearance of two methoxy groups on the two phenyl rings connected to the thiazole and pyrimidine-5-carbonitrile scaffolds strengthened both COX-2 and 15-LOX suppression properties. According to the *in vivo* findings, edema inhibition percentages gradually increased for derivatives 158a–c at various time intervals. After 4 h interval, derivatives 158a–c presented EI = 67.19%, 64.35% and 61.56%, respectively, relative to meloxicam (EI = 71.31%). When compared to meloxicam (UI = 18), the ulcerative effects of 158b, c have been demonstrated to have a better safety profile, with ulcer indices of 2.70 and 2.40, respectively. Fortunately, the three tested candidates 158a–c had a considerable decline in rat serum concentrations of PGE2 (inhibition = 62.35–77.94%), IL-6 (inhibition = 63.08–77.44%) and TNF-α (inhibition = 46.86–75.04%) relative to meloxicam (inhibition = 72.62%, 69.74% and 62.36%, respectively). Furthermore, derivative 158c presented a greater affinity to the COX-2 active site (docking score = −17.14 kcal mol^−1^) than that of celecoxib (docking score = −13.40 kcal mol^−1^). The OCH_3_ group (on thiazole Ph.) is involved in the H-bond with His90, while the other OCH_3_ group contributed to two H-bonds with Tyr355 and Arg513. In addition, the CN group created another H-bond with Arg120 (ref. 98) (Fig. 34).

**Scheme 35:**
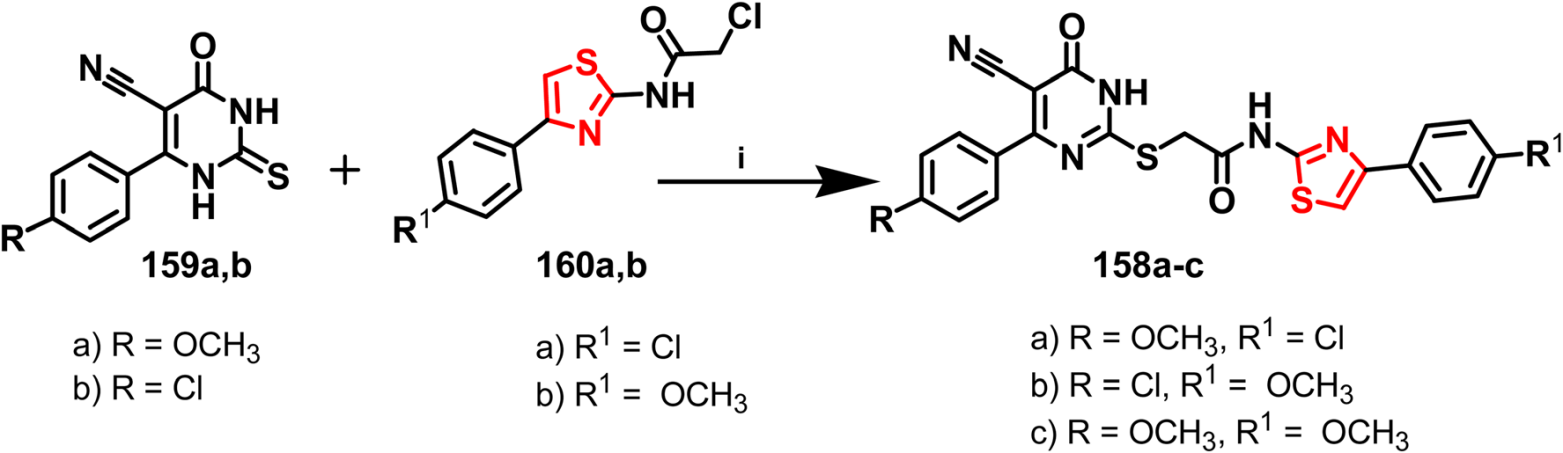
Synthesis of the thiazole carrying pyrimidine-5-carbonitrile candidates 158a–c. Reagents and conditions: (i)K_2_CO_3_, KI, DMF, reflux, 80 °C, 3 h.

The synthesis of intermediates 159a, b (ref. 99) and 160a, b (ref. 100) was described previously. The target derivatives 158a–c were achieved *via* the *S*-alkylation of 159a, b with 160a, b (Scheme 35).^98^

**Fig. 35:**
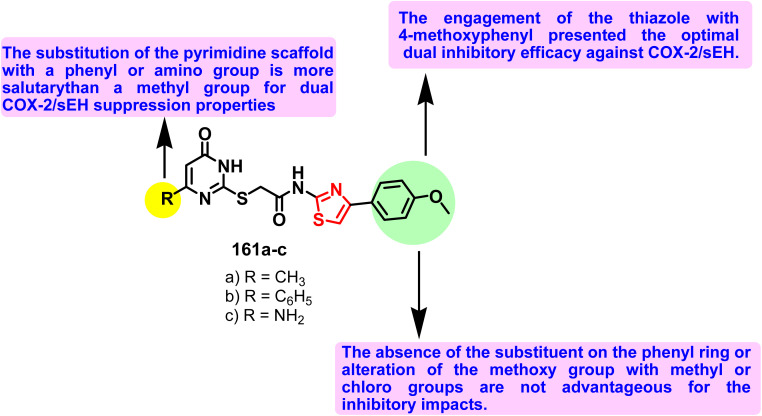
Structures of thiazole-pyrimidine hybrids 161a–c as anti-inflammatory agents targeting dual COX-2/soluble epoxide hydrolase (sEH) enzymes and various inflammatory mediators.

Another thiazole-pyrimidine hybrids 161a–c were disclosed as dual COX-2/soluble epoxide hydrolase (sEH) inhibitors with IC_50_ = 1.43, 1.02 and 1.13 µM, respectively, against COX-2 and IC_50_ = 2.33, 1.97 and 2.01 nM, respectively, against sEH with respect to celecoxib and 12-(3-adamantan-1-yl-ureido)dodecanoic acid standards (IC_50_ = 0.88 µM and 0.80 nM, respectively). It was demonstrated that the engagement of the thiazole with 4-methoxyphenyl presented the optimal dual inhibitory efficacy against COX-2/sEH. However, the absence of the substituent on the phenyl ring or alteration of the methoxy group with methyl or chloro groups are not advantageous for the inhibitory impacts. Otherwise, the substitution of the pyrimidine scaffold with a phenyl or amino group is more salutary than a methyl group for dual COX-2/sEH suppression properties. Furthermore, the hybrids 161a–c substantially reduced serum TNF-α (serum conc. = 58.10–71.80 pg mL^−1^), IL-6 (serum conc. = 74.32–90.80 pg mL^−1^) and PGE2 (serum conc. = 75.56–91.12 pg mL^−1^) levels as compared to celecoxib (serum conc. = 83.72, 110.38 and 84.63 pg mL^−1^). The results demonstrated that the studied hybrids had potent anti-inflammatory actions, surpassing celecoxib with EI% ranging from 33% to 58% 5 hours post derivative administration. Derivative 161b did not disclose any ulceration in the isolated rat stomach, while derivatives 161a, c demonstrated significant hyperemia but no noticeable ulceration (number of gastric ulcers = 1.2 and 0.8, respectively), as compared to celecoxib (number of gastric ulcers = 2.5). Both derivatives 161b, c are well fitted within the COX-2 binding site with docking scores of −16.56 and −16.03 kcal mol^−1^, respectively, superior to that of celecoxib (docking score = – 13.40 kcal mol^−1^). Two H-bonds emerge between the methoxy groups with Ser530 and Tyr385. In addition, Tyr355 and Arg513 established H-bonds with the oxygen of 6-oxopyrimidines. Moreover, hydrophobic interactions were created with the lipophilic moieties. Otherwise, derivatives 161b, c demonstrated the ability to fit into the human sEH binding site with docking scores of −14.37 and −15.78 kcal mol^−1^, respectively. Derivatives 161b, c presented H-bonds in C=O of the thioacetamide linker with Tyr381 and Tyr465. While the NH of thioacetamide established hydrogen bindings with Asp333. Moreover, hydrophobic interactions were noticed^101^ (Fig. 35).

**Scheme 36:**
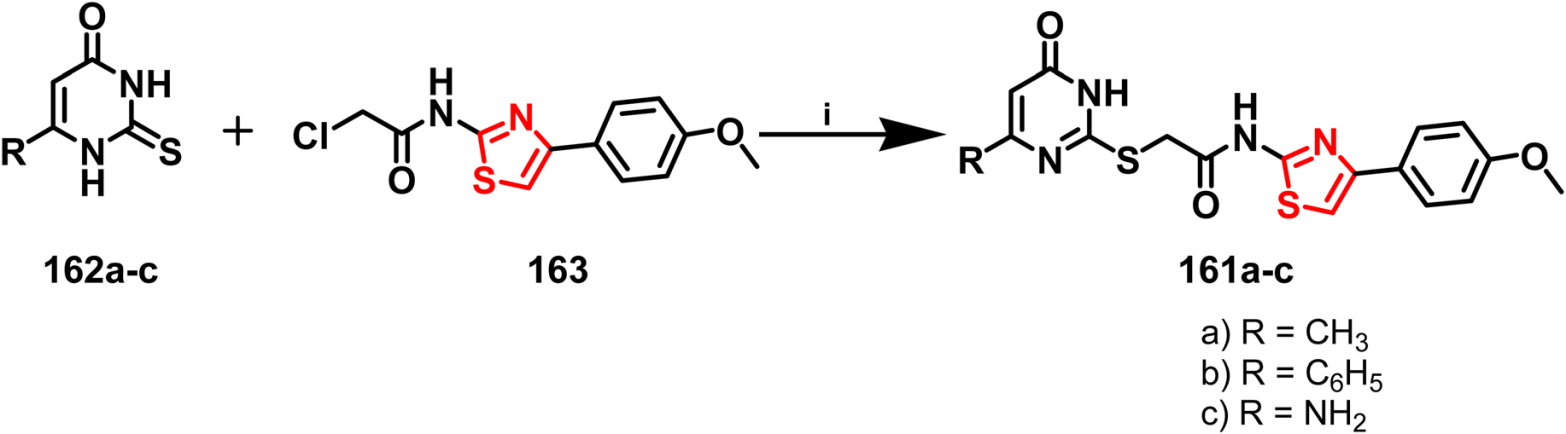
Synthesis of the thiazole-pyrimidine hybrids 161a–c. Reagents and conditions: (i)K_2_CO_3_, KI, DMF, reflux, 80 °C, 3 h.

Thiazole-pyrimidine hybrids 161a–c were prepared *via S*-alkylation of thiouracil derivatives 162a–c with chloroacyl-aminothiazole 163 utilizing anhydrous potassium carbonate (Scheme 36).^101^

**Fig. 36:**
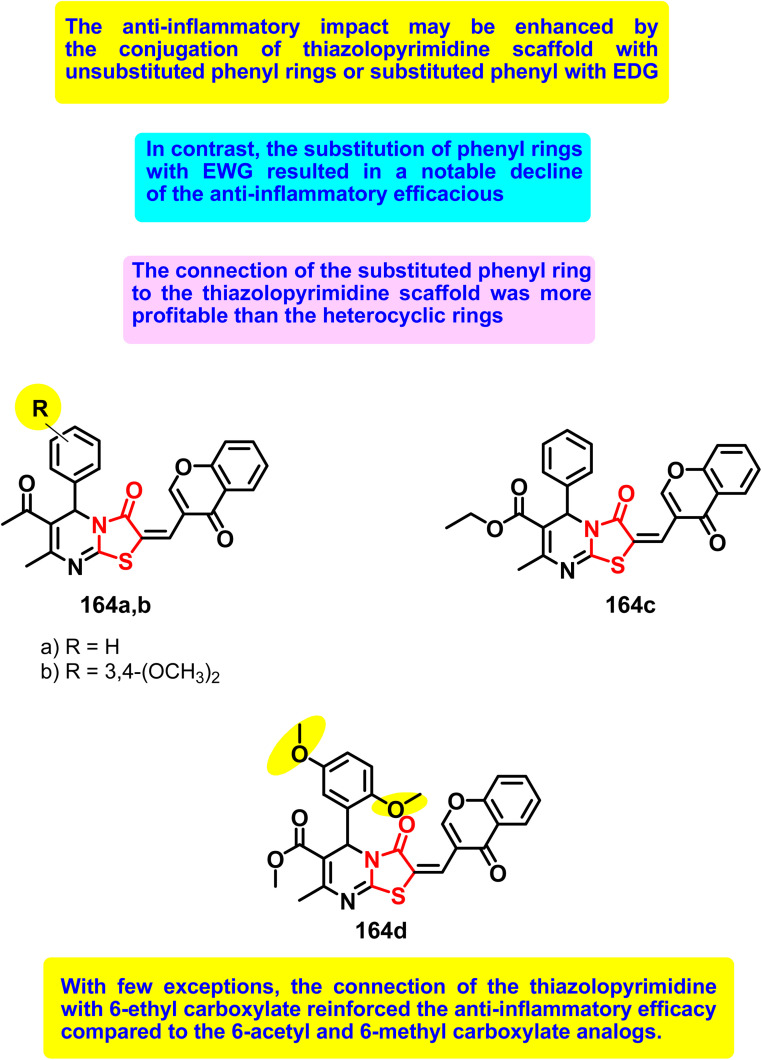
Structures of thiazolopyrimidine-chromone hybrids 164a–d targeting various inflammatory mediators.

The synthesis and *in vivo* anti-inflammatory assessment of novel thiazolopyrimidine-chromone hybrids were demonstrated by Dawood *et al.* Most of the screened hybrids presented outstanding anti-inflammatory efficacy with respect to celecoxib. Particularly, derivatives 164a–d displayed considerable edema inhibition = 78.27%, 75.32%, 79.44% and 84.79%, respectively, after 24 h of administration, comparable to celecoxib (edema inhibition = 80.81%); remarkably, they had no signs of ulceration. Furthermore, the former derivatives stood out as prominent PGE2, IL-6 and TNF-α inhibitors. They manifested notable suppression effectiveness on the release of TNF- α, IL-6, and PGE2 (level of PGE2 varied from 38.46 to 47.21 pg mL^−1^ level of IL-6 from 165.44 to177.17 pg mL^−1^, and level of TNF-α level from 52.30 to 63.56 pg mL^−1^, respectively), in comparison to celecoxib (37.50, 164.49, and 49.77 pg mL^−1^, respectively). The anti-inflammatory impact may be enhanced by the conjugation of the thiazolopyrimidine scaffold with unsubstituted phenyl rings or substituted phenyl with EDG; in contrast, the substitution of phenyl rings with EWG resulted in a notable decline in the anti-inflammatory efficacy. Furthermore, the connection of the substituted phenyl ring to the thiazolopyrimidine scaffold was more profitable than the heterocyclic rings. With few exceptions, the connection of thiazolopyrimidine with 6-ethyl carboxylate reinforced the anti-inflammatory efficacy compared to the 6-acetyl and 6-methyl carboxylate analogs. Moreover, derivatives 164a–d demonstrated high predicted gastric absorption and no BBB penetration. As a result, they are limited to treating peripheral inflammation and are not anticipated to negatively impact the central nervous system. Additionally, these derivatives have an excellent bioavailability rating of 0.55. In addition, the prominent hybrids 164a–d were inserted in the mPGES-1 binding site, resulting in potential energy scores varying from −9.75 to −11.18 kcal mol^−1^. Asn74 and Arg70 amino acids participated in H-bond acceptors with the carbonyl groups of the thiazolone and chromone rings, respectively. Further H-bond acceptors emerged between Arg126 and the C=O groups at the 6th position of the thiazolopyrimidine scaffold in 164a, c. Concerning IL-6, derivatives 164a–d were appropriately embedded with the active site, resulting in energy scores ranging from −9.85 to −10.78 kcal mol^−1^. The chromone scaffolds and the amino acid Gln147 interacted through arene–cation interactions in all the docked derivatives. Moreover, the C=O groups at the 6th position of the thiazolopyrimidine scaffold in 164a, b, d are engaged in H-bonds with Lys105. Conversely, the arene–cation interaction was detected between Lys105 and the phenyl ring at the 5th position of thiazolo[3,2-*a*] pyrimidine in 164c. Furthermore, the carboxylate moiety in 164d demonstrated an H-bond donor with Ser109. Furthermore, derivatives 164a–d presented significant docking scores inside the TNF-α active pocket ranging from −9.88 to 10.63 kcal mol^−1^. Chromone scaffolds were found to provide either hydrophobic or hydrophilic interactions with Gly12. In addition, Tyr119 showed arene–cation interactions with the phenyl ring at the 5th position of thiazolopyrimidine in 164a, c, whereas Tyr119 was forced to bind to the thiazole moiety in 164b, d due to substitution with dimethoxyphenyl^102^ (Fig. 36).

**Scheme 37:**
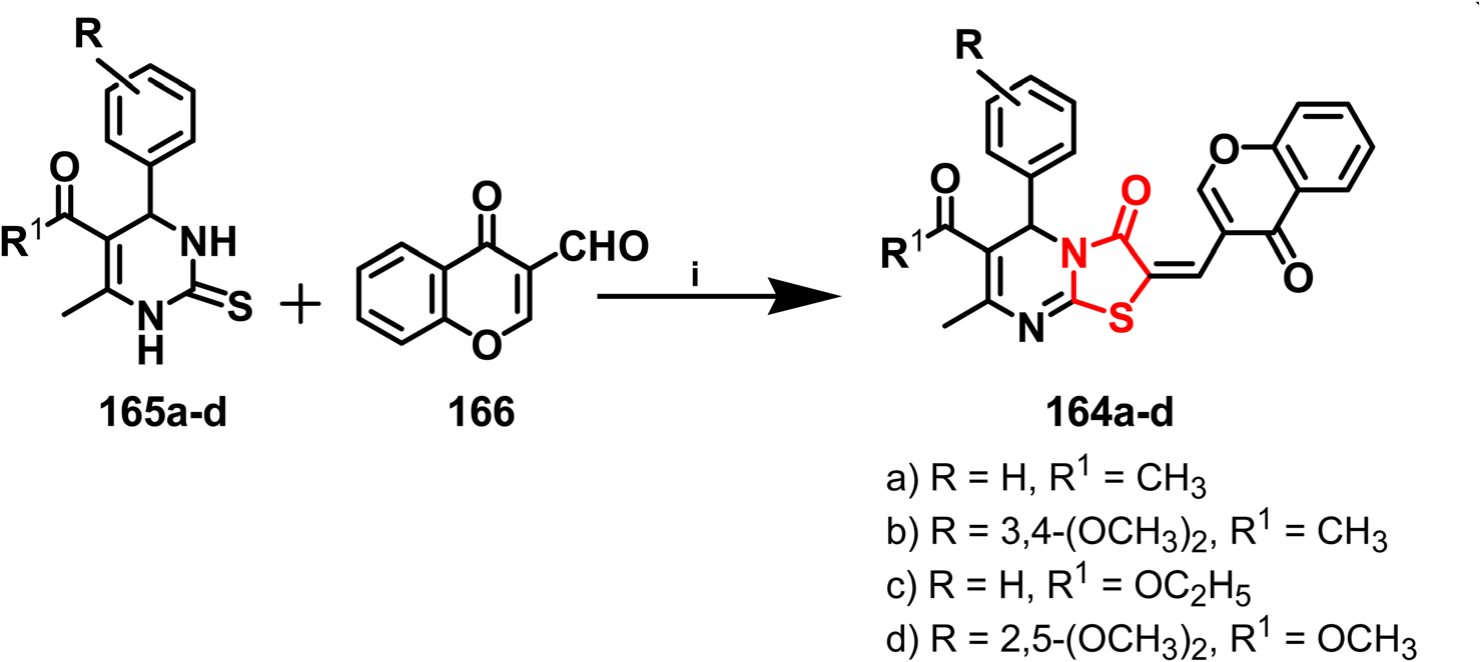
Synthesis of the thiazolopyrimidine-chromone hybrids 164a–d. Reagents and conditions: (i) ClCH_2_COOH, AcOH, AC_2_O, sodium acetate, reflux, 130 °C, 3–5 h.

The intermediates 1,2,3,4-tetrahydropyrimidine-2-thiones 165a–d and chromone-3-carboxaldehyde 166 were refluxed with a mono-chloroacetic acid in a mixture of acetic acid and acetic anhydride solvents employing anhydrous sodium acetate to yield the new target chromone-thiazolopyrimidine hybrids 164a–d in a single step (Scheme 37).^102^

### 2.9 Thiazole-indole hybrids as anti-inflammatory agents

**Fig. 37:**
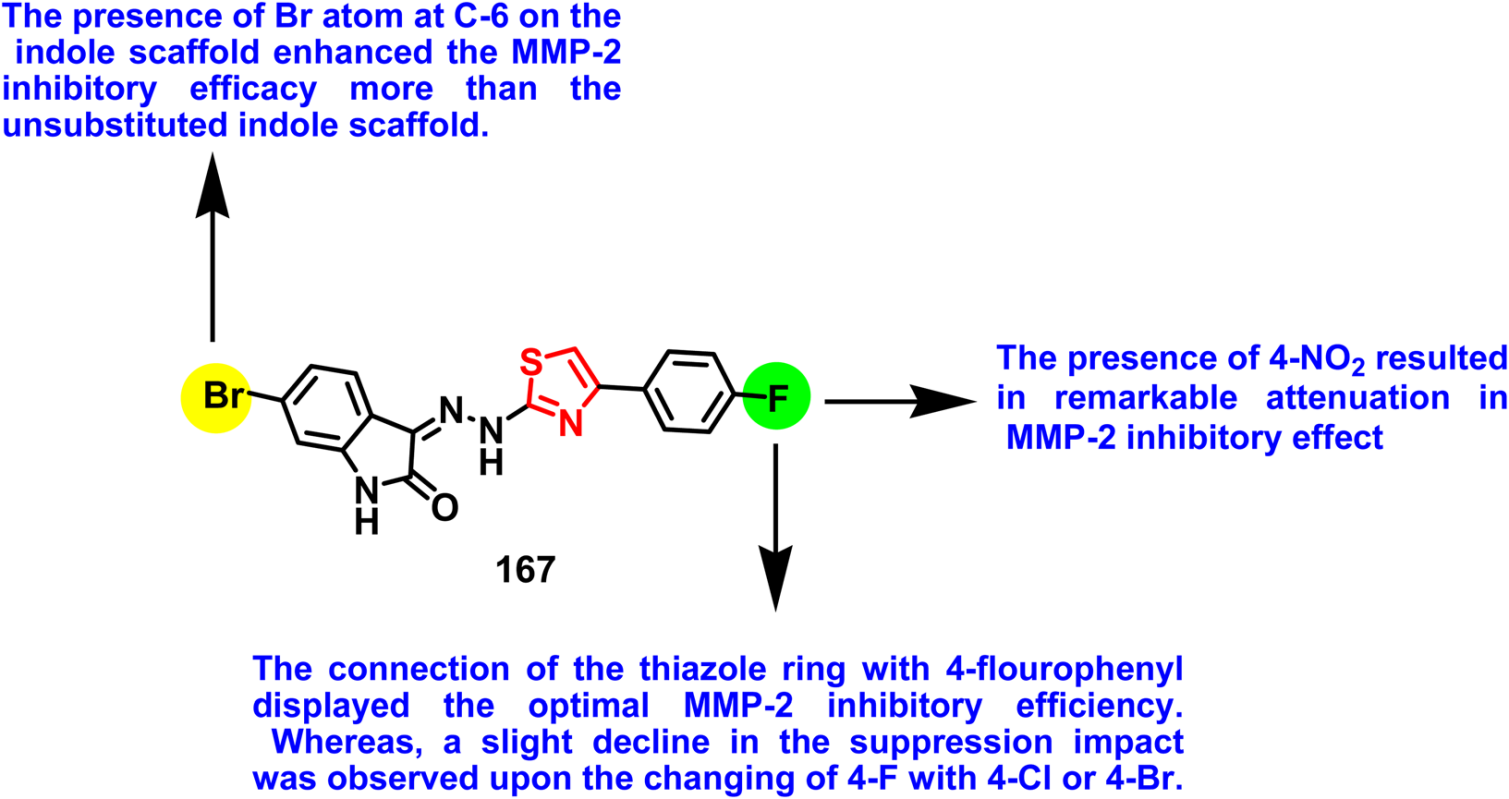
Structure of isatin-thiazole hybrid 167 as an anti-inflammatory agent.

Veeranna *et al.*^103^ described the synthesis and the anti-inflammatory evaluation of new isatin-thiazole hybrids. Among the tested conjugates, derivative 167 displayed eminent inhibitory effects against MMP-2 and MMP-9 (inhibition = 80% and 50%, respectively). It was concluded that the presence of a Br atom at C-6 on the indole scaffold enhanced the inhibitory efficacy against MMP-2 more than the unsubstituted indole scaffold. Furthermore, the connection of the thiazole ring with 4-flourophenyl displayed the optimal MMP-2 inhibitory efficiency, whereas a slight decline in the suppression impact was observed upon the changing of 4-F with 4-Cl or 4-Br. However, the presence of 4-NO_2_ resulted in remarkable attenuation in MMP-2 inhibitory effects (Fig. 37).

**Scheme 38:**
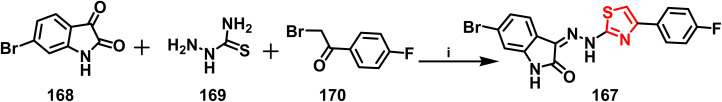
Synthesis of the isatin-thiazole hybrid 167. Reagents and conditions: (i) EtOH, AcOH, reflux, 80 °C, 3 h.

The reaction of 5-bromo isatin 168, thiosemicarbazide 169 and 4-fluoro phenacylbromide 170 furnished the isatin-thiazole hybrid 167 (Scheme 38).^103^

**Fig. 38:**
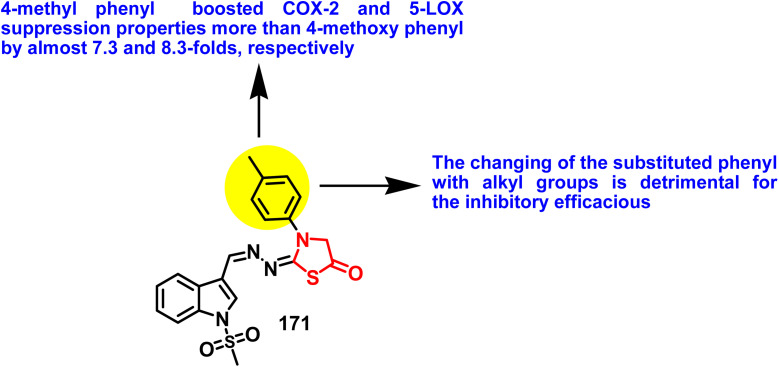
Structure of indole-thiazolidinone candidate 171 as an anti-inflammatory agent targeting dual COX-2 and 5-LOX enzymes and TNF-α.

New indole-thiazolidinone candidate 171 stood out as a dual COX-2 and 5-LOX inhibitor (IC_50_ = 0.67 and 1.10 µM, respectively) with respect to celecoxib and zileuton (IC_50_ = 0.46 and 0.58 µM, respectively). It was inferred that the conjugation of the thiazolidinone ring with 4-methyl phenyl at the 3rd position boosted COX-2 and 5-LOX suppression properties more than 4-methoxy phenyl by almost 7.3 and 8.3 folds, respectively. Otherwise, the changing of the substituted phenyl with alkyl groups is detrimental for the inhibitory efficacy. In comparison to celecoxib (UI = 3.97), derivative 171 displayed an outstanding ulcer index (1.3). The stomach histopathological investigation manifested that the administration of derivative 171 caused a minor decrease in the thickening of the muscularis mucosa, a partial loss of the mucous layer with mild, superficial erosions, the preservation of the number of parietal cells and chief cells in superficial and deep mucosa, and mild mucosal, submucosal edema and inflammations. In addition, derivative 171 demonstrated remarkable *in vitro* anti-inflammatory impacts by declining the TNF-α fold to 0.21, compared to that of celecoxib (0.16). Furthermore, blood cardiac biomarkers troponin I, CK-MB, and LDH were lower in rats treated with derivative 171 (0.0015 ng mL^−1^, 0.315 ng mL^−1^ and 536 U L^−1^, respectively) than in rats treated with celecoxib (0.1500 ng mL^−1^, 0.635 ng mL^−1^ and 690 U L^−1^, respectively), suggesting that derivative 171 had a lower probability of cardiovascular stroke. Derivative 171 presented the highest docking scores within COX-2 active pocket (−6.44 kcal mol^−1^). It created Arene–H interaction between *p*-tolyl moiety and Ser339. Moreover, derivative 171 demonstrated the greatest docking scores within the 5-LOX active site. His432 and His600 interacted hydrophobically with the phenyl group of the indole ring. Additionally, derivative 171 displayed a high intestinal absorption of 99.50%; in addition, it revealed poor to mild permeability for the *in vitro* MDCK and CaCo-2 cells (0.04 and 18.63 nm s^−1^, respectively). Moreover, it possessed a substantial binding impact on plasma protein (100%); however, it had a minimal absorption in the central nervous system (0.09). Interestingly, its log *K*_p_ value = −1.74 cm h^−1^, indicating maximal skin permeability. As a result, it could be a viable candidate for the transdermal delivery systems^104^ (Fig. 38).

**Scheme 39:**
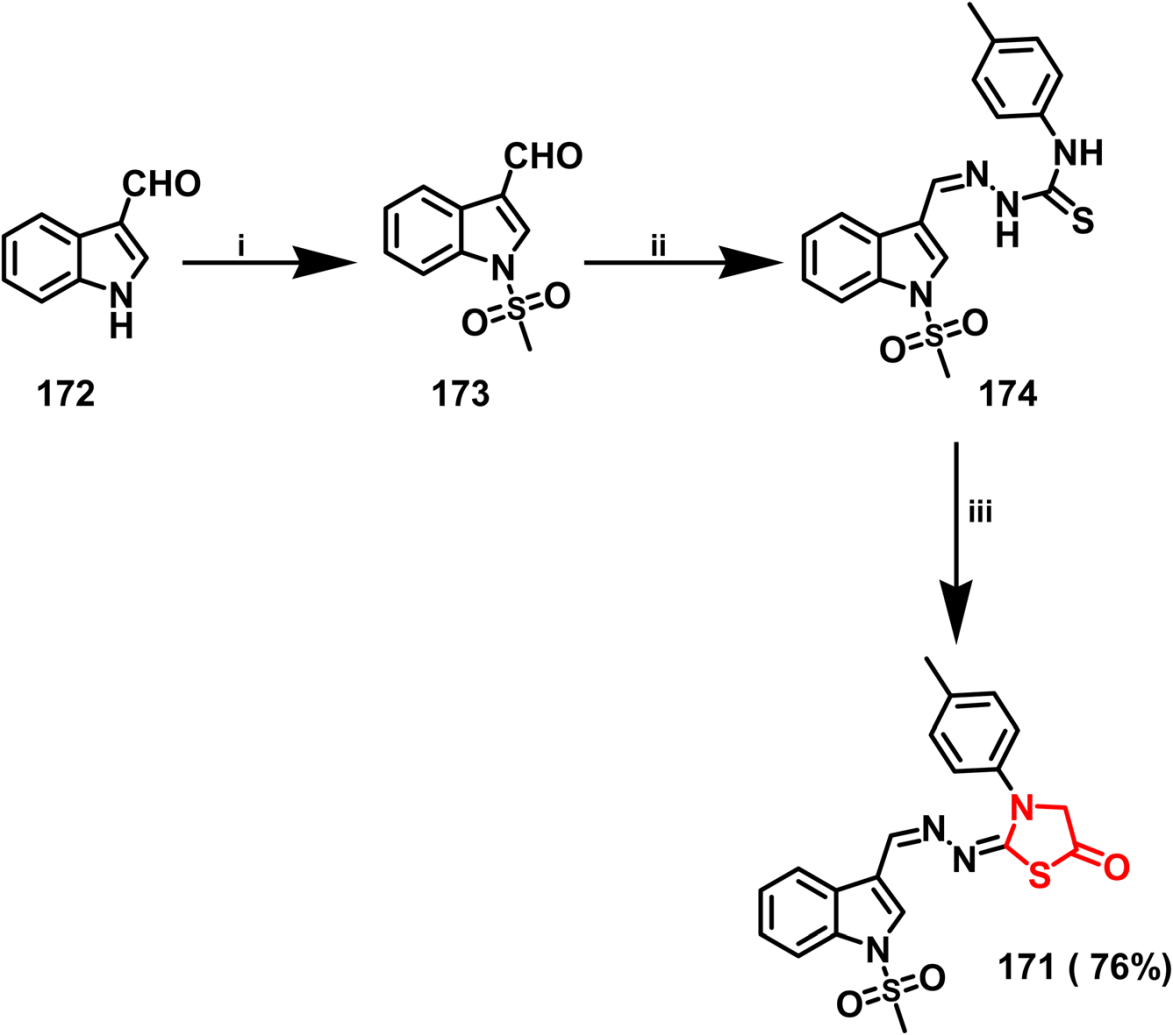
Synthesis of the indole-thiazolidinone candidate 171. Reagents and conditions: (i) CH_3_SO_2_Cl, NaH, DMF, RT, 3 h; (ii) 4-methyl phenylthiosemicarbazide, EtOH/drops DMF, reflux, 4 h; (iii) ClCH_2_COOEt, NaAc, abs. EtOH/drops DMF, reflux, 6 h.

The *N* alkylation of indole-3-carboxaldehyde 172 furnished the *N*-methylsulfonylindole carboxaldehyde analog 173, which underwent a reaction with 4-methyl phenyl thiosemicarbazide to obtain the thiosemicarbazone derivative 174. The corresponding thiazolidinone 171 was then accomplished by cyclizing the obtained thiosemicarbazone derivative 174 using ethyl chloroacetate and sodium acetate (Scheme 39).^104^

**Fig. 39:**
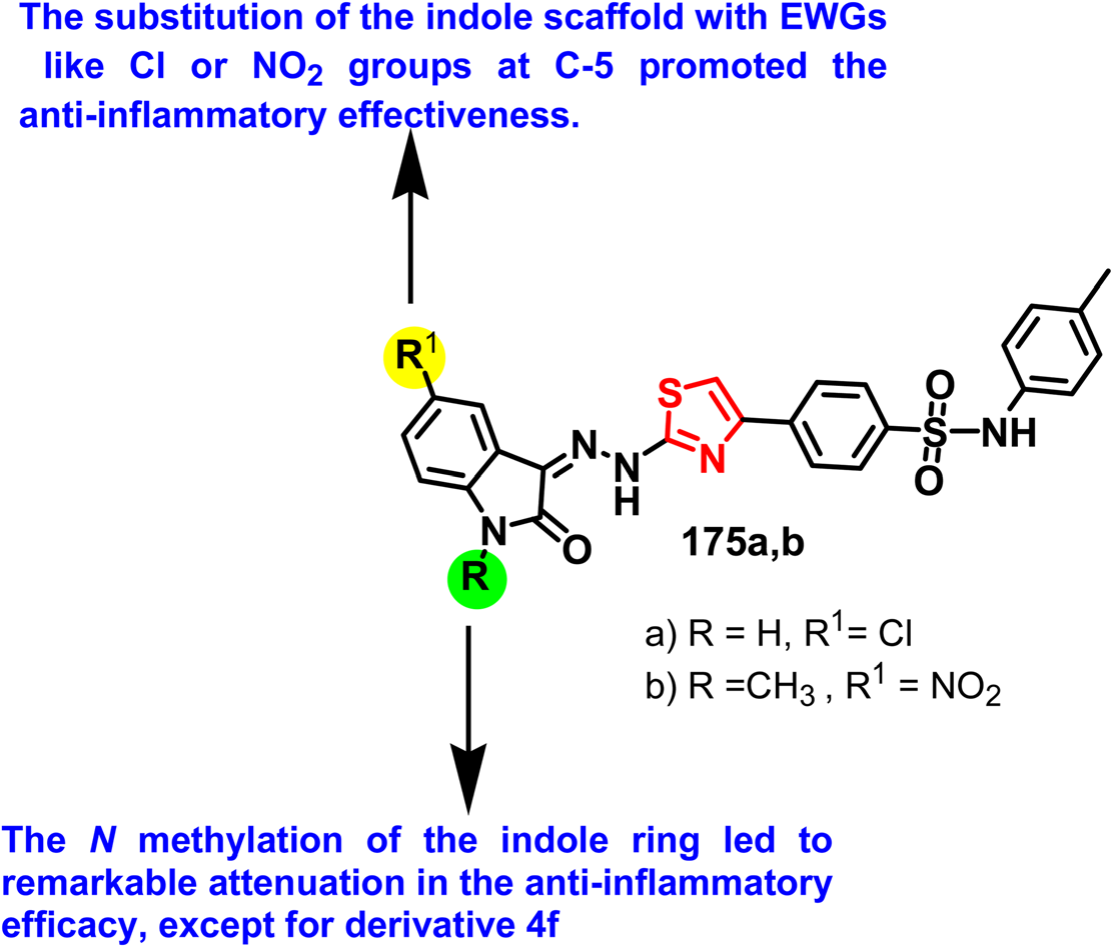
Structures of thiazole-indolin-2-one hybrids 175a, b as anti-inflammatory agents targeting the COX-2 enzyme.

New thiazole-indolin-2-one hybrids 175a, b were discovered as promising anti-inflammatory candidates. They displayed edema inhibition = 45.63% and 41.84%, respectively, with respect to celecoxib (edema inhibition = 49.30%) 5 h post administration. It was noticed that the substitution of the indole scaffold with EWGs such as Cl or NO_2_ groups at C-5 promoted the anti-inflammatory effectiveness. However, the *N*-methylation of the indole ring led to remarkable attenuation in the anti-inflammatory efficacy, except for derivative 175b. Moreover, derivatives 175a, b demonstrated moderate to low COX-2 suppression impacts (IC_50_ = 8.26 and 23.03 µM, respectively) with SI = 2.65 and 4.32, respectively, relative to celecoxib (IC_50_ = 1.60 µM, SI = 4.44). The histopathological investigation on the paw tissue revealed that the administration of derivative 175a displayed a remarkable decline in the inflammatory cells and edema. Moreover, the glandular mucosa and submucosa revealed a generally normal histological structure without any abnormalities, after the administration of derivatives 175a, b. Derivatives 175a, b manifested good fitting inside the COX-2 active region with docking scores = −11.45 and −10.48 kcal mol^−1^, respectively. Derivative 175a displayed several H-bonds with Asp229, Arg376, Gl235 and Trp139 amino acids, in addition to pi-alkyl interactions with Pro538 and Leu145. Furthermore, derivative 175a exhibited pi-cation and amide-pi interactions with Lys333 and Leu224, respectively. Moreover, van der Waals interactions were detected with Asn375 and Ser143. Otherwise, derivative 175b demonstrated 3 H-bonding with Gly135, Cys41 and Arg44. Moreover, other interactions were disclosed as van der Waals interactions with Arg469, Ala156 and Asn34 and amide-pi interaction with Val155, in addition to pi-alkyl bindings with Ala156, Pro153 and Lys468 (ref. 105) (Fig. 39).

**Scheme 40:**
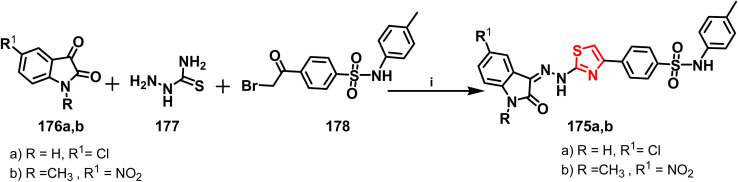
Synthesis of the thiazole-indolin-2-one hybrids 175a, b. Reagents and conditions: (i) EtOH,TEA, reflux, 80 °C, 4 h.

The new thiazole-indolin-2-one hybrids 175a, b were accomplished *via* a one-pot reaction of 5-chloro-isatin/5-nitro-*N*-methyl-isatin 176a, b, thiosemicarbazide 177 and substituted phenacyl bromide 178 (Scheme 40).^105^

### 2.10 Thiazolidinone-based derivatives as anti-inflammatory agents

**Fig. 40:**
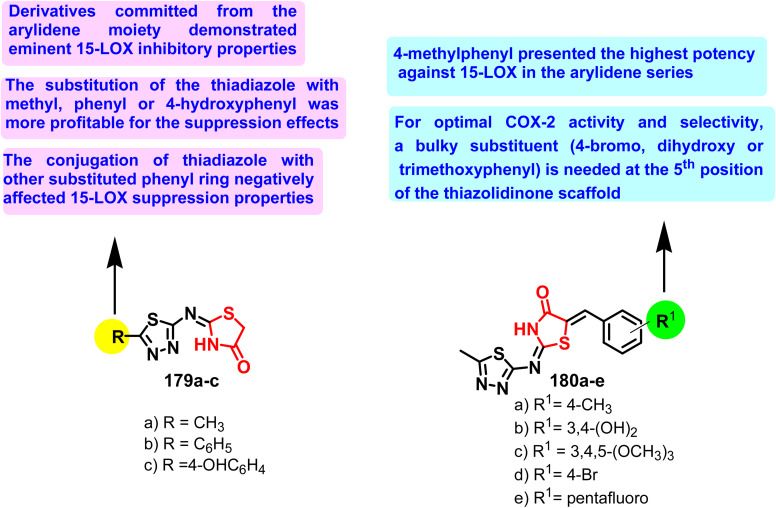
Structures of thiazolidinone-thiadiazole hybrids 179a–c and 180a–e as anti-inflammatory agents targeting COX-2 and 15-LOX enzymes.

Omar *et al.*^106^ described the preparation and the inhibitory assessment of novel thiazolidinone-thiadiazole hybrids against COX-2 and 15-LOX enzymes. Derivatives 179a–c and 180a presented a notable 15-LOX suppression impact (IC_50_ = 2.74, 2.54, 3.11 and 3.11 µM, respectively) superior to zileuton (IC_50_ = 15.6 µM). Derivatives 179a–c committed from the arylidene moiety demonstrated eminent 15-LOX inhibitory properties. It was apparent that the substitution of thiadiazole with methyl, phenyl or 4-hydroxyphenyl was more profitable for the suppression effects, whilst the conjugation of thiadiazole with other substituted phenyl rings negatively affected 15-LOX suppression properties. Derivative 180a with 4-methylphenyl presented the highest potency against 15-LOX in the arylidene series. The LOX suppression efficacy was reduced by lengthening the aliphatic substituent on the phenyl ring. Otherwise, it was disclosed that derivatives 180b–e were the most prominent and selective COX-2 inhibitors with IC_50_ of 0.11, 0.1, 0.1 and 0.1 µM, respectively, and selectivity indices of 103.82, 123.40, 114.20 and 151.10, respectively, with respect to Diclofenac Sodium (IC_50_ = 0.32 µM, SI = 16.78). It was deduced that for optimal COX-2 activity and selectivity, a bulky substituent (4-bromo, dihydroxy or trimethoxyphenyl) is needed at the fifth position of the thiazolidinone scaffold. The potency was significantly attenuated by a reducing number of hydroxyl or methoxy groups. Furthermore, COX-2 effectiveness is significantly reduced when the bromine atom is swapped out for a chlorine atom. Moreover, comparable *in vivo* anti-inflammatory action was demonstrated by derivative 179a (edema inhibition = 44.8%) to celecoxib (edema inhibition = 40%). In contrast to indomethacin, which caused edema, the gastric mucosa did not exhibit any signs of ulceration, edema, or desquamation of the epithelial cells after the administration of derivative 179a. The docking of derivative 179a with the 15-LOX binding site displayed the formation of the H-bond between Ile 676 and NH of the thiazolidinone core; besides, the carbonyl group and iron metal combine to produce a metal complex. Conversely, docking analysis within COX-2 revealed the formation of H-bond between N of thiadiazole with Arg 513 for derivatives 180c, e. Additionally, derivative 180c displayed further binding with Gln192, while derivative 180d presented a H-bond with Arg 120 rather than Arg 513 and a pi–H interaction with Tyr 355 (Fig. 40).

**Scheme 41:**
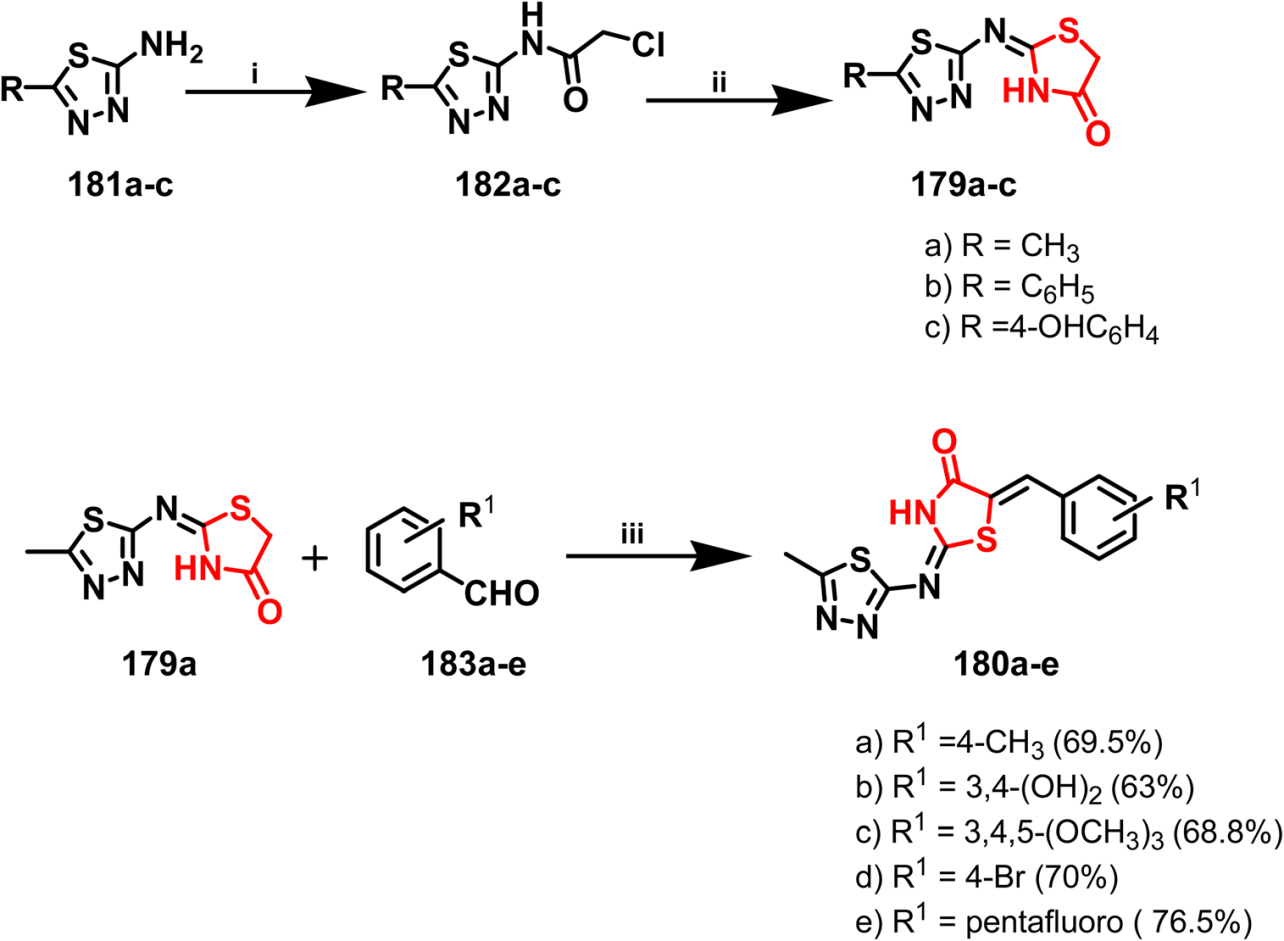
Synthesis of the thiazolidinone-thiadiazole hybrids 179a–c and 180a–e. Reagents and conditions: (i) ClCH_2_COCl, benzene, reflux, 3–5 h; (ii) NH_4_SCN, EtOH, reflux, 3–5 h; (iii) R_1_CHO, sodium acetate/acetic acid buffer, reflux, 24 h.

Derivatives 182a–c were produced by refluxing thiadiazole-2-amines 181a–c with chloroacetyl chloride in dry benzene. The thiazolidinones 179a–c were achieved by heterocyclization of 182a–c with ammonium thiocyanate. Using acetic acid/sodium acetate buffer, Knoevenagel condensation of the thiazolidinone derivative 179a with varied aldehydes 183a–e yielded target derivatives 180a–e (Scheme 41).^106^

**Fig. 41:**
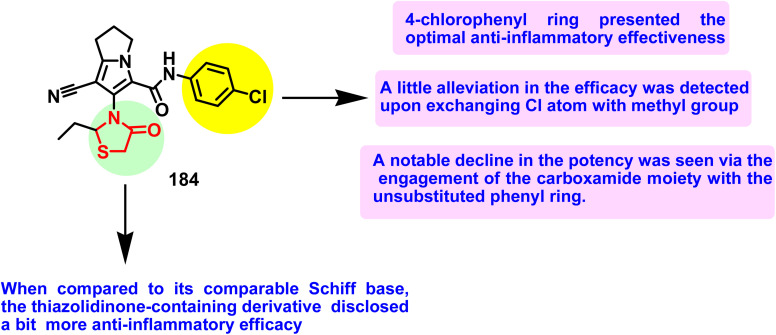
Structure of thiazolidinone derivative 184 as an anti-inflammatory agent targeting the COX-2 enzyme.

Shawky *et al.*^107^ illustrated the preparation of new pyrrolizine-bearing Schiff bases and their cyclized thiazolidinone analogs as prominent anti-inflammatory candidates. Among the thiazolidinone analogs, derivative 184 emerged as an eminent COX-2 suppressor (IC_50_ = 1.15 µM, SI > 86); however, it was less effective than celecoxib by almost 4.6-fold. Additionally, it demonstrated a significant *in vivo* anti-inflammatory impact (edema inhibition = 32.48%) 4 h post-administration with respect to indomethacin (edema inhibition = 56.39%). When compared to its comparable Schiff base, the thiazolidinone-containing derivative 184 disclosed a bit more anti-inflammatory efficacy. In addition, the combination of the carboxamide moiety with a 4-chlorophenyl ring presented the optimal anti-inflammatory effectiveness, while a little alleviation in the efficacy was detected upon exchanging the Cl atom with a methyl group. Otherwise, a notable decline in the potency was seen *via* the engagement of the carboxamide moiety with the unsubstituted phenyl ring. Furthermore, derivative 184 demonstrated excellent potential selectivity for COX-2 with inhibition constants of 43.29 and 1070 nM for COX-2 and COX-1, respectively. The two pi–sulfur interactions with Tyr385 and Trp387 as well as the pi-cation interaction with Arg513 may be accountable for the increased affinity to COX-2. Moreover, one H-bond was formed between the cyano group and Ser530 (Fig. 41).

**Scheme 42:**
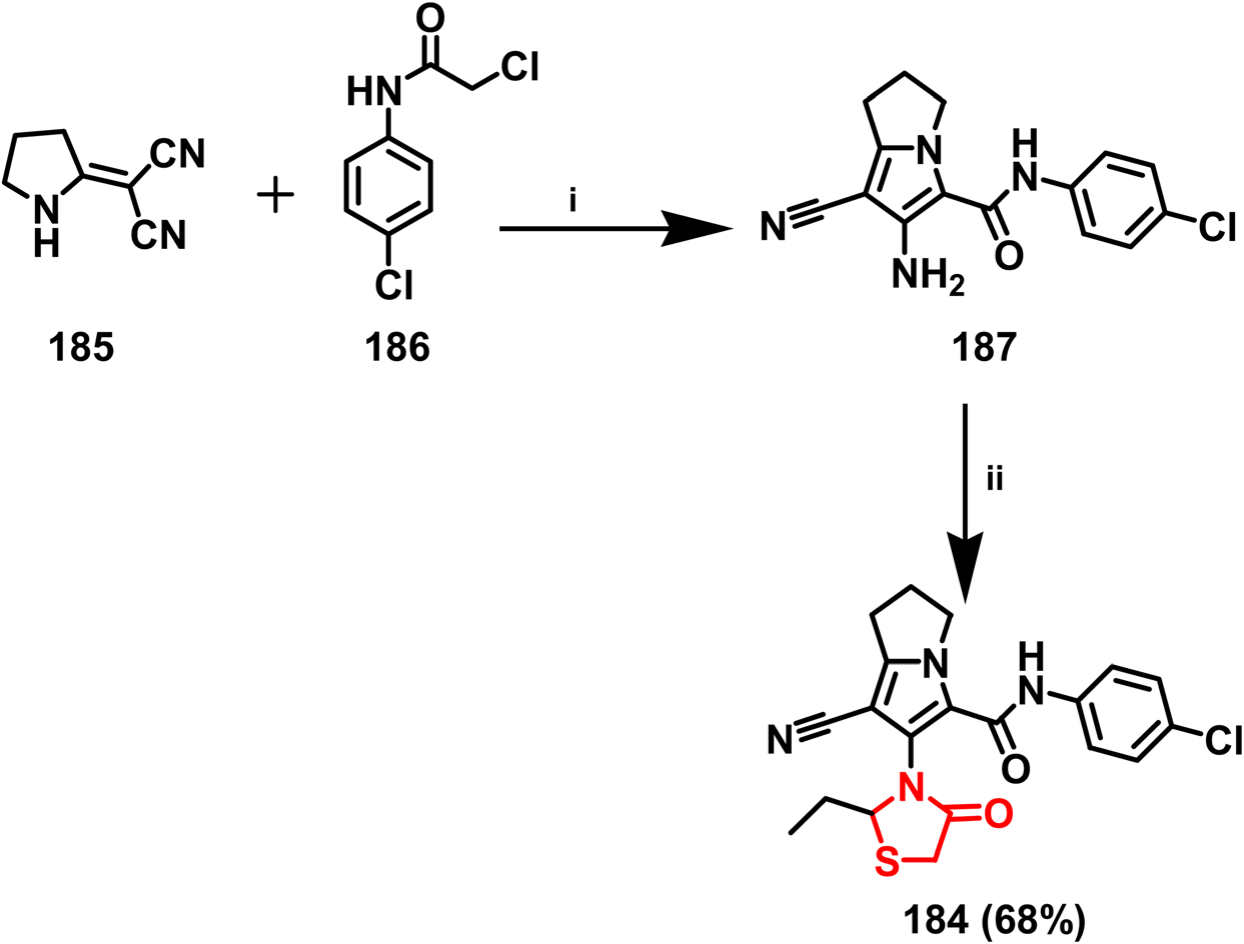
Synthesis of the thiazolidinone derivative 184. Reagents and conditions: (i) acetone, K_2_CO_3_, reflux, 24 h; (ii) propionaldehyde, mercaptoacetic acid, DCC, THF, 0 °C.

The preparation of derivative 187 is illustrated in Scheme 42, following the preceding methods. Furthermore, the thiazolidinone derivative 184 was accomplished from the reaction of derivative 187 with propionaldehyde and mercaptoacetic acid.^107^

**Fig. 42:**
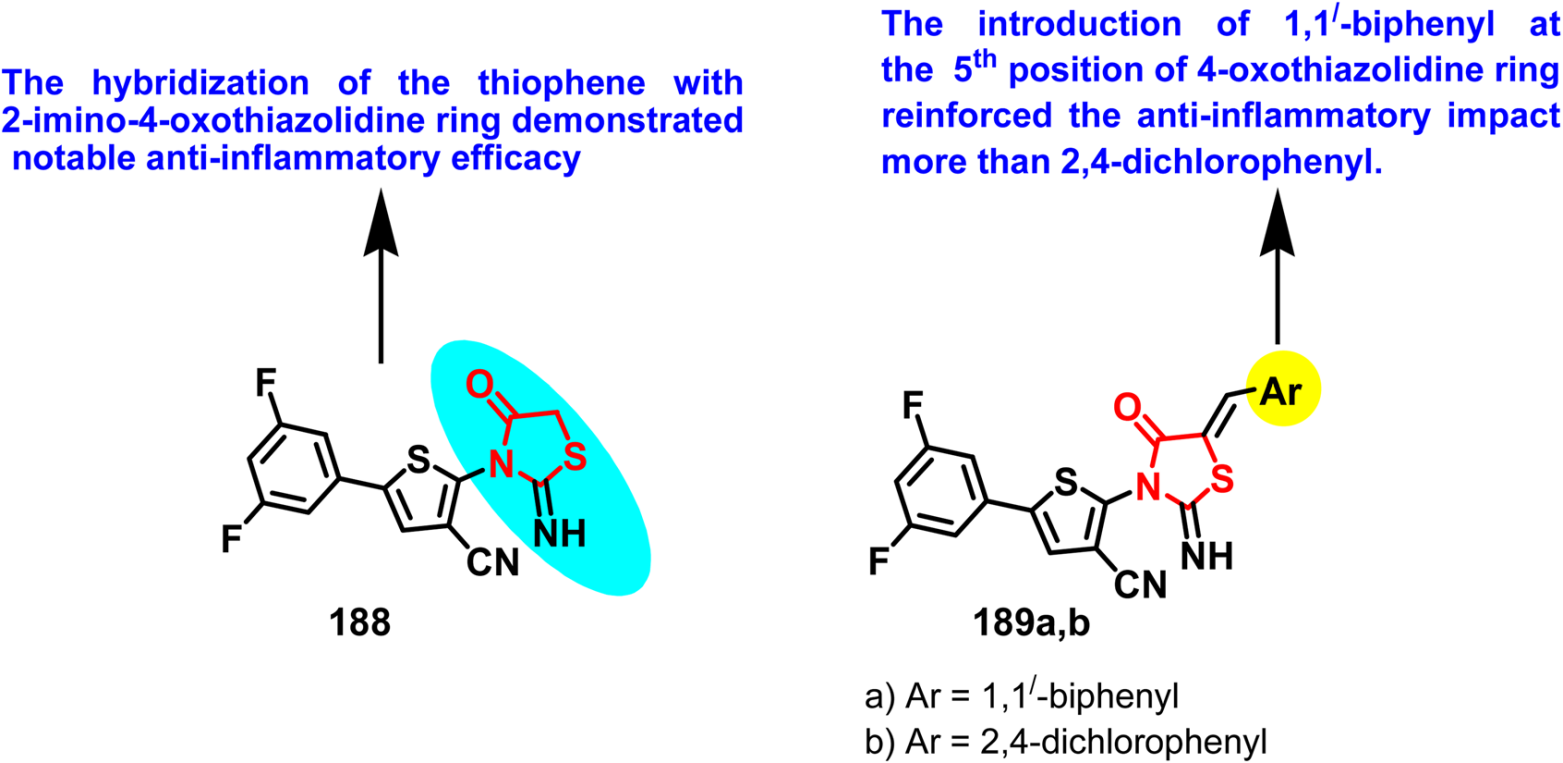
Structures of thiazolidin-4-one-thiophene hybrids 188 and 189a, b as anti-inflammatory agents.

New thiazolidin-4-one-thiophene hybrids 188 and 189a, b were described as prominent anti-inflammatory candidates utilizing an albumin denaturation inhibition assay (IC_50_ = 41.1, 40.9 and 44.7 µg mL^−1^, respectively) relative to diclofenac sodium (IC_50_ = 31.4 µg mL^−1^). These outcomes pointed out that the hybridization of thiophene with the 2-imino-4-oxothiazolidine ring demonstrated notable anti-inflammatory efficacy. Moreover, the introduction of 1,1′-biphenyl at the 5th position of the 4-oxothiazolidine ring reinforced the anti-inflammatory impact more than 2,4-dichlorophenyl. Based on the hemolytic assay at 1000 µg mL^−1^, derivatives 188 and 189a, b are shown to be safe even at larger doses since they are almost nontoxic to red blood cell membranes (hemolysis = 2.56%, 1.57% and 5.17%, respectively)^108^ (Fig. 42).

**Scheme 43:**
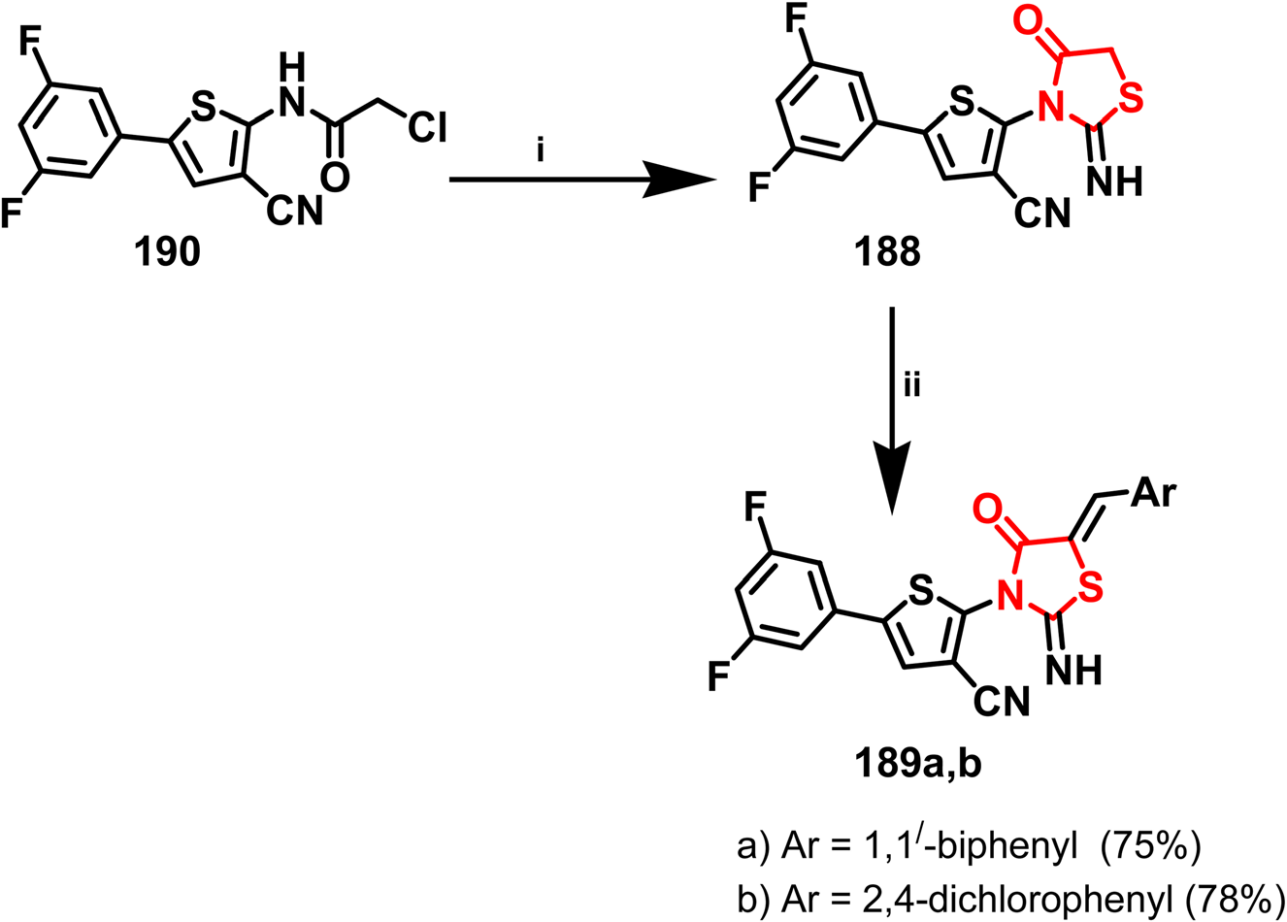
Synthesis of the thiazolidin-4-one-thiophene hybrids 188 and 189a, b. Reagents and conditions: (i) dry acetone, KSCN, reflux; (ii) substituted benzaldehydes, ethanol, catalytic amount of acetic acid, NaOAc, reflux.

The intramolecular cyclization of derivative 190 with potassium thiocyanate in the presence of dry acetone afforded derivative 188. Furthermore, the treatment of intermediate 188 with diverse aromatic aldehydes in acetic acid catalyzed by sodium acetate-furnished derivatives 189a, b (Scheme 43).^108^

**Fig. 43:**
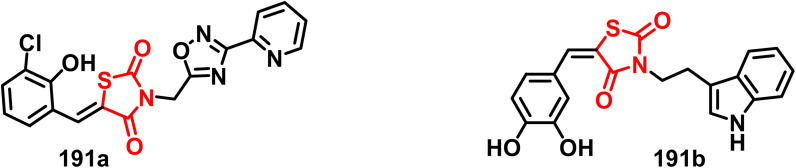
Structures of 2,4-thiazolidinedione-based derivatives 191a, b as mPGES-1 and 5-LOX inhibitors.

Nine 2,4-thiazolidinedione-based derivatives were elected from the virtual screening method to synthesize and investigate their inhibitory effectiveness against mPGES-1 and 5-LOX. Among the screened candidates, derivative 191a was the most prominent mPGES-1 inhibitor (IC_50_ = 3.5 µM), while derivative 191b demonstrated the highest suppression impact against 5-LOX (IC_50_ = 0.2 µM). Moreover, the docking of derivative 191a within the binding region of mPGES-1 displayed π–π interactions between His53 and Phe44 with the terminal 3-chloro-2-hydroxyphenyl group, in addition to π–π interaction between Tyr130 and the oxadiazole ring. Furthermore, various polar interactions were detected with Asp49, Gln36, Thr131 and Arg126 (ref. 109) (Fig. 43).

**Scheme 44:**
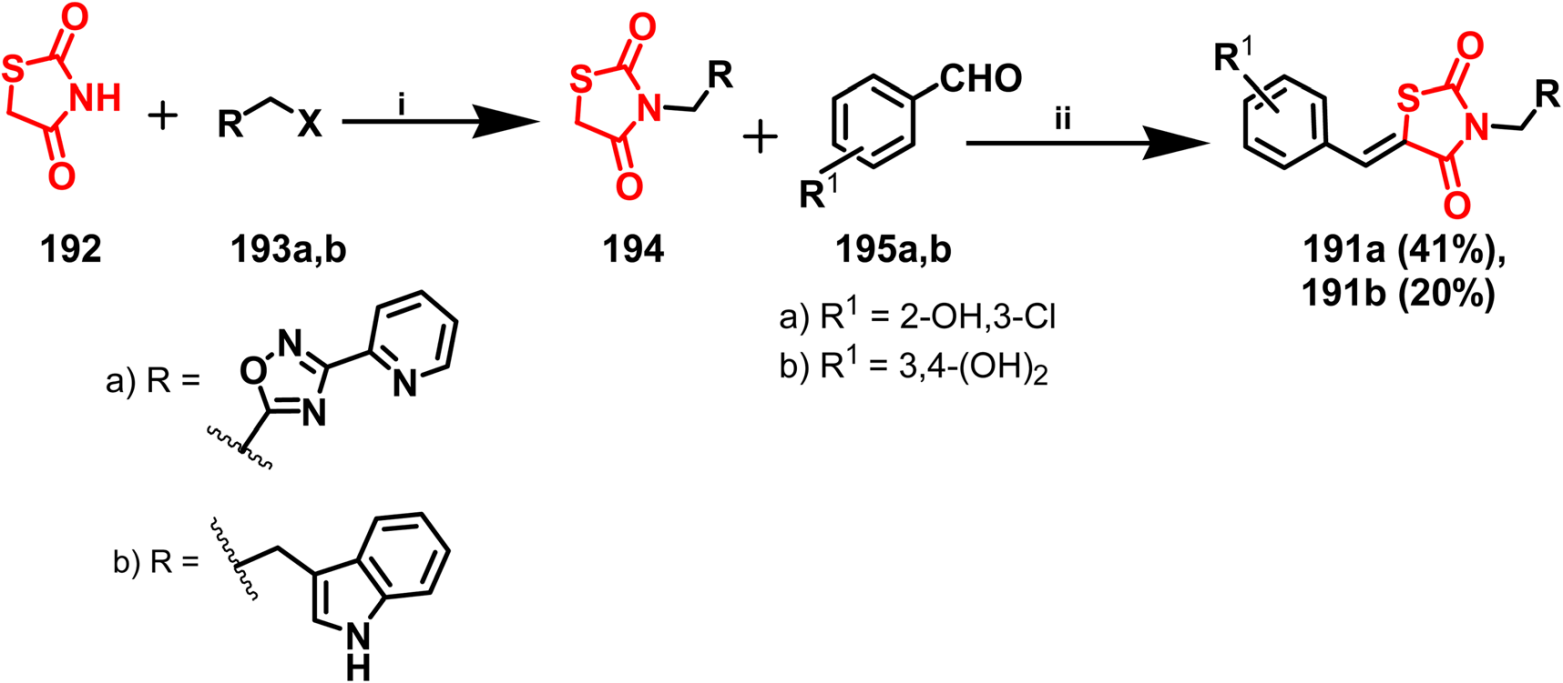
Synthesis of the 2,4-thiazolidinedione-based derivatives 191a, b. Reagents and conditions: (i) NaH, THFdry, reflux, 3 h; (ii) piperidine, EtOH, reflux, overnight.

The reaction of thiazolidine-2,4-dione 192 with varied aliphatic halides 193a, b afforded derivatives 194a, b, which underwent Knoevenagel condensation with various aromatic aldehydes 195a, b to afford the target derivatives 191a, b (Scheme 44).^109^

**Fig. 44:**
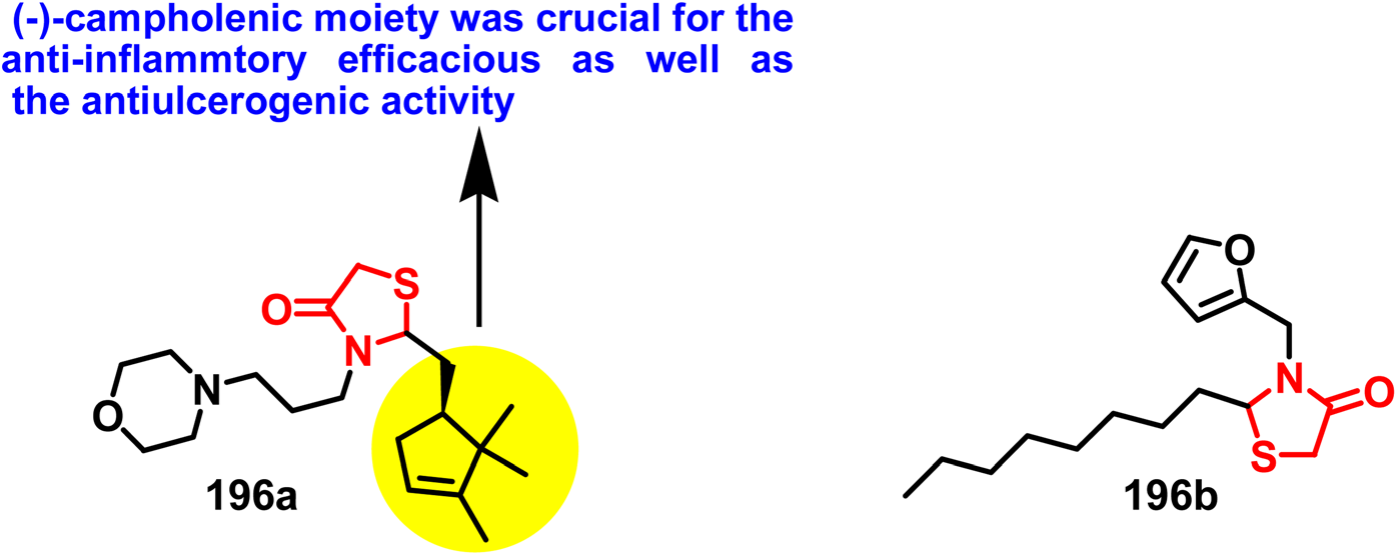
Structures of thiazolidinone derivatives 196a, b as anti-inflammatory agents.

Borisova *et al.*^110^ illustrated the synthesis and the anti-inflammatory estimation of novel thiazolidinones. Among the evaluated candidates, derivatives 196a, b have displayed eminent anti-inflammatory effects (inflammatory edema = 20.74% and 19.65%, respectively) superior to that of diclofenac (inflammatory edema = 22%) utilizing the histamine-stimulated inflammatory edema assay. Furthermore, derivatives 196a, b manifested notable protection against indomethacin-induced ulceration (antiulcerative activity = 5.2 and 3.2, respectively) relative to that of drug omeprazole (antiulcerative activity = 3.8). The pharmacological findings inferred that the (−)-campholenic moiety was crucial for the anti-inflammatory efficacy as well as the anti-ulcerogenic activity (Fig. 44).

**Scheme 45:**
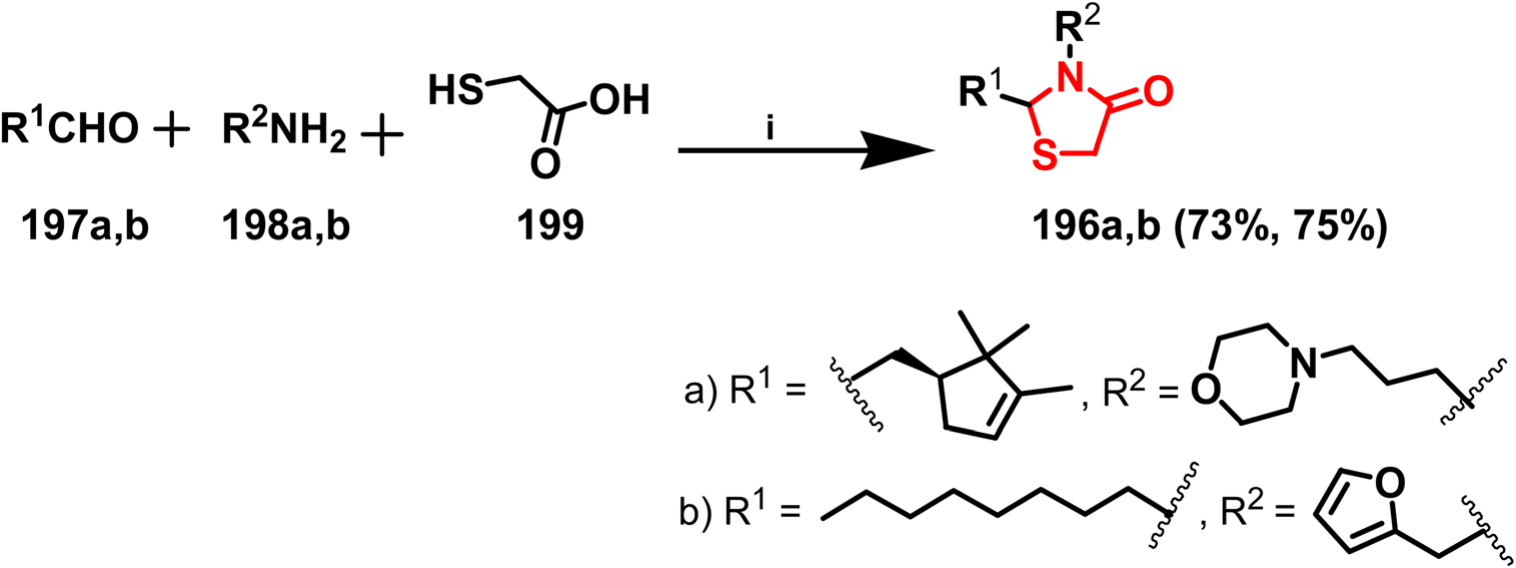
Synthesis of the thiazolidinones derivatives 196a, b. Reagents and conditions: (i) THF, dicyclohexylcarbodiimide, stirring, 0 °C, 3 h.

The reaction of the aldehydes 197a, b with various amines 198a, b and thioglycolic acid 199 utilizing dicyclohexylcarbodiimide afforded the bioactive thiazolidinones 196a, b (Scheme 45).^110^

**Fig. 45:**
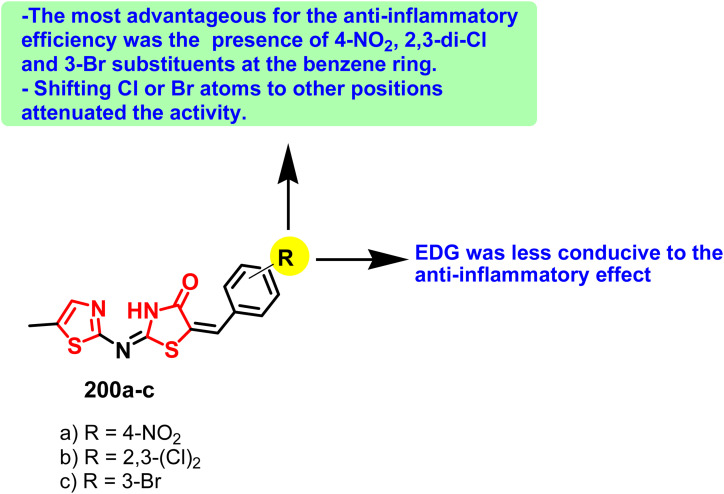
Structures of 5-methylthiazole-thiazolidinone hybrids 200a–c as anti-inflammatory agents targeting the COX-1 enzyme.

The conjugates of 5-methylthiazole-thiazolidinone were synthesized and assessed based on PASS predicted results for their anti-inflammatory properties. Earlier, this tool was utilized to look for COX/LOX inhibitors. The Pa value, which represents the likelihood that a chemical belongs to the class of “active drugs”. The likelihood of confirming the experiment's activity increases with the Pa value. Considering the outcomes of the prediction, derivatives 200a–c with high Pa varying from 0.455 to 0.581 were among the selected derivatives for the experimental assessment of biological efficiency. *In silico* evaluation of the anti-inflammatory properties of these derivatives was carried out against the molecular targets of the LOX, COX-1, and COX-2 enzymes. Derivatives 200a–c displayed superior docking scores against the COX-1 ranging from −9.54 to −11.82 kcal mol^−1^, according to the predicted docking simulation. However, their molecular interactions with LOX and COX-2 were modest. Moreover, compared to naproxen and ibuprofen, two reference medications, derivatives (200a–c) had exceptional molecular interactions against COX-1, suggesting that they may have anti-inflammatory characteristics. A promising anti-inflammatory profile was demonstrated by derivatives 200a–c, which inhibited edema by 55.4%, 57.8% and 57.6%, respectively, relative to indomethacin 47%. The most advantageous for the anti-inflammatory efficiency was the presence of 4-NO_2_, 2,3-di-Cl and 3-Br substituents at the benzene ring. In addition, shifting Cl or Br atoms to other positions attenuated the activity. Moreover, it was discovered that the EDG was less conducive to the anti-inflammatory effect. Based on the *in vitro* test, derivatives 200a–c seemed to be prominent COX-1 inhibitors (IC_50_ = 14.38, 1.10 and 1.08 µM, respectively), generally outperforming naproxen (IC_50_ = 40.10 µM). Otherwise, they did not exhibit any inhibitory activity against the COX-2 enzyme, whereas the three screened derivatives that were tested possessed weak suppression impact against LOX when compared to the reference compound NDGA^111^ (Fig. 45).

**Scheme 46:**
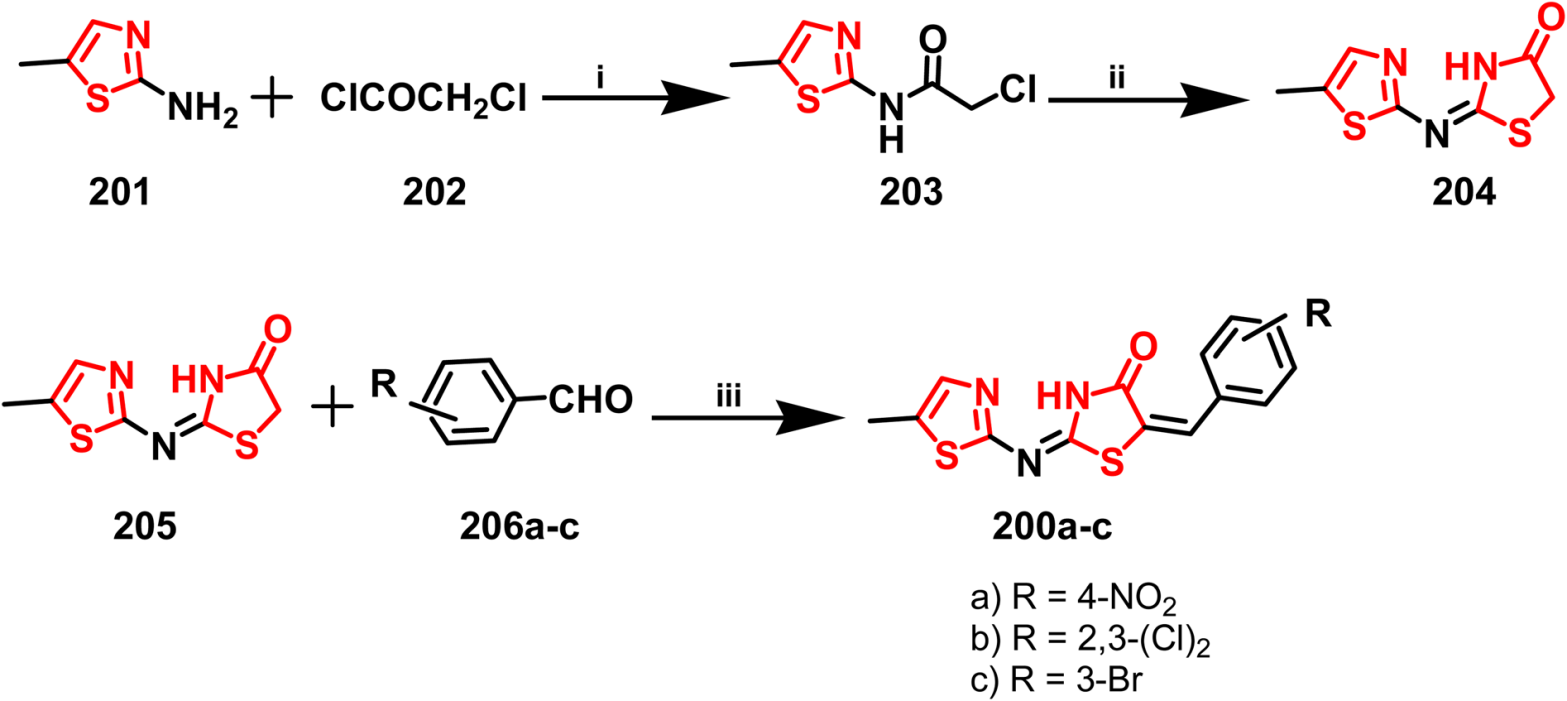
Synthesis of the 5-methylthiazole-thiazolidinone hybrids 200a–c. Reagents and conditions: (i) DMF, RT, 3 h; (ii) NH_4_SCN, EtOH, reflux, 1 h; (iii) AcOH, CH_3_COONa, reflux, 4 h.

Conjugates of 5-methylthiazole-thiazolidinone 200a–c were synthesized as presented in Scheme 46.^111^

**Fig. 46:**
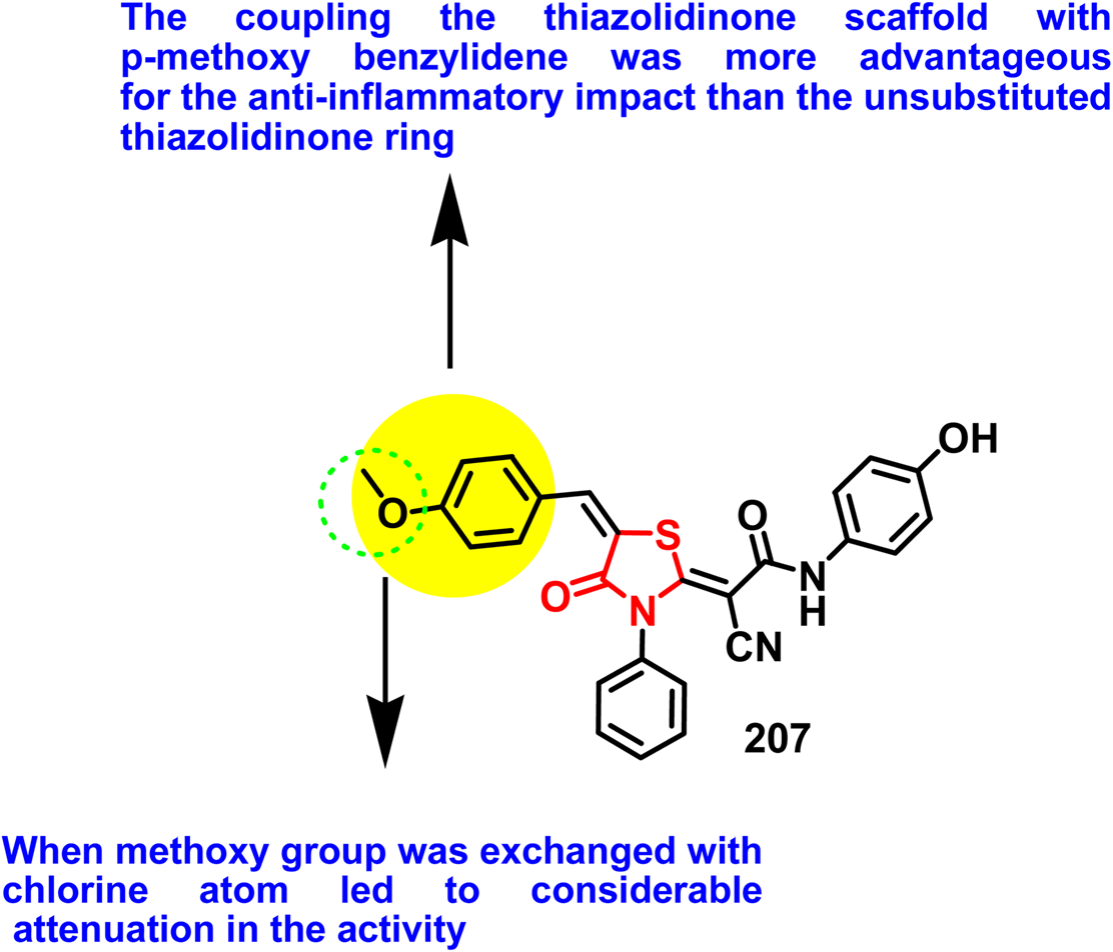
Structure of thiazolidinone derivative 207 as an anti-inflammatory agent.

The thiazolidinone derivative 207 stood out as an eminent anti-inflammatory candidate displaying high edema inhibition ranging from 32.82% to 58.3% at different time intervals, which was greater than that of phenylbutazone (edema inhibition ranging from 0.72% to 43.32%). Additionally, it demonstrated notable IL-6 suppression efficacy (inhibition = 89.31%) superior to that of phenylbutazone (inhibition = 85.59%). It was recognized that coupling the thiazolidinone scaffold with *p*-methoxy benzylidene was more advantageous for the anti-inflammatory impact than the unsubstituted thiazolidinone ring. Moreover, when the methoxy group was exchanged with a chlorine atom, it led to considerable attenuation in the activity. Interestingly, Rats given 207 disclosed that the surface portion of their stomach mucosa looked normal. According to the ADME analysis, derivative 207 demonstrated improved gastrointestinal absorption as well as P-gp non-inhibitors; additionally, it failed to pass through the BBB. Derivative 207 was seen to be stabilized within the IL-6 active site *via* two H-bond acceptors and donors between amidic oxygen and OH groups with Cys102 and Thr188, respectively^112^ (Fig. 46).

**Scheme 47:**
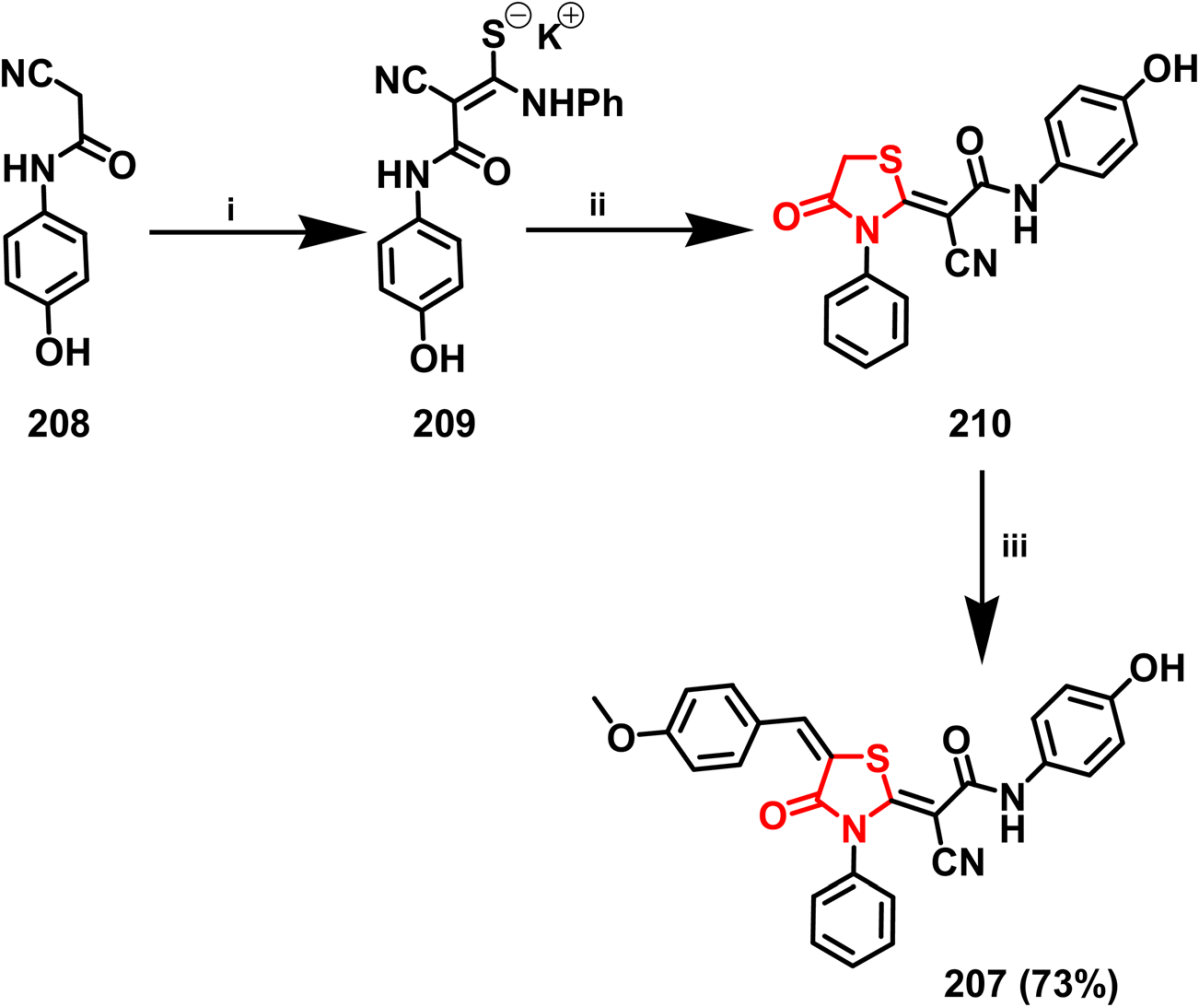
Synthesis of the thiazolidinone derivative 207. Reagents and conditions: (i) PhNCS, DMF, KOH, stirring, 3 h; (ii) ClCH_2_COOC_2_H_5_, stirring, 2 h; (iii) 4- methoxybenzaldehyde, EtOH, piperidine, reflux, 3 h.

The non-isolable intermediate salt 209 was produced by condensing the active methylene group in cyanoacetanilide 208 with phenylisothiocyanate. The reaction between the non-isolable intermediate salt 209 and ethyl chloroacetate yielded the thiazolidinone derivative 210, which underwent reaction with 4-methoxybenzaldehyde to get target 207 (Scheme 47).^112^

**Fig. 47:**
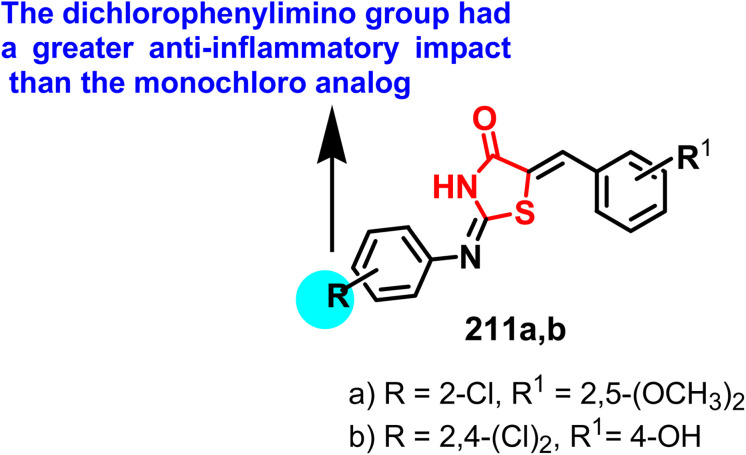
Structures of thiazolidinone derivatives 211a, b as anti-inflammatory agents targeting the COX-2 enzyme and various inflammatory mediators.

The *in vivo* anti-inflammatory assay demonstrated that the thiazolidinone derivatives 211a, b possessed eminent edema inhibition of 45% and 58%, respectively, with respect to etoricoxib (64%) 4 h post derivative administration. In addition, TNF-α and IL-6 levels were considerably reduced in the mice receiving the derivatives 211a, b (TNF-α levels = 64 and 58 pg mL^−1^, respectively; IL-6 levels = 65 and 58 pg mL^−1^, respectively). The dichlorophenylimino group had a greater anti-inflammatory impact than the monochloro analog. Lipinski's rule of five is not violated according to *in silico* properties. The outcome demonstrated that all computed physicochemical descriptors and pharmacokinetic parameters are within the anticipated thresholds. Furthermore, derivatives 211a, b presented good docking scores (−8.89 and −9.03 kcal mol^−1^, respectively) identical to etoricoxib (−9.00 kcal mol^−1^) inside the active site of COX-2. Derivatives 211a, b disclosed the participation of S, C=O and NH groups of the thiazolidinone ring in three H-bonds with Gln447 and Cys26, respectively. While the 5-OCH_3_ and OH groups created H-bonds with Ala142 and Pro140 for derivatives 211a, b, respectively^113^ (Fig. 47).

**Scheme 48:**
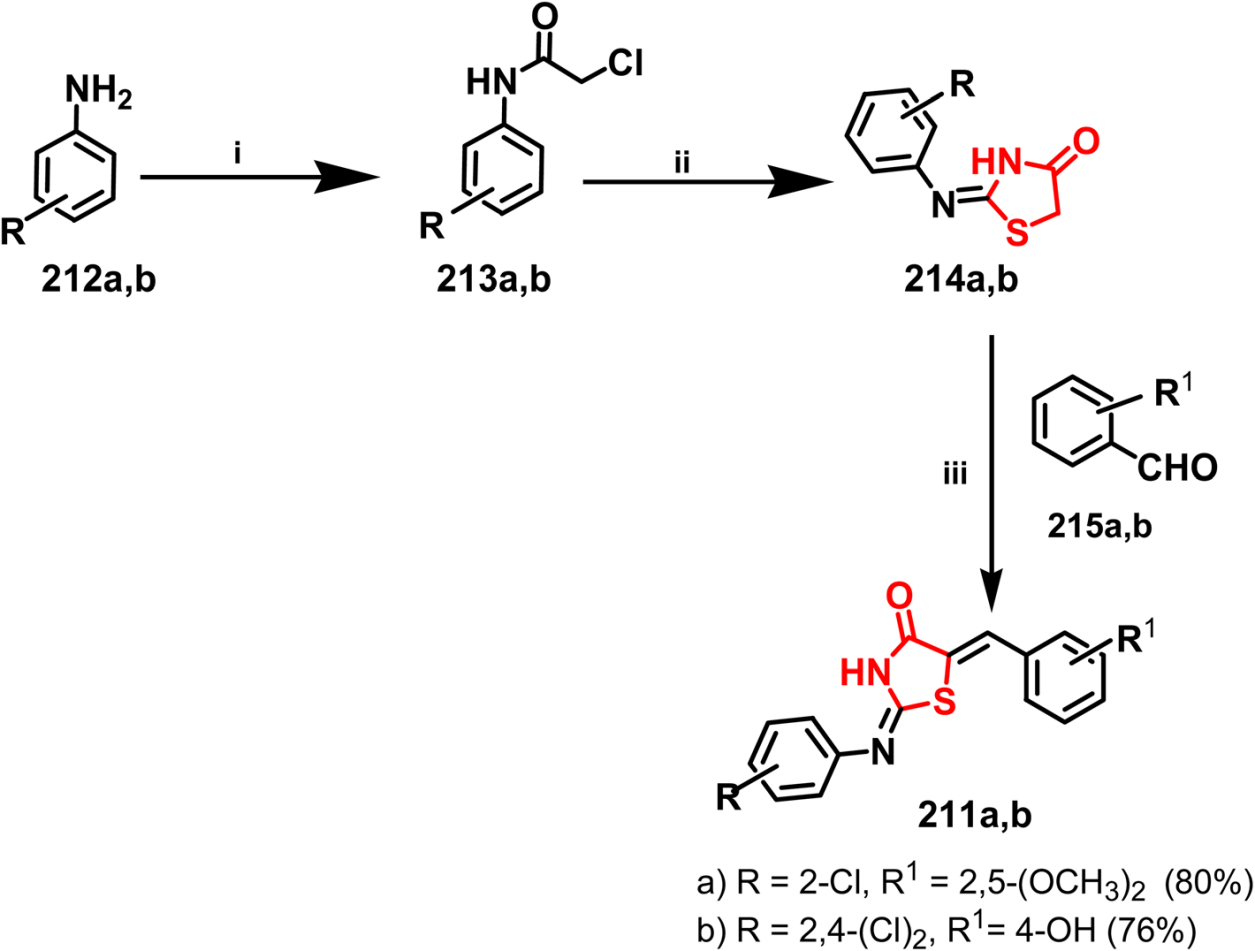
Synthesis of the thiazolidinone derivatives 211a, b. Reagents and conditions: (i) ClCH_2_COCl, acetone, stirring at RT, 6–8 h; (ii) NH4SCN, EtOH, reflux, 5–9 h; (iii) gl.AcOH, sodium acetate, reflux, 10–12 h.

The preparation of thiazolidinone derivatives 211a, b was carried out as described in Scheme 48.^113^

**Fig. 48:**
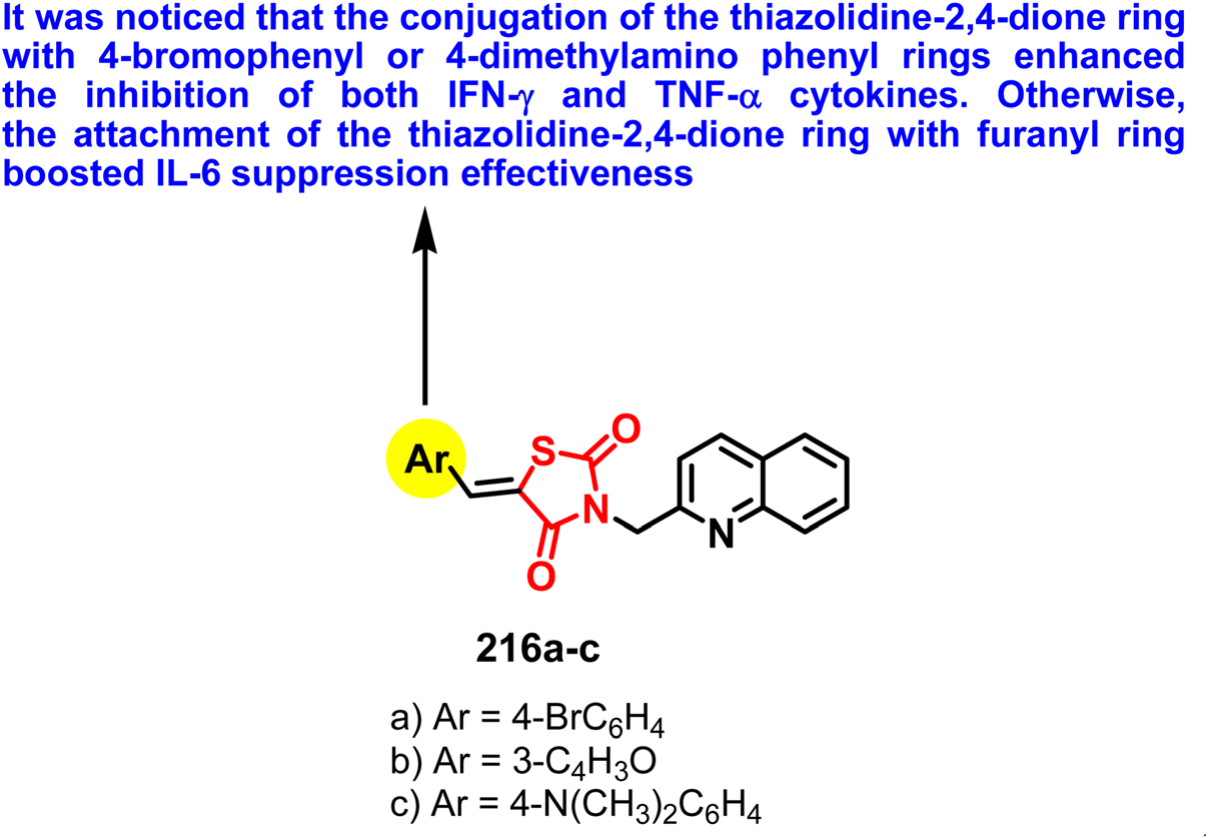
Structures of thiazolidinedione-quinoline hybrids 216a–c as anti-inflammatory agents targeting the COX-2 enzyme and various inflammatory mediators.

New thiazolidinedione-quinoline hybrids were synthesized and investigated for their suppression effects on the cytokines such as IFN-γ, TNF-α and IL-6. Derivatives 216a, c disclosed a remarkable decline in the concentrations of IFN-γ relative to cells stimulated with phytohemagglutinin (PHA). Moreover, derivatives 216a, c have a similar dose-dependent behavior in the inhibition of TNF-α, with a notable TNF-α reduction at a concentration of 100 µM, as compared to cells stimulated with PHA. Furthermore, only derivative 216b remarkably reduced IL-6 at a concentration of 50 µM. It was noticed that the conjugation of the thiazolidine-2,4-dione ring with 4-bromophenyl or 4-dimethylamino phenyl rings enhanced the inhibition of both IFN-γ and TNF-α cytokines. Otherwise, the attachment of the thiazolidine-2,4-dione ring with the furanyl ring boosted IL-6 suppression effectiveness. Moreover, derivatives 216a–c displayed good water solubility values of −5.522, −4.09 and −5.353 log mol L^−1^, respectively, and good intestinal absorption (90.13, 91.59 and 92.99%, respectively); in addition, all analyzed derivatives are substrates of the CYP3A4 enzyme. Only derivatives 216b, c are renal OCT2 substrates, suggesting possible renal elimination for these candidates, where all derivatives follow Lipinski's Rule of 5, in which MW < 500, log *P* < 5 (except ZKD-02), H-bond acceptors < 10, and H-bond donors <5. Derivatives demonstrated good affinity with the COX-2 enzyme, derivatives 216a, b created two H-bonds between the carbonyl atom of the thiazolidinedione ring and the N atom of quinoline with Arg106. Additionally, derivative 216a revealed an interaction between Gln178 and the halogen Br, while derivative 216c demonstrated Pi-cation interaction with Arg106. Moreover, derivatives 216a–c are engaged in hydrophobic interactions between quinoline rings with Val102, Leu78 and Tyr341 (ref. 114) (Fig. 48).

**Scheme 49:**
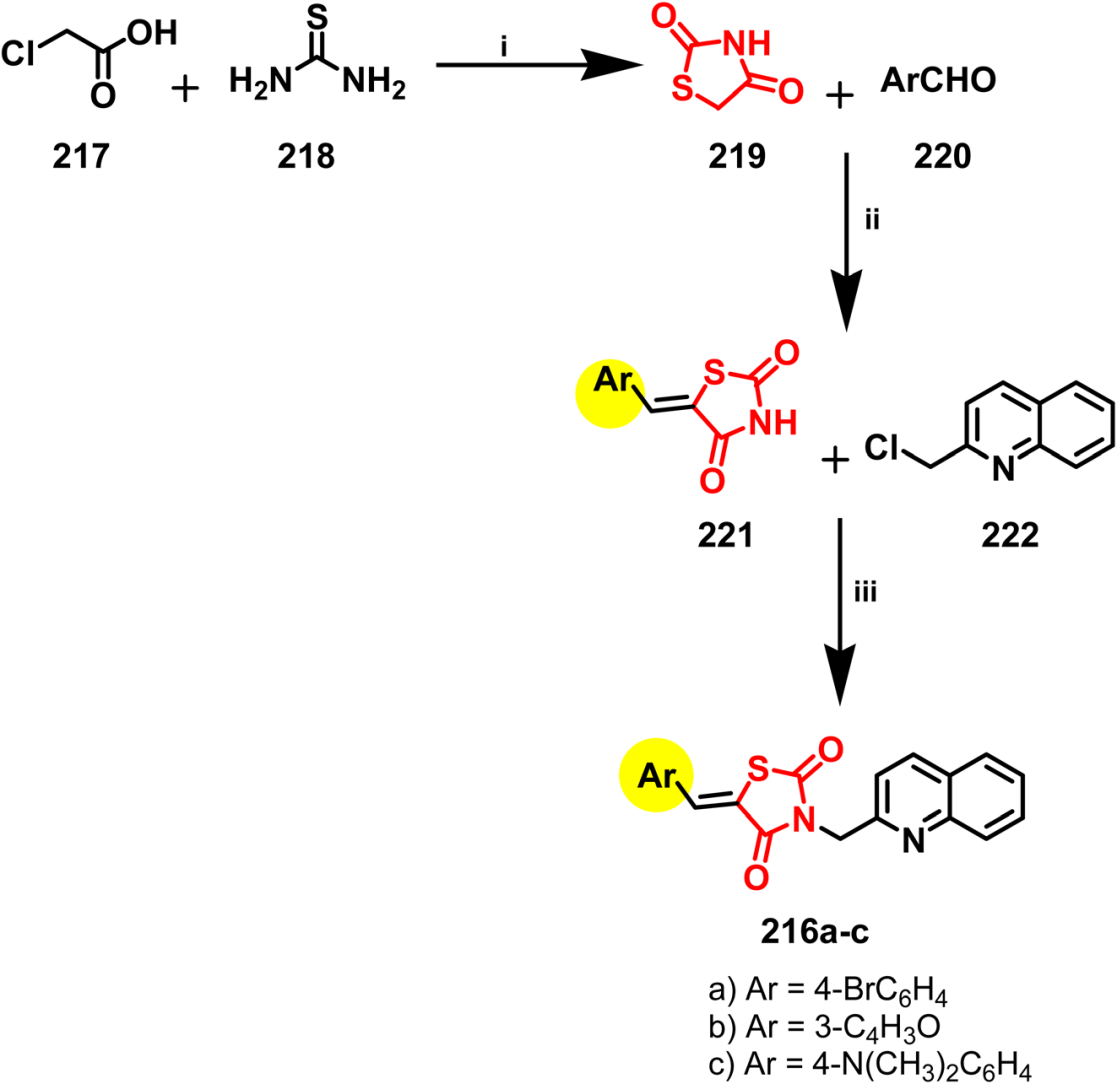
Synthesis of the thiazolidinedione-quinoline hybrids 216a–c. Reagents and conditions: (i) H_2_O, reflux, 90 °C, 24 h; (ii) AcOH, CH_3_COONH_4_, reflux, 110 °C, 5–8 h; (iii) EtOH, NaOH, reflux, 70 °C.

The new thiazolidinedione-quinoline hybrids 216a–c were prepared as described in Scheme 49.^114^

**Fig. 49:**
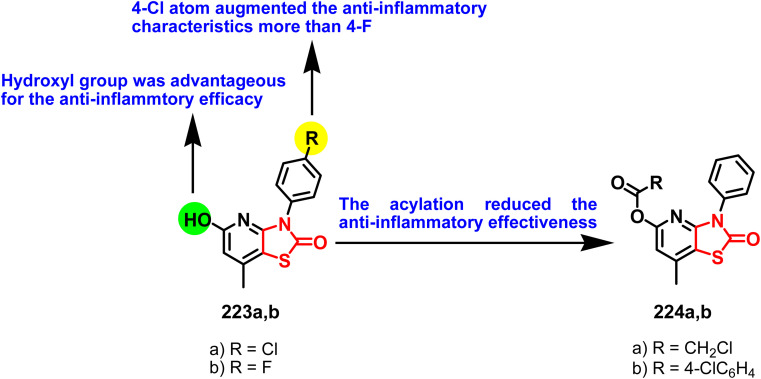
Structures of thiazolo[4,5-*b*]pyridin-2-ones 223a–c and 224a, b as anti-inflammatory agents.

New thiazolo[4,5-*b*]pyridin-2-ones 223a, b and 224a, b emerged as promising anti-inflammatory candidates (edema inhibition = 51.8%, 48.3%, 43.5% and 40.5%, respectively), overriding that of ibuprofen (40.2%). It could be concluded that the substitution of the phenyl ring connected to the thiazolone ring with a 4-Cl atom augmented the anti-inflammatory characteristics more than 4-F. Furthermore, the presence of a hydroxyl group at the 5th position of the thiazolo[4,5-*b*]pyridin-2-one scaffold was advantageous for the anti-inflammatory efficacy. Conversely, acylation reduced the anti-inflammatory effectiveness^115^ (Fig. 49).

**Scheme 50:**
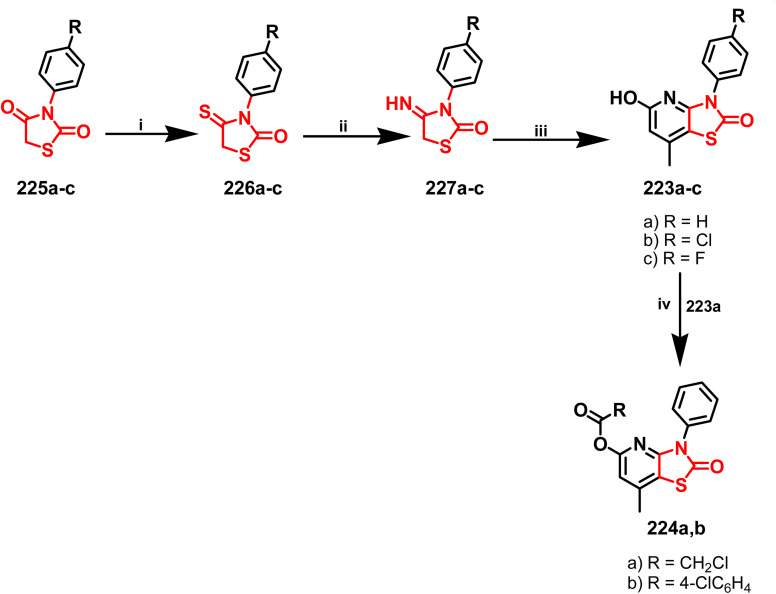
Synthesis of the thiazolo[4,5-*b*]pyridin-2-ones 223a–c and 224a, b. Reagents and conditions: (i) P_2_S_5_, toluene, reflux, 2 h; (ii) 25% aqueous ammonia solution, reflux, 15 min; (iii) CH_3_ONa, CH_3_COCH_2_COOC_2_H_5_, stirring, 20 °C; (iv) (a) acetyl chloride, dioxane, TEA, reflux for derivative a (b) 4-chlorobenzoyl chloride, pyridine, reflux for derivative b.

New thiazolo[4,5-*b*]pyridin-2-ones 223a–c and 224a, b were accomplished as illustrated in Scheme 50.^115^

**Fig. 50:**
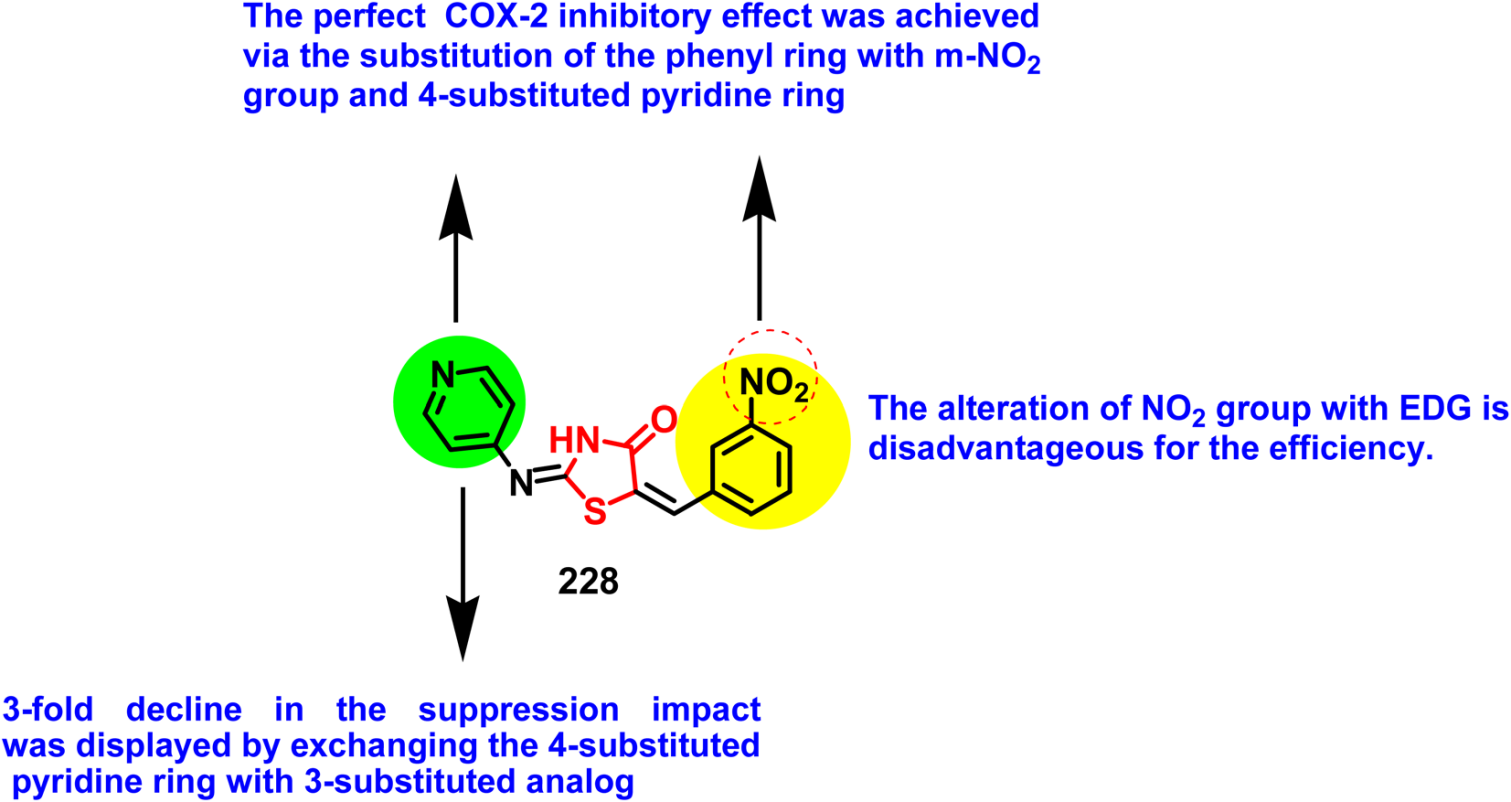
Structure of thiazolidinone-pyridine hybrid 228 as an anti-inflammatory agent targeting the COX-2 enzyme and various inflammatory mediators.

New thiazolidinone-pyridine hybrid 228 was displayed as a prominent COX-2 inhibitor (IC_50_ = 17.32 µM). The type and the position of the substituents on the phenyl ring connected to the thiazolidinone core as well as the position of the iminothiazolidinone moiety on the pyridine ring affected the COX-2 inhibitory efficacy. The perfect COX-2 inhibitory effect was achieved *via* the substitution of the phenyl ring with the *m*-NO_2_ group and the 4-substituted pyridine ring. However, a 3-fold decline in the suppression impact was displayed by exchanging the 4-substituted pyridine ring with a 3-substituted analog. Additionally, the alteration of the NO_2_ group with EDG is disadvantageous for the efficiency. Depending on the albumin denaturation technique, derivative 228 presented a good anti-inflammatory effect (inhibition of denaturation = 69.3%). In the *in vivo* investigation, at a dose of 10 mg kg^−1^, derivative 228 had a promising edema inhibition of 59.70% relative to that of celecoxib which is 67.50%. Moreover, pretreatment with 228 resulted in a dose-dependent reduction in COX-2 expression in RAW 264.7 cells, as demonstrated by fluorescence microscopy and flow cytometry. Moreover, it alleviated the expression of extracellular IL-6 and TNF-α. Furthermore, derivative 228 complied with the Lipinski rule of five. The values of log S of −3.81 and log *P* of 1.24 were within the optimal ranges, as well, and it disclosed an absorption value of 65.71%. The anticipating data suggested little possibility of influencing cardiac action potentials and moderate likelihood of hERG suppression. An LD_50_ value of 1400 mg kg^−1^ indicates that the oral administration is safe, and this thorough toxicity analysis verifies the analog’ safety for more study^116^ (Fig. 50).

**Scheme 51:**
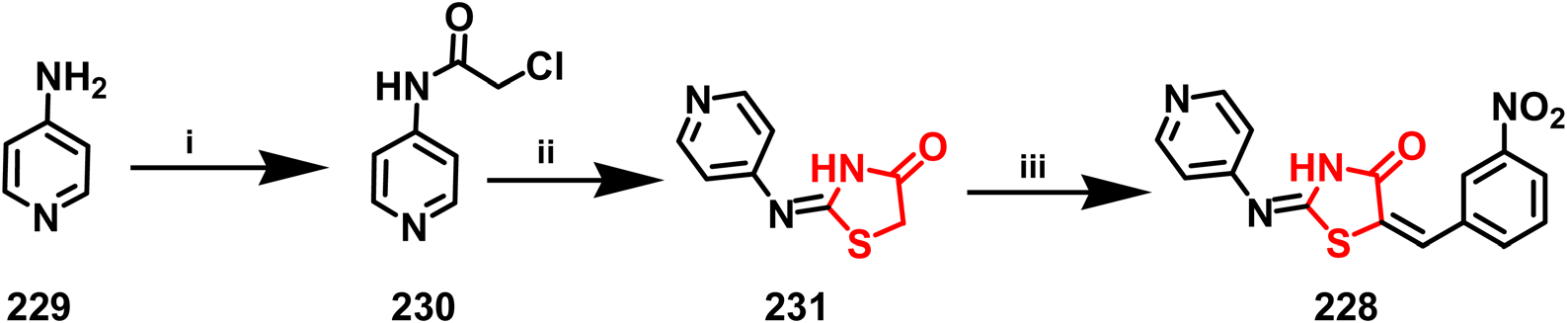
Synthesis of the thiazolidinone-pyridine hybrid 228. Reagents and conditions: (i) ClCH_2_COCl, acetone, stirring at RT, 3 h; (ii) NH_4_SCN, EtOH, reflux, 8 h; (iii) 3-nitrobenzaldehyde, gl.AcOH, sodium acetate, reflux, 15 h.

New thiazolidinone-pyridine hybrid 228 was prepared as depicted in Scheme 51.^116^

### 2.11 Benzothiazole-based derivatives as anti-inflammatory agents

**Fig. 51:**
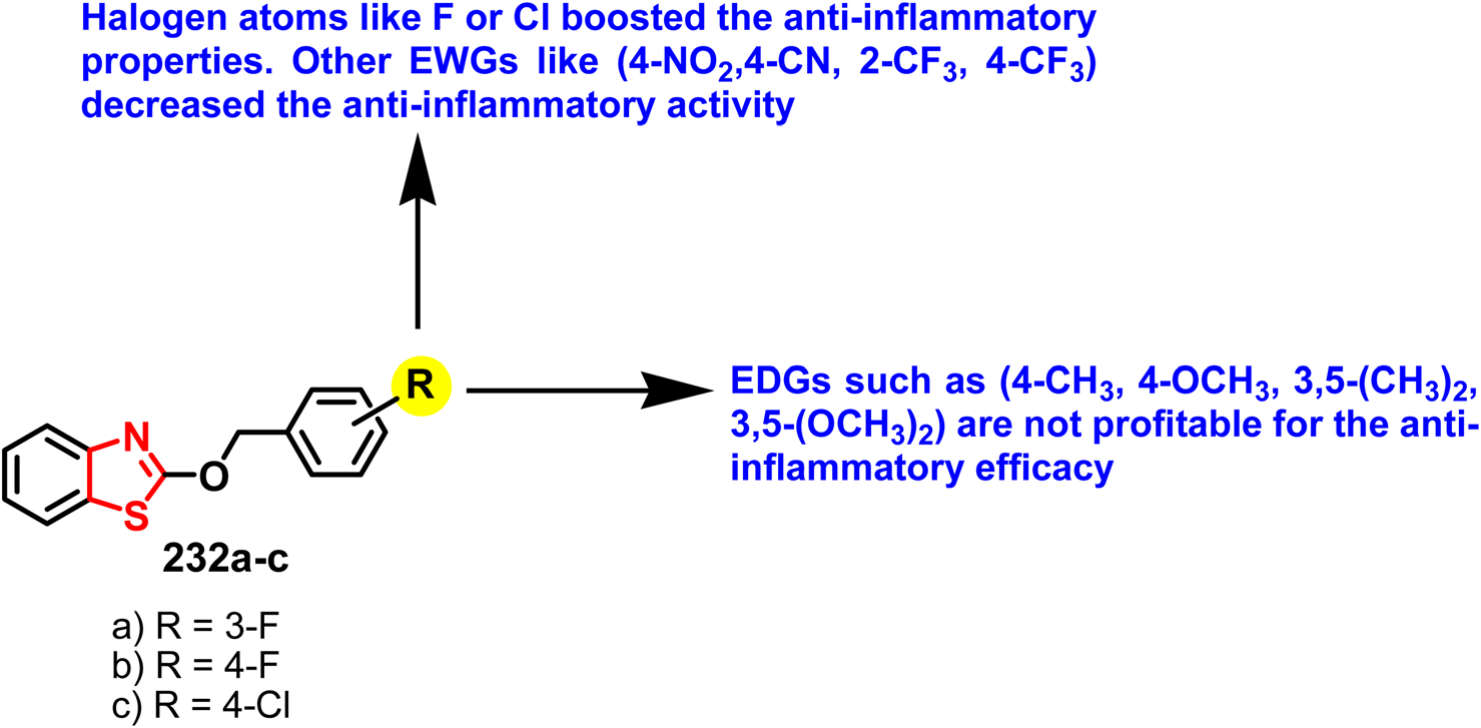
Structures of benzothiazole analogs 232a–c as anti-inflammatory agents targeting the COX-2 enzyme.

Hea *et al.*^117^ illustrated the synthesis and the anti-inflammatory investigation of novel benzothiazole analogs employing an ear edema model. Derivatives 232a–c stood out as the best anti-inflammatory candidates (edema inhibition = 86.8%, 90.7% and 82.9%, respectively) comparable to that of indomethacin (edema inhibition = 82.4%). It was concluded that the substitution of the benzyloxy moiety with EWGs, particularly halogen atoms such as F or Cl, boosted the anti-inflammatory properties. However, the anti-inflammatory efficiency was alleviated by the appearance of other EWGs such as 4-NO_2_, 4-CN, 2-CF_3_, and 4-CF_3_ on the benzyloxy moiety. Additionally, the substitution of the benzyloxy moiety with EDG such as 4-CH_3_, 4-OCH_3_, 3,5-(CH_3_)_2_, and 3,5-(OCH_3_)_2_ is not profitable for the anti-inflammatory efficacy. Furthermore, derivatives 232a–c demonstrated a modest COX-2 suppression effectiveness (IC_50_ = 0.28, 0.37 and 0.77 µM and SI = 18.6, 16.8 and 7.2, respectively) with respect to celecoxib (IC_50_ = 0.27 µM, SI = 19.7). However, they manifested a weak COX-1 inhibitory efficiency (IC_50_ = 5.22, 6.20 and 5.55 µM, respectively) (Fig. 51).

**Scheme 52:**
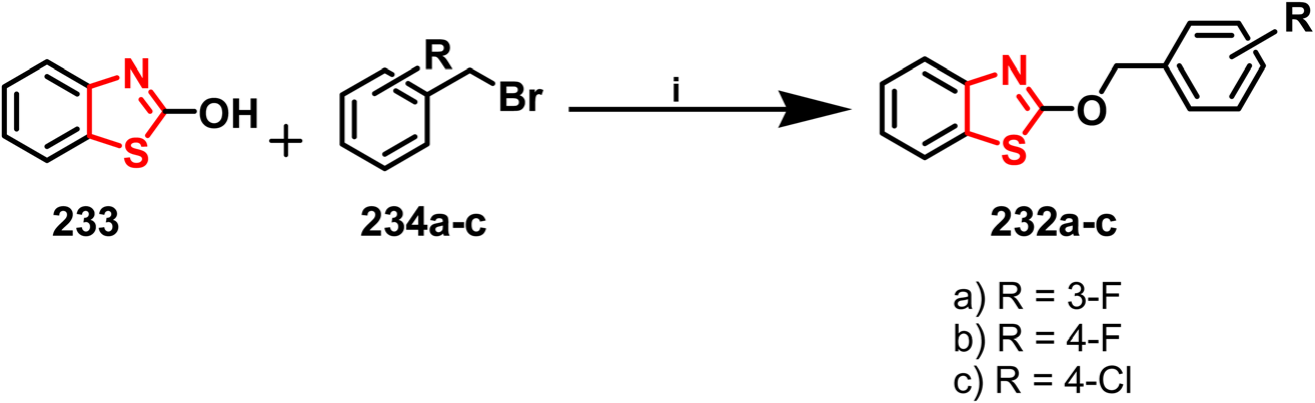
Synthesis of the benzothiazole analogs 232a–c. Reagents and conditions: (i) K_2_CO_3_, acetone, reflux.

Novel benzothiazole analogs 232a–c were prepared as depicted in Scheme 52.^117^

**Fig. 52:**
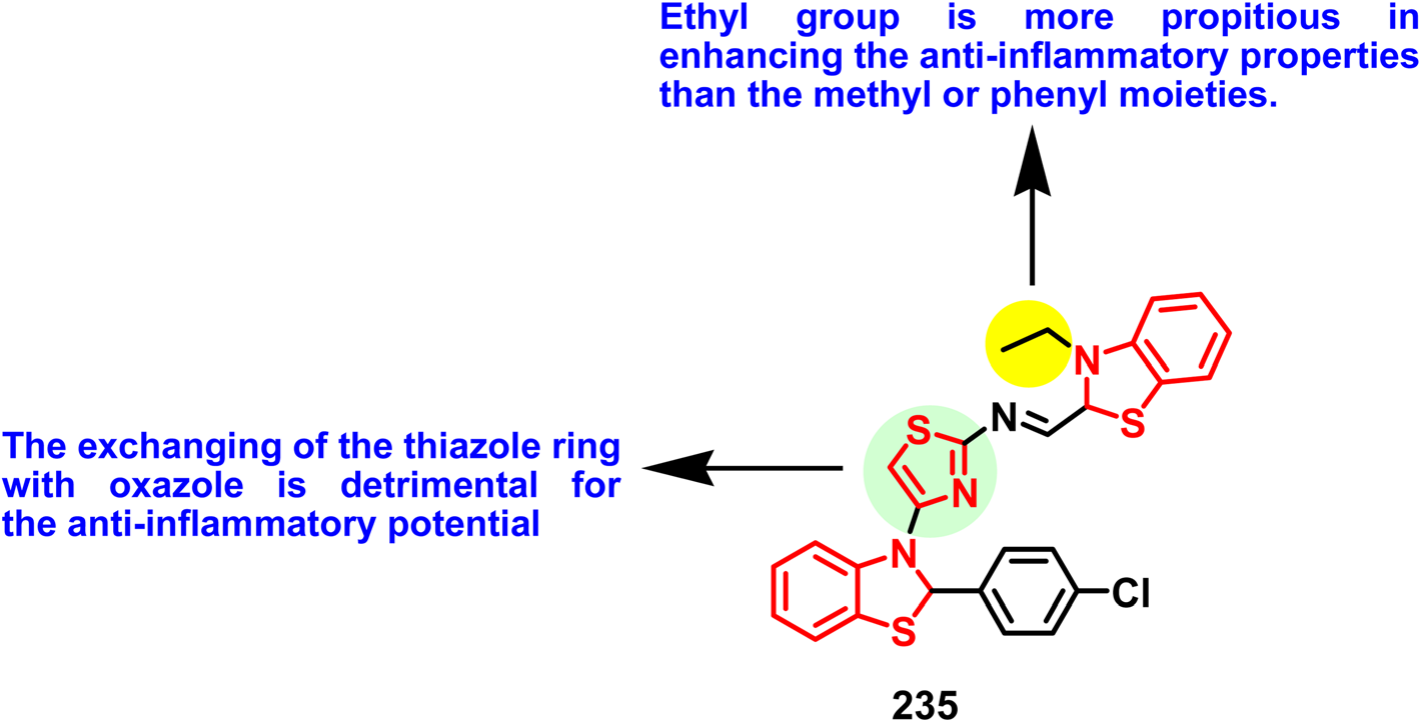
Structure of the bis-benzothiazole scaffold carrying thiazole 235 as an anti-inflammatory agent.

At dosages of 25, 50, and 100 mg kg^−1^ p.o., a new bis benzothiazole scaffold carrying thiazole ring 235 stood out as an eminent anti-inflammatory candidate (edema inhibition = 38.5%, 55.4%, and 69.6%, respectively), overriding that of phenyl butazone (edema inhibition = 22.2%, 35.8% and 66.5%, respectively). It was pointed out that the substitution of the benzothiazole scaffold with the ethyl group is more propitious in enhancing the anti-inflammatory properties than the methyl or phenyl moieties. In addition, the unsubstituted benzothiazole attenuated the anti-inflammatory efficacy. Moreover, the exchange of the thiazole ring with oxazole is detrimental to the anti-inflammatory potential. Interestingly, derivative 235 displayed less ulcerogenic effects (UD_50_ = 245 mg kg^−1^ i.p.) than phenyl butazone (UD_50_ = 66.6 mg kg^−1^ i.p.); however, it had ALD_50_ > 1500 mg kg^−1^ p.o.^118^ (Fig. 52).

**Scheme 53:**
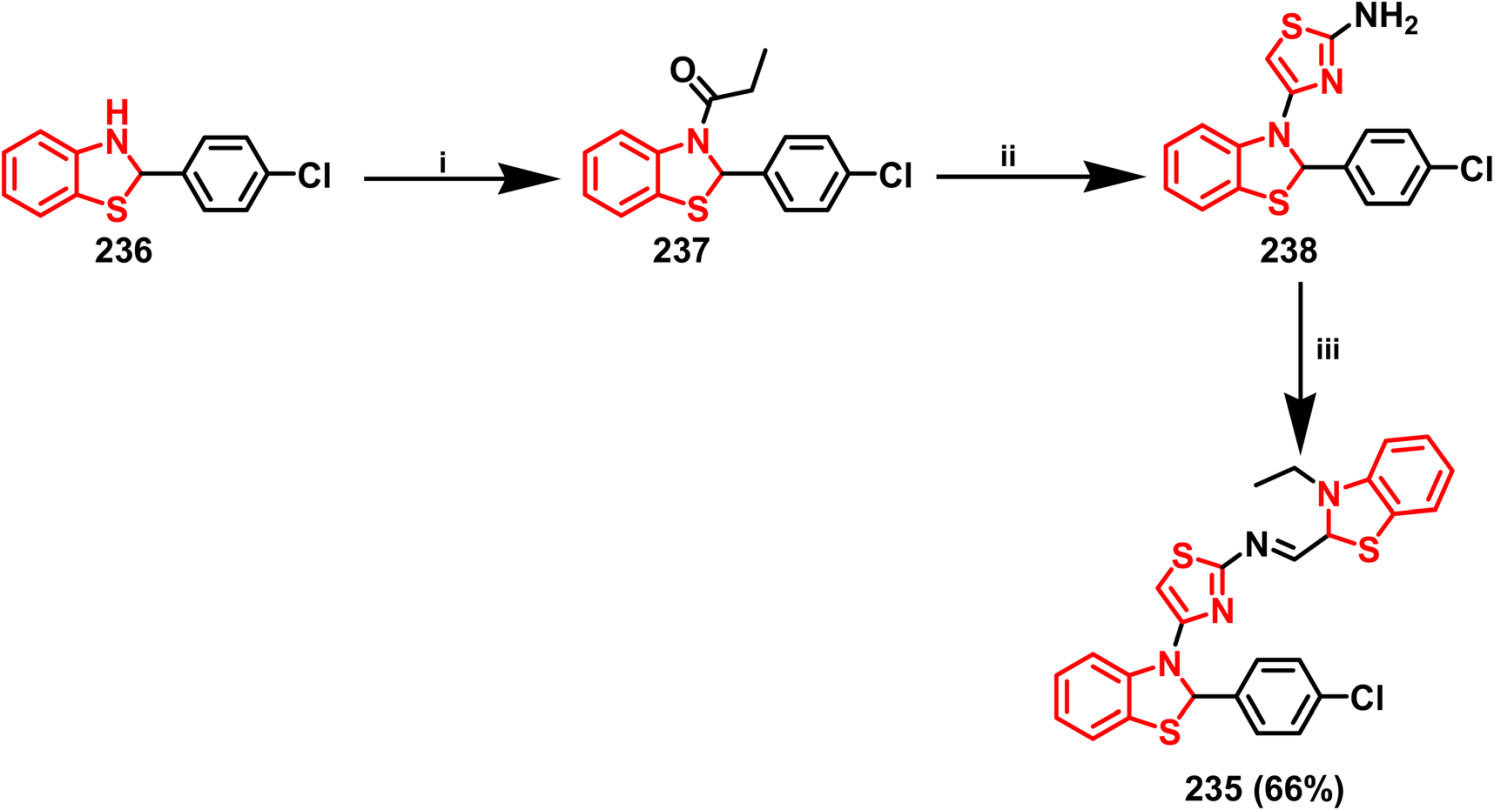
Synthesis of the bis-benzothiazole scaffold carrying thiazole 235. Reagents and conditions: (i) ClCH_2_COCl, acetone, stirring at RT, 3 h; (ii) NH_2_CSNH_2_, EtOH, reflux, 8 h; (iii) 3-ethyl-2-hydrobenzo[*d*]thiazole-2-carbaldehyde, gl.AcOH, reflux, 10 h.

A new bis-benzothiazole scaffold carrying thiazole ring 235 was prepared as described in Scheme 53.^118^

**Fig. 53:**
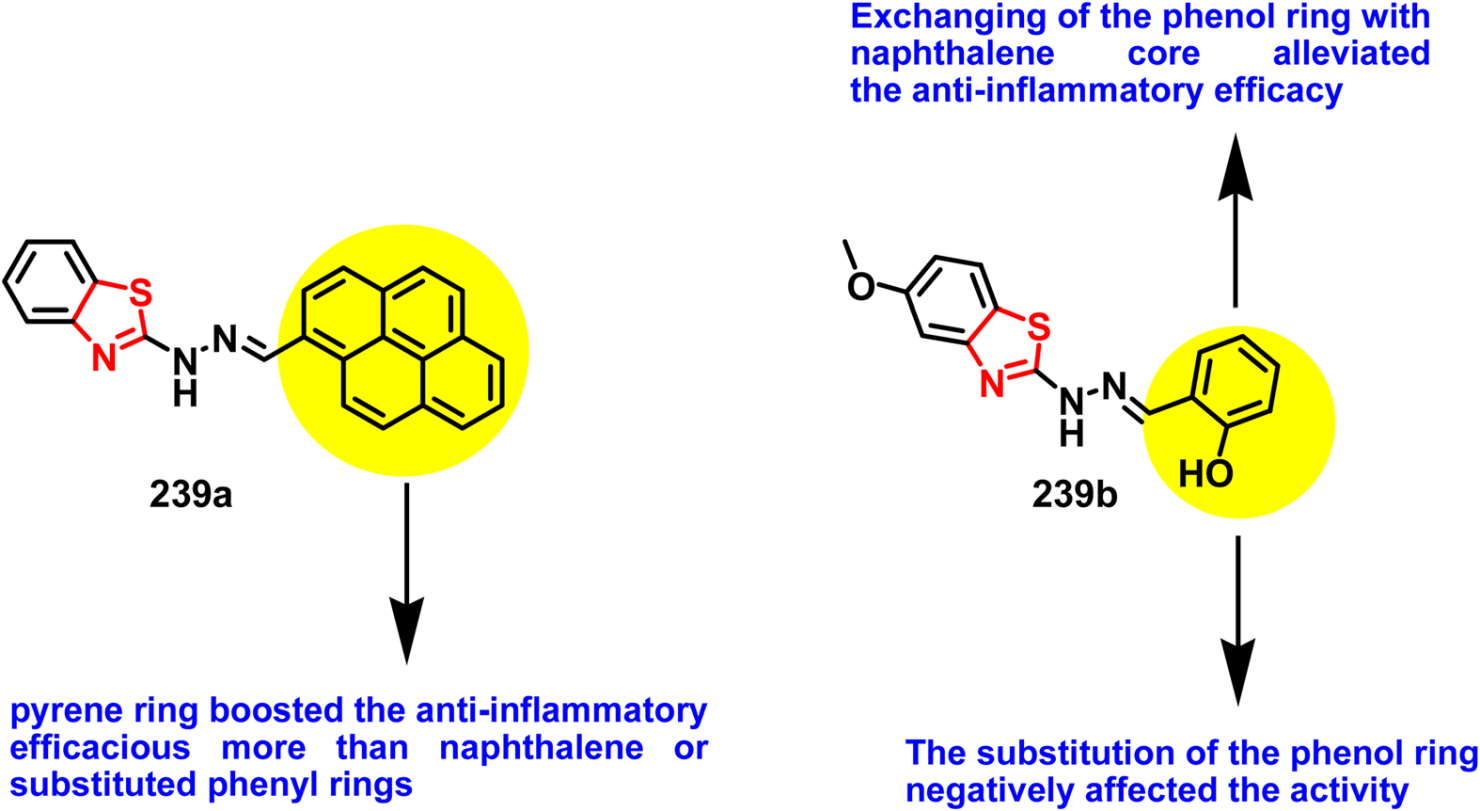
Structures of benzothiazole-hydrazone analogs 239a, b as anti-inflammatory agents targeting the COX-2 enzyme.

New benzothiazole-hydrazone analogs 239a, b were synthesized and estimated for their anti-inflammatory effects utilizing two distinct methods. The benzothiazole-hydrazone 239a demonstrated prominent anti-inflammatory properties for the denaturation of bovine serum albumin (IC_50_ = 26.99 µg mL^−1^), whereas derivative 239b had the highest *in vitro* efficacy in the egg serum albumin technique (IC_50_ = 26.69 µg mL^−1^), reflecting outstanding anti-inflammatory effectiveness. It has been noted that the conjugation of benzothiazole with the pyrene ring boosted the anti-inflammatory efficacy more than naphthalene or substituted phenyl rings. Additionally, the engagement of the 6-methoxy benzothiazole scaffold with the phenol ring presented a notable anti-inflammatory effect. Otherwise, the substitution of the phenol ring negatively affected the activity, and also, exchanging the phenol ring with the naphthalene core alleviated the anti-inflammatory efficacy. Moreover, derivatives 239a, b complied with the Lipinski rule of five and other critical metrics including log *S*, *c* log *P* and CYP3A4, all of which are required for the molecules to function as therapeutic candidates, thus both derivatives presented favorable drug-likeness characteristics. The most effective anti-inflammatory candidates 239a, b displayed docking scores of – 9.2 and – 9.6 kcal mol^−1^, respectively, within COX-2. Deep within the COX-2 active site's inner core, derivative 239b's docking pose revealed connections with Glu364, Trp545 and Arg109 through hydrogen bonding. However, derivative 239a interacted with Lys342 and Asn560 (ref. 119) (Fig. 53).

**Scheme 54:**
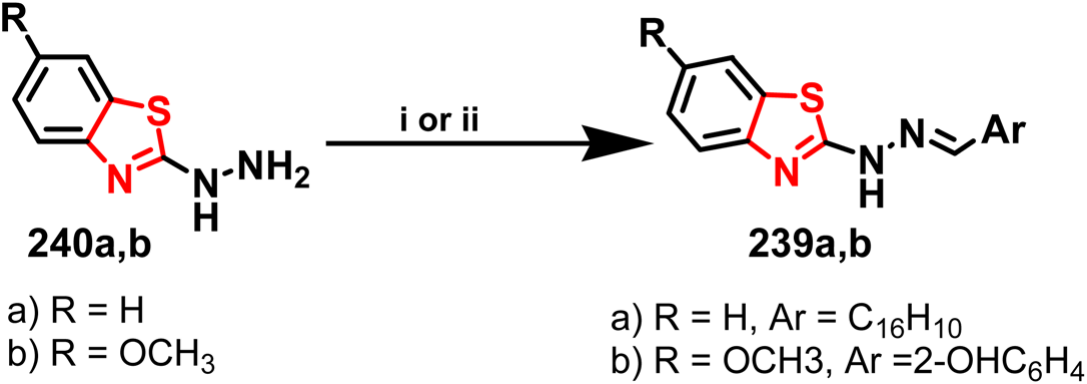
Synthesis of the benzothiazole-hydrazone analogs 239a, b. Reagents and conditions: (i) R_1_CHO, EtOH, AcOH, reflux, 8–10 h; (ii) R_1_CHO, MgZrO_3_@SO_4_^2−^, EtOH, reflux, 60 min.

Using two distinct techniques, as illustrated in Scheme 54, the final benzothiazole hydrazones 239a, b were produced by reacting compounds 240a, b with various aldehydes: one with a heterogenous MgZrO_3_@SO_4_^2−^ solid acid catalyst and another with a homogeneous acetic acid catalyst (Scheme 54).^119^

**Fig. 54:**
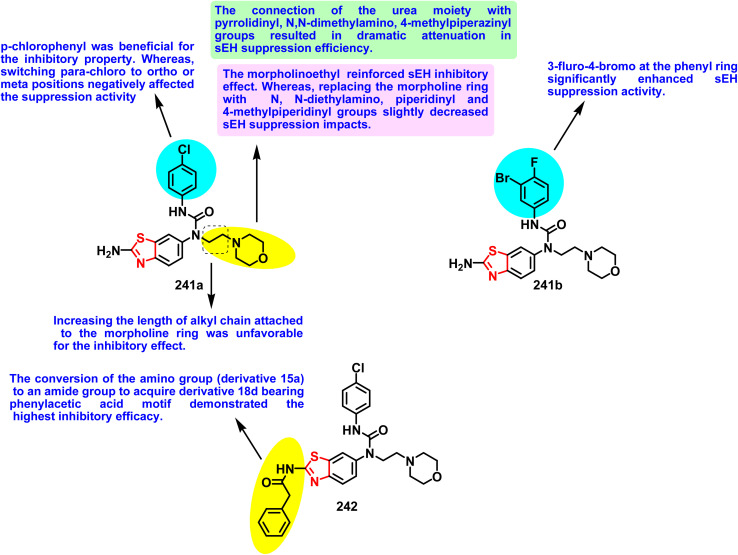
Structures of benzothiazole derivatives 241a, b and 242 as anti-inflammatory agents targeting the sEH enzyme.

In 2021, Han *et al.*^120^ identified benzothiazole derivatives 241a, b and 242 as prominent soluble epoxide hydrolase (sEH) inhibitors (IC_50_ = 2.8, 0.25 and 0.082 nM, respectively) surpassing that of *t*-AUCB (IC_50_ = 6.7 nM). It was seen that the attachment of the urea moiety with the morpholinoethyl reinforced sEH inhibitory effects, whereas replacing the morpholine ring with *N*,*N*-diethylamino, piperidinyl and 4-methylpiperidinyl groups slightly decreased sEH suppression effects. Otherwise, the connection of the urea moiety with pyrrolidinyl, *N*,*N*-dimethylamino, and 4-methylpiperazinyl groups resulted in dramatic attenuation in the sEH suppression efficiency. Additionally, increasing the length of alkyl chain attached to the morpholine ring was unfavorable for the inhibitory effect. The attachment of the urea moiety with *p*-chlorophenyl was beneficial for the inhibitory property, whereas switching *para*-chloro to *ortho* or *meta* positions negatively affected the suppression activity. Remarkably, derivative 241b with 3-fluro-4-bromo at the phenyl ring significantly enhanced sEH suppression activity. Interestingly, the conversion of the amino group (derivative 241a) to an amide group to acquire derivative 242 bearing a phenylacetic acid motif demonstrated the highest inhibitory efficacy. Moreover, derivatives 241a and 242 demonstrated more efficient *in vivo* anti-inflammatory effects (edema inhibition = 23.39% and 20.32%, respectively) than *t*-AUCB (edema inhibition = 24.71%). In addition, derivatives 241a, b and 242 presented low cytotoxicity (IC_50_ values > 25 µM) against HepG-2 cells. Compound 241b had an intrinsic liver clearance of 55.8 mL min mg^−1^ and was more stable than compound 241a, which had an intrinsic liver clearance of 71.9 mL min mg^−1^, according to research on rat liver microsomes. Nevertheless, compound 242 was very unstable in rat liver microsomes, as evidenced by its half-life of 1.7 minutes and an intrinsic liver clearance of 1476.7 mL min mg^−1^. Additionally, derivative 241b demonstrated moderate metabolism in rat liver microsomes and acceptable stability with a half-life of 44.7 minutes. The binding interactions of derivative 241a to sEH detected the formation of three H-bonds between Trp525, Asp335 and Tyr466 with a S atom of 2-aminobenzothiazole, a N atom of 2-ethylmorpholine moiety and O of the carbonyl group, respectively. Otherwise, the interactions of derivative 242 to sEH displayed that the terminal phenylacetyl group dovetailed in the hydrophobic pocket. In addition, three H-bonds were revealed between the N atom of the 2-ethylmorpholine moiety, the O atom of amide and the O atom of urea with Trp525, Ser415 and Tyr466, respectively (Fig. 54).

**Scheme 55:**
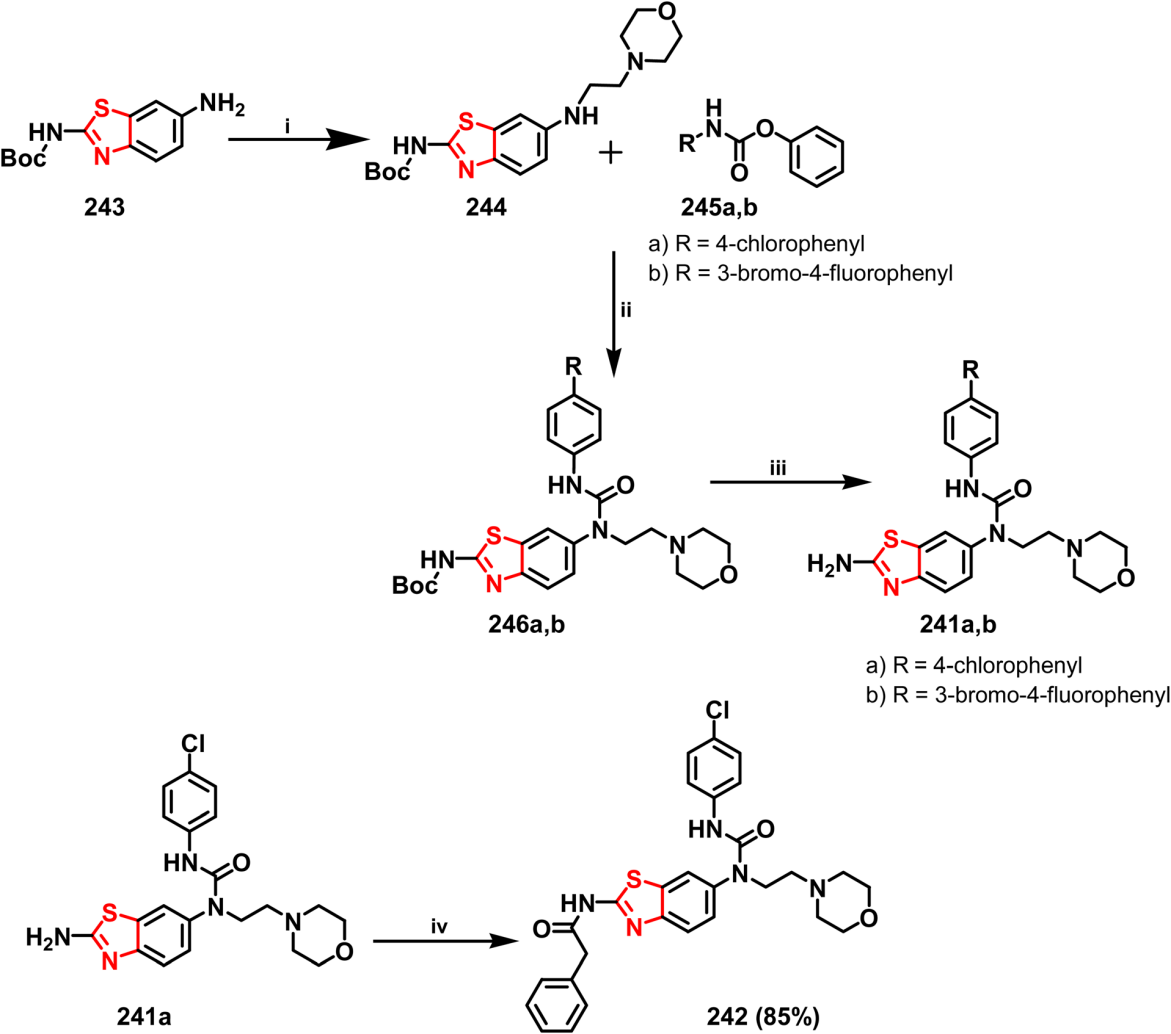
Synthesis of the benzothiazole derivatives 241a, b and 242. Reagents and conditions: (i) 4-(2-chloroethyl)morpholine, K_2_CO_3_, 1,4-dioxane, 90 °C, 12 h; (ii) Et_3_N, 1,4-dioxane, 60 °C, 12 h; (iii) CF_3_COOH, DCM, rt, 3 h; (iv) C_6_H_5_COCl, K_2_CO_3_, EA, rt, 5 h.

The substitution reaction of derivative 243 with 4-(2-chloroethyl)morpholine in the presence of potassium carbonate furnished the corresponding derivative 244. Moreover, the substitution of derivative 244 with different substituent carbamates 245a, b afforded derivatives 246a, b, following by deprotection with trifluoroacetic acid that yielded the target derivatives 241a, b. Compound 242 was then generated through a condensation reaction of derivative 241a with benzoyl chloride (Scheme 55).^120^

**Fig. 55:**
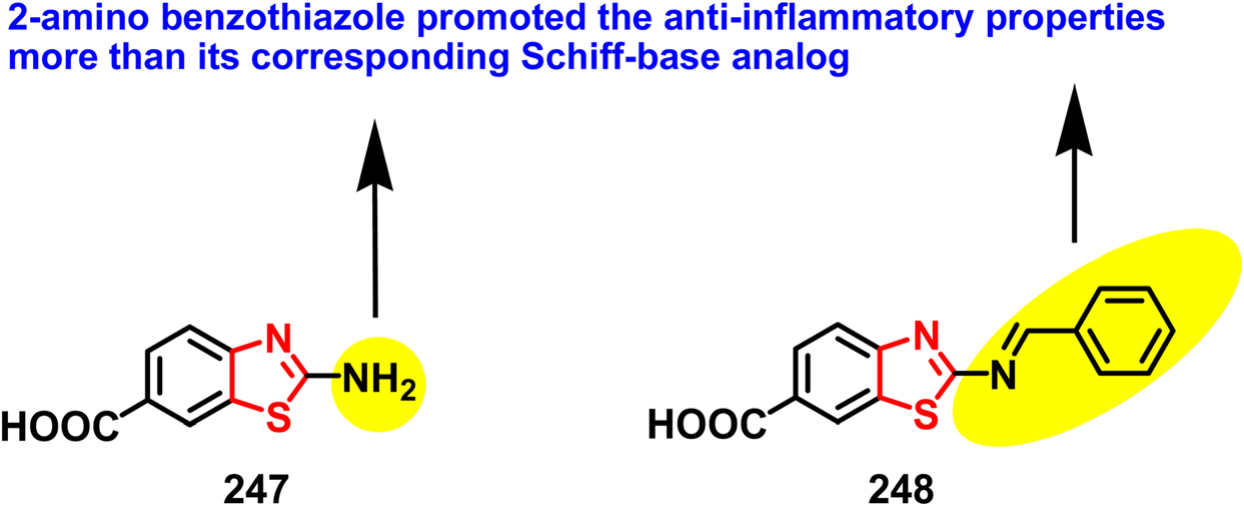
Structures of 2-amino benzothiazole 247 and its corresponding Schiff-base analog 248 as anti-inflammatory agents.

In 2022, 2-amino benzothiazole 247 and its corresponding Schiff-base analog 248 emerged as promising anti-inflammatory candidates with edema inhibition of 59.6% and 54.7%, respectively, comparable to that of nimesulide (edema inhibition = 59.64%) 2 h post administration. Fortunately, both derivatives possessed LD_50_ greater than 1500 mg kg^−1^ p.o. It was deduced that 2-amino benzothiazole 247 promoted the anti-inflammatory properties more than its corresponding Schiff-base analog 248 (ref. 121) (Fig. 55).

**Scheme 56:**
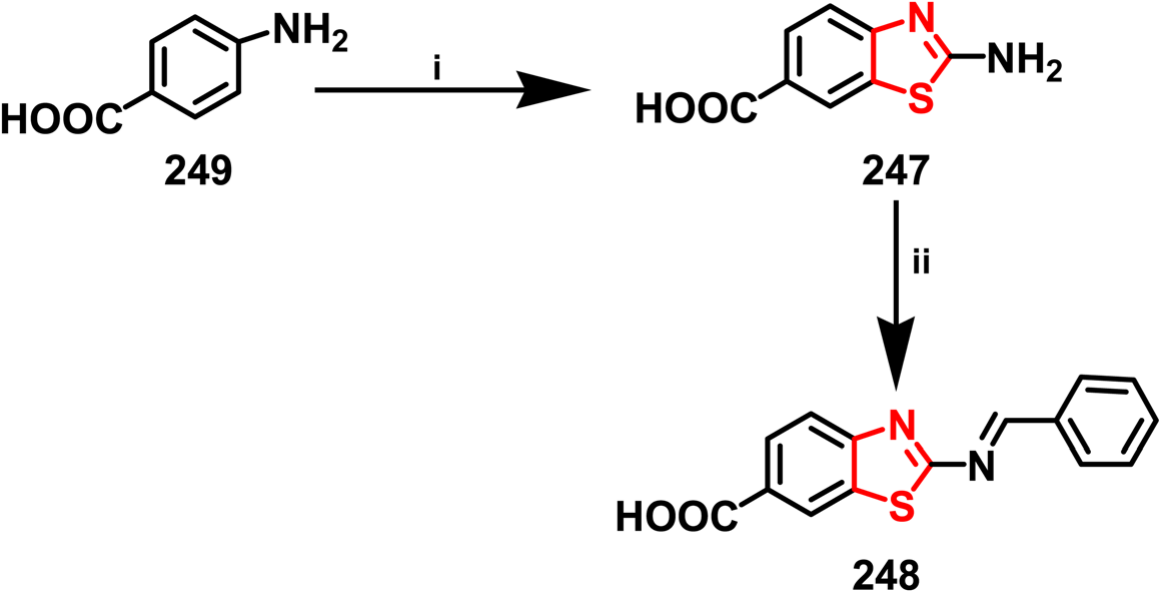
Synthesis of the 2-amino benzothiazole 247 and its corresponding Schiff-base analog 248. Reagents and condition: (i) KSCN, Br_2_, gl.AcOH, 0–5 °C, 2 h, neutralized with a conc ammonia solution; (ii) benzaldehyde, EtOH, anhydrous zinc chloride, reflux, 20 h.

The synthesis of 2-amino benzothiazole 247 and its corresponding Schiff-base analog 248 is illustrated in Scheme 56.^121^

**Fig. 56:**
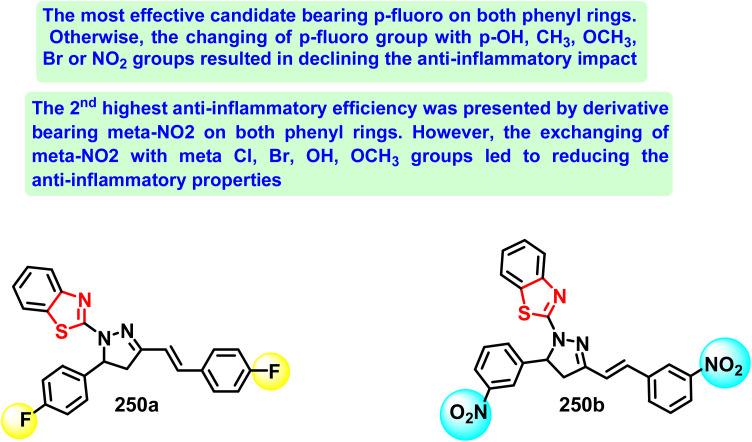
Structures of benzothiazole linked to dihydropyrazole derivatives 250a, b as anti-inflammatory agents targeting the COX-2 enzyme.

New benzothiazole linked to dihydropyrazole derivatives 250a, b emerged as outstanding anti-inflammatory candidates employing an egg albumin assay (IC_50_ = 0.03 and 0.05 µmol mL^−1^, respectively), exceeding that of ibuprofen (IC_50_ = 0.11 µmol mL^−1^). It was deduced that the nature and the position of the substituents on the two phenyl rings connected to the pyrazole ring have significant influence on the anti-inflammatory efficiency. The most effective candidate bears *p*-fluoro on both phenyl rings. Otherwise, the exchange of *p*-fluoro group with *p*-OH, CH_3_, OCH_3_, Br or NO_2_ groups resulted in a decline in the anti-inflammatory impact. The 2nd highest anti-inflammatory efficiency was presented by a derivative bearing *meta*-NO_2_ on both phenyl rings. However, the exchange of *meta*-NO_2_ with meta Cl, Br, OH, or OCH_3_ groups led to reduced anti-inflammatory properties. It was noticed that switching NO_2_ from the *meta* to the *para* position decreased the anti-inflammatory effect by nearly 2.6 folds. Additionally, derivative 250a demonstrated good binding affinity against the target protein PDB:6COX (−10.0 kcal mol^−1^). A H-bond was detected between the N atom of the pyrazoline ring and Tyr115. Moreover, Arg120 participated in pi-cation interaction with the phenyl ring of benzothiazole. In addition, pi-anion, pi-pi-T-shaped interactions as well as pi-sigma interaction were seen between the para fluoro-substituted phenyl rings with Glu524, Tyr122 and Leu123, respectively. However, pi-sigma and amide-pi-stacked interactions were established between the para fluorostyryl ring with Val89 and Leu82, respectively. Furthermore, Arg120, Val89, Lys83, Leu82, Lys79 and Tyr122 prompted pi-alkyl interactions with compound 250a. Derivative 250a complied with Lipinski's and Veber's rules, with the number of hydrogen bond donors = zero, and number of hydrogen bond acceptors = 3, whereas log *P* = 3.90 and TPSA = 28.49 Å^2^. The BBB penetration of derivative, Z13 = 0.46, which indicated moderate absorption to CN, presented a significant human intestinal absorption value of 97.87%. Furthermore, it displayed a prominent *in vitro* plasma protein binding of 96.94% (ref. 122) (Fig. 56).

**Scheme 57:**
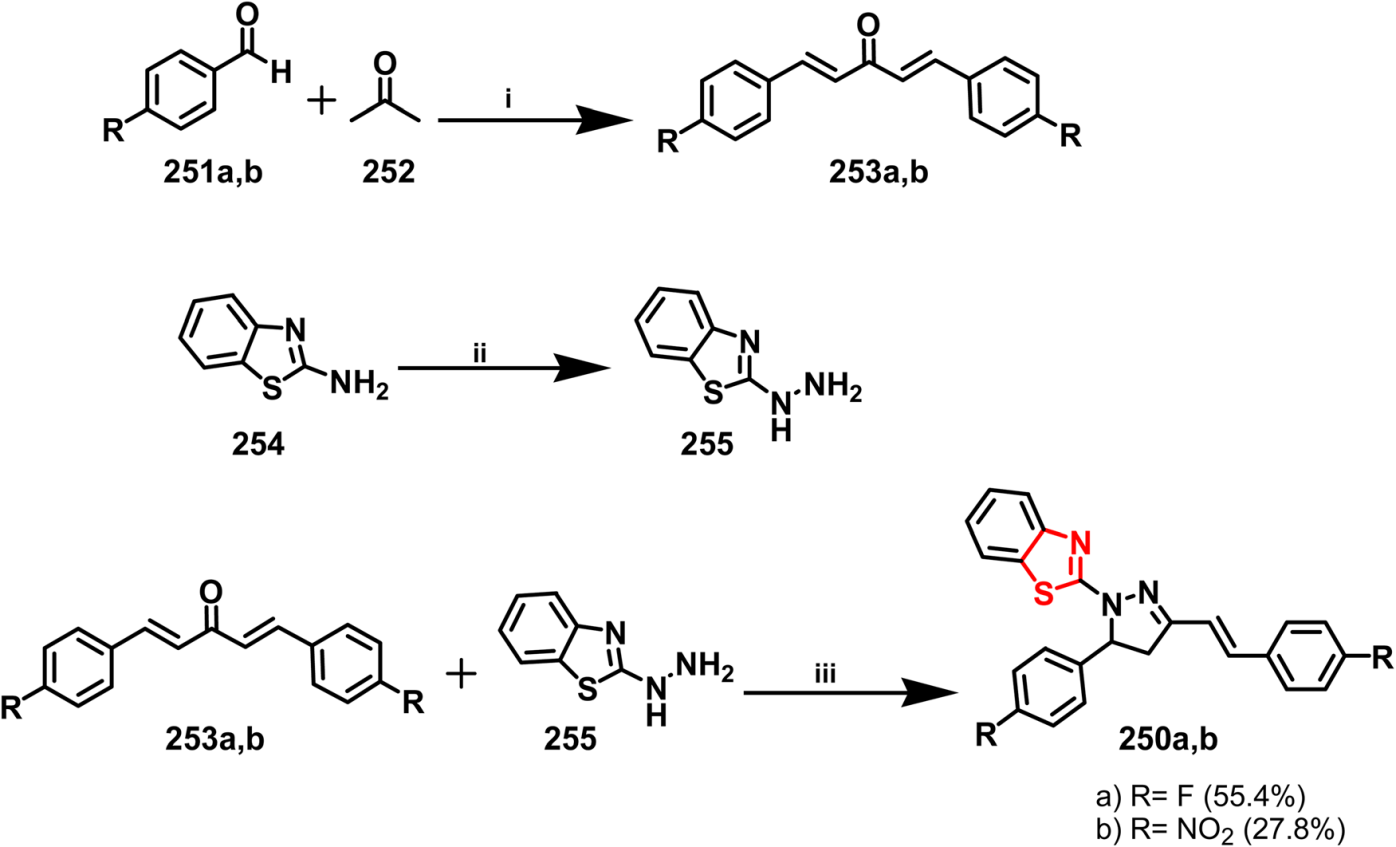
Synthesis of benzothiazole linked to dihydropyrazole derivatives 250a, b. Reagents and condition: (i) EtOH, NaOH, stirring, 1–4 °C, 1 h; (ii) NH_2_NH_2_·H_2_O, ethylene glycol, conc. HCl, reflux, 140 °C, 3 h; (iii) gl.AcOH, reflux.

The aldol condensation reaction of substituted benzaldehydes 251a, b and acetone 252 in an alkaline ethanolic solution furnished the intermediates 253a, b. Moreover,1-(benzo[*d*]thiazol-2-yl)hydrazine intermediate 255 was prepared by the reaction of benzothiazole amine 254 with hydrazine hydrate in the presence of ethylene glycol. Finally, the target derivatives 250a, b were achieved *via* the reaction of the two previous intermediates 253a, b and 255 in the presence of glacial acetic acid (Scheme 57).^122^

**Fig. 57:**
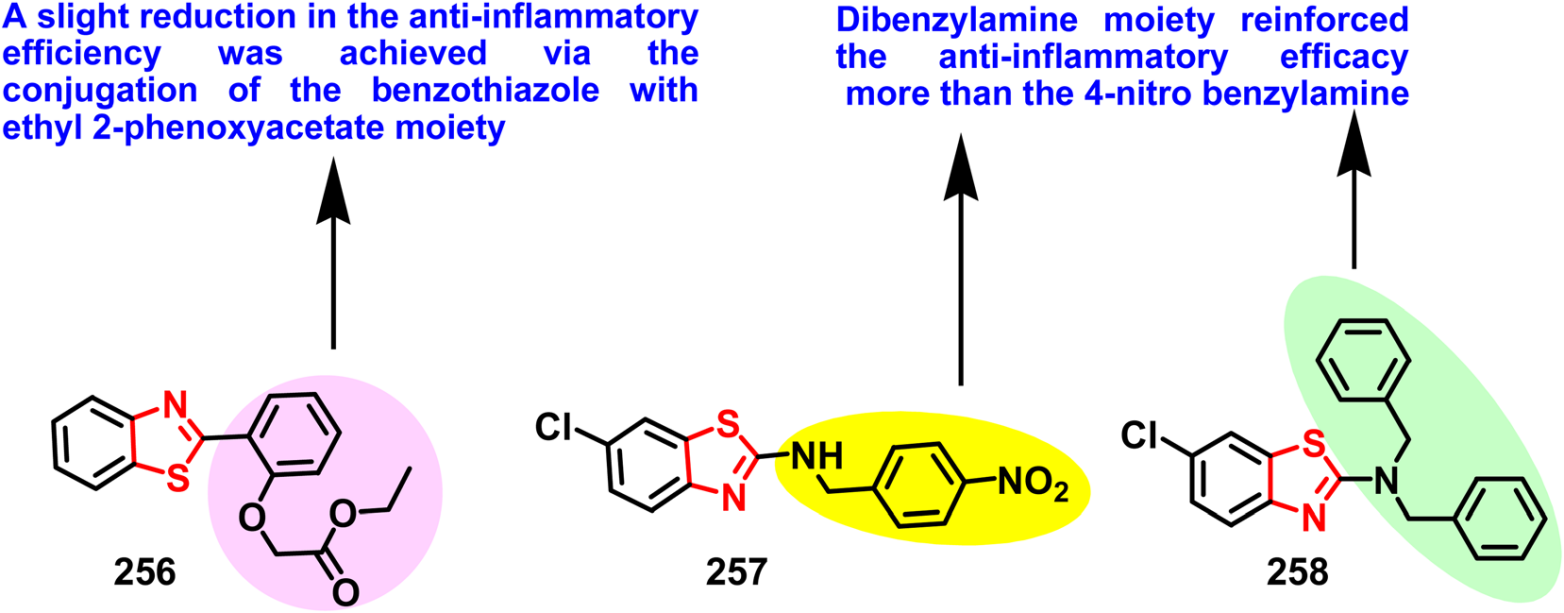
Structures of benzothiazole derivatives 256, 257, and 258 as anti-inflammatory agents targeting various inflammatory mediators.

New benzothiazole derivatives 256, 257, and 258 presented notable inhibitory effects on TNF-α and IL-6 production at 10 µM, implying effective anti-inflammatory activity. In particular, 258 demonstrated the most effective results, followed by 257 and 256. It could be deduced that the attachment of the 6-chlorobenzothiazole scaffold with a dibenzylamine moiety reinforced the anti-inflammatory efficacy more than 4-nitro benzylamine. However, a slight reduction in the anti-inflammatory efficiency was achieved *via* the conjugation of benzothiazole with an ethyl 2-phenoxyacetate moiety^123^ (Fig. 57).

**Scheme 58:**
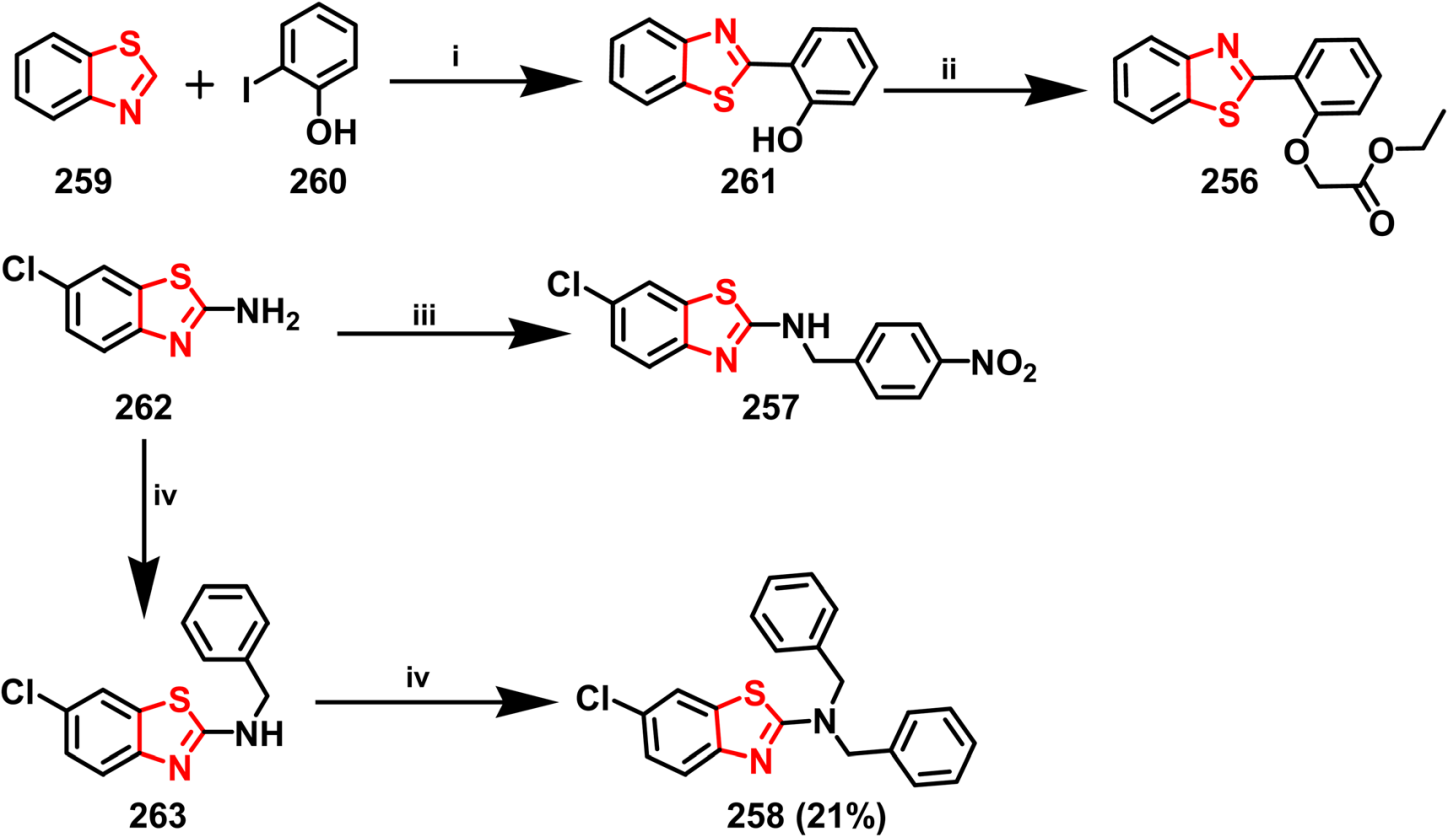
Synthesis of benzothiazole derivatives 256, 257, and 258. Reagents and condition: (i) K_2_CO_3_, DMSO, stirring, 120 °C, 6 h; (ii) BrCH_2_COOC_2_H_5_, K_2_CO_3_, CH_3_CN, stirring, rt, 3 h; (ii) 4-nitrobenzyl bromide, K_2_CO_3_, CH_3_CN, reflux, 65 °C, 6 h; (iv) benzyl bromide, K_2_CO_3_, CH_3_CN, reflux, 65 °C, 6 h.

New benzothiazole derivatives 256, 257, and 258 were prepared as described in Scheme 58.^123^

**Fig. 58:**
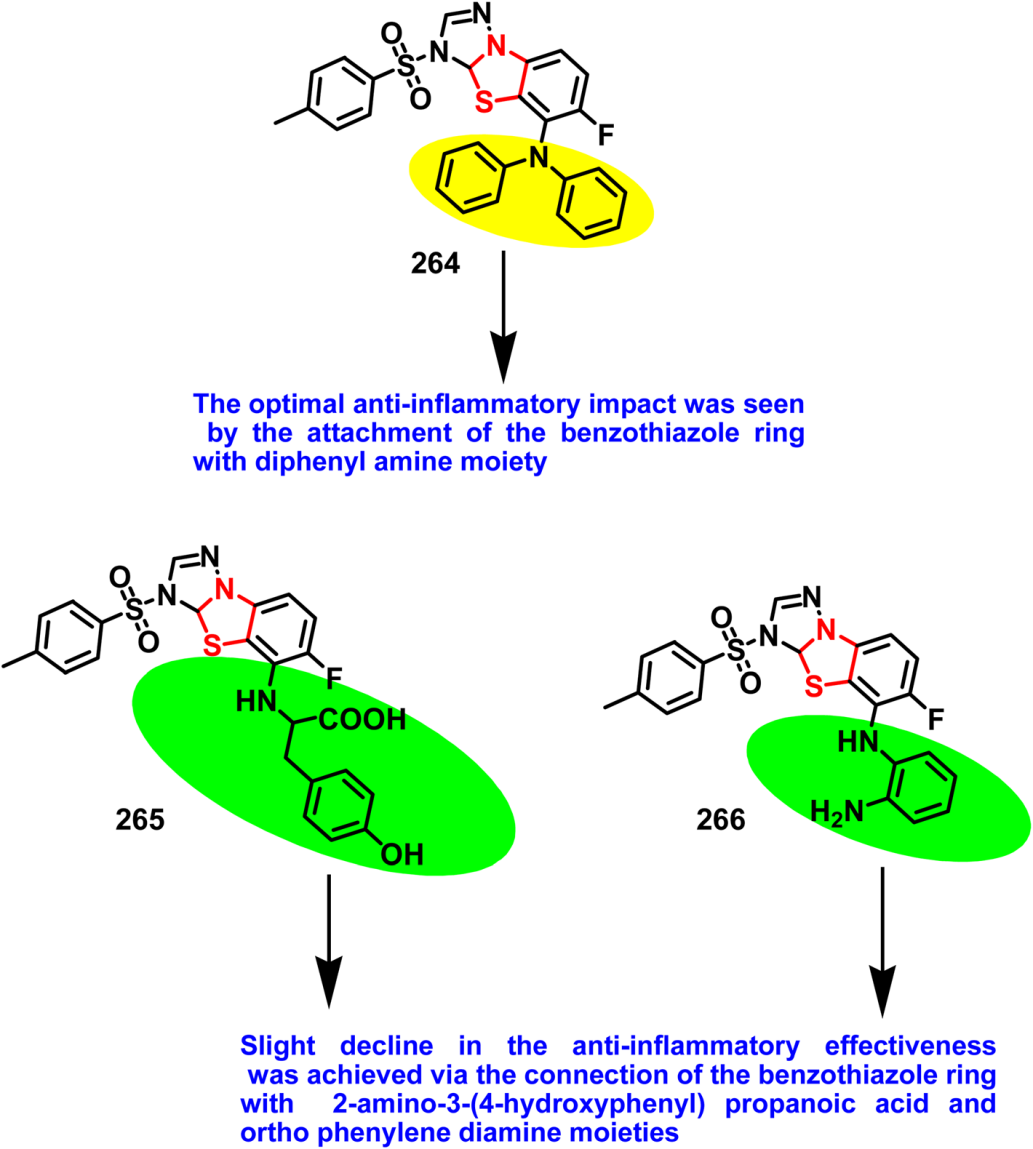
Structures of 6-fluorobenzothiazole fused with triazole 264, 265 and 266 as anti-inflammatory agents targeting the COX-2 enzyme.

New derivatives of 6-fluorobenzothiazole fused with triazole 264, 265 and 266 were discovered as promising *in vivo* anti-inflammatory agents with no acute toxicity (paw volume = 0.21, 0.31 and 0.31 mm, respectively), with respect to diclofenac acid (paw volume = 0.21 mm). The optimal anti-inflammatory impact was seen by the attachment of the benzothiazole ring with the diphenyl amine moiety. Moreover, a slight decline in the anti-inflammatory effectiveness was achieved *via* the connection of the benzothiazole ring with 2-amino-3-(4-hydroxyphenyl)propanoic acid and *ortho* phenylene diamine moieties. Derivatives 264, 265 and 266 displayed good binding affinities to the COX-2 active site ranging from −10.2 to −11.6 kcal mol^−1^, as compared to diclofenac (−8.4 kcal mol^−1^). Derivative 264 demonstrated a H-bond between the F atom and His207, besides hydrophobic interactions with Val291, Leu294, His214. Furthermore, derivative 265 displayed three H-bonds between OH, S=O and C=O groups with Tyr136, Gln461 and Gly135, respectively, as well as hydrophobic interactions with Pro153 and Leu152, while derivative 266 presented two H-bonds between S=O and NH_2_ with Arg44 and Arg469, respectively. Further, hydrophobic interactions were created with Pro153, Lys468 and Cys47 (ref. 124) (Fig. 58).

**Scheme 59:**
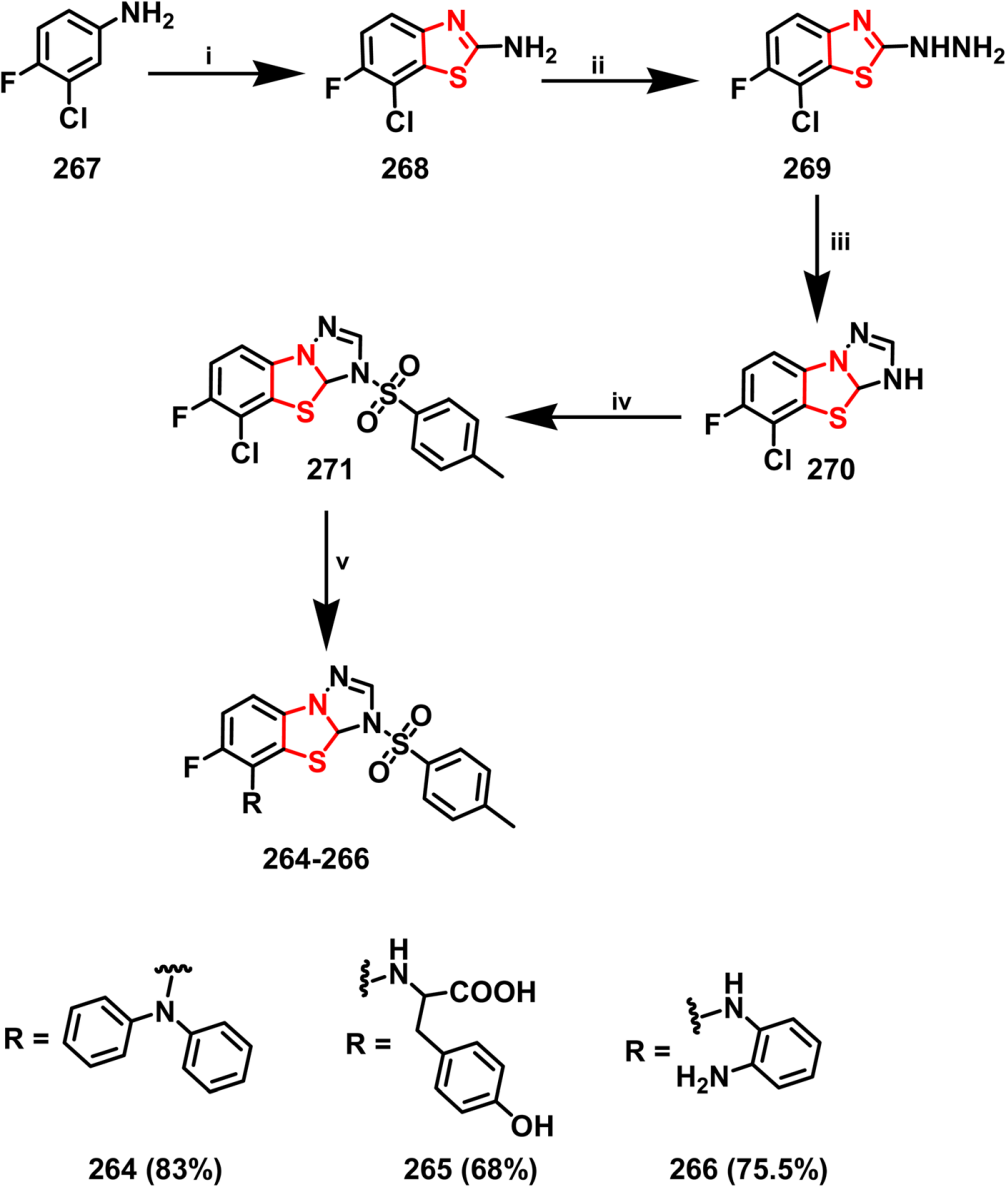
Synthesis of 6-fluorobenzothiazole fused with triazoles 264, 265 and 266. Reagents and conditions: (i) KSCN, Br, CH_3_COOH, stirring, RT; (ii) ethylene glycol, NH2NH2·H_2_O, Conc. HCl, reflux, 3 h; (iii) HCOOH, K_2_CO_3_, reflux, 2 h; (iv) *p*-toluene sulfonamide, pyridine, reflux, 2 h; (v) primary and secondary aromatic amines, DMF, reflux, 2 h.

New derivatives of 6-fluorobenzothiazole fused with triazole 264, 265 and 266 were synthesized, as illustrated in Scheme 59.^124^

**Fig. 59:**
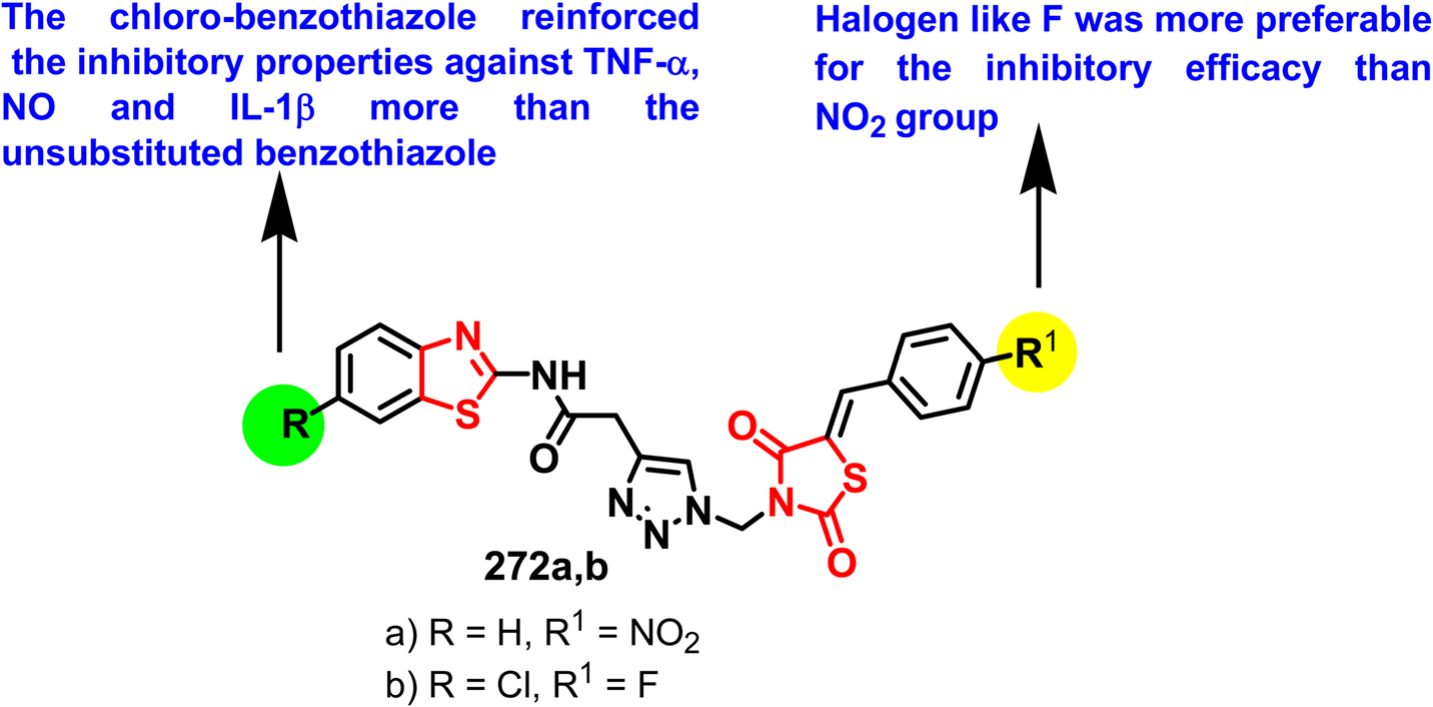
Structures of benzothiazole-thiazolidinedione-triazole hybrids 272a, b as anti-inflammatory agents targeting various inflammatory mediators.

The anti-inflammatory efficiency of benzothiazole-thiazolidinedione-triazole hybrids was evaluated in RAW 264.7 macrophages activated with LPS. Derivatives 272a, b displayed considerable dose-dependent suppression of various inflammatory mediators, such as TNF-α (inhibition = 48.3% and 65%, respectively), NO (inhibition = 86% and 92%, respectively), IL-1β (inhibition = 57.8% and 61.1%, respectively) and IL-6 (inhibition = 44 and 40%, respectively) at 10 µM. It was noticed that chloro-benzothiazole reinforced the inhibitory properties against TNF-α, NO and IL-1β more than the unsubstituted benzothiazole, whereas the substitution of the phenyl ring linked to the thiazolidinedione with halogens such as F was more preferable for the inhibitory efficacy than the NO_2_ group. Furthermore, derivative 272b displayed diminished skin permeability (−2.74) and water solubility (−4.192), indicating challenges in formulation and transdermal administration. Otherwise, it disclosed prominent human intestinal absorption (100%) and mild Caco-2 permeability (0.614), suggesting good oral bioavailability. Due to its P-gp substrate and inhibitor properties, its distribution may be affected, and drug–drug interactions are possible. It may be appropriate for CNS-targeted therapies due to its low volume of distribution (−0.435) and promising BBB permeability (−0.894). Significant medication interactions are less likely because it demonstrated suppression potency against CYP2C9 and CYP2C19 but was not a potent inhibitor of other essential CYP450 enzymes. Its poor overall clearance and weak CYP3A4 metabolism result in an extended half-life. According to toxicity anticipation, derivative 272b is reasonably safe for additional development because it is not mutagenic, non-sensitizing to the skin and non-cardiotoxic^125^ (Fig. 59).

**Scheme 60:**
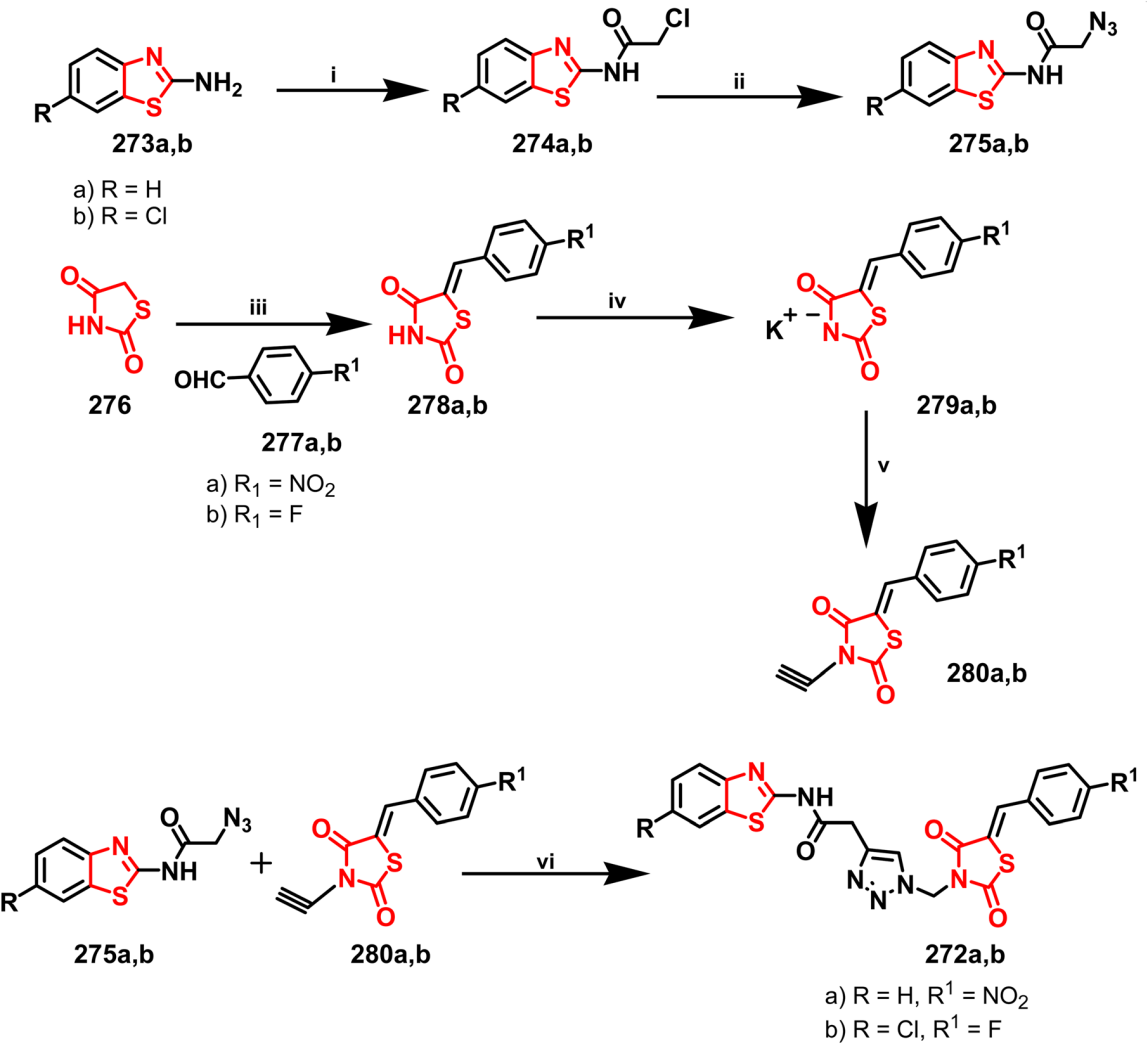
Synthesis of the benzothiazole-thiazolidinedione-triazole hybrids 272a, b. Reagents and conditions: (i) TEA, DCM, rt.; (ii) NaN_3_, DMF, rt.; (iii) AcOH, NaOAc, reflux, 14 h; (iv) EtOH, KOH, reflux, 2 h; (v) DMF, rt., overnight; (vi) l-ascorbic acid, CuSO_4_·5H_2_O, DMF/H_2_O, reflux, 12 h.

Benzo[*d*]thiazol-2-amines 273a, b were treated with chloroacetyl chloride to produce the intermediates 274a, b, which were then reacted with sodium azide to obtain derivatives 275a, b. Additionally, thiazolidinedione 276 undergoes Knoevenagel condensation with different benzaldehydes 277a, b, and subsequently, the production of salt 279a, b, and then *N*-alkylation with propargyl bromide to obtain derivatives 280a, b occur. The final coupling of derivatives 275a, b and 280a, b was accomplished *via* the copper-catalyzed ‘click’ reaction to afford benzothiazole-thiazolidinedione-triazole hybrids 272a, b (Scheme 60).^125^

## 3 Conclusion

This review mainly focuses on thiazole derivatives that were developed between 2020 and 2025 and had prominent anti-inflammatory properties. It was disclosed that the conjugation of the thiazole scaffold with various functional moieties such as hydrazine, hydrazone, amide, amine and imine groups enhanced the anti-inflammatory effects with promising suppression efficacy against 5-LOX and COX-2 enzymes as well as varied inflammatory cytokines such as TNF-α, IL-8 and IL-6. Furthermore, the conjugation of the thiazole scaffold with different five-membered heterocyclic rings including pyrazole, imidazole and triazole, in addition to six-membered heterocyclic rings such as pyridine or pyrimidine as well as bicyclic rings such as indole, reinforced the anti-inflammatory effectiveness. Moreover, thiazolidinone and benzothiazole-based derivatives demonstrated noticeable anti-inflammatory efficiency targeting COX-2, 15-LOX, 5-LOX, sEH and mPGES-1 enzymes and various inflammatory mediators such as IFN-γ, TNF-α, IL-6, IL-1β and NO. This review has addressed the biological activities, SARs, synthetic routes and docking studies of newly reported thiazole derivatives with eminent anti-inflammatory efficiency. We anticipate that this paper will provide further insights into the design of anti-inflammatory molecules to aid in the development of numerous anti-inflammatory drugs with high efficacy and low toxicity.

## Conflicts of interest

There are no conflicts to declare.

## Data Availability

No new data were used in this review article.
